# Chemoselective oxidation of aryl organoboron systems enabled by boronic acid-selective phase transfer†
†Electronic supplementary information (ESI) available: Experimental procedures, assay details and spectra, characterization data for all compounds. See DOI: 10.1039/c6sc04014d


**DOI:** 10.1039/c6sc04014d

**Published:** 2016-10-27

**Authors:** John J. Molloy, Thomas A. Clohessy, Craig Irving, Niall A. Anderson, Guy C. Lloyd-Jones, Allan J. B. Watson

**Affiliations:** a Department of Pure and Applied Chemistry , WestCHEM , University of Strathclyde , 295 Cathedral Street , Glasgow , G1 1XL , UK . Email: allan.watson.100@strath.ac.uk; b GlaxoSmithKline , Medicines Research Centre , Gunnels Wood Road , Stevenage , SG1 2NY , UK; c School of Chemistry , University of Edinburgh West Mains Road , Edinburgh , EH9 3JJ , UK

## Abstract

We report the direct chemoselective Brown-type oxidation of aryl organoboron systems containing two oxidizable boron groups. Basic biphasic reaction conditions enable selective formation and phase transfer of a boronic acid trihydroxyboronate in the presence of boronic acid pinacol (BPin) esters, while avoiding speciation equilibria. Spectroscopic investigations validate a base-promoted phase-selective discrimination of organoboron species. This phenomenon is general across a broad range of organoboron compounds and can also be used to invert conventional protecting group strategies, enabling chemoselective oxidation of BMIDA species over normally more reactive BPin substrates. We also demonstrate the selective oxidation of diboronic acid systems with chemoselectivity predictable *a priori*. The utility of this method is exemplified through the development of a chemoselective oxidative nucleophile coupling.

## Abstract 2

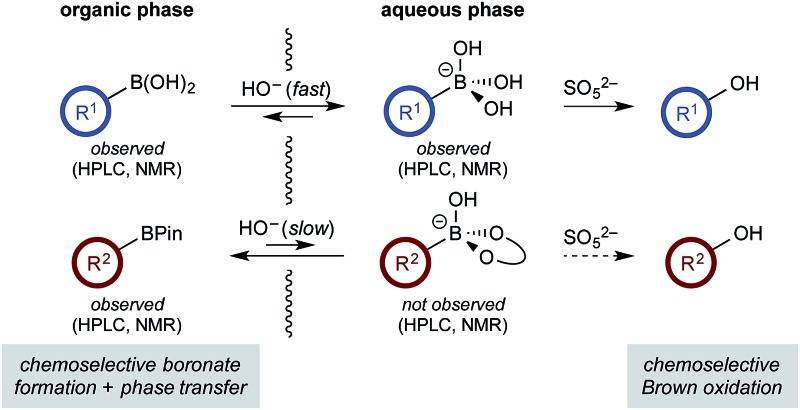
Chemoselective boronate formation and phase transfer allows chemoselective Brown oxidation of boronic acids in the presence of boronic esters.

## Introduction

**Scheme 1:**
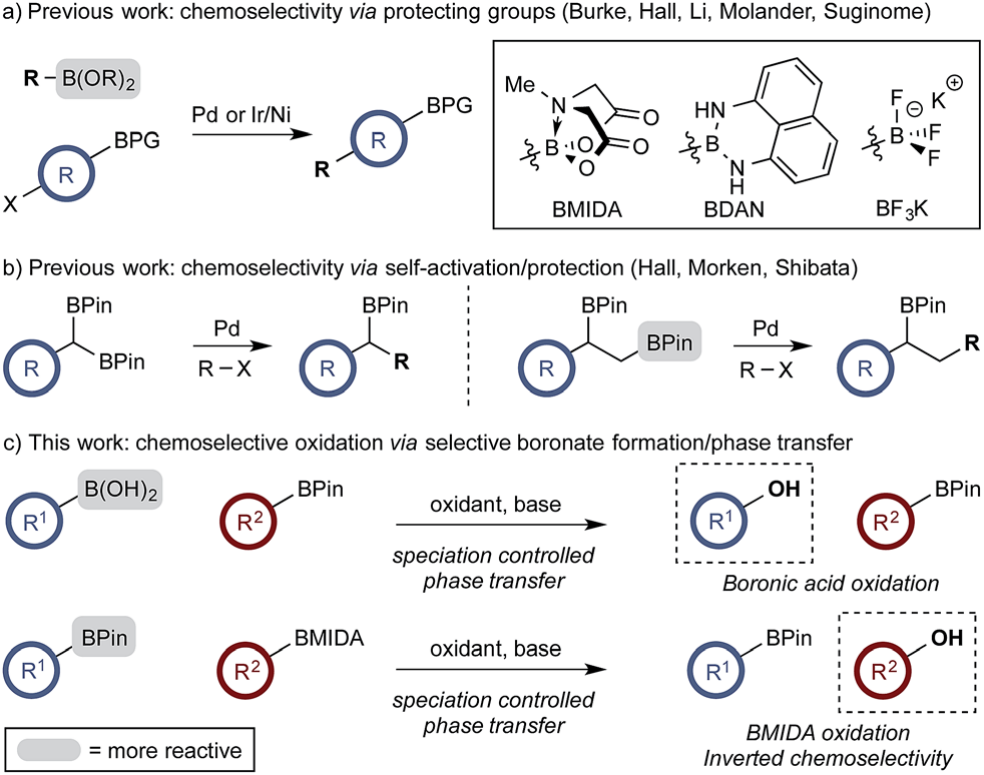
Chemoselective reactions of diboron systems.

The use of di- and multi-boron containing systems has become a powerful approach for the rapid synthesis of highly functionalized molecules from readily accessible starting materials.^1,2^ Chemoselectivity in these systems is currently achieved using only two approaches. Firstly, B-protecting groups render a specific boron unit unreactive under the prevailing reaction conditions. Widely used B-protecting groups include *N*-methyliminodiacetic acid (MIDA) esters,^3^ diaminonaphthalene (DAN)-based aminoboranes,^4^ and potassium organotrifluoroborates (RBF_3_K) (Scheme 1a).^5–7^ Secondly, self-activation/protection mechanisms allow discrimination of geminal and vicinal diboron compounds, enabling chemoselectivity within superficially equivalent systems (Scheme 1b).^8–10^

B-protecting groups are the most widely adopted strategy and are compatible with sp, sp^2^, and sp^3^ organoborons,^1–7^ while self-activation/protection is only applicable with sp^3^ organoborons.^1,8,9^ Accordingly, chemoselectivity within systems containing more than one aryl organoboron compound is currently only achievable by employing a suitable protecting group strategy. Based on the broadly similar reactivity profiles of boronic acids and esters,^11^ and the added complication of speciation equilibria,^11–13^ establishing chemoselective control within mixed organoboron systems represents a significant challenge. However, the identification of new chemoselective control mechanisms would be a fundamental advance, enabling the design and development of new synthetic methods for systems containing more than one reactive organoboron compound.

Here, we establish a new method for achieving chemoselectivity within systems containing two unprotected aryl organoboron compounds. Specifically, we show that chemoselective oxidation of aryl boronic acid/BPin systems can be achieved by selective boronate phase transfer while controlling potential solution speciation processes (Scheme 1c). In addition, we show that this approach can formally invert conventional chemoselectivity profiles using established MIDA protecting group chemistry. Spectroscopic investigations of the biphasic reactions provide insight into the mechanism by which chemoselectivity is achieved and how this may be predicted *a priori*.

## Results and discussion

To probe chemoselectivity in systems containing two non-protected aryl organoboron compounds we selected a workhorse reaction. The Brown oxidation of an organoboron compound to the corresponding alcohol or phenol is a fundamental method within the synthetic organic chemistry toolbox.^11,14^ Boronic acids and esters are typically rapidly and indiscriminately oxidized and, consequently, chemoselective oxidation of a system containing two reactive organoboron compounds is unknown.

**Scheme 2:**
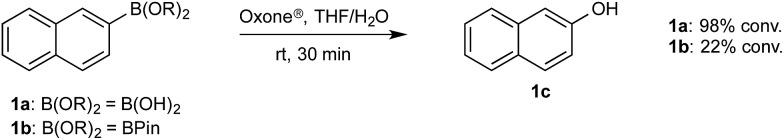
Oxidation of **1a** and **1b** using Oxone®.

**Chart 1:**
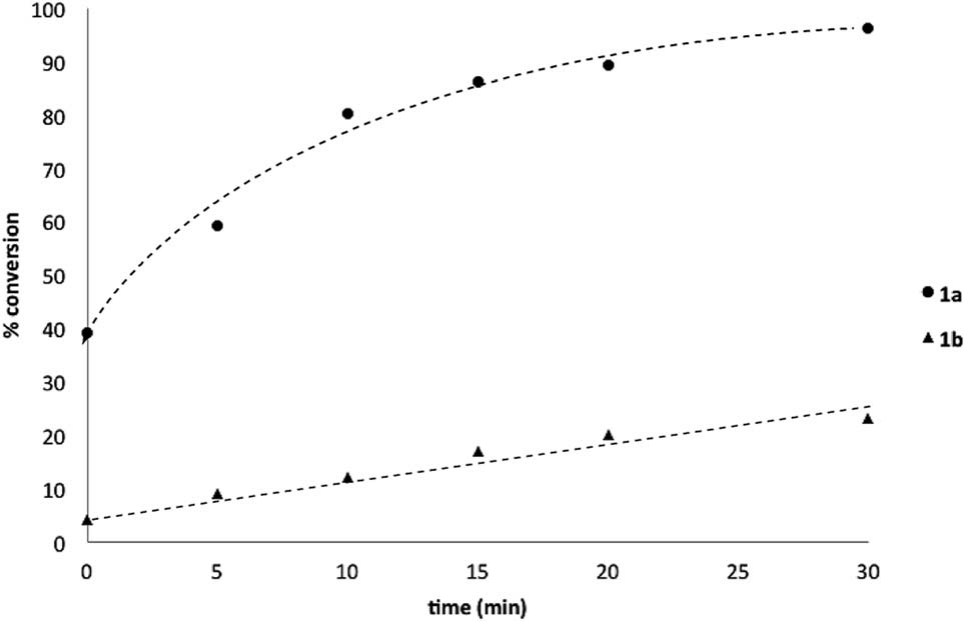
Oxidation of **1a** and **1b** using Oxone® under biphasic reaction conditions (THF/H_2_O) over 30 min at 350 rpm (*vide infra*). Determined by HPLC using an internal standard, see ESI.† §

In the general sense, small differences in reactivity of boronic acids and esters have been observed, albeit in non-competitive systems.^15^ Accordingly, to initiate this study, we examined the oxidation of naphthyl boronic acid **1a** and BPin ester **1b** under a variety of reaction conditions.‡
‡The following numbering key has been used throughout: R(BOH)_2_ = **Xa**; RBPin = **Xb**; ROH = **Xc**; RB(OH)_3_^–^ = **Xd**; RBPin(OH)^–^ = **Xe**; RBMIDA = **Xf**. Common oxidants such as H_2_O_2_, NaBO_3_, *m*-CPBA provided an uncontrollable oxidation from which no useful rate discrimination was observed (a range of oxidants and reaction conditions were surveyed, see ESI†). However, milder oxidants were useful and, in particular, a small rate difference favoring a more rapid oxidation of **1a** was found using Oxone® under biphasic reaction conditions (Scheme 2 and Chart 1).§
§Line added as a visual aid – no function has been fitted. Specifically, the oxidation of **1a** appeared to show a significant conversion in the burst phase while the oxidation of **1b** exhibited a more linear profile.

Based on the observed reactivity profiles in the non-optimized, non-competitive system, we considered it might be possible to leverage chemoselectivity in the corresponding mixed system (*i.e.*, containing both a boronic acid and BPin ester). However, translating these reaction conditions to a model system consisting of **1a** and BPin **2b** provided high conversion but with only trace levels of chemoselectivity (Table 1, entry 1).

**Table 1:** Chemoselective oxidation of B(OH)_2_ **1a** *vs.* BPin **2b**: reaction optimization

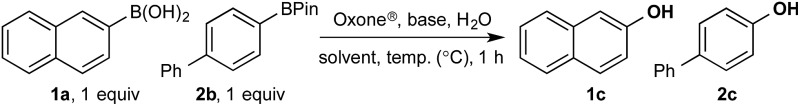				
Entry	Base	Temp. (°C)	Solvent	Conv. (**1c** : **2c**)^*a*^
1	—	20	THF/H_2_O (1 : 1)	95% (1.1 : 1)
2	—	20	THF	0%
3	K_3_PO_4_	20	THF	0%
4	K_3_PO_4_	20	THF/H_2_O (1 : 1)	54% (13.5 : 1)
5	K_3_PO_4_	60	THF/H_2_O (1 : 1.5)	81% (18 : 1)
**6**	**K**_**3**_ **PO**_**4**_	**70**	**CPME/H**_**2**_ **O (1** **:** **1.5)**	**100% (>99** **:** **1)**

^*a*^Determined by HPLC analysis using an internal standard. See ESI.†

In a purely organic medium (THF), no conversion was observed either in the absence or presence of base (entries 2 and 3 – a range of bases was evaluated, see ESI†), likely due to the poor solubility of Oxone®.^16^ However, upon addition of K_3_PO_4_ to the original biphasic system (*i.e.*, entry 1), we immediately noted moderate conversion, now with significant levels of chemoselectivity for the desired oxidation of **1a** (entry 4). A systematic evaluation of the reaction medium composition and temperature (see ESI†) revealed that conversion and chemoselectivity could both be improved using additional H_2_O at 60 °C (entry 5). A solvent survey revealed CPME as the optimum organic phase that allowed quantitative oxidation of the boronic acid and with very high levels of chemoselectivity at 70 °C under basic biphasic reaction conditions (entry 6 – a range of solvents was evaluated, see ESI†).^16^

### Determination of the origin of chemoselectivity

Basic biphasic reaction conditions allow chemoselective oxidation of boronic acid **1a** over BPin **2b**. While a small difference in rate using **1a** and **1b** was observed in the non-competitive system (Chart 1), oxidation was non-selective in the equivalent mixed organoboron system (Table 1, entry 1), suggesting that kinetic discrimination based on reactivities of the organoboron reagents with the oxidant was not the origin of the observed selectivity.

**Scheme 3:**
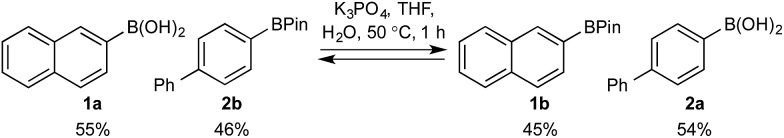
Speciation equilibria of **1a** and **2b** in a basic biphasic medium.

**Chart 2:**
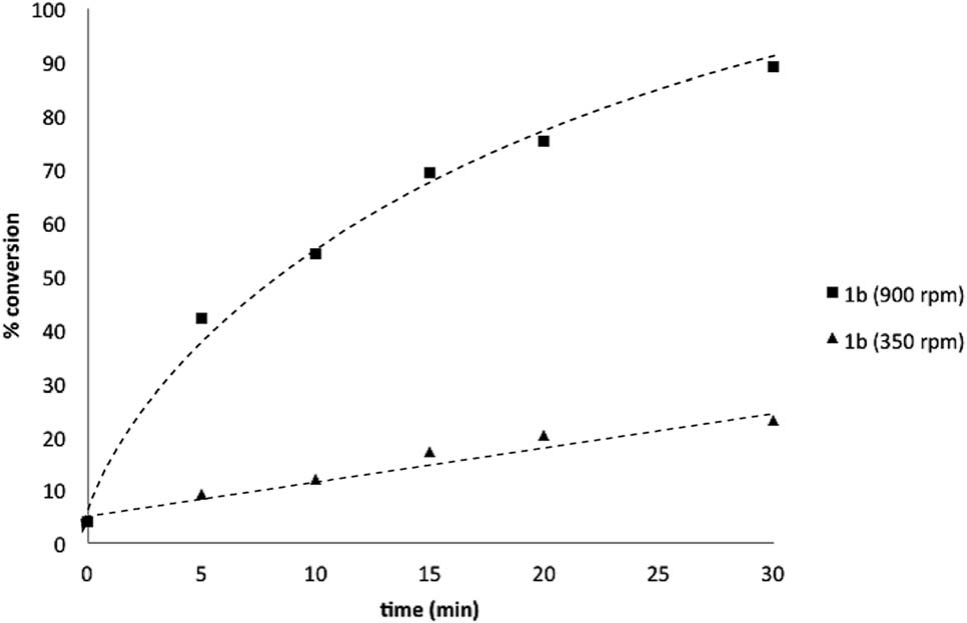
Oxidation of **1b** to **1c** using Oxone® under biphasic reaction conditions (THF/H_2_O) over 30 min at 350 and 900 rpm. Determined by HPLC using an internal standard, see ESI.† §

Several other data were notable: (i) Oxone® is poorly soluble in organic solvents^17^ and in the absence of H_2_O, no reaction was observed (Table 1, entries 2 and 3) suggesting a phase transfer process; (ii) speciation behavior of **1a** and **2b** was observed in similar basic biphasic media resulting in pinacol transfer to produce a mixture of **1a**, **1b**, **2a**, and **2b** in approx. 1 : 1 : 1 : 1 ratio (Scheme 3), accordingly, pinacol exchange is avoided under the optimized reaction conditions;^11–13,18^ (iii) chemoselectivity counter-intuitively increased with increasing temperature (Table 1); and (iv) shearing profoundly impacted the chemoselectivity of oxidation with high stirring rates resulting in lower chemoselectivity and *vice versa*. The impact of stirring rate was clearly seen in the change of reaction profile of BPin oxidation where increasing the stirring rate changed the reaction profile from linear at 350 rpm to exhibiting a burst phase at 900 rpm similar to the oxidation of **1a** (Chart 2).^19^

In relation to speciation (Scheme 3), full equilibration was observed to occur in *ca.* 1 h. Since the oxidation reaction also proceeds to completion in 1 h, we surmise that chemoselectivity is aided by Le Chateliers's principle, *i.e.*, consumption of **1a** *via* oxidation inhibits diol exchange and enforces high levels of chemoselectivity.

Based on all of the above, we suspected that oxidation was taking place *via* a phase transfer process where the boronic acid was selectively transported to and oxidized in the aqueous phase with the equivalent process for the BPin ester much slower in comparison.

Hall has shown that various polyols can be used to stoichiometrically transfer boronic acids to an aqueous phase as their boronate derivatives to allow purification by phase separation^20^ as well as providing a method for bioconjugation.^21^ No such phase-transfer catalyst was employed in the present oxidation; however, boronates are considerably more soluble in aqueous media than organic,^10^ suggesting chemoselective boronate formation could be taking place (boronic acid over BPin)^22^ while avoiding speciation processes in the basic biphasic medium. Boronic esters are more Lewis acidic than boronic acids;^23^ therefore, selective boronic acid trihydroxyboronate formation must be under kinetic control – this is typically a very rapid (practically barrier-less) process.^24^ To confirm this hypothesis, we undertook detailed analysis of the basic biphasic reaction mixture.

#### 1. HPLC analysis

The organic and aqueous phases of various relevant biphasic mixtures were analyzed by HPLC using a calibrated internal standard to allow quantitative determination of phase distribution (Table 2).

**Table 2:** Phase distribution of **1a** and **2b** in the presence of relevant inorganics and with temperature variation

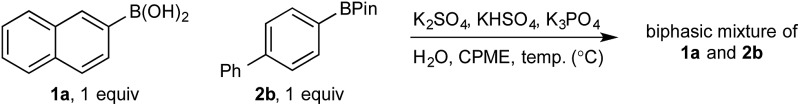				
Entry	Inorganics	Temp. (°C)	Organic : aqueous^*a*^ (%)	
			**1a**	**2b**
1	—	20, 50, 70	>99 : 1	>99 : 1
2	K_3_PO_4_	20	54 : 46	>99 : 1
3	K_3_PO_4_	50	46 : 54	96 : 4
4	K_3_PO_4_	70	29 : 71	98 : 2
5	KHSO_4_, K_2_SO_4_	20	>99 : 1	>99 : 1
6	KHSO_4_, K_2_SO_4_	50	>99 : 1	>99 : 1
7	KHSO_4_, K_2_SO_4_	70	98 : 2	>99 : 1
8	K_3_PO_4_, KHSO_4_, K_2_SO_4_	20	67 : 33	>99 : 1
9	K_3_PO_4_, KHSO_4_, K_2_SO_4_	50	59 : 41	>99 : 1
10	K_3_PO_4_, KHSO_4_, K_2_SO_4_	70	54 : 46	>99 : 1

^*a*^Determined by HPLC analysis using an internal standard. See ESI.†

In the absence of any inorganics, both **1a** and **2b** were confined to the organic phase (entry 1). However, addition of K_3_PO_4_ immediately distorted this distribution, with *ca.* 1 : 1 distribution of **1a** in each phase but with no effect on the distribution of **2b** (entry 2). The concentration of **1a** in the aqueous phase increased with temperature, reaching *ca.* 70% at the optimum reaction temperature of 70 °C, with the distribution of **2b** again remaining unchanged throughout (entries 2–4). Addition of Oxone®-relevant inorganics (without the active oxidant, KHSO_5_)^25^ had no effect on the distribution of either **1a** or **2b** at any temperature, with similar results to that observed in the absence of any inorganics (entries 5–7 *vs.* entry 1). In the presence of K_3_PO_4_, KHSO_4_, and K_2_SO_4_, **1a** was once again observed to distribute in both phases, up to *ca.* 1 : 1 at 70 °C, while **2b** remained confined to the organic phase, even at elevated temperatures (entries 8–10). The lower concentration of **1a** in the aqueous phase in the presence of all inorganics may be attributable to buffering. Lastly, no speciation behavior (*i.e.*, diol transfer, see Scheme 3) was observed throughout. However, it should be noted that speciation (diol transfer) was observed when mixtures of **1a** and **2a** were left for extended time periods at elevated temperatures.

#### 2. NMR analysis

While HPLC analysis allowed quantification of **1a** and **2b** in each phase, determination of speciation (neutral and charged species) was not possible; *i.e.*, whether **1a**/**2b** existed as the neutral boronic acid/ester species or their cognate hydroxyboronates in the aqueous phase and the relationship, if any, between these in the presence of the added inorganics. A complementary analysis was therefore conducted using a series of biphasic NMR experiments in which a single phase could be observed in isolation (see ESI for full details†).

Initial control experiments were informative and provided further empirical evidence to support selective hydroxyboronate formation. Specifically, while trihydroxyboronate **1d** could be formed using aq. K_3_PO_4_, BPin hydroxyboronate **2e** was not observed under similar conditions. Indeed, **2e** was only observed upon treatment with aq. KOH, which also led to extensive hydrolysis,^26^ generating the corresponding boronic acid **2a** and, consequently, its trihydroxyboronate derivative. This supported the hypothesis that under the reaction conditions, boronic acid trihydroxyboronates may be formed selectively, thereby allowing selective phase transport and subsequent chemoselective oxidation.

**Scheme 4:**
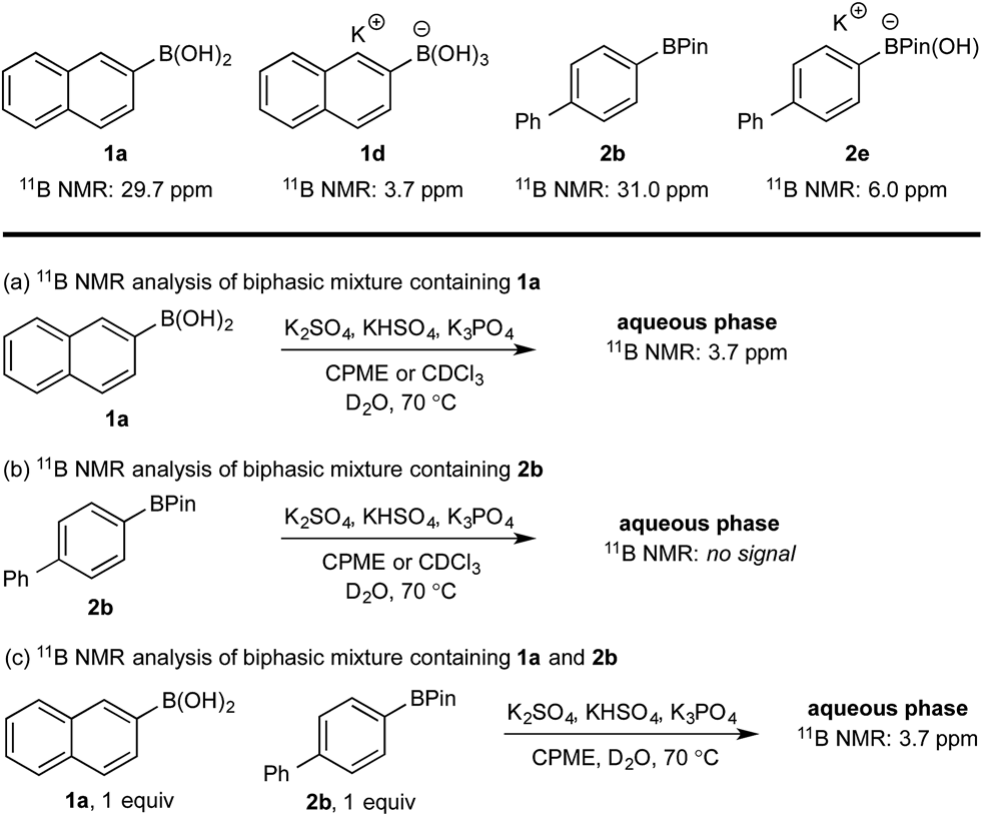
^11^B NMR analysis of mono- and diboron systems of **1a** and **2b** under representative biphasic conditions.

^11^B NMR analysis of the aqueous phase of the biphasic monoboron system containing **1a** and relevant inorganics (again without KHSO_5_) showed the presence of a single boron species with a resonance at 3.7 ppm, consistent with trihydroxyboronate **1d** (Scheme 4a) while no signal was detected in the aqueous phase for the equivalent experiment using only **2b** (Scheme 4b). Analysis of the corresponding model system containing both **1a** and **2b** revealed a single signal in the aqueous phase at 3.7 ppm, consistent with **1d** (Scheme 4c). This analysis agreed with the HPLC data (Table 2) and also supported selective phase transport of **1a** to the aqueous phase as its trihydroxyboronate derivative, **1d**.^27^ No charged species (**1d** or **2e**) or anhydride formation were observed in a complementary analysis of the organic phase – only the neutral species (**1a** and **2b**) were observed.

**Scheme 5:**
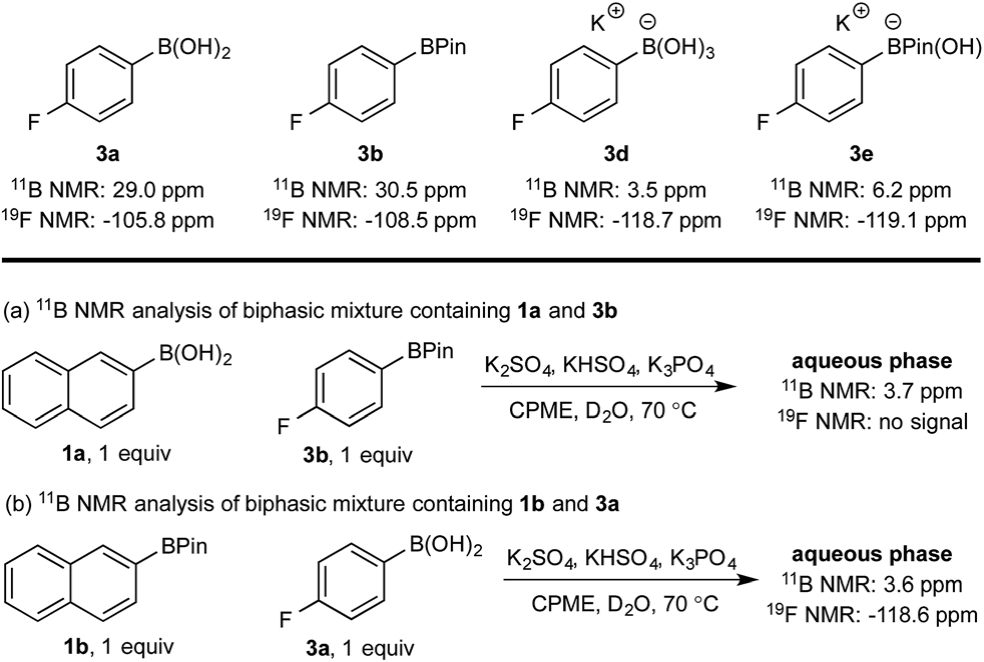
^11^B and ^19^F NMR analysis of mono-fluorinated diboron systems under representative biphasic conditions.

Hydroxyboronates **1d** and **2e** are distinguishable by ^11^B NMR (see ESI†) and the assignment of the observed ^11^B NMR signal at 3.7 ppm was attributed to **1d**. However, it is conceivable that *in situ* hydrolysis of **2b** could occur to deliver **2a** and ultimately its trihydroxyboronate derivative (**2d**), which has a similar ^11^B NMR resonance to **1d** (3.6 ppm, see ESI†). The **2d** signal may be obscured by **1d**, preventing detection at low concentration. To ensure a robust assignment, we analyzed two mono-fluorinated diboron systems by ^11^B and ^19^F NMR analysis (Scheme 5).

^11^B and ^19^F NMR analysis of the aqueous phase of the system containing **1a** and **3b** revealed a single ^11^B NMR signal at 3.7 ppm, consistent with **1d**; no ^19^F NMR signals were detected (Scheme 5a). Conversely, analysis of the mixture of **1b** and **3a** showed one ^11^B NMR signal at 3.6 ppm and one ^19^F NMR signal at –118.6 ppm, both of which were consistent with trihydroxyboronate **3d** (Scheme 5b). Thus, these experiments support the hypothesis of a selective boronic acid trihydroxyboronate formation and that diol transfer is inhibited.

**Fig. 1:**
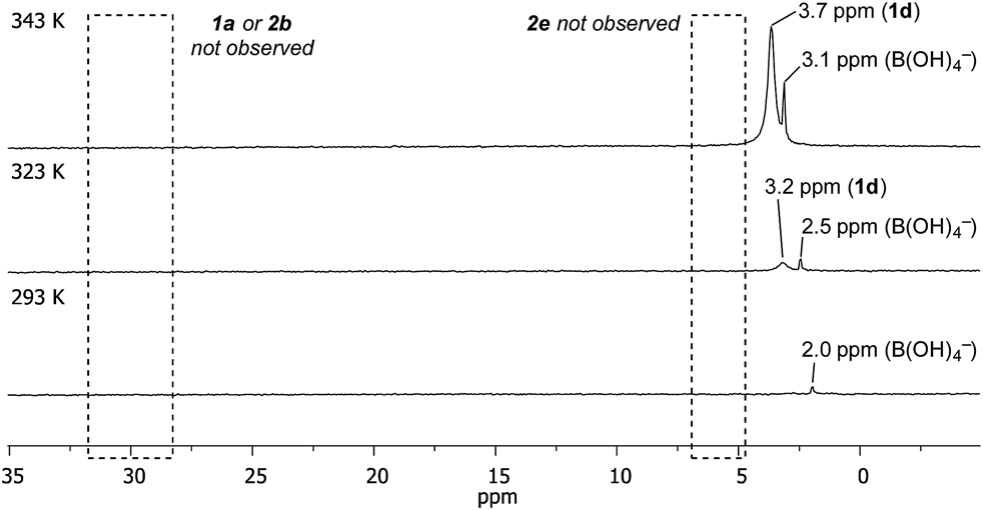
Temperature- and time-proportional concentration of **1d** in the aqueous phase by ^11^B NMR analysis.

Temporal profiling of the aqueous phase *via* variable temperature NMR provided further data to assist in explaining the observed trends (Fig. 1 – for temperature/temporal profiling of all systems, see ESI†). Consistent with the HPLC analysis (Table 2), variable temperature ^11^B NMR revealed that [**1d**] increased with temperature, with no detectable increase in [**1a**], [**2b**] or [**2e**].^28^ [**1d**] also increased over time (see ESI†). This combined HPLC and NMR data set assists with the interpretation of the non-intuitive temperature-proportional increase in chemoselectivity.^29^

**Scheme 6:**
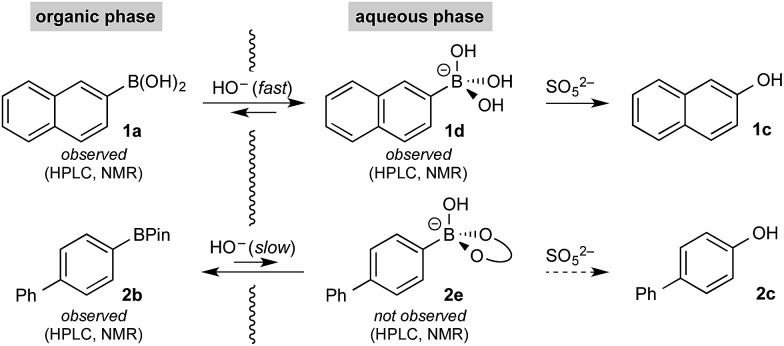
Spectroscopically observed organoboron species.

The rate of oxidation was found to be rapid in the burst phase (see Chart 1 and ESI†). Therefore, the rate determining process for oxidation under the developed biphasic reaction conditions appears to be phase transfer of the organoboron species to the aqueous phase, which is assisted by trihydroxyboronate formation. Since oxidation occurs exclusively in the aqueous phase and BPin hydroxyboronate formation was not observed under the reaction conditions, chemoselective oxidation is achieved since boronic acid phase transfer is significantly more favorable than BPin transfer (Scheme 6).

Interestingly, in the absence of the active oxidant (KHSO_5_), protodeboronation was observed to increase proportionally with both time and temperature giving the expected product B(OH)_4_^–^.^30,31^ In the oxidative system this was not particularly problematic, since the rate of oxidation was rapid. However, this has clear implications for transition metal catalysis using organoboron species under basic biphasic reaction conditions (*e.g.*, Suzuki–Miyaura cross-coupling) in which transmetallation proceeds *via* the neutral organoboron species^15^ and must engage a presumably largely organic phase-bound catalyst.

Lastly, formation of trihydroxyboronate from the boronic acid or hydroxyboronate from the BPin ester requires access to HO^–^. Boronic acids have typically greater aqueous solubility than the corresponding BPin.^11^ clog *P* calculations (see ESI†) indeed indicate greater aqueous solubility for boronic acids *vs.* BPins and this was also found for the corresponding boronate adducts. For example, boronic acid **1a** has clog *P* = 2.64 while BPin **1b** has clog *P* = 5.58. The corresponding boronates display the same trend with **1d** cLogP = 0.50 and **1e** clog *P* = 3.44. Accordingly, since no charged species were observed in the organic phase, we believe that selective ionization of the boronic acid occurs either at the organic/aqueous interface or in the aqueous phase following transfer of the neutral species due to its comparatively greater solubility.

### Generality of the chemoselective oxidation process

**Scheme 7:**
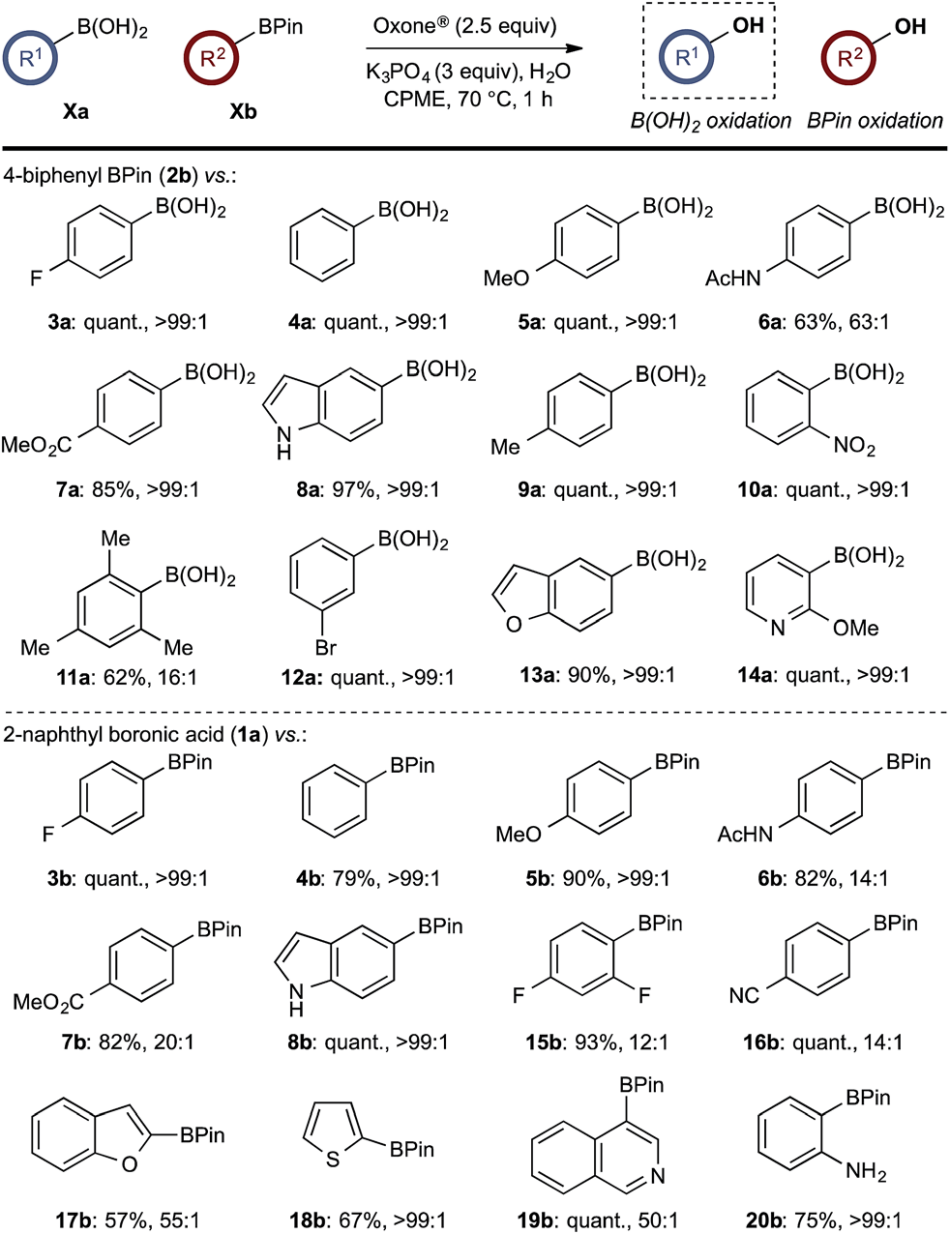
Chemoselective oxidation of aryl diboron system: B(OH)_2_ *vs.* BPin substrate scope. Ratio given for oxidation of **Xa** : **Xb**. Determined by HPLC, see ESI.†

With effective reaction conditions for this model system, the generality of the chemoselective oxidation was explored (Scheme 7).

The biphasic reaction conditions were found to be general across a wide variety of aryl boronic acid and BPin ester reaction partners. Conversion to products over a 1 h reaction time were generally high and the chemoselectivity for boronic acid oxidation was typically >20 : 1 and exclusively selective (>99 : 1) in many cases, regardless of functionality or regiochemistry and whether boronic acid or BPin (*e.g.*, **3a–8a** *vs.* **3b–8b**). Alkenyl boronic acids were less effective substrates, giving mixtures of products.

**Scheme 8:**
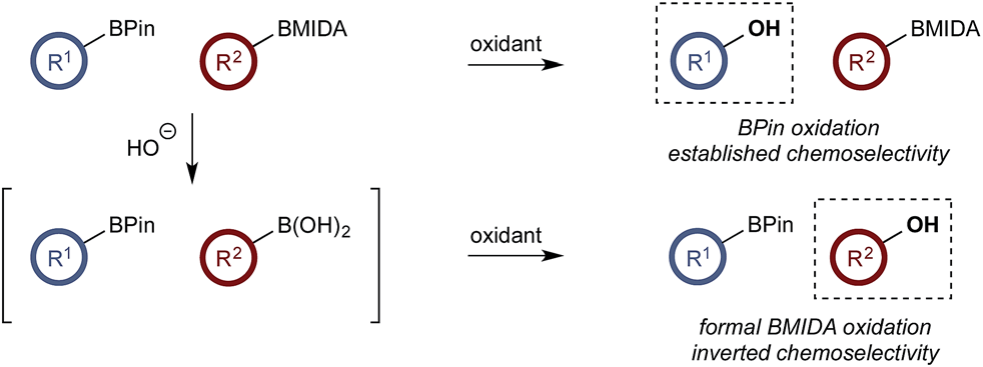
Inverting established chemoselectivity: oxidation of BMIDA in the presence of BPin.

With a framework for chemoselective oxidation of reactive diboron systems established, we sought to explore whether this biphasic protocol could challenge conventional reactivity profiles. BPin esters are typically readily oxidized in the presence of BMIDA esters.^13*d*^ However, we reasoned that it might be possible to reverse this profile and selectively oxidize the BMIDA component of a BMIDA/BPin aryl diboron system *via* speciation-controlled hydrolysis of the BMIDA^32^ and oxidation of the latent boronic acid (Scheme 8).

**Scheme 9:**
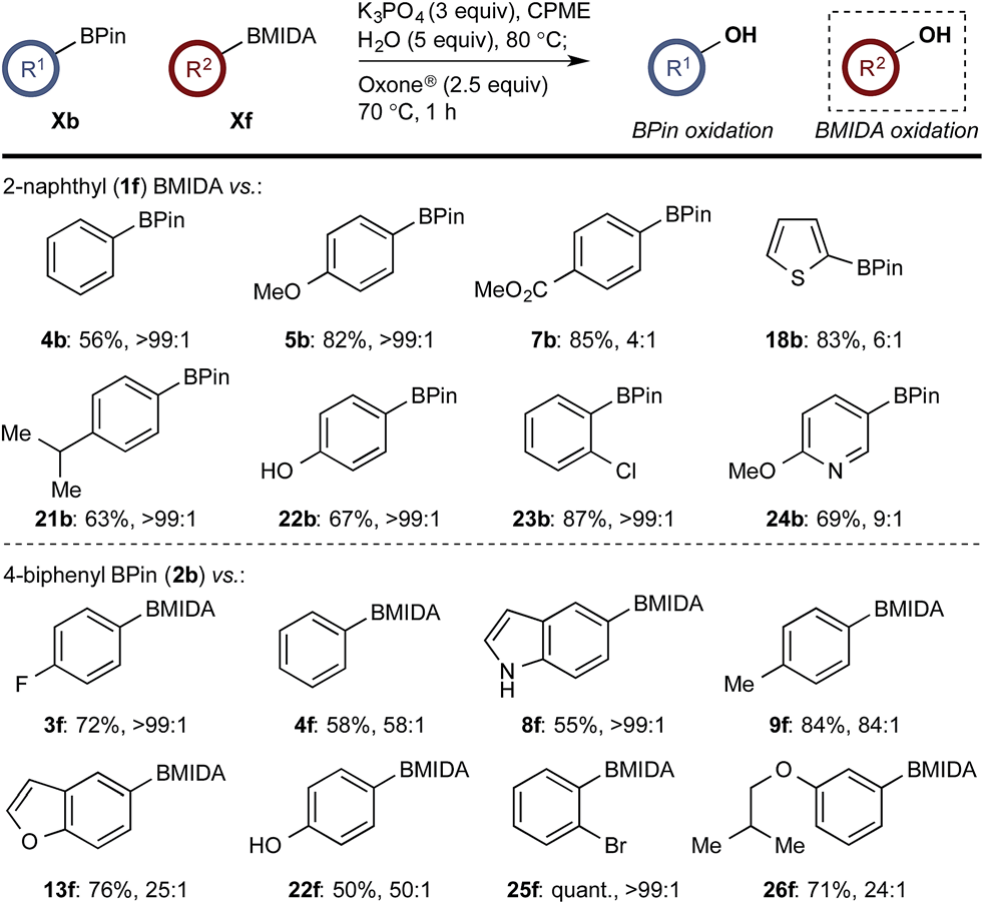
Chemoselective oxidation of aryl diboron system: BMIDA *vs.* BPin substrate scope. Ratio given for oxidation of **Xf** : **Xb**. Determined by HPLC, see ESI.†

In the event, heating the reaction mixture to 80 °C for 15 min in the absence of Oxone® provided a smooth hydrolysis, which avoided any diol equilibration, and returning to 70 °C before addition of the oxidant allowed chemoselective oxidation of ArBMIDA in the presence of ArBPin (Scheme 9).

Once more, the efficiencies and selectivities of the process were typically excellent, with some diminished selectivity observed using specific heterocyclic derivatives (*e.g.*, **18b**, **24b**). Addition of Oxone® after the hydrolysis event was necessary to avoid buffering of the basic medium. This buffering effect impeded the rate of hydrolysis providing sufficient time for equilibration and ultimately diminishing the chemoselectivity of the process.

**Scheme 10:**
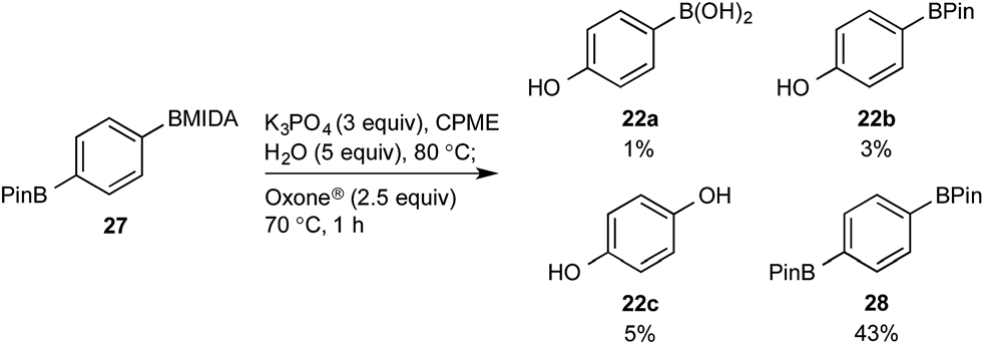
Attempted chemoselective oxidation of diboron compound **27**. Determined by HPLC, see ESI.†

This BMIDA oxidation process provided the opportunity to further confirm the hypothesis of the requirement to physically separate the two boron residues in order to achieve chemoselectivity. Diboron compound **27** (where both boron residues were located on the same aryl unit) was a very poor substrate that, under optimized conditions, delivered a mixture of the desired phenol **22b** as well as **22a** (the product of BPin oxidation and BMIDA hydrolysis), **22c** (the product of global oxidation), but mainly **28** (the product of equilibration) (Scheme 10).^32^

### Chemoselective oxidation of diboronic acid systems

**Scheme 11:**
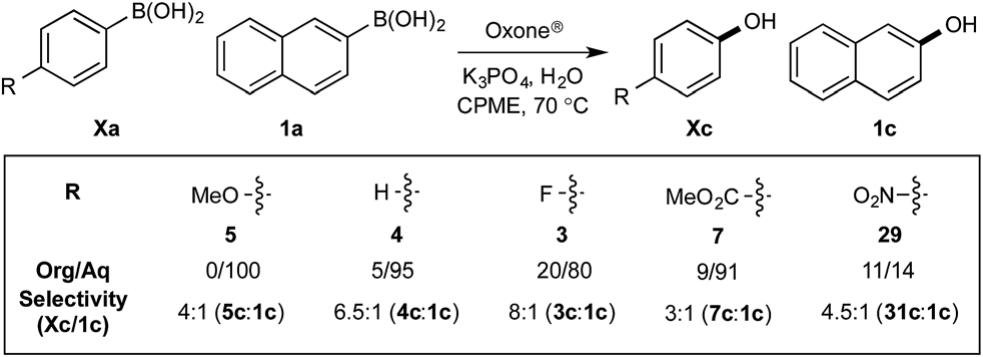
Phase distribution and chemoselective oxidation of electronically distinct aryl boronic acids *vs.* **1a**. Determined by HPLC, see ESI.†

Chemoselectivity in this system has been driven by competing boronate formation between dissimilar organoboron compounds (B(OH)_2_ *vs.* BPin). However, the formation of aryl boronates, and therefore aqueous solubility, is heavily influenced by the electronics of the aryl unit.^19^ Specifically, substitution on the aryl unit will influence the Lewis acidity of the boronic acid and can heavily influence the aqueous solubility. Based on this, we reasoned that chemoselective discrimination within a system containing two boronic acids might be achievable by exploiting electronic effects to drive competitive boronate formation and subsequent selective phase transfer. The relative chemoselectivity might then be gauged *a priori* by assessing the phase separation by HPLC or NMR. This proved to be feasible and was initially evaluated using several electronically distinct phenylboronic acids *vs.* 2-naphthyl boronic acid **1a** (Scheme 11).

Under representative reaction conditions in the absence of oxidant, phenylboronic acids **3a**, **4a**, **5a**, and **7a** were found to preferentially distribute to the aqueous phase while **1a** remained comparatively more organic phase-bound (**1a** org/aq average = 71/29). This translated to chemoselective oxidation of the phenyl boronic acid species over **1a** in all cases. In the case of the strongly electron-deficient boronic acid **29a**, protodeboronation occurred rapidly and phase distribution was less reliable as an indicator of selectivity. While the measured phase distribution allowed prediction of the favored oxidation, the exact ratio of oxidation products could not be extrapolated from this analysis. This phenomenon was also found to be transferable across a range of substrates (Table 3).

**Table 3:** Chemoselective oxidation of aryl boronic acids

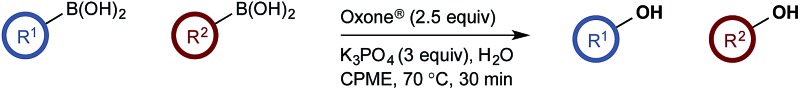				
Entry	R^1^B(OH)_2_	R^2^B(OH)_2_	Conv.^*a*^	Oxidation (R^1^ : R^2^)^*a*^ ^,^ ^*b*^
1	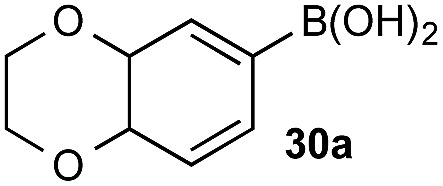	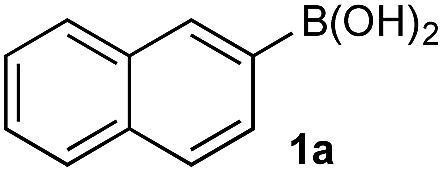	86%	4 : 1
2	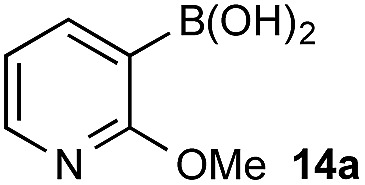	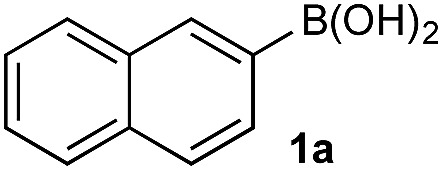	62%	3 : 1
3	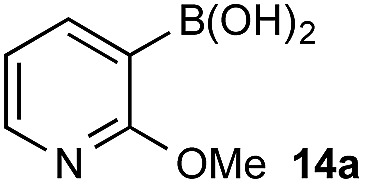	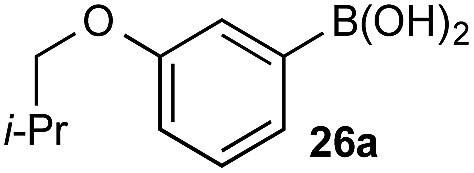	71%	3 : 1
4	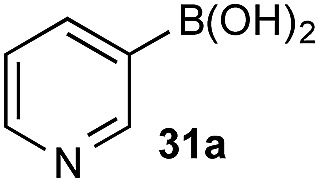	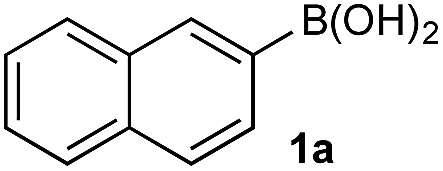	85%	2 : 1
5	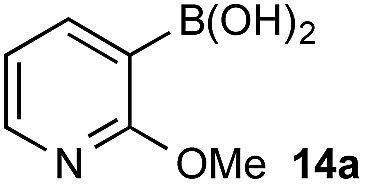	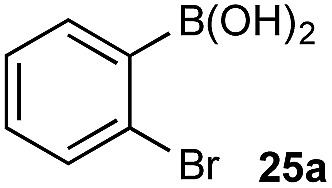	42%	2 : 1

^*a*^Determined by HPLC analysis using an internal standard. See ESI.†

^*b*^Ratio given for oxidation of R^1^-B(OH)_2_ : R^2^-B(OH)_2_.

The efficiencies and chemoselectivities of the process were not as pronounced as more substantially differentiated diboron systems (*e.g.*, B(OH)_2_ *vs.* BPin in the studies above); however, this represents the first chemoselective oxidation of two ostensibly equivalent boronic acid species based on subtle differences in the substitution of the pendant aryl unit.^33,34^

### Chemoselective oxidative nucleophile coupling

**Scheme 12:**
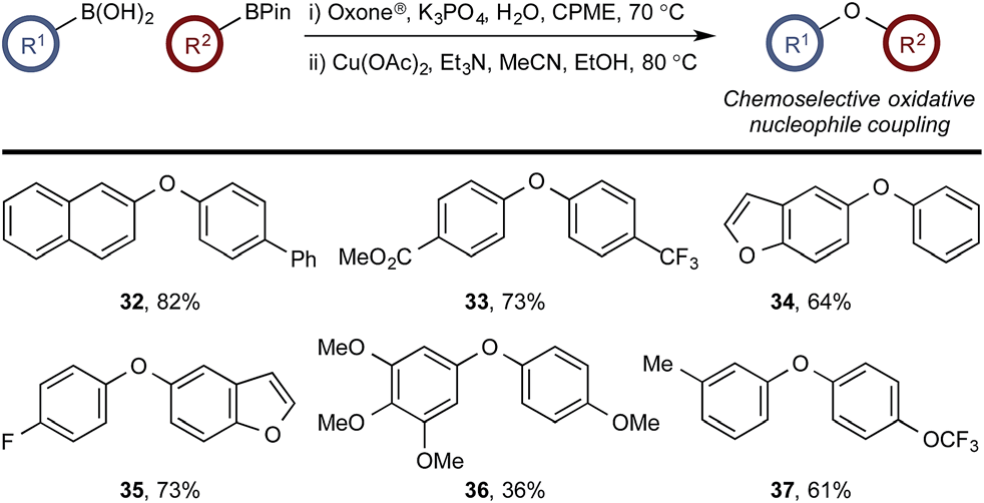
Chemoselective oxidative nucleophile coupling.

The medium-controlled chemoselective reaction manifold can potentially be leveraged to provide a number of enabling synthetic methods. As a demonstration, we have developed a chemoselective oxidative nucleophile coupling (Scheme 12).

Following chemoselective oxidation, a Chan–Evans–Lam etherification^35,36^ of the generated phenol with the remaining BPin can be effected. This process proceeds with high efficiency for the desired cross-coupled product with minimal homo-coupling detected. Biaryl ethers are prominent scaffolds in natural products, pharmaceuticals, agrochemicals, and materials,^37^ for example, the anticancer agents, including **36**.^38^ Modern catalysis methods, such as Ir-catalyzed C–H activation,^39^ have provided convenient methods for accessing borylated arenes with substitution patterns that are not readily accessible by other methods. As such, this chemoselective oxidative nucleophile coupling process provides a novel and step-efficient synthesis of valuable chemotypes from readily accessible precursors.

## Conclusions

In conclusion, chemoselective oxidation of boronic acid/BPin systems can be readily achieved in a basic biphasic reaction medium. Conventional protecting group strategies can be overturned to allow oxidation of BMIDA compounds in the presence of a normally more reactive BPin species. Spectroscopic investigations revealed that chemoselectivity is derived from a selective boronate formation and phase transfer of boronic acids to the aqueous phase. We have also shown that it is possible to chemoselectively oxidize a mixture of two boronic acids and predict the outcome of the reaction *a priori* by HPLC analysis of the phase distribution of the reacting partners. Lastly, the concept of chemoselectivity *via* medium control enabled the development of a chemoselective oxidative nucleophile coupling. The data in this study has significant ramifications for enabling chemoselectivity in non-protected organoboron systems as well as for the understanding of catalytic reactions of organoboron compounds in biphasic media.

## Abbreviations

CPME: Cyclopentyl methyl ether
DAN: Diaminonaphthalene
HPLC: High performance liquid chromatography
MIDA: *N*-Methyliminodiacetic acid/*N*-methyliminodiacetate
NMR: Nuclear magnetic resonance
Pin: Pinacolato
rt: Room temperature
