# Supplementary material for: Chemoselective oxidation of aryl organoboron systems enabled by boronic acid-selective phase transfer
Source: Chem Sci. 2016 Oct 27;8(2):1551–9. doi: 10.1039/c6sc04014d (PMC5452267; doi:10.1039/c6sc04014d)

Electronic Supplementary Material (ESI) for Chemical Science.
This journal is © The Royal Society of Chemistry 2016

Chemoselective oxidation of aryl organoboron systems enabled by boronic acid-selective phase transfer.

John J. Molloy,[a] Thomas A. Clohessy,[a,b] Craig Irving,[a] Niall A. Anderson,[b] Guy C. Lloyd-Jones[c] and Allan J. B. Watson[a]*

[a] Department of Pure and Applied Chemistry, WestCHEM, University of Strathclyde, 295 Cathedral Street, Glasgow, G1 1XL, UK.

[b] GlaxoSmithKline, Medicines Research Centre, Gunnels Wood Road, Stevenage, SG1 2NY, UK.

[c] School of Chemistry, University of Edinburgh West Mains Road, Edinburgh, EH9 3JJ, UK.

Email: allan.watson.100@strath.ac.uk

Contents

1. General

2. General Experimental Procedures

3. Oxidation of Monoaryl Boron Systems – Kinetic Study

    3.1. Oxidant Study

    3.2. Time Study

4. Reaction Optimization Data

    4.1. Boronic Acid Selective Oxidation

        4.1.1. Base and Water Study

        4.1.2. Oxidant Study

        4.1.3. Water Study

        4.1.4. Acetone Study

        4.1.5. Time Study

        4.1.6. Oxidant Equivalents Study

        4.1.7. Base Study

        4.1.8. Base Equivalents Study

        4.1.9. Temperature Study

        4.1.10. Solvent Study

    4.2. BMIDA Selective Oxidation

        4.2.1 Hydrolysis Solvent Study

4.2.2. Hydrolysis Time Study

4.2.3. Hydrolysis Temperature Study

5. Determination of the Origin of Chemoselectivity

5.1. Boronic Acid and BPin Equilibration Investigation

5.2. Shearing Effect Investigation

5.3. Determination of Phase Distribution – HPLC Analysis

5.4. Determination of Phase Distribution – NMR Analysis

5.4.1. Setup

5.4.2. Monoaryl Boron Systems

5.4.3. Diaryl Boron Systems

5.5. cLogP Parameters for Boron Species

6. Chemoselective Oxidation – Boronic Acid *vs.* BPin

7. Chemoselective Oxidation – BMIDA *vs.* BPin

8. Chemoselective Oxidation – Boronic Acid *vs.* Boronic Acid

8.1. Determination of Boronic Acid *vs.* Boronic Acid Phase Distribution – HPLC Analysis

8.2. Boronic Acid *vs.* Boronic Acid – Substrate Scope

9. Compound Characterization Data

9.1. Characterization Data for BMIDA Intermediates

9.2. Characterization Data for NMR analysis

9.3. Characterization Data for Oxidative Nucleophile Coupling

9.4. Assay Characterization Data

10. References

11. HPLC Retention Times and Conversion Factors of Products

12. HPLC Spectra

13. Appendices

1. General

All reagents and solvents were obtained from commercial suppliers and were used without further purification unless otherwise stated. Purification was carried out according to standard laboratory methods.[1]

1.1 Purification of Solvents

Dry THF and toluene were obtained from a PureSolv SPS-400-5 solvent purification system. These solvents were transferred to and stored in a septum-sealed oven-dried flask over previously activated 4 Å molecular sieves and purged with and stored under nitrogen. CH_2Cl_2, Et_2O, CPME, EtOAc, MeCN, 1,4-dioxane, 2-MeTHF, DMF, IPA, $CHCl_3$, and petroleum ether 40-60° for purification purposes were used as obtained from suppliers without further purification.

1.2 Drying of Inorganic Bases

K_3PO_4, K_2CO_3, and Cs_2CO_3 were dried in a Heraeus Vacutherm oven at 60 °C under vacuum for a minimum of 24 hours before use.

1.3 Experimental Details

Reactions were carried out using conventional glassware (preparation of intermediates) or in capped 5 mL microwave vials (for all other experiments excluding NMR study). Microwave vials were purchased from Biotage (2–5 mL Biotage Microwave Reaction Kit, catalogue number 351521). Magnetic stirrer bars were used as supplied in the Biotage Microwave Reaction Kit. The glassware was oven-dried (150 °C) and purged with N_2 before use. Purging refers to a vacuum/nitrogen-refilling procedure. Room temperature was generally *ca.* 20 °C. Reactions were carried out at elevated temperatures in a sand bath using a temperature-regulated hotplate/stirrer. Temperature quoted is a measurement of the sand bath heating block. Temperature-regulated hotplate/stirrers employed over the course of this study were either of the following: An IKA® RCT basic, a Heidolph MR 3004 safety, or Heidolph MR 3002.

1.4 Purification of Products

Thin layer chromatography was carried out using Merck silica plates coated with fluorescent indicator UV254. These were analyzed under 254 nm UV light or developed using potassium permanganate solution. Normal phase flash chromatography was carried out using ZEOprep 60 HYD 40-63 μm silica gel. Reverse phase flash chromatography was carried out using IST Isolute C18 cartridges.

1.5 Analysis of Products

Fourier Transformed Infra-Red (FTIR) spectra were obtained on a Shimadzu IRAffinity-1 machine. ^{19}F NMR spectra were obtained on a Bruker AV 400 spectrometer (Oxford magnet) at 376 MHz. ^{11}B NMR spectra were obtained on a Bruker AV 400 spectrometer (Oxford magnet) at 128 MHz. ^{1}H and ^{13}C, NMR spectra were obtained on either a Bruker AV 400 (Oxford magnet) at 400 MHz and 101 MHz, respectively, or Bruker Ascend AV(III) HD 500 at 500 MHz and 126 MHz, respectively. ^{11}B NMR was obtained in Norell® natural quartz 5 mm NMR tubes (500 MHz limit). Chemical shifts are reported in ppm and coupling constants are reported in Hz: $CDCl_3$ is referenced at 7.26 (^{1}H) and 77.0 (^{13}C), DMSO-d_6 referenced at 2.50 (^{1}H) and 39.5 (^{13}C). High-resolution mass spectra were obtained through analysis at the EPSRC UK National Mass Spectrometry Facility at Swansea University –or at the Mass Spectrometry Facility at Glasgow University. Reversed phase HPLC data was obtained on an Agilent 1200 series HPLC using a Machery-Nagel Nucleodur C18 column, which was maintained at a constant temperature of 40 °C. Analysis was performed using a gradient method, eluting with 5–80% MeCN/H_2O over 16 min at a flow rate of 2 mL/min. Samples for HPLC analysis were prepared through the addition of 2 mL of caffeine standard (to the completed reaction mixture, the resulting solution was then stirred before the removal of a 200 µL aliquot. The aliquot was diluted to 1 mL with MeCN, a 200 µL aliquot of the diluted solution was then filtered and further diluted with 800 µL MeCN and 500 µL H_2O for HPLC analysis against established conversion factors. Conversion factors were established as a 1:1 ratio caffeine/product. Reaction HPLC samples were run with a 1:4 ratio caffeine/product unless stated otherwise. cLogP values were obtained from JChem for office.

2. General Experimental Procedures

General Procedure A: Oxidation Study of Monoaryl Boron Systems (Scheme 2, Charts 1 and 2)

For example, oxidation of naphthalen-2-ylboronic acid, **1a**

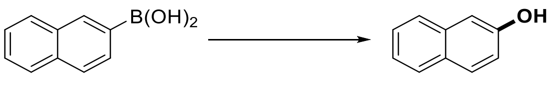

To an oven-dried microwave vial was added naphthalen-2-ylboronic acid (28 mg, 0.16 mmol, 1 equiv). THF (0.63 mL, 0.25 M) was added followed by a slurry of Oxone® (125 mg, 0.40 mmol, 2.5 equiv) in H_2O (1.28 mL). The reaction mixture was stirred at room temperature for 30 min. Sodium metabisulphite (122 mg, 0.64 mmol, 4 equiv) was added and conversion to product was determined by HPLC against an internal standard (caffeine) indicating oxidation of the naphthalen-2-ylboronic acid (98% yield).

General Procedure B: Optimized Reaction Boronic Acid *vs.* BPin or Boronic Acid (Table 1, entry 6)

For example, selective oxidation of naphthalen-2-ylboronic acid (**1a**) *vs.* [1,1'-biphenyl]-4-ylboronic acid, pinacol ester (**2b**)

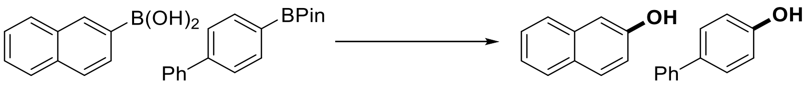

To an oven-dried 5 mL microwave vial was added naphthalen-2-ylboronic acid (28 mg, 0.16 mmol, 1 equiv), [1,1'-biphenyl]-4-ylboronic acid, pinacol ester (45 mg, 0.16 mmol, 1 equiv), and K_3PO_4 (103 mg, 0.48 mmol, 3 equiv). CPME (0.63 mL, 0.25 M) was added followed by a slurry of Oxone® (125 mg, 0.40 mmol, 2.5 equiv) in H_2O (1.28 mL) and CPME (0.25 mL). The reaction mixture was then heated to 70 °C with stirring in a sand bath for 1 h. The reaction was allowed to cool to room temperature before addition of sodium metabisulphite (122 mg, 0.64 mmol, 4 equiv). Conversion to products was determined by HPLC against an internal standard (caffeine) indicating selective oxidation of the naphthalen-2-ylboronic acid (quant., >99:1 selectivity).

General Procedure C: BMIDA Hydrolysis Optimization

For example, selective hydrolysis of naphthalen-2-ylboronic acid, MIDA ester (**1f**) *vs.* [1,1'-biphenyl]-4-ylboronic acid, pinacol ester (**2b**)

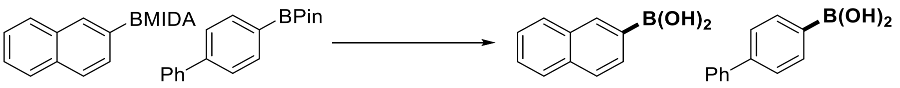

To an oven dried 5 mL microwave vial was added naphthalen-2-ylboronic acid, MIDA ester (45 mg, 0.16 mmol, 1 equiv), [1,1'-biphenyl]-4-ylboronic acid, pinacol ester (45 mg, 0.16 mmol, 1 equiv), and K_3PO_4 (103 mg, 0.48 mmol, 3 equiv). The vial was then capped and purged with N_2 before addition of CPME (0.63 mL, 0.25 M) and H_2O (14.5 µL, 0.80 mmol, 5 equiv). The reaction mixture was then heated to 80 °C in a sand bath with stirring for 15 min. Conversion to products was determined by HPLC against an internal standard (caffeine) indicating selective hydrolysis of naphthalen-2-ylboronic acid, MIDA ester (86% yield, 85:1 selectivity).

General Procedure D: Equilibration Reaction (Scheme 3)

For example, equilibration of naphthalen-2-ylboronic acid (**1a**) *vs.* [1,1'-biphenyl]-4-ylboronic acid, pinacol ester (**2b**)

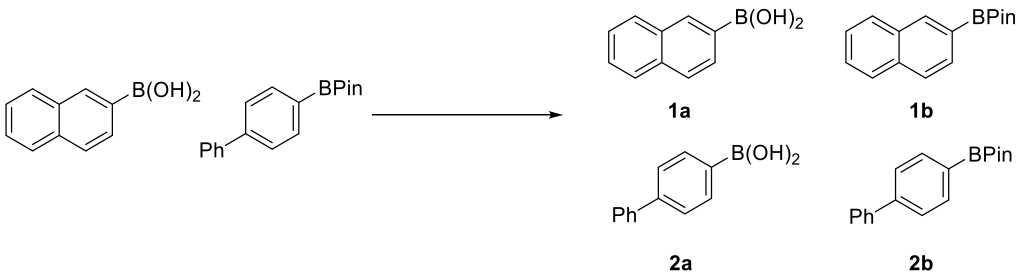

To an oven-dried 5 mL microwave vial was added [1,1'-biphenyl]-4-ylboronic acid, pinacol ester (45 mg, 0.16 mmol, 1 equiv), naphthalen-2-ylboronic acid (28 mg, 0.16 mmol, 1 equiv), and K_3PO_4 (103 mg, 0.48 mmol, 3 equiv). A mixture of THF and H_2O (10:1, 0.7 mL) was added and the reaction mixture was heated to 50 °C with stirring in a sand bath for 1 h. The reaction mixture was allowed to cool to room temperature and the conversion to products was determined by HPLC against an internal standard (caffeine) indicating a 55:46:45:54 mixture of products **1a**:**2b**:**1b**:**2a**.

General Procedure E: Origin of Chemoselectivity – HPLC Analysis (Table 2)

For example, HPLC analysis of biphasic system for naphthalen-2-ylboronic acid (**1a**) *vs.* [1,1'-biphenyl]-4-ylboronic acid, pinacol ester (**2b**)

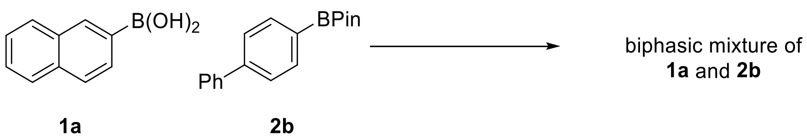

To an oven-dried 5 mL microwave vial was added naphthalen-2-ylboronic acid (28 mg, 0.16 mmol, 1 equiv), [1,1'-biphenyl]-4-ylboronic acid, pinacol ester (45 mg, 0.16 mmol, 1 equiv), K_3PO_4 (103 mg, 0.48 mmol, 3 equiv), $KHSO_4$ (27 mg, 0.2 mmol, 1.25 equiv), and K_2SO_4 (34 mg, 0.2 mmol, 1.25 equiv). A mixture of H_2O (1.28 mL) and CPME (0.88 mL) were added and the reaction mixture was heated to 70 °C with stirring in a sand bath for 10 min. The reaction mixture was removed from agitation and allowed to settle to form a biphase. A 200 µL aliquot was removed from each phase (aqueous and organic) and distribution of products was determined by HPLC against a known quantity of internal standard (caffeine) indicating selective phase transfer of naphthalen-2-ylboronic acid **1a**, 54:46 (organic/aqueous), **2b**, >99:1 (organic/aqueous).

General Procedure F: Boronate Formation of Boron Species (Scheme 4 and 5)

For example, synthesis of potassium trihydroxy(naphthalen-2-yl)borate, **1d**

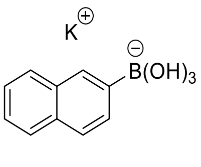

Naphthalen-2-ylboronic acid (6.1 mg, 0.036 mmol, 1 equiv) and K_3PO_4 (22.7 mg, 0.11 mmol, 3 equiv) were weighed out into a vial. D_2O (0.75 ml) was added and the mixture was sonicated until a solution was formed. The solution was transferred to a quartz NMR tube and a ^{11}B NMR was recorded at 343 K. Potassium trihydroxy(naphthalen-2-yl)borate provided a signal at 3.7 ppm.

General Procedure G: Origin of Chemoselectivity – NMR Analysis (Scheme 4, Scheme 5, and Figure 1)

For example, NMR analysis of biphasic system for naphthalen-2-ylboronic acid (**1a**) *vs.* [1,1'-biphenyl]-4-ylboronic acid, pinacol ester (**2b**)

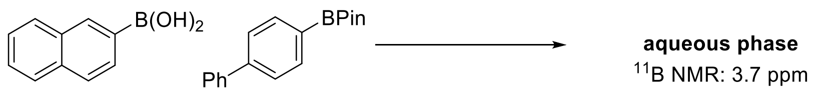

Naphthalen-2-ylboronic acid (17.2 mg, 0.1 mmol, 1 equiv) and [1,1'-biphenyl]-4-ylboronic acid, pinacol ester (28 mg, 0.1 mmol, 1 equiv) were dissolved in CPME (0.4 mL, 0.25 M) and transferred to a quartz NMR tube (Tube A). K_3PO_4 (63 mg, 0.3 mmol, 3 equiv), $KHSO_4$ (17 mg, 0.125 mmol, 1.25 equiv), and K_2SO_4 (21.5 mg, 0.125 mmol, 1.25 equiv) were weighed out into a vial (Vial A) and were dissolved in D_2O (0.8 mL) for later use. A D_2O blank (0.8 mL) NMR sample tube (Tube B) was prepared and used as a lock on the NMR machine. After locking (Tube B) was complete, Vial A containing inorganics was transferred slowly *via* syringe and long needle (needle must reach the bottom of the NMR tube) to Tube A to generate an aqueous biphasic system. The biphasic NMR sample (Tube A) was placed in the magnet and after shimming a data set was recorded every 5 min for 1 h at 293 K (128 scan per data set recording). After 1 h the temperature was increased to 323 K and a data set was recorded every 5 min for 1 h. After 1 h the temperature was further increased to 343 K and a data set was recorded every 5 min for 1 h. (No spinning was used in this NMR study)

General Procedure H: Optimized Reaction BMIDA *vs.* BPin (Scheme 9)

For example, selective oxidation of naphthalen-2-ylboronic acid, MIDA ester (**1f**) *vs.* [1,1'-biphenyl]-4-ylboronic acid, pinacol ester (**2b**)

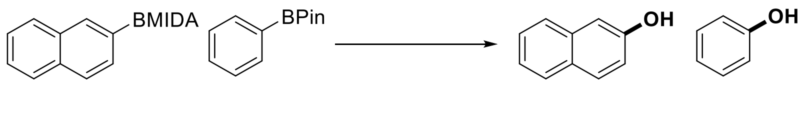

To an oven dried 5 mL microwave vial was added naphthalen-2-ylboronic acid, MIDA ester (45 mg, 0.16 mmol, 1 equiv), phenylboronic acid, pinacol ester (45 mg, 0.16 mmol, 1 equiv), and K_3PO_4 (103 mg, 0.48 mmol, 3 equiv). The vial was then capped and purged with N_2 before addition of CPME (0.63 mL, 0.25 M) and H_2O (14.5 µL, 0.80 mmol, 5 equiv). The reaction mixture was then heated to 80 °C in a sand bath with stirring for 10 min. The vial was then decapped and Oxone® (125 mg, 0.40

mmol, 2.5 equiv) was added as a slurry in H_2O (1.28 mL) and CPME (0.25 mL). The reaction was heated to 70 °C with stirring in a sand bath for 1 h. The reaction was allowed to cool to room temperature before addition of sodium metabisulphite (122 mg, 0.64 mmol, 4 equiv). Conversion to products was determined by HPLC against an internal standard (caffeine) indicating selective oxidation of the naphthalen-2-ylboronic acid, MIDA ester (56% yield, >99:1 selectivity).

General Procedure I: Synthesis of MIDA Esters from Boronic Acids

For example, for the preparation of (1*H*-indol-5-yl)boronic acid, MIDA ester, **S1**

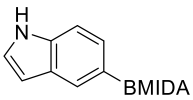

A mixture of (1*H*-indol-5-yl)boronic acid (2 g, 12.4 mmol, 1 equiv), *N*-methyliminodiacetic acid (1.9 g, 13.02 mmol, 1.05 equiv) in DMF (50 mL) was heated to 90 °C for 18 h. The reaction mixture was allowed to cool to room temperature and concentrated under vacuum to give an off-white slurry. EtOAc (100 mL) was added and the resulting precipitate was collected by filtration. The precipitate was washed with H_2O (2 × 50 mL) and Et_2O (2 × 50 mL) before being dried under vacuum to give the desired product as a white crystalline solid (3.3 g, 98%).

υ_{max} (solid): 3401, 3008, 2962, 1766, 1744, 1578, 1455, 1340, 1245, 1236 cm^{-1}.

^1H NMR ($CDCl_3$, 400 MHz): δ 11.02 (s, 1H), 7.62 (s, 1H), 7.37 (d, *J* = 8.2 Hz, 1H), 7.3 (t, *J* = 2.7 Hz, 1H), 7.14 (d, *J* = 8.2 Hz, 1H), 6.41 (s, 1H), 4.30 (d, *J* = 17.2 Hz, 2H), 4.08 (d, *J* = 17.2 Hz, 2H), 2.45 (s, 3H).

^{13}C NMR ($CDCl_3$, 101 MHz): δ 169.5, 136.5, 127.5, 124.9, 124.5, 110.8, 101.1, 61.6, 47.5. Carbon bearing boron not observed.

^{11}B NMR ($CDCl_3$, 128 MHz): δ 12.52.

HRMS: exact mass calculated for $[M+H]^+$ ($C_{13}H_{13}BN_2O_4$) requires *m/z* 273.1041, found *m/z* 273.1045.

General Procedure J: Synthesis of Diaryl Ethers *via* Oxidative Nucleophile Coupling

For example, for the preparation of 2-([1,1'-biphenyl]-4-yloxy)naphthalene, **32**

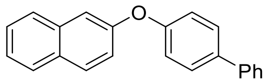

To an oven-dried 5 mL microwave vial was added naphthalen-2-ylboronic acid (28 mg, 0.16 mmol, 1 equiv), [1,1'-biphenyl]-4-ylboronic acid, pinacol ester (225 mg, 0.8 mmol, 5 equiv), and K_3PO_4 (103 mg, 0.48 mmol, 3 equiv). CPME (0.63 mL, 0.25 M) was added followed by a slurry of Oxone® (125 mg, 0.40 mmol, 2.5 equiv) in H_2O (1.28 mL) and CPME (0.25 mL). The reaction mixture was heated to 70 °C with stirring in a sand bath for 1 h. The reaction was allowed to cool to room temperature before addition of sodium metabisulphite (122 mg, 0.64 mmol, 4 equiv). EtOAc (20 mL) was added and organics washed with NH_4Cl (50 mL), H_2O (50 mL), and brine (50 mL). Solvent was removed under reduced pressure and the residue was transferred into an oven-dried 5 mL microwave vial. $Cu(OAc)_2$(58 mg, 0.32 mmol, 2 equiv), powdered activated molecular sieves, MeCN (350 µ*L*), EtOH (16 µL), and Et_3N (45 µL, 0.32 mmol, 2 equiv) were added and the vial was sealed under air. The reaction mixture was heated to 80 ˚C with stirring for 24 h. The reaction was allowed to cool to room temperature, EtOAc (20 mL) was added, and the mixture was passed through a layer of celite. The filtrate was washed with H_2O (50 mL) and brine (50 ml), dried through a hydrophobic frit, and solvent removed under reduced pressure. The crude product was purified by flash silica chromatography (0-10% EtOAc in petroleum ether) to yield the title compound as a white solid (39.1 mg, 82% yield).

3. Oxidation of Monoaryl Boron Systems – Kinetic Study

3.1. Oxidant Study

Reactions were carried out according to General Procedure A using either naphthalen-2-ylboronic acid **1a** (28 mg, 0.16 mmol, 1 equiv), or naphthalen-2-ylboronic acid, pinacol ester **1b** (40.6 mg, 0.16 mmol, 1 equiv), **oxidant** (0.4 mmol, 2.5 equiv), H_2O (1.28 mL), and THF (0.63 mL, 0.25 M). Reactions were run at room temperature for 30 min.

Entry	Boron Species	Oxidant	Conversion
**1**	**1a**	30% wt. aq. H_2O_2 (45.3 µL)	24%
**2**	**1b**	30% wt. aq. H_2O_2 (45.3 µL)	19%
**3**	**1a**	$NaBO_3 \bullet 4H_2O$ (62 mg)	quant.
**4**	**1b**	$NaBO_3 \bullet 4H_2O$ (62 mg)	quant.
**5**	**1a**	50% wt. *m*CPBA (138.4 mg)	92%

**6**	**1b**	50% wt. *m*CPBA (138.4 mg)	75%
**7**	**1a**	Oxone® (125 mg)	98%
**8**	**1b**	Oxone® (125 mg)	22%

3.2. Time Study (Chart 1)

Reactions were carried out according to General Procedure A using either naphthalen-2-ylboronic acid **1a** (28 mg, 0.16 mmol, 1 equiv), or [1,1'-biphenyl]-4-ylboronic acid, pinacol ester **1b** (45 mg, 0.16 mmol, 1 equiv), Oxone® (125 mg, 0.4 mmol, 2.5 equiv) as a slurry in H_2O (1.28 mL), and THF (0.63 mL, 0.25 M). Reactions were run at room temperature for **X** min.

Entry	Boron Species	Time (min)	Conversion
**1**	**1a**	0*	39%
**2**	**1a**	5	59%
**3**	**1a**	10	80%
**4**	**1a**	15	86%
**5**	**1a**	20	89%
**6**	**1a**	30	96%
**7**	**1b**	0*	4%
**8**	**1b**	5	9%
**9**	**1b**	10	12%
**10**	**1b**	15	17%
**11**	**1b**	20	20%
**12**	**1b**	30	23%

* - Reactions were quenched after 10 seconds.

4. Reaction Optimization Data

4.1. Boronic Acid Selective Oxidation

4.1.1. Base and Water Study

Reactions were carried out according to General Procedure B using naphthalen-2-ylboronic acid (28 mg, 0.16 mmol, 1 equiv), [1,1'-biphenyl]-4-ylboronic acid, pinacol ester (45 mg, 0.16 mmol, 1 equiv), **K_3PO_4** (103 mg, 0.48 mmol, 3 equiv), THF (0.63 mL, 0.25 M), and Oxone® (125 mg, 0.40 mmol, 2.5 equiv) as a slurry in **H_2O** (1.28 mL) and THF (0.25 mL). Reactions were run at room temperature for 1 h.

Entry	Base	Water	Conversion	1a:2b
**1**	-	-	0%	-
**2**	✓	-	0%	-
**3**	-	✓	95%	1.1:1
**4**	✓	✓	54%	14:1

4.1.2. Oxidant Study

Reactions were carried out according to General Procedure B using naphthalen-2-ylboronic acid (28 mg, 0.16 mmol, 1 equiv), [1,1'-biphenyl]-4-ylboronic acid, pinacol ester (45 mg, 0.16 mmol, 1 equiv), **Base** (0.48 mmol, 3 equiv), THF (0.63 mL, 0.25 M), and **Oxidant** (0.40 mmol, 2.5 equiv). Reactions were run at room temperature for 1 h.

Entry	Base	Oxidant	Conversion	1a:2b
**1**	-	30% wt. aq. H_2O_2 (45.3 µL)	90%	1:1
**2**	KOH (27 mg)	30% wt. aq. H_2O_2 (45.3 µL)	quant.	1:1
**3**	K_3PO_4 (103 mg)	30% wt. aq. H_2O_2 (45.3 µL)	quant.	1.1:1
**4**	K_3PO_4 (103 mg)	Oxone® (125 mg) in H_2O (1.28 mL) and THF (0.25 mL)	56%	5:1

4.1.3. Water Study

Reactions were carried out according to General Procedure B using naphthalen-2-ylboronic acid (28 mg, 0.16 mmol, 1 equiv), [1,1'-biphenyl]-4-ylboronic acid, pinacol ester (45 mg, 0.16 mmol, 1 equiv), K_3PO_4 (103 mg, 0.48 mmol, 3 equiv), THF (0.63 mL, 0.25 M), and Oxone® (125 mg, 0.40 mmol, 2.5 equiv) as a slurry in H_2O (**X** equiv), THF (0.25 mL), and acetone (0.25 mL). Reactions were run at 60 °C for 1 h.

Entry	Water (volume)	Conversion	1c:2c
**1**	100 equiv (0.29 mL)	72%	1:1
**2**	200 equiv (0.58 mL)	77%	1.1:1
**3**	300 equiv (0.87 mL)	quant.	1:1
**4**	400 equiv (1.16 mL)	quant.	1.1:1
**5**	450 equiv (1.28 mL)	91%	1.5:1
**6**	500 equiv (1.44 mL)	quant.	1:1
**7**	550 equiv (1.6 mL)	62%	3:1

4.1.4. Acetone Study

Reactions were carried out according to General Procedure B using naphthalen-2-ylboronic acid (28 mg, 0.16 mmol, 1 equiv), [1,1'-biphenyl]-4-ylboronic acid, pinacol ester (45 mg, 0.16 mmol, 1 equiv), K_3PO_4 (103 mg, 0.48 mmol, 3 equiv), THF (0.63 mL, 0.25 M), and Oxone® (125 mg, 0.40 mmol, 2.5 equiv) as a slurry in H_2O (1.28 mL), THF (0.25 mL), and acetone (**X** equiv). Reactions were run at 60 °C for 1 h.

Entry	Acetone (volume	Conversion	1c:2c
**1**	-	81%	18:1
**2**	5 equiv (60 µL)	54%	6:1
**3**	10 equiv (119 µL)	45%	3:1
**4**	20 equiv (239 µL)	98%	2:1

4.1.5. Time Study

Reactions were carried out according to General Procedure B using naphthalen-2-ylboronic acid (28 mg, 0.16 mmol, 1 equiv), [1,1'-biphenyl]-4-ylboronic acid, pinacol ester (45 mg, 0.16 mmol, 1 equiv), K_3PO_4 (103 mg, 0.48 mmol, 3 equiv), THF (0.63 mL, 0.25 M), and Oxone® (125 mg, 0.40 mmol, 2.5 equiv) as a slurry in H_2O (1.28 mL) and THF (0.25 mL). Reactions were run at 60 °C for **X min**.

Entry	Time (min)	Conversion	1c:2c
**1**	30	66%	25:1
**2**	45	78%	18:1
**3**	60	81%	18:1

4.1.6. Oxidant Equivalents Study

Reactions were carried out according to General Procedure B using naphthalen-2-ylboronic acid (28 mg, 0.16 mmol, 1 equiv), [1,1'-biphenyl]-4-ylboronic acid, pinacol ester (45 mg, 0.16 mmol, 1 equiv), K_3PO_4 (103 mg, 0.48 mmol, 3 equiv), THF (0.63 mL, 0.25 M), and Oxone® (**X** equiv) as a slurry in H_2O (1.28 mL) and THF (0.25 mL). Reactions were run at 60 °C for 1 h.

Entry	Oxone® (mass)	Conversion	1c:2c
**1**	2.5 equiv (125 mg)	81%	18:1
**2**	3.5 equiv (175 mg)	71%	16:1
**3**	4.5 equiv (225 mg)	61%	8:1

4.1.7. Base Study

Reactions were carried out according to General Procedure B using naphthalen-2-ylboronic acid (28 mg, 0.16 mmol, 1 equiv), [1,1'-biphenyl]-4-ylboronic acid, pinacol ester (45 mg, 0.16 mmol, 1 equiv), **Base** (0.48 mmol, 3 equiv), THF (0.63 mL, 0.25 M), and Oxone® (125 mg, 0.40 mmol, 2.5 equiv) as a slurry in H_2O (1.28 mL) and THF (0.25 mL). Reactions were run at 60 °C for 1 h.

Entry	Base (mass)	Conversion	1c:2c
**1**	K_3PO_4 (103 mg)	81%	18:1

**2**	Cs_2CO_3 (157 mg)	59%	14:1
**3**	K_2CO_3 (67 mg)	53%	12:1
**4**	KOAc (47 mg)	quant.	1:1
**5**	KOH (27 mg)	quant.	1.5:1

4.1.8. Base Equivalents Study

Reactions were carried out according to General Procedure B using naphthalen-2-ylboronic acid (28 mg, 0.16 mmol, 1 equiv), [1,1'-biphenyl]-4-ylboronic acid, pinacol ester (45 mg, 0.16 mmol, 1 equiv), K_3PO_4 (**X** equiv), THF (0.63 mL, 0.25 M), and Oxone® (125 mg, 0.40 mmol, 2.5 equiv) as a slurry in H_2O (1.28 mL) and THF (0.25 mL). Reactions were run at 60 °C for 1 h.

Entry	Base (mass)	Conversion	1c:2c
**1**	1 equiv (34 mg)	quant.	2:1
**2**	2 equiv (69 mg)	67%	4:1
**3**	3 equiv (103 mg)	81%	18:1

4.1.9. Temperature Study

Reactions were carried out according to General Procedure B using naphthalen-2-ylboronic acid (28 mg, 0.16 mmol, 1 equiv), [1,1'-biphenyl]-4-ylboronic acid, pinacol ester (45 mg, 0.16 mmol, 1 equiv), K_3PO_4 (103 mg, 0.48 mmol, 3 equiv), THF (0.63 mL, 0.25 M), and Oxone® (125 mg, 0.40 mmol, 2.5 equiv) as a slurry in H_2O (1.28 mL) and THF (0.25 mL). Reactions were run at **X** °C for 1 h.

Entry	Temperature	Conversion	1c:2c
**1**	rt	quant.	2:1
**2**	30 °C	quant.	5:1
**3**	40 °C	87%	7:1
**4**	50 °C	62%	9:1
**5**	60 °C	81%	18:1

**6**	70 °C	93%	13:1

4.1.10. Solvent Study

Reactions were carried out according to General Procedure B using naphthalen-2-ylboronic acid (28 mg, 0.16 mmol, 1 equiv), [1,1'-biphenyl]-4-ylboronic acid, pinacol ester (45 mg, 0.16 mmol, 1 equiv), K_3PO_4 (103 mg, 0.48 mmol, 3 equiv), **solvent** (0.63 mL, 0.25 M), and Oxone® (125 mg, 0.40 mmol, 2.5 equiv) as a slurry in H_2O (1.28 mL) and **solvent** (0.25 mL). Reactions were run at 70 °C for 1 h.

Entry	Solvent	Conversion	1c:2c
**1**	2-MeTHF	84%	47:1
**2**	CPME	quant.	>99:1
**3**	Toluene	59%	>99:1
**4**	1,4 Dioxane	quant.	2:1
**5**	EtOAc	quant.	63:1
**6**	IPA	quant.	1:1
**7**	EtOH	quant.	1:1
**8**	Chloroform	quant.	>99:1
**9**	DMF	quant.	1.5:1
**10**	MeCN	quant.	1:1
**11**	THF	73%	13:1

4.2. BMIDA Selective Oxidation

4.2.1 Hydrolysis Solvent Study

Reactions were carried out according to General Procedure C using naphthalen-2-ylboronic acid, MIDA ester (45 mg, 0.16 mmol, 1 equiv), [1,1'-biphenyl]-4-ylboronic acid, pinacol ester (45 mg, 0.16 mmol, 1 equiv), K_3PO_4 (103 mg, 0.48 mmol, 3 equiv), **solvent** (0.63 mL, 0.25 M), and H_2O (14.5 µL, 0.80 mmol, 5 equiv). Reactions were run at 90 °C for 30 min.

Entry	Solvent	Conversion to 1a	1a:2a
**1**	2-MeTHF	quant.	2:1
**2**	CPME	quant.	5:1
**3**	Toluene	83%	4:1
**4**	EtOAc	quant.	2:1

4.2.2. Hydrolysis Time Study

Reactions were carried out according to General Procedure C using naphthalen-2-ylboronic acid, MIDA ester (45 mg, 0.16 mmol, 1 equiv), [1,1'-biphenyl]-4-ylboronic acid, pinacol ester (45 mg, 0.16 mmol, 1 equiv), K_3PO_4 (103 mg, 0.48 mmol, 3 equiv), CPME (0.63 mL, 0.25 M), and H_2O (14.5 µL, 0.80 mmol, 5 equiv). Reactions were run at 90 °C for **X** min.

Entry	Time (min)	Conversion to 1a	1a:2a
**1**	15	87%	86:1
**2**	30	quant.	5:1
**3**	45	quant.	2:1
**4**	60	quant.	2:1
**5**	75	quant.	2:1
**6**	90	quant.	2:1

4.2.3. Hydrolysis Temperature Study

Reactions were carried out according to General Procedure C using naphthalen-2-ylboronic acid, MIDA ester (45 mg, 0.16 mmol, 1 equiv), [1,1'-biphenyl]-4-ylboronic acid, pinacol ester (45 mg, 0.16 mmol, 1 equiv), K_3PO_4 (103 mg, 0.48 mmol, 3 equiv), CPME (0.63 mL, 0.25 M), and H_2O (14.5 µL, 0.80 mmol, 5 equiv). Reactions were run at **X °C** for 30 min.

Entry	Temperature	Conversion to 1a	1a:2a
**1**	70 °C	10%	1:1

**2**	80 °C	quant.	20:1
**3**	90 °C	quant.	5:1
**4**	100 °C	quant.	2:1
**5**	110 °C	quant.	2:1

5. Determination of the Origin of Chemoselectivity

5.1. Boronic Acid and BPin Equilibration Investigation (Scheme 3)

Reactions were carried out according to General Procedure D using [1,1'-biphenyl]-4-ylboronic acid, pinacol ester (45 mg, 0.16 mmol, 1 equiv), naphthalen-2-ylboronic acid (28 mg, 0.16 mmol, 1 equiv), **Base** (0.48 mmol, 3 equiv), THF, and H_2O (10:1, 0.7 mL). Reactions were run at 50 °C for 1 h.

Entry	Base (mass)	1a (conv.)	2b (conv.)	1b (conv.)	2a (conv.)
**1**	-	96%	93%	4%	7%
**2**	K_3PO_4 (103 mg)	55%	46%	45%	54%
**3**	KOH (27 mg)	47%	47%	53%	53%

5.2. Shearing Effect Investigation (Chart 2)

Reactions were carried out according to General Procedure A using [1,1'-biphenyl]-4-ylboronic acid, pinacol ester (45 mg, 0.16 mmol, 1 equiv), Oxone® (125 mg, 0.4 mmol, 2.5 equiv) as a slurry in H_2O (1.28 mL), and THF (0.63 mL, 0.25 M). Reactions were run at room temperature with stirring at **X** rpm for **X** min.

Entry	Stir Rate (rpm)	Time (min)	Conversion
**1**	900	0*	4%
**2**	900	5	42%
**3**	900	10	54%
**4**	900	15	69%

**5**	900	20	75%
**6**	900	30	89%
**7**	350	0*	4%
**8**	350	5	9%
**9**	350	10	12%
**10**	350	15	17%
**11**	350	20	20%
**12**	350	30	23%

* Reactions were quenched after 10 seconds.

5.3. Determination of Phase Distribution – HPLC Analysis (Table 2)

Reactions were carried out according to General Procedure E using naphthalen-2-ylboronic acid (28 mg, 0.16 mmol, 1 equiv), [1,1'-biphenyl]-4-ylboronic acid, pinacol ester (45 mg, 0.16 mmol, 1 equiv) and a mixture of H_2O and CPME (1.28:0.88 mL). Reactions were run at **X** °C for 10 min using varying combinations of the following salts: $\mathbf{K_3PO_4}$ (103 mg, 0.48 mmol, 3 equiv), $\mathbf{KHSO_4}$ (27 mg, 0.2 mmol, 1.25 equiv), $\mathbf{K_2SO_4}$ (34 mg, 0.2 mmol, 1.25 equiv).

Entry	Inorganics Used	Temp (°C)	1a*	1b*
**1**	-	20	>99:1	>99:1
**2**	-	50	>99:1	>99:1
**3**	-	70	>99:1	>99:1
**4**	K_3PO_4	20	54:46	>99:1
**5**	K_3PO_4	50	46:54	75%
**6**	K_3PO_4	70	29:71	89%
**7**	$KHSO_4$, K_2SO_4	20	>99:1	>99:1
**8**	$KHSO_4$, K_2SO_4	50	>99:1	>99:1

**9**	$KHSO_4$, K_2SO_4	70	98:2	>99:1
**10**	K_3PO_4, $KHSO_4$, K_2SO_4	20	67:33	>99:1
**11**	K_3PO_4, $KHSO_4$, K_2SO_4	50	59:41	>99:1
**12**	K_3PO_4, $KHSO_4$, K_2SO_4	70	54:46	>99:1

*Ratios describe product distribution - organic:aqueous (%).

5.4. Determination of Phase Distribution – NMR Analysis

5.4.1. Setup

In order to assess only the aqueous phase during the process it was important to employ a reaction scale in which only the D_2O layer is detected by the receiver/transmitter coil. By ensuring that the D_2O layer and phase boundary of the biphasic system was > 0.5 mL, single analysis of the D_2O layer could be successfully achieved. The aqueous phase was analyzed at various temperatures over time to investigate mass transfer of specific boron species.

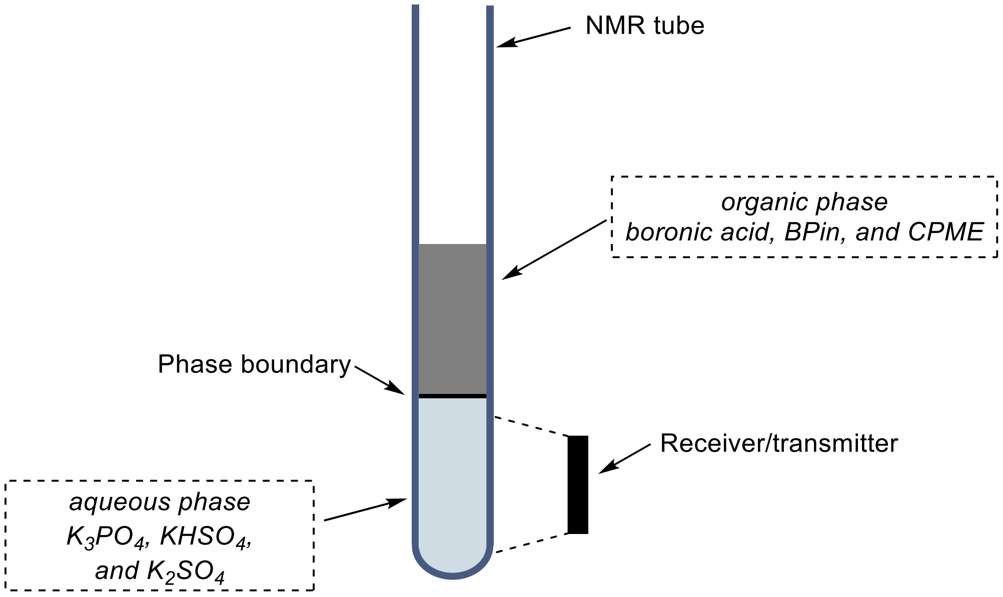

5.4.2. NMR Analysis of Monoaryl Boron Systems

NMR aqueous phase analysis of naphthalen-2-ylboronic acid, **1a**

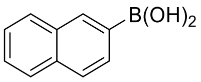

The NMR experiment was prepared according to General Procedure G using naphthalen-2-ylboronic acid (17.2 mg, 0.1 mmol, 1 equiv), CPME (0.4 mL, 0.25 M), K_3PO_4 (63 mg, 0.3 mmol, 3 equiv), $KHSO_4$ (17 mg, 0.125 mmol, 1.25 equiv), K_2SO_4 (21.5 mg, 0.125 mmol, 1.25 equiv), and D_2O (0.8 mL). An NMR was recorded (128 scans) at 293 K every 5 min for 1 h. This process was repeated with the same sample at both 323 K and 343 K.

NMR aqueous phase analysis of [1,1'-biphenyl]-4-ylboronic acid, pinacol ester, **2b**

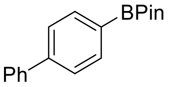

The NMR experiment was prepared according to General Procedure G using [1,1'-biphenyl]-4-ylboronic acid, pinacol ester (28 mg, 0.1 mmol, 1 equiv), CPME (0.4 mL, 0.25 M), K_3PO_4 (63 mg, 0.3 mmol, 3 equiv), $KHSO_4$ (17 mg, 0.125 mmol, 1.25 equiv), K_2SO_4 (21.5 mg, 0.125 mmol, 1.25 equiv), and D_2O (0.8 mL). A ^{11}B NMR was recorded (128 scans) according to the general procedure.

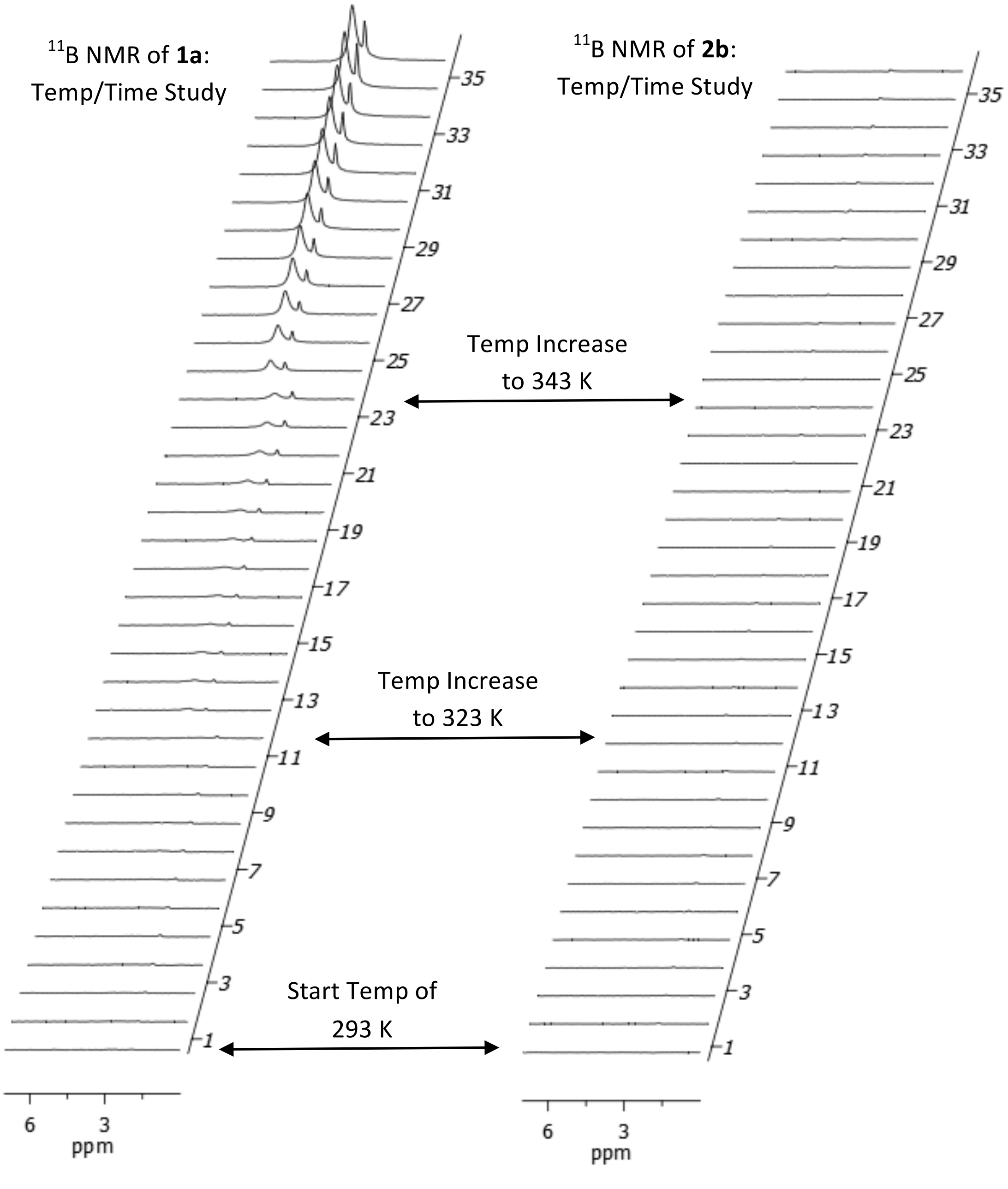

5.4.3. NMR Analysis of Diaryl Boron Systems

NMR aqueous phase analysis of naphthalen-2-ylboronic acid (**1a**) *vs.* [1,1'-biphenyl]-4-ylboronic acid, pinacol ester (**2b**)

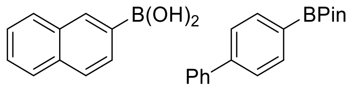

The NMR experiment was prepared according to General Procedure G using naphthalen-2-ylboronic acid (17.2 mg, 0.1 mmol, 1 equiv), [1,1'-biphenyl]-4-ylboronic acid, pinacol ester (28 mg, 0.1 mmol, 1 equiv), CPME (0.4 mL, 0.25 M), K_3PO_4 (63 mg, 0.3 mmol, 3 equiv), $KHSO_4$ (17 mg, 0.125 mmol, 1.25 equiv), K_2SO_4 (21.5 mg, 0.125 mmol, 1.25 equiv), and D_2O (0.8 mL). A ^{11}B NMR was recorded (128 scans) according to the general procedure.

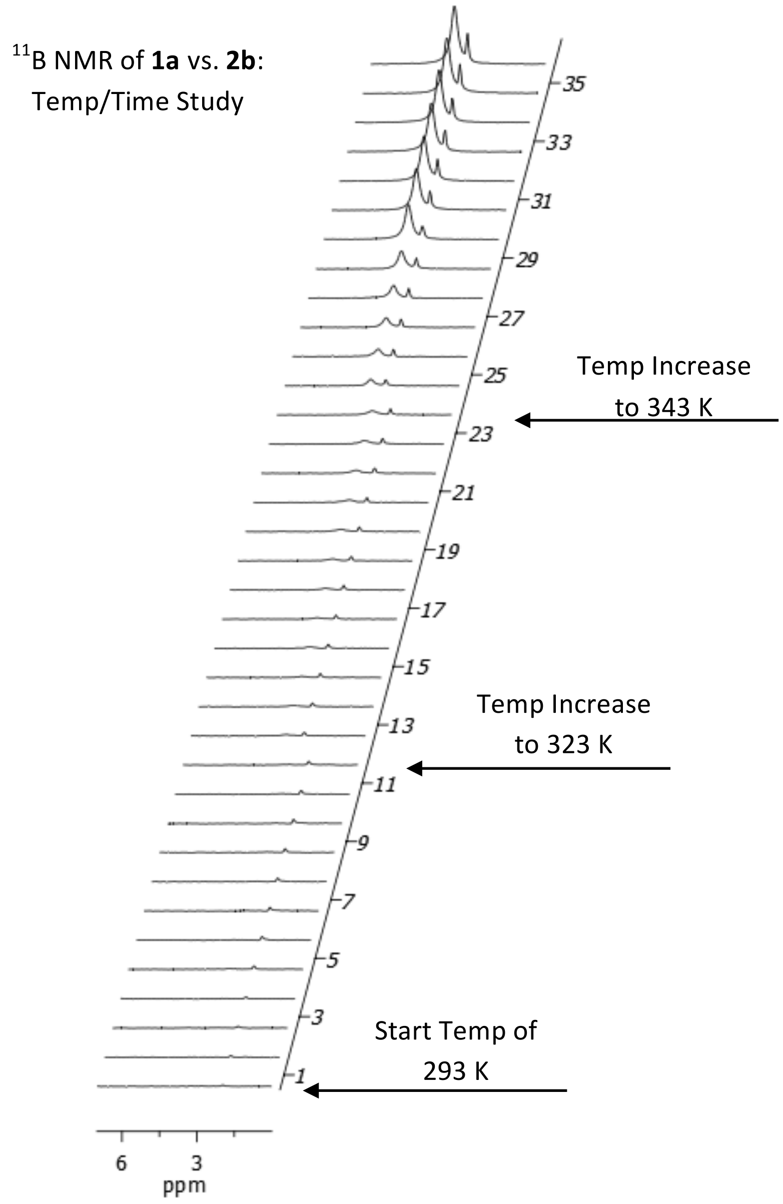

NMR aqueous phase analysis of naphthalen-2-ylboronic acid (**1a**) *vs.* (4-fluorophenyl)boronic acid, pinacol ester (**3b**)

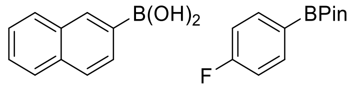

The NMR experiment was prepared according to General Procedure G using naphthalen-2-ylboronic acid (17.2 mg, 0.1 mmol, 1 equiv), (4-fluorophenyl)boronic acid, pinacol ester (22 mg, 0.1 mmol, 1 equiv), CPME (0.4 mL, 0.25 M), K_3PO_4 (63 mg, 0.3 mmol, 3 equiv), $KHSO_4$ (17 mg, 0.125 mmol, 1.25 equiv), K_2SO_4 (21.5 mg, 0.125 mmol, 1.25 equiv), and D_2O (0.8 mL). A ^{11}B NMR was recorded (128 scans) according to the general procedure. The overall process was repeated on a new sample for ^{19}F NMR (16 scans).

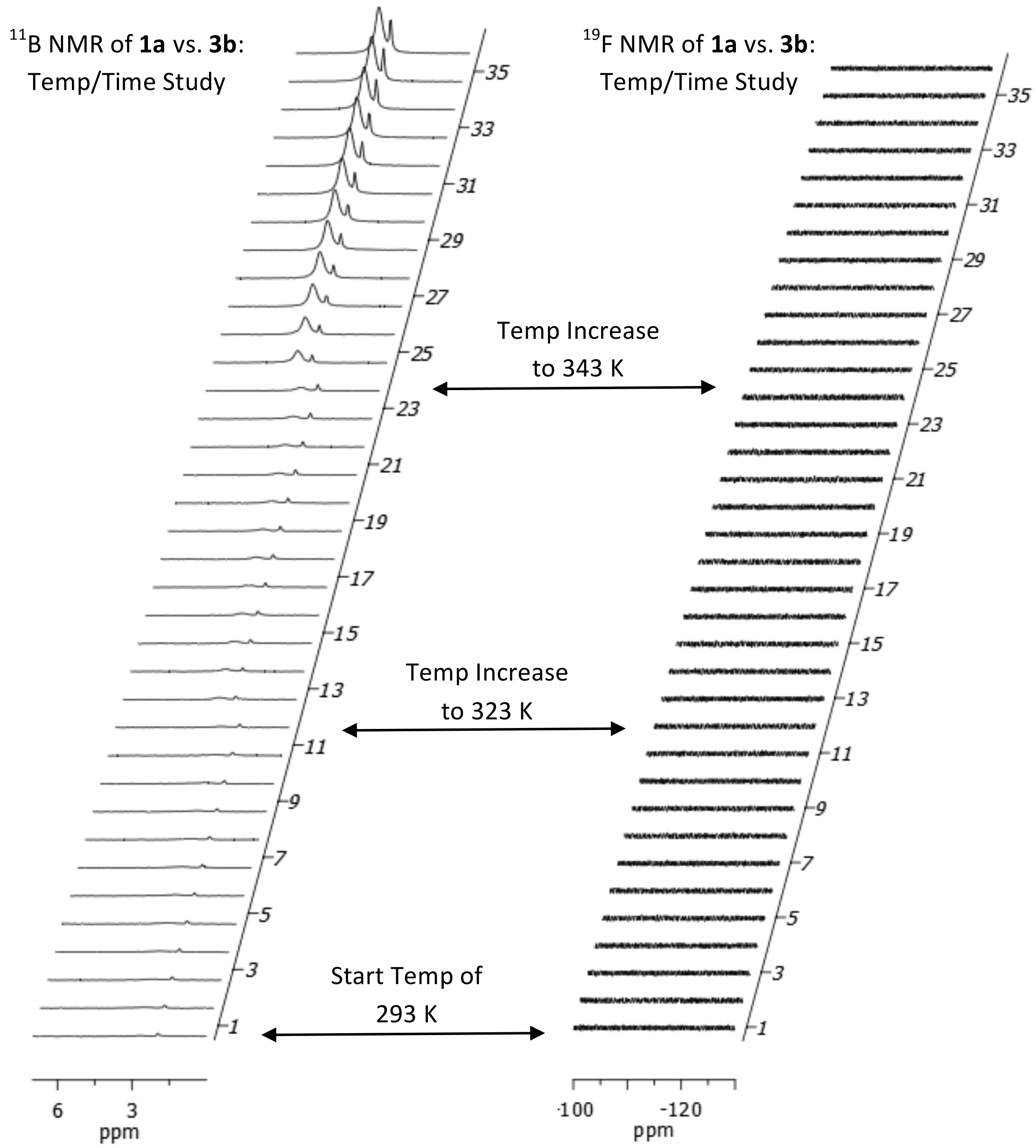

NMR aqueous phase analysis of (4-fluorophenyl)boronic acid (**3a**) *vs.* [1,1'-biphenyl]-4-ylboronic acid, pinacol ester (**2b**)

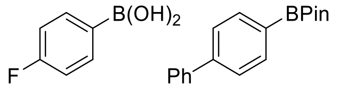

The NMR experiment was prepared according to General Procedure G using (4-fluorophenyl)boronic acid (14 mg, 0.1 mmol, 1 equiv), [1,1'-biphenyl]-4-ylboronic acid, pinacol ester (28 mg, 0.1 mmol, 1 equiv), CPME (0.4 mL, 0.25 M), K_3PO_4 (63 mg, 0.3 mmol, 3 equiv), $KHSO_4$ (17 mg, 0.125 mmol, 1.25 equiv), K_2SO_4 (21.5 mg, 0.125 mmol, 1.25 equiv), and D_2O (0.8 mL). A ^{11}B NMR was recorded (128 scans) according to the general procedure. The overall process was repeated on a new sample for ^{19}F NMR (16 scans).

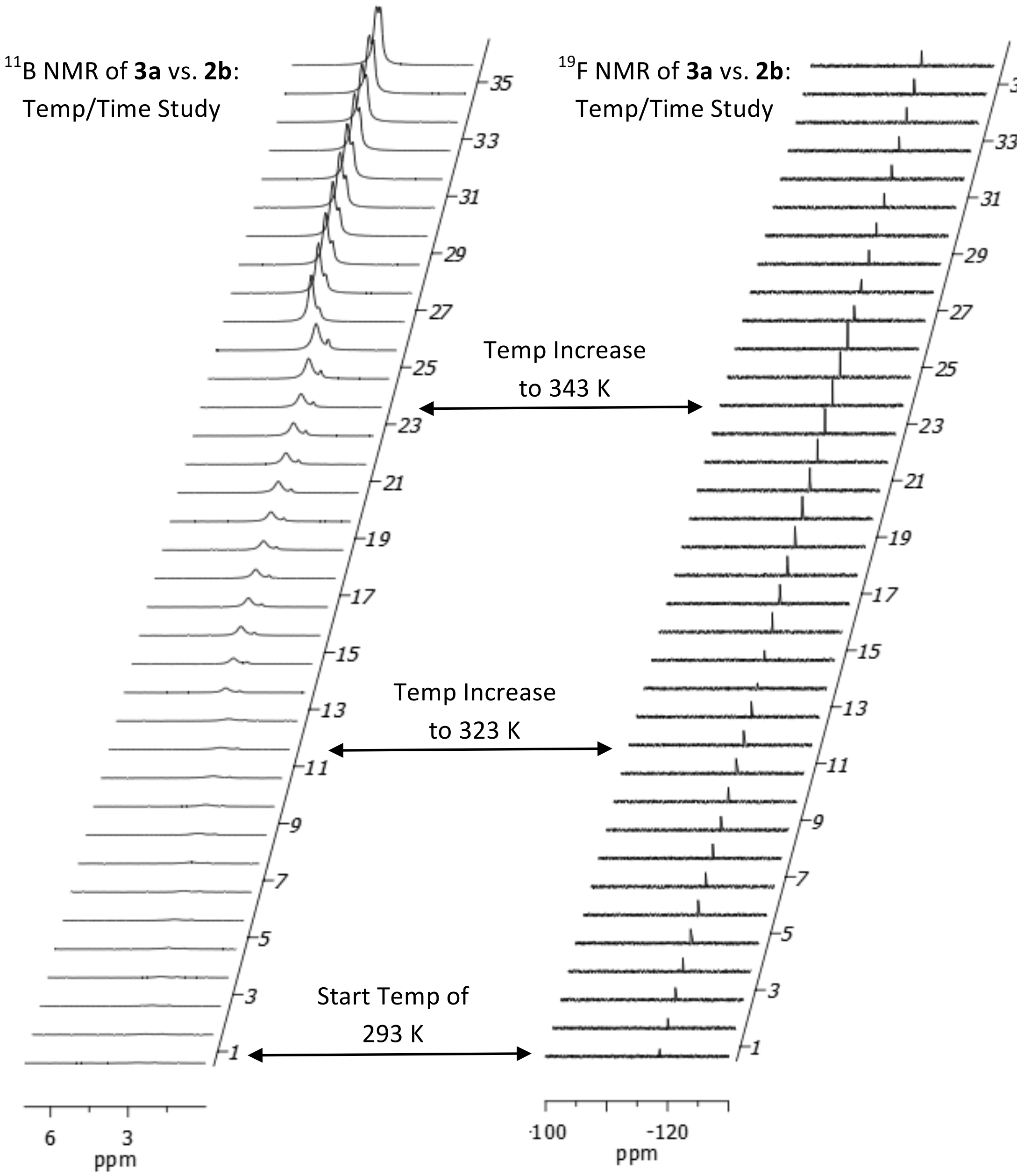

5.5. cLogP Parameters for Boron Species

Listed below are cLogP values for neutral boronic acids and boronic acid pinacol esters as well as their potassium boronate derivatives.[2]

Boron Species	cLogP Value
**1a**	2.64
**3a**	1.78
**4a**	1.64
**5a**	1.39
**6a**	0.97
**7a**	1.79
**8a**	1.74
**9a**	2.11
**10a**	1.59
**11a**	3.04
**12a**	2.43
**13a**	1.92
**14a**	0.77
**1d**	0.50
**3d**	- 0.37
**4d**	- 0.51
**5d**	- 0.76
**6d**	- 1.18
**7d**	- 0.36
**8d**	- 0.41

**9d**	- 0.04
**10d**	- 0.55
**11d**	0.90
**12d**	0.29
**13d**	- 0.23
**14d**	- 1.37
**2b**	5.58
**3b**	4.04
**4b**	3.90
**5b**	3.65
**6b**	3.23
**7b**	4.05
**8b**	4.00
**15b**	4.18
**16b**	3.72
**17b**	4.12
**18b**	3.49
**19b**	3.59
**20b**	3.12
**2e**	3.44
**3e**	1.89
**4e**	1.74
**5e**	1.50
**6e**	1.08

**7e**	1.90
**8e**	1.86
**15e**	2.03
**16e**	1.57
**17e**	1.98
**18e**	1.35
**19e**	1.44
**20e**	0.97

6. Chemoselective Oxidation - Boronic Acid vs. BPin (Table 1 and Scheme 7)

Naphthalen-2-ylboronic acid (**1a**) *vs.* [1,1'-biphenyl]-4-ylboronic acid, pinacol ester (**2b**), Table 1 entry 6

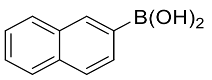

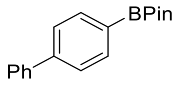

The reaction was carried out according to General Procedure B using naphthalen-2-ylboronic acid (28 mg, 0.16 mmol, 1 equiv), [1,1'-biphenyl]-4-ylboronic acid, pinacol ester (45 mg, 0.16 mmol, 1 equiv), K_3PO_4 (103 mg, 0.48 mmol, 3 equiv), CPME (0.63 mL, 0.25 M), Oxone® (125 mg, 0.40 mmol, 2.5 equiv) as a slurry in H_2O (1.28 mL) and CPME (0.25 mL). The reaction was run at 70 °C for 1 h. Conversion to products was analyzed by HPLC as outlined in the general procedure indicating selective oxidation of naphthalen-2-ylboronic acid (quant., >99:1).

(4-Fluorophenyl)boronic acid (**3a**) *vs.* [1,1'-biphenyl]-4-ylboronic acid, pinacol ester (**2b**)

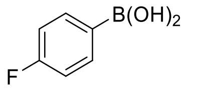

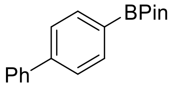

The reaction was carried out according to General Procedure B using (4-fluorophenyl)boronic acid (22 mg, 0.16 mmol, 1 equiv), [1,1'-biphenyl]-4-ylboronic acid, pinacol ester (45 mg, 0.16 mmol, 1 equiv), K_3PO_4 (103 mg, 0.48 mmol, 3 equiv), CPME (0.63 mL, 0.25 M), Oxone® (125 mg, 0.40

mmol, 2.5 equiv) as a slurry in H_2O (1.28 mL) and CPME (0.25 mL). The reaction was run at 70 °C for 1 h. Conversion to products was analyzed by HPLC as outlined in the general procedure indicating selective oxidation of (4-fluorophenyl)boronic acid (quant., >99:1).

Phenylboronic acid (**4a**) *vs.* [1,1'-biphenyl]-4-ylboronic acid, pinacol ester (**2b**)

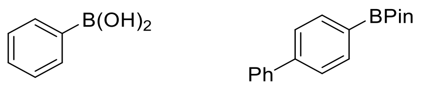

The reaction was carried out according to General Procedure B using phenylboronic acid (20 mg, 0.16 mmol, 1 equiv), [1,1'-biphenyl]-4-ylboronic acid, pinacol ester (45 mg, 0.16 mmol, 1 equiv), K_3PO_4 (103 mg, 0.48 mmol, 3 equiv), CPME (0.63 mL, 0.25 M), Oxone® (125 mg, 0.40 mmol, 2.5 equiv) as a slurry in H_2O (1.28 mL) and CPME (0.25 mL). The reaction was run at 70 °C for 1 h. Conversion to products was analyzed by HPLC as outlined in the general procedure indicating selective oxidation of phenylboronic acid (quant., >99:1).

(4-Methoxyphenyl)boronic acid (**5a**) *vs.* [1,1'-biphenyl]-4-ylboronic acid, pinacol ester (**2b**)

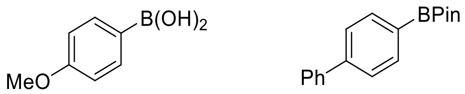

The reaction was carried out according to General Procedure B using (4-methoxyphenyl)boronic acid (24 mg, 0.16 mmol, 1 equiv), [1,1'-biphenyl]-4-ylboronic acid, pinacol ester (45 mg, 0.16 mmol, 1 equiv), K_3PO_4 (103 mg, 0.48 mmol, 3 equiv), CPME (0.63 mL, 0.25 M), Oxone® (125 mg, 0.40 mmol, 2.5 equiv) as a slurry in H_2O (1.28 mL) and CPME (0.25 mL). The reaction was run at 70 °C for 1 h. Conversion to products was analyzed by HPLC as outlined in the general procedure indicating selective oxidation of (4-methoxyphenyl)boronic acid (quant., >99:1).

(4-Acetamidophenyl)boronic acid (**6a**) *vs.* [1,1'-biphenyl]-4-ylboronic acid, pinacol ester (**2b**)

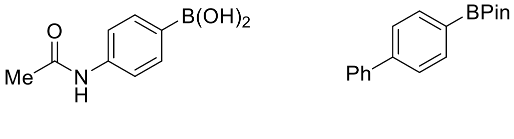

The reaction was carried out according to General Procedure B using (4-acetamidophenyl)boronic acid (29 mg, 0.16 mmol, 1 equiv), [1,1'-biphenyl]-4-ylboronic acid, pinacol ester (45 mg, 0.16 mmol, 1 equiv), K_3PO_4 (103 mg, 0.48 mmol, 3 equiv), CPME (0.63 mL, 0.25 M), Oxone® (125 mg, 0.40 mmol, 2.5 equiv) as a slurry in H_2O (1.6 mL) and CPME (0.25 mL). The reaction was run at 70 °C for

1 h. Conversion to products was analyzed by HPLC as outlined in the general procedure indicating selective oxidation of (4-acetamidophenyl)boronic acid (63%, 63:1).

(4-(Methoxycarbonyl)phenyl)boronic acid (**7a**) *vs.* [1,1'-biphenyl]-4-ylboronic acid, pinacol ester (**2b**)

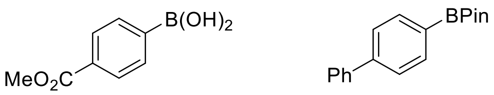

The reaction was carried out according to General Procedure B using (4-(methoxycarbonyl)phenyl)boronic acid (29 mg, 0.16 mmol, 1 equiv), [1,1'-biphenyl]-4-ylboronic acid, pinacol ester (45 mg, 0.16 mmol, 1 equiv), K_3PO_4 (103 mg, 0.48 mmol, 3 equiv), CPME (0.63 mL, 0.25 M), Oxone® (125 mg, 0.40 mmol, 2.5 equiv) as a slurry in H_2O (1.28 mL) and CPME (0.25 mL). The reaction was run at 70 °C for 1 h. Conversion to products was analyzed by HPLC as outlined in the general procedure indicating selective oxidation of (4-(methoxycarbonyl)phenyl)boronic acid (85%, >99:1).

(1*H*-Indol-5-yl)boronic acid (**8a**) *vs.* [1,1'-biphenyl]-4-ylboronic acid, pinacol ester (**2b**)

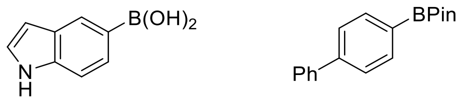

The reaction was carried out according to General Procedure B using (1*H*-indol-5-yl)boronic acid (26 mg, 0.16 mmol, 1 equiv), [1,1'-biphenyl]-4-ylboronic acid, pinacol ester (45 mg, 0.16 mmol, 1 equiv), K_3PO_4 (103 mg, 0.48 mmol, 3 equiv), CPME (0.63 mL, 0.25 M), Oxone® (125 mg, 0.40 mmol, 2.5 equiv) as a slurry in H_2O (1.6 mL) and CPME (0.25 mL). The reaction was run at 70 °C for 1 h. Conversion to products was analyzed by HPLC as outlined in the general procedure indicating selective oxidation of (1*H*-indol-5-yl)boronic acid (97%, >99:1).

4-Methylphenylboronic acid (**9a**) *vs.* [1,1'-biphenyl]-4-ylboronic acid, pinacol ester (**2b**)

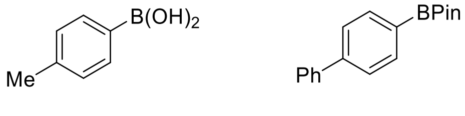

The reaction was carried out according to General Procedure B using 4-methylphenylboronic acid (22 mg, 0.16 mmol, 1 equiv), [1,1'-biphenyl]-4-ylboronic acid, pinacol ester (45 mg, 0.16 mmol, 1 equiv), K_3PO_4 (103 mg, 0.48 mmol, 3 equiv), CPME (0.63 mL, 0.25 M), Oxone® (125 mg, 0.40 mmol, 2.5 equiv) as a slurry in H_2O (1.28 mL) and CPME (0.25 mL). The reaction was run at 70 °C for 1 h.

Conversion to products was analyzed by HPLC as outlined in the general procedure indicating selective oxidation of 4-methylphenylboronic acid (quant., >99:1).

(2-Nitrophenyl)boronic acid (**10a**) *vs.* [1,1'-biphenyl]-4-ylboronic acid, pinacol ester (**2b**)

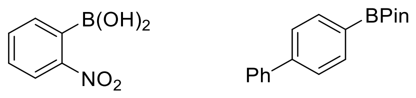

The reaction was carried out according to General Procedure B using (2-nitrophenyl)boronic acid (27 mg, 0.16 mmol, 1 equiv), [1,1'-biphenyl]-4-ylboronic acid, pinacol ester (45 mg, 0.16 mmol, 1 equiv), K_3PO_4 (103 mg, 0.48 mmol, 3 equiv), CPME (0.63 mL, 0.25 M), Oxone® (125 mg, 0.40 mmol, 2.5 equiv) as a slurry in H_2O (1.28 mL) and CPME (0.25 mL). The reaction was run at 70 °C for 1 h. Conversion to products was analyzed by HPLC as outlined in the general procedure indicating selective oxidation of (2-nitrophenyl)boronic acid (quant., >99:1).

Mesitylboronic acid (**11a**) *vs.* [1,1'-biphenyl]-4-ylboronic acid, pinacol ester (**2b**)

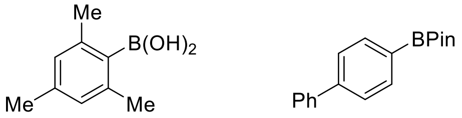

The reaction was carried out according to General Procedure B using mesitylboronic acid (26 mg, 0.16 mmol, 1 equiv), [1,1'-biphenyl]-4-ylboronic acid, pinacol ester (45 mg, 0.16 mmol, 1 equiv), K_3PO_4 (103 mg, 0.48 mmol, 3 equiv), CPME (0.63 mL, 0.25 M), Oxone® (125 mg, 0.40 mmol, 2.5 equiv) as a slurry in H_2O (1.6 mL) and CPME (0.25 mL). The reaction was run at 70 °C for 1 h. Conversion to products was analyzed by HPLC as outlined in the general procedure indicating selective oxidation of mesitylboronic acid (62%, 16:1).

(3-Bromophenyl)boronic acid (**12a**) *vs.* [1,1'-biphenyl]-4-ylboronic acid, pinacol ester (**2b**)

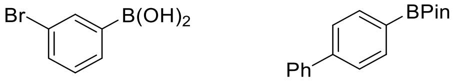

The reaction was carried out according to General Procedure B using (3-bromophenyl)boronic acid (32 mg, 0.16 mmol, 1 equiv), [1,1'-biphenyl]-4-ylboronic acid, pinacol ester (45 mg, 0.16 mmol, 1 equiv), K_3PO_4 (103 mg, 0.48 mmol, 3 equiv), CPME (0.63 mL, 0.25 M), Oxone® (125 mg, 0.40

mmol, 2.5 equiv) as a slurry in H_2O (1.28 mL) and CPME (0.25 mL). The reaction was run at 70 °C for 1 h. Conversion to products was analyzed by HPLC as outlined in the general procedure indicating selective oxidation of (3-bromophenyl)boronic acid (quant., >99:1).

Benzofuran-5-ylboronic acid (**13a**) *vs.* [1,1'-biphenyl]-4-ylboronic acid, pinacol ester (**2b**)

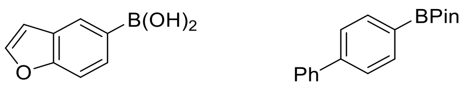

The reaction was carried out according to General Procedure B using benzofuran-5-ylboronic acid (26 mg, 0.16 mmol, 1 equiv), [1,1'-biphenyl]-4-ylboronic acid, pinacol ester (45 mg, 0.16 mmol, 1 equiv), K_3PO_4 (103 mg, 0.48 mmol, 3 equiv), CPME (0.63 mL, 0.25 M), Oxone® (125 mg, 0.40 mmol, 2.5 equiv) as a slurry in H_2O (1.28 mL) and CPME (0.25 mL). The reaction was run at 70 °C for 1 h. Conversion to products was analyzed by HPLC as outlined in the general procedure indicating selective oxidation of benzofuran-5-ylboronic acid (90%, >99:1).

(2-Methoxypyridin-3-yl)boronic acid (**14a**) *vs.* [1,1'-biphenyl]-4-ylboronic acid, pinacol ester (**2b**)

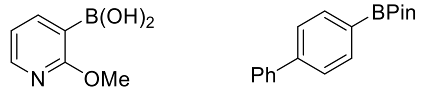

The reaction was carried out according to General Procedure B using (2-methoxypyridin-3-yl)boronic acid (24 mg, 0.16 mmol, 1 equiv), [1,1'-biphenyl]-4-ylboronic acid, pinacol ester (45 mg, 0.16 mmol, 1 equiv), K_3PO_4 (103 mg, 0.48 mmol, 3 equiv), CPME (0.63 mL, 0.25 M), Oxone® (125 mg, 0.40 mmol, 2.5 equiv) as a slurry in H_2O (1.28 mL) and CPME (0.25 mL). The reaction was run at 70 °C for 1 h. Conversion to products was analyzed by HPLC as outlined in the general procedure indicating selective oxidation of (2-methoxypyridin-3-yl)boronic acid (quant., >99:1).

Naphthalen-2-ylboronic acid (**1a**) *vs.* (4-fluorophenyl)boronic acid, pinacol ester (**3b)**

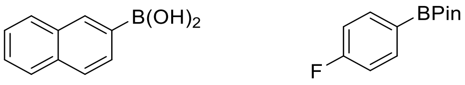

The reaction was carried out according to General Procedure B using naphthalen-2-ylboronic acid (28 mg, 0.16 mmol, 1 equiv), (4-fluorophenyl)boronic acid, pinacol ester (36 mg, 0.16 mmol, 1 equiv), K_3PO_4 (103 mg, 0.48 mmol, 3 equiv), CPME (0.63 mL, 0.25 M), Oxone® (125 mg, 0.40 mmol, 2.5

equiv) as a slurry in H_2O (1.6 mL) and CPME (0.25 mL). The reaction was run at 70 °C for 1 h. Conversion to products was analyzed by HPLC as outlined in the general procedure indicating selective oxidation of naphthalen-2-ylboronic acid (quant., >99:1).

Naphthalen-2-ylboronic acid (**1a**) *vs.* phenylboronic acid, pinacol ester (**4b**)

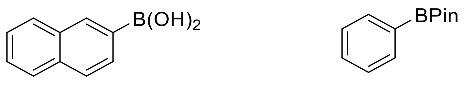

The reaction was carried out according to General Procedure B using naphthalen-2-ylboronic acid (28 mg, 0.16 mmol, 1 equiv), phenylboronic acid, pinacol ester (33 mg, 0.16 mmol, 1 equiv), K_3PO_4 (103 mg, 0.48 mmol, 3 equiv), CPME (0.63 mL, 0.25 M), Oxone® (125 mg, 0.40 mmol, 2.5 equiv) as a slurry in H_2O (1.28 mL) and CPME (0.25 mL). The reaction was run at 70 °C for 1 h. Conversion to products was analyzed by HPLC as outlined in the general procedure indicating selective oxidation of naphthalen-2-ylboronic acid (79%, >99:1).

Naphthalen-2-ylboronic acid (**1a**) *vs.* (4-methoxyphenyl)boronic acid, pinacol ester (**5b**)

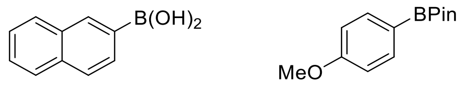

The reaction was carried out according to General Procedure B using naphthalen-2-ylboronic acid (28 mg, 0.16 mmol, 1 equiv), (4-methoxyphenyl)boronic acid, pinacol ester (37 mg, 0.16 mmol, 1 equiv), K_3PO_4 (103 mg, 0.48 mmol, 3 equiv), CPME (0.63 mL, 0.25 M), and Oxone® (125 mg, 0.40 mmol, 2.5 equiv) as a slurry in H_2O (1.28 mL) and CPME (0.25 mL). The reaction was run at 70 °C for 1h. Conversion to products was analyzed by HPLC as outlined in the general procedure indicating selective oxidation of naphthalen-2-ylboronic acid (90%, 85:1).

Naphthalen-2-ylboronic acid (**1a**) *vs.* (4-acetamidophenyl)boronic acid, pinacol ester (**6b**)

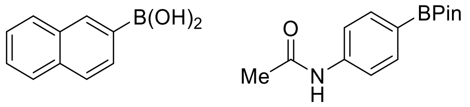

The reaction was carried out according to General Procedure B using naphthalen-2-ylboronic acid (28 mg, 0.16 mmol, 1 equiv), (4-acetamidophenyl)boronic acid, pinacol ester (42 mg, 0.16 mmol, 1 equiv), K_3PO_4 (103 mg, 0.48 mmol, 3 equiv), CPME (0.63 mL, 0.25 M), Oxone® (125 mg, 0.40

mmol, 2.5 equiv) as a slurry in H_2O (1.6 mL) and CPME (0.25 mL). The reaction was run at 70 °C for 1 h. Conversion to products was analyzed by HPLC as outlined in the general procedure indicating selective oxidation of naphthalen-2-ylboronic acid (82%, 14:1).

Naphthalen-2-ylboronic acid (**1a**) *vs.* (4-(methoxycarbonyl)phenyl)boronic acid, pinacol ester (**7b**)

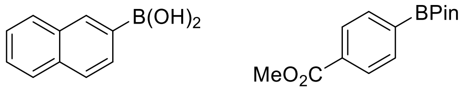

The reaction was carried out according to General Procedure B using naphthalen-2-ylboronic acid (28 mg, 0.16 mmol, 1 equiv), (4-(methoxycarbonyl)phenyl)boronic acid, pinacol ester (42 mg, 0.16 mmol, 1 equiv), K_3PO_4 (103 mg, 0.48 mmol, 3 equiv), CPME (0.63 mL, 0.25 M), Oxone® (125 mg, 0.40 mmol, 2.5 equiv) as a slurry in H_2O (1.6 mL) and CPME (0.25 mL). The reaction was run at 70 °C for 1 h. Conversion to products was analyzed by HPLC as outlined in the general procedure indicating selective oxidation of naphthalen-2-ylboronic acid (82%, 20:1).

Naphthalen-2-ylboronic acid (**1a**) *vs.* (1*H*-indol-5-yl)boronic acid, pinacol ester (**8b**)

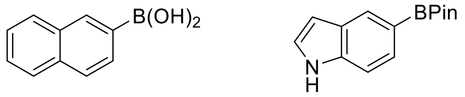

The reaction was carried out according to General Procedure B using naphthalen-2-ylboronic acid (28 mg, 0.16 mmol, 1 equiv), (1*H*-indol-5-yl)boronic acid, pinacol ester (39 mg, 0.16 mmol, 1 equiv), K_3PO_4 (103 mg, 0.48 mmol, 3 equiv), CPME (0.63 mL, 0.25 M), Oxone® (125 mg, 0.40 mmol, 2.5 equiv) as a slurry in H_2O (1.28 mL) and CPME (0.25 mL). The reaction was run at 70 °C for 1 h. Conversion to products was analyzed by HPLC as outlined in the general procedure indicating selective oxidation of naphthalen-2-ylboronic acid (quant., 99:1).

Naphthalen-2-ylboronic acid (**1a**) *vs.* (2,4-difluorophenyl)boronic acid, pinacol ester (**15b**)

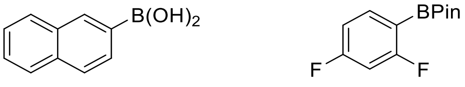

The reaction was carried out according to General Procedure B using naphthalen-2-ylboronic acid (28 mg, 0.16 mmol, 1 equiv), (2,4-difluorophenyl)boronic acid, pinacol ester (38 mg, 0.16 mmol, 1 equiv), K_3PO_4 (103 mg, 0.48 mmol, 3 equiv), CPME (0.63 mL, 0.25 M), Oxone® (125 mg, 0.40

mmol, 2.5 equiv) as a slurry in H_2O (1.28 mL) and CPME (0.25 mL). The reaction was run at 70 °C for 1 h. Conversion to products was analyzed by HPLC as outlined in the general procedure indicating selective oxidation of naphthalen-2-ylboronic acid (93%, 12:1).

Naphthalen-2-ylboronic acid (**1a**) *vs.* (4-cyanophenyl)boronic acid, pinacol ester (**16b**)

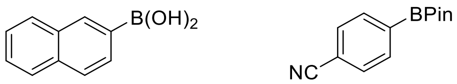

The reaction was carried out according to General Procedure B using naphthalen-2-ylboronic acid (28 mg, 0.16 mmol, 1 equiv), (4-cyanophenyl)boronic acid, pinacol ester (37 mg, 0.16 mmol, 1 equiv), K_3PO_4 (103 mg, 0.48 mmol, 3 equiv), CPME (0.63 mL, 0.25 M), Oxone® (125 mg, 0.40 mmol, 2.5 equiv) as a slurry in H_2O (1.28 mL) and CPME (0.25 mL). The reaction was run at 70 °C for 1 h. Conversion to products was analyzed by HPLC as outlined in the general procedure indicating selective oxidation of naphthalen-2-ylboronic acid (quant., 14:1).

Naphthalen-2-ylboronic acid (**1a**) *vs.* (benzofuran-2-yl)boronic acid, pinacol ester (**17b**)

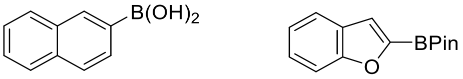

The reaction was carried out according to General Procedure B using naphthalen-2-ylboronic acid (28 mg, 0.16 mmol, 1 equiv), (benzofuran-2-yl)boronic acid, pinacol ester (42 mg, 0.16 mmol, 1 equiv), K_3PO_4 (103 mg, 0.48 mmol, 3 equiv), CPME (0.63 mL, 0.25 M), Oxone® (125 mg, 0.40 mmol, 2.5 equiv) as a slurry in H_2O (1.28 mL) and CPME (0.25 mL). The reaction was run at 70 °C for 1 h. Conversion to products was analyzed by HPLC as outlined in the general procedure indicating selective oxidation of naphthalen-2-ylboronic acid (57%, 55:1).

Naphthalen-2-ylboronic acid (**1a**) *vs.* thiophen-2-ylboronic acid, pinacol ester (**18b**)

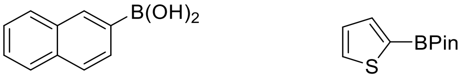

The reaction was carried out according to General Procedure B using naphthalen-2-ylboronic acid (28 mg, 0.16 mmol, 1 equiv), thiophen-2-ylboronic acid, pinacol ester (34 mg, 0.16 mmol, 1 equiv), K_3PO_4 (103 mg, 0.48 mmol, 3 equiv), CPME (0.63 mL, 0.25 M), Oxone® (125 mg, 0.40 mmol, 2.5

equiv) as a slurry in H_2O (1.28 mL) and CPME (0.25 mL). The reaction was run at 70 °C for 1 h. Conversion to products was analyzed by HPLC as outlined in the general procedure indicating selective oxidation of naphthalen-2-ylboronic acid (67%, >99:1).

Naphthalen-2-ylboronic acid (**1a**) *vs.* isoquinolin-4-ylboronic acid, pinacol ester (**19b**)

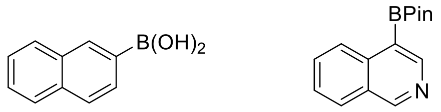

The reaction was carried out according to General Procedure B using naphthalen-2-ylboronic acid (28 mg, 0.16 mmol, 1 equiv), isoquinolin-4-ylboronic acid, pinacol ester (41 mg, 0.16 mmol, 1 equiv), K_3PO_4 (103 mg, 0.48 mmol, 3 equiv), CPME (0.63 mL, 0.25 M), Oxone® (125 mg, 0.40 mmol, 2.5 equiv) as a slurry in H_2O (1.28 mL) and CPME (0.25 mL). The reaction was run at 70 °C for 1 h. Conversion to products was analyzed by HPLC as outlined in the general procedure indicating selective oxidation of naphthalen-2-ylboronic acid (quant., 50:1).

Naphthalen-2-ylboronic acid (**1a**) *vs.* (2-aminophenyl)boronic acid, pinacol ester (**20b**)

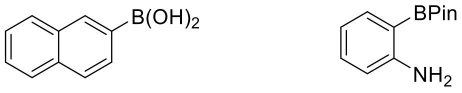

The reaction was carried out according to General Procedure B using naphthalen-2-ylboronic acid (28 mg, 0.16 mmol, 1 equiv), (2-aminophenyl)boronic acid, pinacol ester (35 mg, 0.16 mmol, 1 equiv), K_3PO_4 (103 mg, 0.48 mmol, 3 equiv), CPME (0.63 mL, 0.25 M), Oxone® (125 mg, 0.40 mmol, 2.5 equiv) as a slurry in H_2O (1.28 mL) and CPME (0.25 mL). The reaction was run at 70 °C for 1 h. Conversion to products was analyzed by HPLC as outlined in the general procedure indicating selective oxidation of naphthalen-2-ylboronic acid (75%, >99:1).

7. Chemoselective Oxidation - BMIDA vs. BPin (Scheme 9)

Naphthalen-2-ylboronic acid, MIDA ester (**1f**) *vs.* phenylboronic acid, pinacol ester (**4b**)

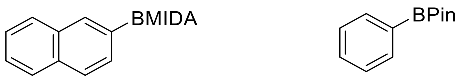

The reaction was carried out according to General Procedure H using naphthalen-2-ylboronic acid, MIDA ester (45 mg, 0.16 mmol, 1 equiv), phenylboronic acid, pinacol ester (33 mg, 0.16 mmol, 1 equiv), K_3PO_4 (103 mg, 0.48 mmol, 3 equiv), CPME (0.63 mL, 0.25 M), and H_2O (14.5 µL, 0.80 mmol, 5 equiv). The reaction was run at 80 °C for 15 min. Oxone® (125 mg, 0.40 mmol, 2.5 equiv) was added as a slurry in H_2O (1.6 mL) and CPME (0.25 mL) and the reaction was run at 70 °C for 1 h. Conversion to products was analyzed by HPLC as outlined in the general procedure indicating selective oxidation of naphthalen-2-ylboronic acid, MIDA ester (56%, >99:1).

Naphthalen-2-ylboronic acid, MIDA ester (**1f**) *vs.* (4-methoxyphenyl)boronic acid, pinacol ester (**5b**)

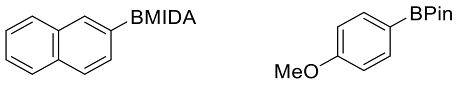

The reaction was carried out according to General Procedure H using naphthalen-2-ylboronic acid, MIDA ester (45 mg, 0.16 mmol, 1 equiv), (4-methoxyphenyl)boronic acid, pinacol ester (37 mg, 0.16 mmol, 1 equiv), K_3PO_4 (103 mg, 0.48 mmol, 3 equiv), CPME (0.63 mL, 0.25 M), and H_2O (14.5 µL, 0.80 mmol, 5 equiv). The reaction was run at 80 °C for 15 min. Oxone® (125 mg, 0.40 mmol, 2.5 equiv) was added as a slurry in H_2O (1.6 mL) and CPME (0.25 mL) and the reaction was run at 70 °C for 1 h. Conversion to products was analyzed by HPLC as outlined in the general procedure indicating selective oxidation of naphthalen-2-ylboronic acid, MIDA ester (82%, >99:1).

Naphthalen-2-ylboronic acid, MIDA ester (**1f**) *vs.* (4-(methoxycarbonyl)phenyl)boronic acid, pinacol ester (**7b**)

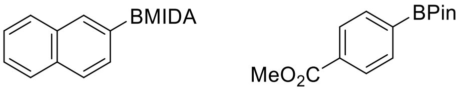

The reaction was carried out according to General Procedure H using naphthalen-2-ylboronic acid, MIDA ester (45 mg, 0.16 mmol, 1 equiv), (4-(methoxycarbonyl)phenyl)boronic acid, pinacol ester (42 mg, 0.16 mmol, 1 equiv), K_3PO_4 (103 mg, 0.48 mmol, 3 equiv), CPME (0.63 mL, 0.25 M), and H_2O (14.5 µL, 0.80 mmol, 5 equiv). The reaction was run at 80 °C for 15 min. Oxone® (125 mg, 0.40 mmol, 2.5 equiv) was added as a slurry in H_2O (1.6 mL) and CPME (0.25 mL) and the reaction was run at 70 °C for 1 h. Conversion to products was analyzed by HPLC as outlined in the general procedure indicating selective oxidation of naphthalen-2-ylboronic acid, MIDA ester (85%, 4:1).

Naphthalen-2-ylboronic acid, MIDA ester (**1f**) *vs.* thiophen-2-ylboronic acid, pinacol ester (**18b**)

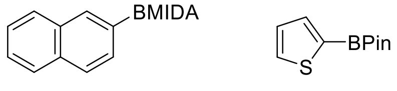

The reaction was carried out according to General Procedure H using naphthalen-2-ylboronic acid, MIDA ester (45 mg, 0.16 mmol, 1 equiv), thiophen-2-ylboronic acid (34 mg, 0.16 mmol, 1 equiv), K_3PO_4 (103 mg, 0.48 mmol, 3 equiv), CPME (0.63 mL, 0.25 M), and H_2O (14.5 μL, 0.80 mmol, 5 equiv). The reaction was run at 80 °C for 15 min. Oxone® (125 mg, 0.40 mmol, 2.5 equiv) was added as a slurry in H_2O (1.6 mL) and CPME (0.25 mL) and the reaction was run at 70 °C for 1 h. Conversion to products was analyzed by HPLC as outlined in the general procedure indicating selective oxidation of naphthalen-2-ylboronic acid, MIDA ester (83%, 6:1).

Naphthalen-2-ylboronic acid, MIDA ester (**1f**) *vs.* (4-isopropylphenyl)boronic acid, pinacol ester (**21b**)

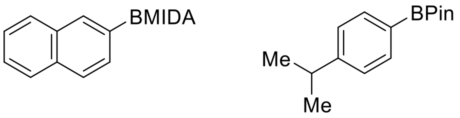

The reaction was carried out according to General Procedure H using naphthalen-2-ylboronic acid, MIDA ester (45 mg, 0.16 mmol, 1 equiv), (4-isopropylphenyl)boronic acid (39 mg, 0.16 mmol, 1 equiv), K_3PO_4 (103 mg, 0.48 mmol, 3 equiv), CPME (0.63 mL, 0.25 M), and H_2O (14.5 μL, 0.80 mmol, 5 equiv). The reaction was run at 80 °C for 15 min. Oxone® (125 mg, 0.40 mmol, 2.5 equiv) was added as a slurry in H_2O (1.6 mL) and CPME (0.25 mL) and the reaction was run at 70 °C for 1 h. Conversion to products was analyzed by HPLC as outlined in the general procedure indicating selective oxidation of naphthalen-2-ylboronic acid, MIDA ester (63%, >99:1).

Naphthalen-2-ylboronic acid, MIDA ester (**1f**) *vs.* (4-hydroxyphenyl)boronic acid, pinacol ester (**22b**)

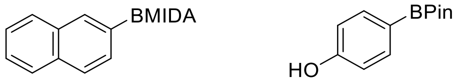

The reaction was carried out according to General Procedure H using naphthalen-2-ylboronic acid, MIDA ester (45 mg, 0.16 mmol, 1 equiv), (4-hydroxyphenyl)boronic acid, pinacol ester (35 mg, 0.16 mmol, 1 equiv), K_3PO_4 (103 mg, 0.48 mmol, 3 equiv), CPME (0.63 mL, 0.25 M), and H_2O (14.5 μL, 0.80 mmol, 5 equiv). The reaction was run at 80 °C for 15 min. Oxone® (125 mg, 0.40 mmol, 2.5 equiv) was added as a slurry in H_2O (1.6 mL) and CPME (0.25 mL) and the reaction was run at 70 °C

for 1 h. Conversion to products was analyzed by HPLC as outlined in the general procedure indicating selective oxidation of naphthalen-2-ylboronic acid, MIDA ester (67%, >99:1).

Naphthalen-2-ylboronic acid, MIDA ester (**1f**) *vs.* (2-chlorophenyl)boronic acid, pinacol ester (**23b**)

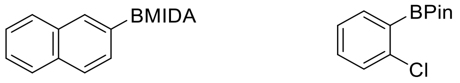

The reaction was carried out according to General Procedure H using naphthalen-2-ylboronic acid, MIDA ester (45 mg, 0.16 mmol, 1 equiv), (2-chlorophenyl)boronic acid, pinacol ester (38 mg, 0.16 mmol, 1 equiv), K_3PO_4 (103 mg, 0.48 mmol, 3 equiv), CPME (0.63 mL, 0.25 M), and H_2O (14.5 μL, 0.80 mmol, 5 equiv). The reaction was run at 80 °C for 15 min. Oxone® (125 mg, 0.40 mmol, 2.5 equiv) was added as a slurry in H_2O (1.6 mL) and CPME (0.25 mL) and the reaction was run at 70 °C for 1 h. Conversion to products was analyzed by HPLC as outlined in the general procedure indicating selective oxidation of naphthalen-2-ylboronic acid, MIDA ester (87%, >99:1).

Naphthalen-2-ylboronic acid, MIDA ester (**1f**) *vs.* (6-methoxypyridin-3-yl)boronic acid, pinacol ester (**24b**)

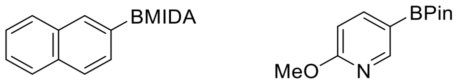

The reaction was carried out according to General Procedure H using naphthalen-2-ylboronic acid, MIDA ester (45 mg, 0.16 mmol, 1 equiv), (6-methoxypyridin-3-yl)boronic acid, pinacol ester (38 mg, 0.16 mmol, 1 equiv), K_3PO_4 (103 mg, 0.48 mmol, 3 equiv), CPME (0.63 mL, 0.25 M), and H_2O (14.5 μL, 0.80 mmol, 5 equiv). The reaction was run at 80 °C for 15 min. Oxone® (125 mg, 0.40 mmol, 2.5 equiv) was added as a slurry in H_2O (1.6 mL) and CPME (0.25 mL) and the reaction was run at 70 °C for 1 h. Conversion to products was analyzed by HPLC as outlined in the general procedure indicating selective oxidation of naphthalen-2-ylboronic acid, MIDA ester (69%, 9:1).

(4-Fluorophenyl)boronic acid, MIDA ester (**3f**) *vs.* [1,1'-biphenyl]-4-ylboronic acid, pinacol ester (**2b**)

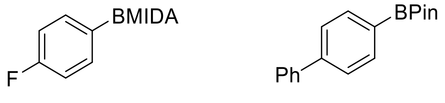

The reaction was carried out according to General Procedure H using (4-fluorophenyl)boronic acid, MIDA ester (40 mg, 0.16 mmol, 1 equiv), [1,1'-biphenyl]-4-ylboronic acid, pinacol ester (45 mg, 0.16 mmol, 1 equiv), K_3PO_4 (103 mg, 0.48 mmol, 3 equiv), CPME (0.63 mL, 0.25 M), and H_2O (14.5 µL, 0.80 mmol, 5 equiv). The reaction was run at 80 °C for 15 min. Oxone® (125 mg, 0.40 mmol, 2.5 equiv) was added as a slurry in H_2O (1.6 mL) and CPME (0.25 mL) and the reaction was run at 70 °C for 1 h. Conversion to products was analyzed by HPLC as outlined in the general procedure indicating selective oxidation of (4-fluorophenyl)boronic acid, MIDA ester (72%, >99:1).

Phenylboronic acid, MIDA ester (**4f**) *vs.* [1,1'-biphenyl]-4-ylboronic acid, pinacol ester (**2b**)

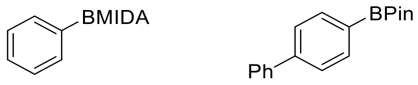

The reaction was carried out according to General Procedure H using phenylboronic acid, MIDA ester (37 mg, 0.16 mmol, 1 equiv), [1,1'-biphenyl]-4-ylboronic acid, pinacol ester (45 mg, 0.16 mmol, 1 equiv), K_3PO_4 (103 mg, 0.48 mmol, 3 equiv), CPME (0.63 mL, 0.25 M), and H_2O (14.5 µL, 0.80 mmol, 5 equiv). The reaction was run at 80 °C for 15 min. Oxone® (125 mg, 0.40 mmol, 2.5 equiv) was added as a slurry in H_2O (1.6 mL) and CPME (0.25 mL) and the reaction was run at 70 °C for 1 h. Conversion to products was analyzed by HPLC as outlined in the general procedure indicating selective oxidation of phenylboronic acid, MIDA ester (58%, 58:1).

(1*H*-Indol-5-yl)boronic acid, MIDA ester (**8f**) *vs.* [1,1'-biphenyl]-4-ylboronic acid, pinacol ester (**2b**)

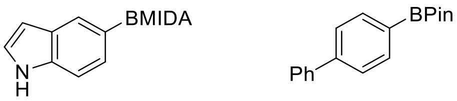

The reaction was carried out according to General Procedure H using (1*H*-indol-5-yl)boronic acid, MIDA ester (44 mg, 0.16 mmol, 1 equiv), [1,1'-biphenyl]-4-ylboronic acid, pinacol ester (45 mg, 0.16 mmol, 1 equiv), K_3PO_4 (103 mg, 0.48 mmol, 3 equiv), CPME (0.63 mL, 0.25 M), and H_2O (14.5 µL, 0.80 mmol, 5 equiv). The reaction was run at 80 °C for 15 min. Oxone® (125 mg, 0.40 mmol, 2.5 equiv) was added as a slurry in H_2O (1.6 mL) and CPME (0.25 mL) and the reaction was run at 70 °C for 1 h. Conversion to products was analyzed by HPLC as outlined in the general procedure indicating selective oxidation of (1*H*-indol-5-yl)boronic acid, MIDA ester (55%, >99:1).

4-Methylphenylboronic acid, MIDA ester (**9f**) *vs.* [1,1'-biphenyl]-4-ylboronic acid, pinacol ester (**2b**)

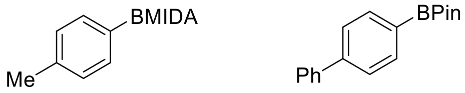

The reaction was carried out according to General Procedure H using 4-methylphenylboronic acid, MIDA ester (40 mg, 0.16 mmol, 1 equiv), [1,1'-biphenyl]-4-ylboronic acid, pinacol ester (45 mg, 0.16 mmol, 1 equiv), K_3PO_4 (103 mg, 0.48 mmol, 3 equiv), CPME (0.63 mL, 0.25 M), and H_2O (14.5 µL, 0.80 mmol, 5 equiv). The reaction was run at 80 °C for 15 min. Oxone® (125 mg, 0.40 mmol, 2.5 equiv) was added as a slurry in H_2O (1.6 mL) and CPME (0.25 mL) and the reaction was run at 70 °C for 1 h. Conversion to products was analyzed by HPLC as outlined in the general procedure indicating selective oxidation of 4-methylphenylboronic acid, MIDA ester (84%, 84:1).

Benzofuran-5-ylboronic acid, MIDA ester (**13f**) *vs.* [1,1'-biphenyl]-4-ylboronic acid, pinacol ester (**2b**)

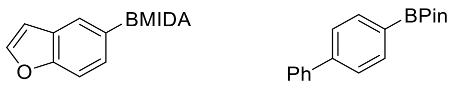

The reaction was carried out according to General Procedure H using benzofuran-5-ylboronic acid, MIDA ester (44 mg, 0.16 mmol, 1 equiv), [1,1'-biphenyl]-4-ylboronic acid, pinacol ester (45 mg, 0.16 mmol, 1 equiv), K_3PO_4 (103 mg, 0.48 mmol, 3 equiv), CPME (0.63 mL, 0.25 M), and H_2O (14.5 µL, 0.80 mmol, 5 equiv). The reaction was run at 80 °C for 15 min. Oxone® (125 mg, 0.40 mmol, 2.5 equiv) was added as a slurry in H_2O (1.6 mL) and CPME (0.25 mL) and the reaction was run at 70 °C for 1 h. Conversion to products was analyzed by HPLC as outlined in the general procedure indicating selective oxidation of benzofuran-5-ylboronic acid, MIDA ester (76%, 25:1).

(4-Hydroxyphenyl)boronic acid, MIDA ester (**22f**) *vs.* [1,1'-biphenyl]-4-ylboronic acid, pinacol ester (**2b**)

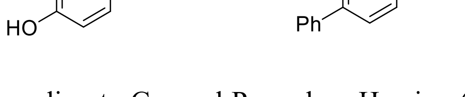

The reaction was carried out according to General Procedure H using (4-hydroxyphenyl)boronic acid, MIDA ester (40 mg, 0.16 mmol, 1 equiv), [1,1'-biphenyl]-4-ylboronic acid, pinacol ester (45 mg, 0.16 mmol, 1 equiv), K_3PO_4 (103 mg, 0.48 mmol, 3 equiv), CPME (0.63 mL, 0.25 M), and H_2O (14.5 µL, 0.80 mmol, 5 equiv). The reaction was run at 80 °C for 15 min. Oxone® (125 mg, 0.40 mmol, 2.5 equiv) was added as a slurry in H_2O (1.6 mL) and CPME (0.25 mL) and the reaction was run at 70 °C

for 1 h. Conversion to products was analyzed by HPLC as outlined in the general procedure indicating selective oxidation of (4-hydroxyphenyl)boronic acid, MIDA ester (50%, 50:1).

(2-Bromophenyl)boronic acid, MIDA ester (**25f**) *vs.* [1,1'-biphenyl]-4-ylboronic acid, pinacol ester (**2b**)

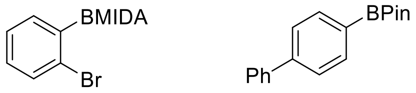

The reaction was carried out according to General Procedure H using (2-bromophenyl)boronic acid, MIDA ester (50 mg, 0.16 mmol, 1 equiv), [1,1'-biphenyl]-4-ylboronic acid, pinacol ester (45 mg, 0.16 mmol, 1 equiv), K_3PO_4 (103 mg, 0.48 mmol, 3 equiv), CPME (0.63 mL, 0.25 M), and H_2O (14.5 µL, 0.80 mmol, 5 equiv). The reaction was run at 80 °C for 15 min. Oxone® (125 mg, 0.40 mmol, 2.5 equiv) was added as a slurry in H_2O (1.6 mL) and CPME (0.25 mL) and the reaction was run at 70 °C for 1 h. Conversion to products was analyzed by HPLC as outlined in the general procedure indicating selective oxidation of (2-bromophenyl)boronic acid, MIDA ester (quant., >99:1).

(3-Isobutoxyphenyl)boronic acid, MIDA ester (**26f**) *vs.* [1,1'-biphenyl]-4-ylboronic acid, pinacol ester (**2b**)

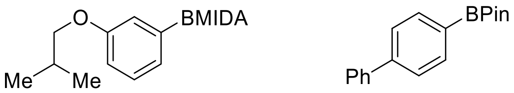

The reaction was carried out according to General Procedure H using (3-isobutoxyphenyl)boronic acid, MIDA ester (31 mg, 0.16 mmol, 1 equiv), [1,1'-biphenyl]-4-ylboronic acid, pinacol ester (45 mg, 0.16 mmol, 1 equiv), K_3PO_4 (103 mg, 0.48 mmol, 3 equiv), CPME (0.63 mL, 0.25 M), and H_2O (14.5 µL, 0.80 mmol, 5 equiv). The reaction was run at 80 °C for 15 min. Oxone® (125 mg, 0.40 mmol, 2.5 equiv) was added as a slurry in H_2O (1.6 mL) and CPME (0.25 mL) and the reaction was run at 70 °C for 1 h. Conversion to products was analyzed by HPLC as outlined in the general procedure indicating selective oxidation of (3-isobutoxyphenyl)boronic acid, MIDA ester (71%, 24:1).

8. Chemoselective Oxidation - Boronic Acid *vs.* Boronic Acid

8.1. Determination of Boronic Acid *vs.* Boronic Acid Phase Distribution – HPLC Analysis (Scheme 11)

Naphthalen-2-ylboronic acid (**1a**) *vs.* (4-methoxyphenyl)boronic acid (**5a**),

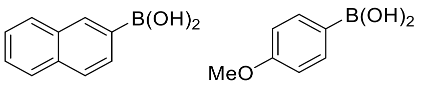

The reaction was carried out according to General Procedure E using naphthalen-2-ylboronic acid (28 mg, 0.16 mmol, 1 equiv), (4-methoxyphenyl)boronic acid (24 mg, 0.16 mmol, 1 equiv), K_3PO_4 (103 mg, 0.48 mmol, 3 equiv), and a mixture of H_2O and CPME (1.28:0.88 mL). The reaction was run at 70 °C for 10 min. Distribution of products was analyzed by HPLC as outlined in the general procedure indicating phase transfer of **5a**, 0:100 (organic/aqueous), and **1a**, 72:28 (organic/aqueous).

Naphthalen-2-ylboronic acid (**1a**) *vs.* phenylboronic acid (**4a**),

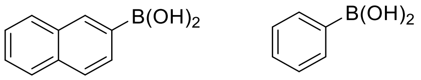

The reaction was carried out according to General Procedure E using naphthalen-2-ylboronic acid (28 mg, 0.16 mmol, 1 equiv), phenylboronic acid (20 mg, 0.16 mmol, 1 equiv), K_3PO_4 (103 mg, 0.48 mmol, 3 equiv), and a mixture of H_2O and CPME (1.28:0.88 mL). The reaction was run at 70 °C for 10 min. Distribution of products was analyzed by HPLC as outlined in the general procedure indicating phase transfer of **4a**, 5:95 (organic/aqueous), and **1a**, 72:28 (organic/aqueous).

Naphthalen-2-ylboronic acid (**1a**) *vs.* (4-fluorophenyl)boronic acid (**3a**),

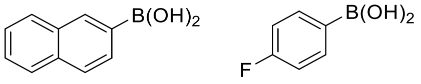

The reaction was carried out according to General Procedure E using naphthalen-2-ylboronic acid (28 mg, 0.16 mmol, 1 equiv), (4-fluorophenyl)boronic acid (22 mg, 0.16 mmol, 1 equiv), K_3PO_4 (103 mg, 0.48 mmol, 3 equiv), and a mixture of H_2O and CPME (1.28:0.88 mL). The reaction was run at 70 °C for 10 min. Distribution of products was analyzed by HPLC as outlined in the general procedure indicating phase transfer of **3a**, 20:80 (organic/aqueous), and **1a**, 63:37 (organic/aqueous).

Naphthalen-2-ylboronic acid (**1a**) *vs.* (4-(methoxycarbonyl)phenyl)boronic acid (**7a**),

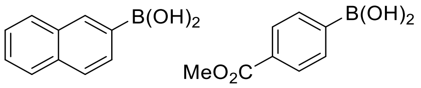

The reaction was carried out according to General Procedure E using naphthalen-2-ylboronic acid (28 mg, 0.16 mmol, 1 equiv), (4-(methoxycarbonyl)phenyl)boronic acid (28 mg, 0.16 mmol, 1 equiv), K_3PO_4 (103 mg, 0.48 mmol, 3 equiv), and a mixture of H_2O and CPME (1.28:0.88 mL). The reaction was run at 70 °C for 10 min. Distribution of products was analyzed by HPLC as outlined in the general procedure indicating phase transfer of **7a**, 9:91 (organic/aqueous), and **1a**, 85:15 (organic/aqueous).

Naphthalen-2-ylboronic acid (**1a**) *vs.* (4-nitrophenyl)boronic acid (**31a**),

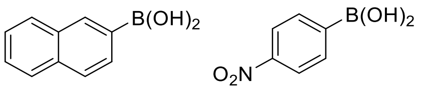

The reaction was carried out according to General Procedure E using naphthalen-2-ylboronic acid (28 mg, 0.16 mmol, 1 equiv), (4-nitrophenyl)boronic acid (27 mg, 0.16 mmol, 1 equiv), K_3PO_4 (103 mg, 0.48 mmol, 3 equiv), and a mixture of H_2O and CPME (1.28:0.88 mL). The reaction was run at 70 °C for 10 min. Distribution of products was analyzed by HPLC as outlined in the general procedure indicating phase transfer of **31a**, 11:14 (organic/aqueous), and **1a**, 64:36 (organic/aqueous).

8.2. Boronic Acid *vs.* Boronic Acid – Substrate Scope (Scheme 11 and Table 3)

Naphthalen-2-ylboronic acid (**1a**) *vs.* (4-methoxyphenyl)boronic acid (**5a**), Scheme 11

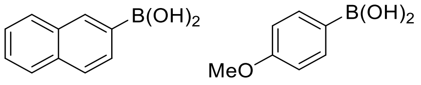

The reaction was carried out according to General Procedure B using naphthalen-2-ylboronic acid (28 mg, 0.16 mmol, 1 equiv), (4-methoxyphenyl)boronic acid (24 mg, 0.16 mmol, 1 equiv), K_3PO_4 (103 mg, 0.48 mmol, 3 equiv), CPME (0.63 mL, 0.25 M), Oxone® (125 mg, 0.40 mmol, 2.5 equiv) as a slurry in H_2O (1.6 mL) and CPME (0.25 mL). The reaction was run at 70 °C for 30 min. Conversion to products was analyzed by HPLC as outlined in the general procedure indicating selective oxidation of (4-methoxyphenyl)boronic acid (quant., 4:1).

Naphthalen-2-ylboronic acid (**1a**) *vs.* phenylboronic acid (**4a**), Scheme 11

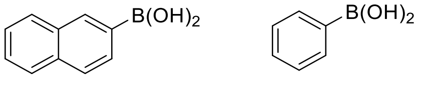

The reaction was carried out according to General Procedure B using naphthalen-2-ylboronic acid (28 mg, 0.16 mmol, 1 equiv), phenylboronic acid (20 mg, 0.16 mmol, 1 equiv), K_3PO_4 (103 mg, 0.48 mmol, 3 equiv), CPME (0.63 mL, 0.25 M), Oxone® (125 mg, 0.40 mmol, 2.5 equiv) as a slurry in H_2O (1.6 mL) and CPME (0.25 mL). The reaction was run at 70 °C for 30 min. Conversion to products was analyzed by HPLC as outlined in the general procedure indicating selective oxidation of phenylboronic acid (88%, 6.5:1).

Naphthalen-2-ylboronic acid (**1a**) *vs.* (4-fluorophenyl)boronic acid (**3a**), Scheme 11

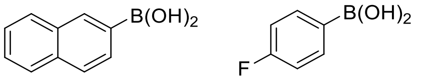

The reaction was carried out according to General Procedure B using naphthalen-2-ylboronic acid (28 mg, 0.16 mmol, 1 equiv), (4-fluorophenyl)boronic acid (22 mg, 0.16 mmol, 1 equiv), K_3PO_4 (103 mg, 0.48 mmol, 3 equiv), CPME (0.63 mL, 0.25 M), Oxone® (125 mg, 0.40 mmol, 2.5 equiv) as a slurry in H_2O (1.6 mL) and CPME (0.25 mL). The reaction was run at 70 °C for 30 min. Conversion to products was analyzed by HPLC as outlined in the general procedure indicating selective oxidation of (4-fluorophenyl)boronic acid (88%, 8:1).

Naphthalen-2-ylboronic acid (**1a**) *vs.* (4-(methoxycarbonyl)phenyl)boronic acid (**7a**), Scheme 11

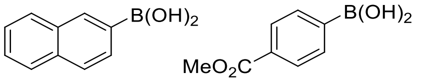

The reaction was carried out according to General Procedure B using naphthalen-2-ylboronic acid (28 mg, 0.16 mmol, 1 equiv), (4-(methoxycarbonyl)phenyl)boronic acid (29 mg, 0.16 mmol, 1 equiv), K_3PO_4 (103 mg, 0.48 mmol, 3 equiv), CPME (0.63 mL, 0.25 M), Oxone® (125 mg, 0.40 mmol, 2.5 equiv) as a slurry in H_2O (1.6 mL) and CPME (0.25 mL). The reaction was run at 70 °C for 30 min. Conversion to products was analyzed by HPLC as outlined in the general procedure indicating selective oxidation of (4-(methoxycarbonyl)phenyl)boronic acid (58%, 3:1).

Naphthalen-2-ylboronic acid (**1a**) *vs.* (4-nitrophenyl)boronic acid (**31a**), Scheme 11

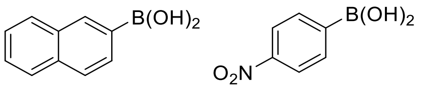

The reaction was carried out according to General Procedure B using naphthalen-2-ylboronic acid (28 mg, 0.16 mmol, 1 equiv), (4-nitrophenyl)boronic acid (27 mg, 0.16 mmol, 1 equiv), K_3PO_4 (103 mg, 0.48 mmol, 3 equiv), CPME (0.63 mL, 0.25 M), Oxone® (125 mg, 0.40 mmol, 2.5 equiv) as a slurry in H_2O (1.6 mL) and CPME (0.25 mL). The reaction was run at 70 °C for 30 min. Conversion to products was analyzed by HPLC as outlined in the general procedure indicating selective oxidation of (4-nitrophenyl)boronic acid (77%, 4.4:1).

Naphthalen-2-ylboronic acid (**1a**) *vs.* (2,3-dihydrobenzo[b][1,4]dioxin-6-yl)boronic acid (**29a**), Table 3, entry 1

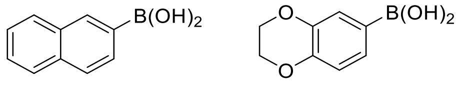

The reaction was carried out according to General Procedure B using naphthalen-2-ylboronic acid (28 mg, 0.16 mmol, 1 equiv), (2,3-dihydrobenzo[b][1,4]dioxin-6-yl)boronic acid (29 mg, 0.16 mmol, 1 equiv), K_3PO_4 (103 mg, 0.48 mmol, 3 equiv), CPME (0.63 mL, 0.25 M), Oxone® (125 mg, 0.40 mmol, 2.5 equiv) as a slurry in H_2O (1.6 mL) and CPME (0.25 mL). The reaction was run at 70 °C for 30 min. Conversion to products was analyzed by HPLC as outlined in the general procedure indicating selective oxidation of (2,3-dihydrobenzo[b][1,4]dioxin-6-yl)boronic acid (86%, 4:1).

Naphthalen-2-ylboronic acid (**1a**) *vs.* (2-methoxypyridin-3-yl)boronic acid (**14a**), Table 3, entry 2

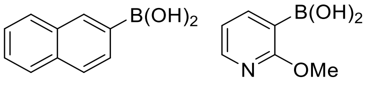

The reaction was carried out according to General Procedure B using naphthalen-2-ylboronic acid (28 mg, 0.16 mmol, 1 equiv), (2-methoxypyridin-3-yl)boronic acid (24 mg, 0.16 mmol, 1 equiv), K_3PO_4 (103 mg, 0.48 mmol, 3 equiv), CPME (0.63 mL, 0.25 M), Oxone® (125 mg, 0.40 mmol, 2.5 equiv) as a slurry in H_2O (1.6 mL) and CPME (0.25 mL). The reaction was run at 70 °C for 30 min. Conversion to products was analyzed by HPLC as outlined in the general procedure indicating selective oxidation of (2-methoxypyridin-3-yl)boronic acid (24 mg, 0.16 mmol, 1 equiv) (62%, 3:1).

(2-Methoxypyridin-3-yl)boronic acid (**14a**) *vs.* (3-isobutoxyphenyl)boronic acid (**26a**), Table 3, entry 3

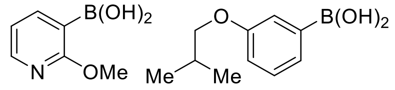

The reaction was carried out according to General Procedure B using (2-methoxypyridin-3-yl)boronic acid (24 mg, 0.16 mmol, 1 equiv), (3-isobutoxyphenyl)boronic acid (31 mg, 0.16 mmol, 1 equiv), K_3PO_4 (103 mg, 0.48 mmol, 3 equiv), CPME (0.63 mL, 0.25 M), Oxone® (125 mg, 0.40 mmol, 2.5 equiv) as a slurry in H_2O (1.6 mL) and CPME (0.25 mL). The reaction was run at 70 °C for 30 min. Conversion to products was analyzed by HPLC as outlined in the general procedure indicating selective oxidation of (2-methoxypyridin-3-yl)boronic acid (71%, 3:1).

Naphthalen-2-ylboronic acid (**1a**) *vs.* pyridin-3-ylboronic acid (**30a**), Table 3, entry 4

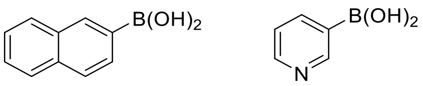

The reaction was carried out according to General Procedure B using naphthalen-2-ylboronic acid (28 mg, 0.16 mmol, 1 equiv), pyridin-3-ylboronic acid (20 mg, 0.16 mmol, 1 equiv), K_3PO_4 (103 mg, 0.48 mmol, 3 equiv), CPME (0.63 mL, 0.25 M), Oxone® (125 mg, 0.40 mmol, 2.5 equiv) as a slurry in H_2O (1.6 mL) and CPME (0.25 mL). The reaction was run at 70 °C for 30 min. Conversion to products was analyzed by HPLC as outlined in the general procedure indicating selective oxidation of pyridin-3-ylboronic acid (85%, 2:1).

(2-Methoxypyridin-3-yl)boronic acid (**14a**) *vs.* (2-bromophenyl)boronic acid (**25a**), Table 3, entry 5

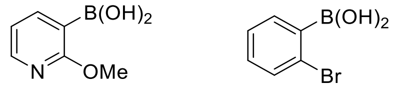

The reaction was carried out according to General Procedure B using (2-methoxypyridin-3-yl)boronic acid (24 mg, 0.16 mmol, 1 equiv), (2-bromophenyl)boronic acid (32 mg, 0.16 mmol, 1 equiv), K_3PO_4 (103 mg, 0.48 mmol, 3 equiv), CPME (0.63 mL, 0.25 M), Oxone® (125 mg, 0.40 mmol, 2.5 equiv) as a slurry in H_2O (1.6 mL) and CPME (0.25 mL). The reaction was run at 70 °C for 30 min. Conversion to products was analyzed by HPLC as outlined in the general procedure indicating selective oxidation of (2-methoxypyridin-3-yl)boronic acid (42%, 2:1).

9. Compound Characterization

9.1. Characterization Data for BMIDA Intermediates

(1*H*-Indol-5-yl)boronic acid, MIDA ester, **8f**

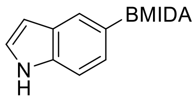

Prepared according to General Procedure I using (1*H*-indol-5-yl)boronic acid (2 g, 12.4 mmol, 1 equiv), *N*-methyliminodiacetic acid (1.9 g, 13.02 mmol, 1.05 equiv), and DMF (50 mL) to afford the desired product as a white solid (3.3 g, 98% yield).

υ_{max} (solid): 3401, 3008, 2962, 1766, 1744, 1578, 1455, 1340, 1245, 1236 cm^{-1}.

^{1}H NMR ($CDCl_3$, 400 MHz): δ 11.02 (s, 1 H), 7.62 (s, 1 H), 7.37 (d, *J* = 8.2 Hz, 1 H), 7.3 (t, *J* = 2.7 Hz, 1 H), 7.14 (d, *J* = 8.2 Hz, 1 H), 6.41 (s, 1 H), 4.30 (d, *J* = 17.2 Hz, 2 H), 4.08 (d, *J* = 17.2 Hz, 2 H), 2.45 (s, 3 H).

^{13}C NMR ($CDCl_3$, 101 MHz): δ 169.5, 136.5, 127.5, 124.9, 124.5, 110.8, 101.1, 61.6, 47.5. Carbon bearing boron not observed.

^{11}B NMR ($CDCl_3$, 128 MHz): δ 12.52.

HRMS: exact mass calculated for $[M+H]^+$ ($C_{13}H_{13}BN_2O_4$) requires *m/z* 273.1041, found *m/z* 273.1045.

(Benzofuran-5-yl)boronic acid, MIDA ester, **13f**

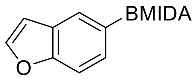

Prepared according to General Procedure I using (benzofuran-5-yl)boronic acid (200 mg, 0.74 mmol, 1 equiv), *N*-methyliminodiacetic acid (107 mg, 0.77 mmol, 1.05 equiv), and DMF (12 mL) to afford desired product as a white solid (284 mg, 85% yield).

υ_{max} (solid): 3145, 3112, 2967, 1760, 1738, 1340, 1260 cm^{-1}.

^{1}H NMR (DMSO-d_6, 400 MHz): δ 7.96 (d, *J* = 2.1 Hz, 1 H), 7.72 (s, 1 H), 7.55–7.58 (m, 1 H), 7.37 (dd, *J* = 8.2, 1.2 Hz, 1 H), 6.96 (dd, *J* = 2.1, 0.9 Hz, 1 H), 4.34 (d, *J* = 17.4 Hz, 2 H), 4.12 (d, *J* = 17.1 Hz, 2 H), 2.48 (s, 3 H).

^{13}C NMR (DMSO-d$_6$, 101 MHz): δ 169.4, 155.1, 145.6, 128.5, 126.9, 125.6, 110.5, 106.7, 61.7, 47.6. Carbon bearing boron not observed.

^{11}B NMR (DMSO-d$_6$, 160 MHz): δ 11.72.

HRMS: exact mass calculated for $[M+H]^+$ ($C_{13}H_{13}BNO_5$) requires *m/z* 274.0881, found *m/z* 274.0886.

4-Hydroxyphenylboronic acid, MIDA ester, **22f**

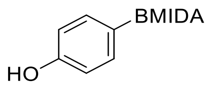

Prepared according to General Procedure I using 4-hydroxyphenylboronic acid (1.75 g, 12.7 mmol, 1 equiv), *N*-methyliminodiacetic acid (1.89 g, 12.8 mmol, 1.01 equiv), and DMF (160 mL) to afford the desired product as a white solid (3 g, 95% yield).

υ_{max} (solid): 3361, 3010, 1740, 1610, 1584 cm^{-1}.

^{1}H NMR (DMSO-d$_6$, 400 MHz): δ 9.39 (br. s., 1 H), 7.21(d, *J* = 8.3 Hz, 2 H), 6.74 (d, *J* = 8.6 Hz, 2 H), 4.27 (d, *J* = 17.2 Hz, 2 H), 4.04 (d, *J* = 17.2 Hz, 2 H), 2.46 (s, 3 H).

^{13}C NMR (DMSO-d$_6$, 101 MHz): δ 169.4, 158.1, 133.6, 114.7, 61.5, 47.4, Carbon bearing boron not observed.

^{11}B NMR (DMSO-d$_6$, 128 MHz): δ 12.20.

HRMS: exact mass calculated for $[M-H]^-$ ($C_{11}H_{11}NO_5B$) requires *m/z* 248.0736, found *m/z* 248.0730.

(3-Isobutoxyphenyl)boronic acid, MIDA ester, **26f**

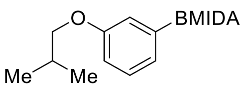

Prepared according to General Procedure I using (3-isobutoxyphenyl)boronic acid (600 mg, 3.1 mmol, 1 equiv), *N*-methyliminodiacetic acid (477 mg, 3.24 mmol, 1.05 equiv), and DMF (30 mL) to afford the desired product as a white solid (900 mg, 95% yield).

υ_{max} (solid): 3004, 2956, 2872, 1768, 1748, 1577, 1457, 1424, 1286, 1253 cm^{-1}.

^{1}H NMR (DMSO-d$_6$, 400 MHz): δ 7.26 (t, *J* = 7.7 Hz, 1 H), 6.99–6.92 (m, 2 H), 6.91 (dd, *J* = 8.1, 2.6 Hz, 1 H), 4.31 (d, *J* = 17.2 Hz, 2 H), 4.10 (d, *J* = 17.2 Hz, 2 H), 3.73 (d, *J* = 6.5 Hz, 2 H), 2.51 (s, 3 H), 2.00 (m, 1 H), 0.98 (d, *J* = 6.7 Hz, 6 H).

^{13}C NMR (DMSO-d$_6$, 101 MHz): δ 169.4, 158.3, 128.8, 124.4, 118.2, 114.7, 73.4, 61.8, 47.5, 27.8, 19.1. Carbon bearing boron not observed.

^{11}B NMR (DMSO-d$_6$, 128 MHz): δ 11.06.

HRMS: exact mass calculated for $[M+H]^+$ ($C_{15}H_{20}BNO_5$) requires *m/z* 305.1507, found *m/z* 305.1513.

Benzene-1-boronic acid, pinacol ester-4-boronic acid, MIDA ester, **27**

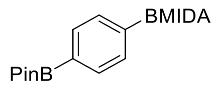

(4-Bromophenyl)boronic acid, MIDA ester (78 mg, 0.25 mmol, 1 equiv), bis(pinacolato)diboron (91 mg, 0.36 mmol, 1,4 equiv), Pd(dppf)Cl_2·CH_2Cl_2 (10 mg, 0.0125 mmol, 5 mol%), and KOAc (81 mg, 0.825 mmol, 3.3 equiv) were weighed out into an oven-dried 5 mL microwave vial. The vial was capped and purged with nitrogen. DMSO (2 mL, 0.125 M) was added *via* syringe and the reaction was heated to 75 °C in a sand bath with stirring for 24 h. The reaction was allowed to cool to room temperature and was vented, decapped, and poured into EtOAc (50 mL) and H_2O (40 mL) was added. Organics were separated and washed with water (2 x 40 mL). The aqueous layer was extracted with a further 25 mL EtOAc and both organics combined. Organics were passed through a hydrophobic frit and concentrated under vacuum. Crude product was purified by flash chromatography (silica gel, 10-70% acetone in ether) to afford title compound as a white crystalline solid (76 mg, 85% yield).

υ_{max} (solid): 2978, 1761, 1748, 1517, 1457, 1362 cm^{-1}.

^{1}H NMR (DMSO-d$_6$, 400 MHz): δ 7.66 (d, *J* = 8.0 Hz, 2H), 7.45 (d, *J* = 8.0 Hz, 2H), 4.34 (d, *J* = 17.2 Hz, 2H), 4.10 (d, *J* = 17.2 Hz, 2H), 2.46 (s, 3H), 1.29 (s, 12H).

^{13}C NMR (DMSO-d$_6$, 101 MHz): δ 169.3, 133.6, 131.8, 83.6, 61.8, 47.6, 24.7.

^{11}B NMR (DMSO-d$_6$, 128 MHz): δ 32.46, 11.78.

HRMS: exact mass calculated for $[M-H]^-$ ($C_{17}H_{22}B_2NO_6$) requires *m/z* 358.1639, found *m/z* 358.1634.

9.2. Characterization Data for NMR Analysis

Potassium trihydroxy(naphthalen-2-yl)borate, **1d**

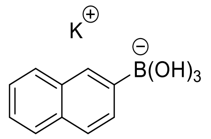

Prepared according to General Procedure F using naphthalen-2-ylboronic acid (6.1 mg, 0.036 mmol, 1 equiv) and K_3PO_4 (22.7 mg, 0.11 mmol, 3 equiv), and D_2O (0.75 ml). The NMR sample was run at 343 K.

^{11}B NMR (D_2O, 128 MHz): δ 3.67.

Potassium [1,1'-biphenyl]-4-yltrihydroxyborate, pinacol ester, **2e**

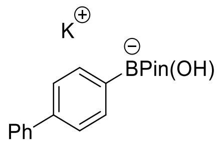

Prepared according to General Procedure F using [1,1'-biphenyl]-4-ylboronic acid, pinacol ester (10 mg, 0.036 mmol, 1 equiv), **base** (0.11 mmol, 3 equiv), and D_2O (0.75 mL). The NMR sample was run at **X** K.

Entry	Base (mass)	Temp (K)	^{11}B Signal
**1**[a]	K_3PO_4 (22.7 mg)	293	-
**2**[a]	K_3PO_4 (22.7 mg)	343	-
**3**	KOH (6 mg)	293	6.0 ppm
**4**[b]	KOH (6 mg)	343	3.57 ppm

[a]-starting material did not form a solution to transfer into the NMR tube, [b]-BPin boronate hydrolysis to the corresponding boronic acid boronate occurred.

Potassium (4-fluorophenyl)trihydroxyborate, **3d**

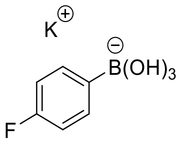

Prepared according to General Procedure F using (4-fluorophenyl)boronic acid (5 mg, 0.036 mmol, 1 equiv) and K_3PO_4 (22.7 mg, 0.11 mmol, 3 equiv), and D_2O (0.75 ml). The NMR sample was run at 343 K.

^{11}B NMR (D_2O, 128 MHz): δ 3.49.

^{19}F NMR (D_2O, 376 MHz): δ –118.65.

Potassium (4-fluorophenyl)trihydroxyborate, pinacol ester, **3e**

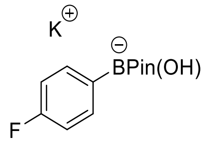

Prepared according to General Procedure F using (4-fluorophenyl)boronic acid (5 mg, 0.036 mmol, 1 equiv) and K_3PO_4 (22.7 mg, 0.11 mmol, 3 equiv), and D_2O (0.75 ml). The NMR sample was run at 343 K. Hydrolysis of BPin boronate was seen by NMR (3.49 and -188.65 ppm for ^{11}B and ^{19}F NMR respectively).

^{11}B NMR (D_2O, 128 MHz, 343 K): δ 6.24.

^{19}F NMR (D_2O, 376 MHz, 343 K): δ –119.07.

9.3. Characterisation Data for Oxidative Nucleophile Coupling (Scheme 12)

2-([1,1'-Biphenyl]-4-yloxy)naphthalene, **32**

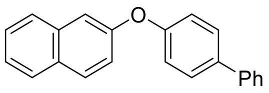

Prepared according to General Procedure J using naphthalen-2-ylboronic acid (28 mg, 0.16 mmol, 1 equiv), [1,1'-biphenyl]-4-ylboronic acid, pinacol ester (225 mg, 0.8 mmol, 5 equiv), K_3PO_4 (103

mg, 0.48 mmol, 3 equiv), CPME (0.63 mL, 0.25 M), and Oxone® (125 mg, 0.40 mmol, 2.5 equiv) as a slurry in H_2O (1.28 mL) and CPME (0.25 mL), $Cu(OAc)_2$ (58 mg, 0.32 mmol, 2 equiv), powdered activated molecular sieves, MeCN (350 μ*L*), EtOH (16 μL), and Et_3N (45 μL, 0.32 mmol, 2 equiv) to afford title compound as a white solid (39.1 mg, 82% yield).

υ_{max} (solid): 3055, 3032, 2922, 2852, 1597, 1588 cm^{-1}.

^1H NMR ($CDCl_3$, 400 MHz): δ 7.85 (t, J = 8.3 Hz, 2H), 7.73 (d, J = 8.1 Hz, 1H), 7.62–7.56 (m, 4H), 7.50–7.40 (m, 4H), 7.34 (m, 3H), 7.15 (d, J = 8.8 Hz, 2H).

^{13}C NMR ($CDCl_3$, 101 MHz): δ 156.9, 155.1, 140.7, 136.7, 134.5, 130.4, 130.1, 129.0, 128.7, 127.9, 127.3, 127.2, 127.1, 126.7, 124.9, 120.2, 119.4, 114.5.

HRMS: exact mass calculated for $[M]^+$ ($C_{22}H_{16}O$) requires *m/z* 296.1201, found *m/z* 296.1208.

Methyl 4-(4-(trifluoromethyl)phenoxy)benzoate, **33**

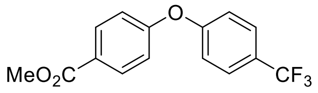

Prepared according to General Procedure J using (4-(methoxycarbonyl)phenyl)boronic acid (29 mg, 0.16 mmol, 1 equiv), (4-(trifluoromethyl)phenyl)boronic acid, pinacol ester (217 mg, 0.8 mmol, 5 equiv), K_3PO_4 (103 mg, 0.48 mmol, 3 equiv), CPME (0.63 mL, 0.25 M), and Oxone® (125 mg, 0.40 mmol, 2.5 equiv) as a slurry in H_2O (1.28 mL) and CPME (0.25 mL), $Cu(OAc)_2$ (58 mg, 0.32 mmol, 2 equiv), powdered activated molecular sieves, MeCN (350 μ*L*), EtOH (16 μL), and Et_3N (45 μL, 0.32 mmol, 2 equiv) to afford title compound as an off white solid (34.6 mg, 73% yield).

υ_{max} (solid): 3075, 2960, 2922, 1722, 1599, 1506, 1433 cm^{-1}.

^1H NMR ($CDCl_3$, 400 MHz): δ 8.09–8.03 (m, 2H), 7.63 (d, J = 8.4 Hz, 2H), 7.16–7.09 (m, 2H), 7.09–7.02 (m, 2H), 3.92 (s, 3H).

^{13}C NMR ($CDCl_3$, 101 MHz): δ 166.4, 160.2, 158.9, 131.9, 127.4 (q, $^3J_{C\text{-}F}$ = 3.1 Hz), 126.2 (app. d, $^2J_{C\text{-}F}$ = 33.0 Hz), 125.8, 124.0 (app. d, $^1J_{C\text{-}F}$ = 271.4 Hz), 119.2, 118.5, 52.1.

^{19}F NMR ($CDCl_3$, 376 MHz): δ -62.0 (s, 3F).

HRMS: exact mass calculated for $[M+H]^+$ ($C_{15}H_{12}F_3O$) requires *m/z* 297.0736, found *m/z* 297.0733.

5-Phenoxybenzofuran, **34**

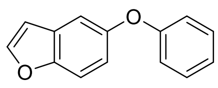

Prepared according to General Procedure J using benzofuran-5-ylboronic acid (26 mg, 0.16 mmol, 1 equiv), phenyl boronic acid, pinacol ester (164 mg, 0.8 mmol, 5 equiv), K_3PO_4 (103 mg, 0.48 mmol, 3 equiv), CPME (0.63 mL, 0.25 M), and Oxone® (125 mg, 0.40 mmol, 2.5 equiv) as a slurry in H_2O (1.28 mL) and CPME (0.25 mL), $Cu(OAc)_2$ (58 mg, 0.32 mmol, 2 equiv), powdered activated molecular sieves, MeCN (350 μ*L*), EtOH (16 μL), and Et_3N (45 μL, 0.32 mmol, 2 equiv) to afford an inseparable mixture (60:40) of title compound and phenyl boronic acid, pinacol ester, (36.2 mg, 64% NMR yield).

υ_{max} (Film): 3064, 3040, 2922, 1590, 1491, 1457, 1217, 1184 cm^{-1}.

^1H NMR ($CDCl_3$, 400 MHz): δ 7.65 (d, *J* = 2.2 Hz, 1H), 7.48 (d, *J* = 8.8 Hz, 1H), 7.36–7.29 (m, 2H), 7.24 (d, *J* = 2.4 Hz, 1H), 7.10–6.97 (m, 4H), 6.72 (dd, *J* = 2.2, 0.9 Hz, 1H).

^{13}C NMR ($CDCl_3$, 101 MHz): δ 158.7, 152.6, 151.7, 146.3, 129.8, 128.5, 122.7, 118.1, 117.2, 112.3, 111.6, 106.9.

HRMS: exact mass calculated for $[M]^+$ ($C_{14}H_{10}O_2$) requires *m/z* 210.0681, found *m/z* 210.0651.

5-(4-Fluorophenoxy)benzofuran, **35**

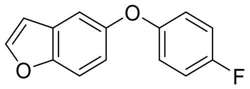

Prepared according to General Procedure J using benzofuran-5-ylboronic acid (26 mg, 0.16 mmol, 1 equiv), (4-fluorophenyl)boronic acid, pinacol ester (178 mg, 0.8 mmol, 5 equiv), K_3PO_4 (103 mg, 0.48 mmol, 3 equiv), CPME (0.63 mL, 0.25 M), and Oxone® (125 mg, 0.40 mmol, 2.5 equiv) as a slurry in H_2O (1.28 mL) and CPME (0.25 mL), $Cu(OAc)_2$ (58 mg, 0.32 mmol, 2 equiv), powdered activated molecular sieves, MeCN (350 μ*L*), EtOH (16 μL), and Et_3N (45 μL, 0.32 mmol, 2 equiv) to afford an inseparable mixture (80:20) of title compound and (4-fluorophenyl)boronic acid, pinacol ester (33.6 mg, 73% NMR yield).

υ_{max} (Film): 3116, 3073, 2922, 1500, 1461, 1197, 1184 cm^{-1}.

^1H NMR ($CDCl_3$, 400 MHz): δ 7.64 (d, *J* = 2.2 Hz, 1H), 7.45 (s, 1H), 7.18 (d, *J* = 2.5 Hz, 1H), 7.04–6.93 (m, 5H), 6.72 (dd, *J* = 2.2, 0.9 Hz, 1H).

^{13}C NMR ($CDCl_3$, 101 MHz): δ 158.6 (d, $^1J_{C-F}$ = 241.1 Hz), 154.4, 153.2, 151.6, 146.4, 128.5, 119.6 (d, $^3J_{C-F}$ = 7.9 Hz), 116.5 (d, $^2J_{C-F}$ = 21.1 Hz), 116.2, 112.3, 110.9, 106.9.

^{19}F NMR ($CDCl_3$, 376 MHz): δ -121.1 (m, 1F).

HRMS: exact mass calculated for $[M]^+$ ($C_{14}H_9FO_2$) requires *m/z* 228.0587, found *m/z* 228.0582.

1,2,3-Trimethoxy-5-(4-methoxyphenoxy)benzene, **36**

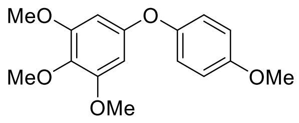

Prepared according to General Procedure J using (3,4,5-trimethoxyphenyl)boronic acid (34 mg, 0.16 mmol, 1 equiv), (4-methoxyphenyl)boronic acid, pinacol ester (187 mg, 0.8 mmol, 5 equiv), K_3PO_4 (103 mg, 0.48 mmol, 3 equiv), CPME (0.63 mL, 0.25 M), and Oxone® (125 mg, 0.40 mmol, 2.5 equiv) as a slurry in H_2O (1.6 mL) and CPME (0.25 mL), $Cu(OAc)_2$ (58 mg, 0.32 mmol, 2 equiv), powdered activated molecular sieves, MeCN (350 µ*L*), EtOH (16 µL), and Et_3N (45 µL, 0.32 mmol, 2 equiv) to afford title compound as a clear gum (16.8 mg, 36% yield).

υ_{max} (Film): 3001, 2935, 2837, 1601, 1498, 1213, 1132 cm^{-1}.

^1H NMR (500 MHz, $CDCl_3$) δ 6.97 (d, *J* = 9.1 Hz, 1H), 6.88 (d, *J* = 9.1 Hz, 1H), 6.20 (s, 1H), 3.81 (d, *J* = 1.4 Hz, 3H), 3.78 (s, 3H).

^{13}C NMR ($CDCl_3$, 101 MHz): δ 155.3, 154.1, 153.3, 149.9, 133.1, 119.8, 114.3, 95.1, 60.5, 55.6, 55.2.

HRMS: exact mass calculated for $[M]^+$ ($C_{16}H_{18}O_5$) requires *m/z* 290.1154, found *m/z* 290.1152.

1-Methyl-3-(4-(trifluoromethoxy)phenoxy)benzene, **37**

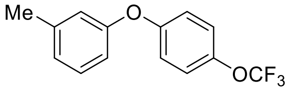

Prepared according to General Procedure J using (4-(trifluoromethoxy)phenyl)boronic acid (33 mg, 0.16 mmol, 1 equiv), *m*-tolylboronic acid, pinacol ester (175 mg, 0.8 mmol, 5 equiv), K_3PO_4 (103 mg, 0.48 mmol, 3 equiv), CPME (0.63 mL, 0.25 M), and Oxone® (125 mg, 0.40 mmol, 2.5 equiv) as a slurry in H_2O (1.28 mL) and CPME (0.25 mL), $Cu(OAc)_2$ (58 mg, 0.32 mmol, 2 equiv), powdered activated molecular sieves, MeCN (350 µ*L*), EtOH (16 µL), and Et_3N (45 µL, 0.32 mmol, 2 equiv) to afford title compound as a clear gum (26.2 mg, 61% yield).

υ_{max} (Film): 2926, 1608, 1588, 1502, 1489, 1251, 1193 cm^{-1}.

^{1}H NMR ($CDCl_3$, 400 MHz): δ 7.23 (d, *J* = 7.8 Hz, 1H), 7.17 (dd, *J* = 9.0, 0.7 Hz, 2H), 7.01–6.94 (m, 3H), 6.83 (d, *J* = 5.8 Hz, 2H), 2.35 (s, 3H).

^{13}C NMR ($CDCl_3$, 101 MHz): δ 156.8, 156.2, 144.5, 140.3, 129.8, 124.8, 122.7, 120.7 (q, $^1J_{C\text{-}F}$ = 256.3 Hz). 120.0, 119.6, 116.3, 21.5.

^{19}F NMR $CDCl_3$, 376 MHz): δ – 58.3 (s, 3F).

HRMS: exact mass calculated for $[M]^+$ ($C_{14}H_{11}F_3O_2$) requires *m/z* 268.0711, found *m/z* 268.0724.

9.4. Assay Characterization

Naphthalen-2-ylboronic acid, pinacol ester,[3] **1b**

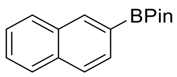

υ_{max} (solid): 3052, 2978, 2971, 1629, 1599 cm^{-1}.

^{1}H NMR ($CDCl_3$, 500 MHz): δ 8.38 (s, 1 H), 7.88–7.91 (m, 1 H), 7.82–7.86 (m, 3 H), 7.46–7.55 (m, 2 H), 1.41 (s, 12 H).

Naphthalen-2-ol,[4] **1c**

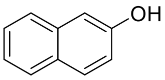

υ_{max} (solid): 3241, 3053, 3043, 1744, 1630 cm^{-1}.

^{1}H NMR (DMSO-d_6, 500 MHz): δ 7.75–7.80 (m, 2 H), 7.69 (d, *J* = 8.2 Hz, 1 H), 7.44 (m, 1 H), 7.34 (m, 1 H), 7.16 (d, *J* = 2.4 Hz, 1 H), 7.11 (dd, *J* = 8.9, 2.4 Hz, 1 H). OH proton not observed.

[1,1'-Biphenyl]-4-ylboronic acid,[5] **2a**

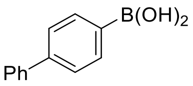

υ_{max} (solid): 3344, 3054, 3034, 1608, 1552 cm^{-1}.

^{1}H NMR (DMSO-d_6, 500 MHz): δ 8.07 (s, 2 H), 7.89 (d, J = 8.2 Hz, 2 H), 7.69 (d, J = 7.3 Hz, 2 H), 7.64 (d, *J* = 7.9 Hz, 2 H), 7.48 (t, *J* = 7.8 Hz, 2 H), 7.38 (t, *J* = 7.3 Hz, 1 H).

[1,1'-Biphenyl]-4-ylboronic acid, pinacol ester,[6] **2b**

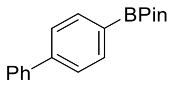

$υ_{max}$ (solid): 3034, 2976, 1612, 1400, 1361 cm^{-1}.

^{1}H NMR ($CDCl_3$, 500 MHz): δ 7.92 (d, *J* = 8.2 Hz, 2 H), 7.63–7.66 (m, 4 H), 7.45–7.49 (m, 2 H), 7.38, (m, 1 H), 1.39 (s, 12 H).

[1,1'-Biphenyl]-4-ol,[7] **2c**

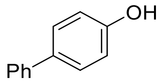

$υ_{max}$ (solid): 3378, 3036, 2921, 1610, 1597 cm^{-1}.

^{1}H NMR (DMSO-d_6, 500 MHz): δ 9.51 (s, 1 H), 7.54–7.59 (m, 2 H), 7.46–7.50 (m, 2 H), 7.40 (t, *J* = 7.8 Hz, 2 H), 7.25–7.29 (m, 1 H), 6.82–6.87 (m, 2 H).

4-Fluorophenol,[8] **3c**

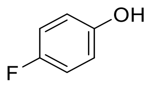

$υ_{max}$ (solid): 3181, 2898, 2682, 1871, 1506, 1448 cm^{-1}.

^{1}H NMR (DMSO-d_6, 400 MHz): δ 6.90–6.97 (m, 2 H), 6.75–6.81 (m, 2 H). OH proton not observed.

Phenol,[9] **4c**

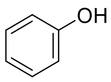

$υ_{max}$ (solid): 3211, 3045, 3023, 2960, 1595 cm^{-1}.

^{1}H NMR (DMSO-d_6, 500 MHz): δ 7.23–7.27 (m, 2 H), 6.94 (m, 1 H), 6.83–6.86 (m, 2 H). OH proton not observed.

4-Methoxyphenol,[10] **5c**

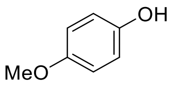

υ_{max} (solid): 3378, 3032, 3013, 2950, 2833, 1504, 1452, 1442 cm^{-1}.

^{1}H NMR (DMSO-d_6, 500 MHz): δ 6.76–6.82 (m, 4 H), 3.77 (s, 3 H). OH proton not observed.

4-Acetamidophenol,[11] **6c**

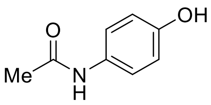

υ_{max} (solid): 3323, 3163, 3110, 1653, 1612, 1565, 1508 cm^{-1}.

^{1}H NMR (DMSO-d_6, 500 MHz): δ 9.62 (s, 1 H), 9.10 (s, 1 H), 7.30–7.35 (m, 2 H), 6.64–6.69 (m, 2 H), 1.97 (s, 3 H). OH proton not observed.

Methyl 4-hydroxybenzoate,[12] **7c**

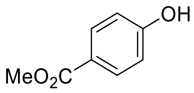

υ_{max} (solid): 3306, 2962, 1748, 1679, 1588 cm^{-1}.

^{1}H NMR ($CDCl_3$, 500 MHz): δ 7.95–7.99 (m, 2 H), 6.85–6.89 (m, 2 H), 3.90 (s, 3 H). OH proton not observed.

1*H*-Indol-5-ol,[13] **8c**

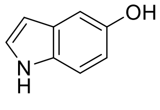

υ_{max} (solid): 3330, 2954, 2922, 2852, 1467 cm^{-1}.

^{1}H NMR ($CDCl_3$, 500 MHz): δ 7.64–7.71 (m, 2 H), 7.52–7.58 (m, 1 H), 7.44–7.50 (m, 2 H). OH and NH protons not observed.

p-Cresol,[14] **9c**

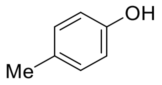

υ_{max} (film): 3333, 2967, 2960, 1613, 1600, 1509, 1500, 1223 cm^{-1}.

^{1}H NMR ($CDCl_3$, 500 MHz): δ 7.03–7.06 (m, 2 H), 6.72–6.76 (m, 2 H), 2.28 (s, 3 H). OH proton not observed.

2-Nitrophenol,[15] **10c**

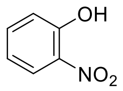

υ_{max} (solid): 3237, 3114, 3093, 1612, 1589, 1580, 1532, 1476, 1446 cm^{-1}.

^{1}H NMR ($CDCl_3$, 500 MHz): δ 10.60 (s, 1 H), 8.13 (dd, *J* = 8.5, 1.5 Hz, 1 H), 7.57–7.62 (m, 1 H), 7.18 (dd, *J* = 8.5, 1.2 Hz, 1 H), 6.98–7.03 (m, 1 H).

2,4,6-Trimethylphenol,[16] **11c**

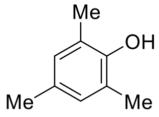

υ_{max} (solid): 3388, 3016, 2975, 2917, 2857, 1485 cm^{-1}.

^{1}H NMR (DMSO-d_6, 500 MHz): δ 6.80 (s, 2 H), 2.21–2.24 (m, 9 H). OH proton not observed.

3-Bromophenol,[17] **12c**

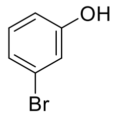

υ_{max} (film): 3629, 3426, 1599, 1582, 1474, 1439, 1296 cm^{-1}.

^{1}H NMR ($CDCl_3$, 500 MHz): δ 7.06–7.13 (m, 2 H), 7.03 (t, *J* = 2.0 Hz, 1 H), 6.78 (m, 1 H). OH proton not observed.

Benzofuran-5-ol,[18] **13c**

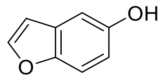

υ_{max} (film): 3315, 1621, 1597, 1465, 1454, 1191 cm^{-1}.

^{1}H NMR ($CDCl_3$, 500 MHz): δ 7.60 (d, *J* = 2.1 Hz, 1 H), 7.37 (d, *J* = 8.5 Hz, 1 H), 7.03 (d, = 2.4 Hz, 1 H), 6.83 (dd, *J* = 8.9, 2.4 Hz, 1 H), 6.68 (dd, *J* = 2.1, 0.9 Hz, 1 H). OH proton not observed.

2-Methoxypyridin-3-ol,[19] **14c**

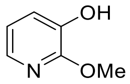

υ_{max} (solid): 3049, 2963, 2889, 2839, 2683, 2660, 1602, 1498, 1455, 1429, 1264 cm^{-1}.

^{1}H NMR ($CDCl_3$, 500 MHz): δ 7.71 (d, *J* = 4.9 Hz, 1 H), 7.14 (d, *J* = 7.9 Hz, 1 H), 6.83 (dd, *J* = 7.5, 5.0 Hz, 1 H), 4.05 (s, 3 H). OH proton not observed.

2,4-Difluorophenylboronic acid, pinacol ester,[20] **15b**

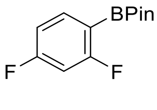

υ_{max} (solid): 3075, 2980, 1617, 1595 cm^{-1}.

^{1}H NMR (DMSO-d_6, 500 MHz): δ 7.70–7.76 (m, 1 H), 6.85–6.90 (m, 1 H), 6.74–6.80 (m, 1 H), 1.36 (s, 12 H).

4-Cyanophenylboronic acid, pinacol ester,[21] **16b**

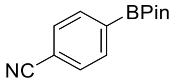

υ_{max} (solid): 3006, 2974, 2932, 2227, 1400, 1381, 1355 cm^{-1}.

^{1}H NMR (DMSO-d_6, 500 MHz): δ 7.88–7.91 (m, 2 H), 7.63–7.66 (m, 2 H), 1.36 (m, 12 H).

(Benzofuran-2-yl)boronic acid, pinacol ester,[22] **17b**

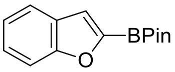

υ_{max} (solid): 3060, 2974, 2928, 1567, 1361, 1325 cm^{-1}.

^{1}H NMR (DMSO-d_6, 500 MHz): δ 7.64 (d, J = 7.9 Hz, 1 H), 7.58 (d, J = 8.2 Hz, 1 H), 7.41 (s, 1 H), 7.35 (td, J = 7.8, 1.2 Hz, 1 H), 7.22–7.26 (m, 1 H), 1.40 (s, 12 H).

Thiophen-2-ylboronic acid,[23] **18a**

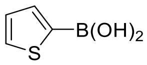

υ_{max} (solid): 3215, 1521, 1424, 1359 cm^{-1}.

^{1}H NMR (DMSO-d_6, 500 MHz): δ 8.16 (s, 2 H), 7.74 (d, J = 4.9 Hz, 1 H), 7.68 (d, J = 3.1 Hz, 1 H), 7.15–7.18 (m, 1 H).

Thiophen-2-ylboronic acid, pinacol ester,[24] **18b**

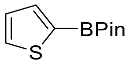

υ_{max} (solid): 3101, 2978, 1523, 1426 cm^{-1}.

^{1}H NMR (DMSO-d_6, 500 MHz): δ 7.59 (dd, J = 3.5, 0.8 Hz, 1 H), 7.57 (dd, J = 4.7, 0.8 Hz, 1 H), 7.20 (dd, J = 4.6, 3.4 Hz, 1 H), 1.36 (s, 12 H).

Isoquinolin-4-ylboronic acid, pinacol ester,[25] **19b**

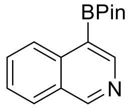

υ_{max} (solid): 2980, 2930, 1630, 1498 cm^{-1}.

^{1}H NMR (DMSO-d$_6$, 500 MHz): δ 9.48–9.51 (m, 1 H), 8.98 (d, *J* = 8.8 Hz, 1 H), 8.95 (s, 1 H), 8.30 (d, *J* = 8.2 Hz, 1 H), 8.15–8.19 (m, 1 H), 7.94–7.99 (m, 1 H), 1.45 (s, 12 H).

2-Aminophenol,[26] **20c**

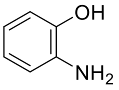

υ_{max} (solid): 3372, 3300, 3049, 2709, 2584, 1600, 1511, 1461, 1403 cm^{-1}.

^{1}H NMR (DMSO-d$_6$, 500 MHz): δ 8.88 (br. s, 1 H), 6.60–6.64 (m, 1 H), 6.55–6.59 (m, 1 H), 6.52 (m, 1 H), 6.38 (m, 1 H), 4.44 (br. s, 2 H).

4-Isopropylphenol,[27] **21c**

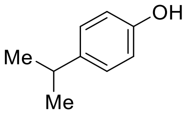

υ_{max} (solid): 3291, 3017, 2976, 2952, 1614, 1601, 1513 cm^{-1}.

^{1}H NMR ($CDCl_3$, 500 MHz): δ 7.09–7.13 (m, 2 H), 6.75–6.79 (m, 2 H), 2.86 (sept, *J* = 7.0 Hz, 1 H), 1.23 (d, *J* = 6.7 Hz, 6 H). OH proton not observed.

Hydroquinone,[28] **22c**

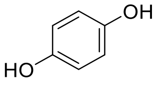

υ_{max} (solid): 3136, 3028, 1515, 1463, 1353 cm^{-1}.

^{1}H NMR (DMSO-d$_6$, 500 MHz): δ 8.59 (s, 2 H), 6.55 (s, 4 H).

2-Chlorophenol,[29] **23c**

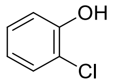

υ_{max} (film): 3514, 3073, 3038, 1595, 1584, 1480, 1452 cm^{-1}.

^{1}H NMR ($CDCl_3$, 500 MHz): δ 7.33 (dd, J = 8.2, 1.5 Hz, 1 H), 7.16–7.22 (m, 1 H), 7.04 (dd, J = 8.2, 1.5 Hz, 1 H), 6.86–6.91 (m, 1 H), 5.56 (br. s, 1 H).

(6-Methoxypyridin-3-yl)boronic acid, pinacol ester,[30] **24b**

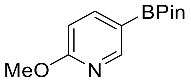

υ_{max} (solid): 3010, 2973, 2948, 2846, 1599, 1563, 1355 cm^{-1}.

^{1}H NMR (DMSO-d_6, 500 MHz): δ 8.56 (d, J = 1.8 Hz, 1 H), 7.94 (d, J = 8.2 Hz, 1 H), 6.73 (d, J = 8.2 Hz, 1 H), 3.98 (s, 3 H), 1.35 (m, 12 H).

2-Bromophenol,[31] **25c**

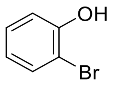

υ_{max} (film): 3493, 3069, 1576, 1474, 1448 cm^{-1}.

^{1}H NMR ($CDCl_3$, 500 MHz): δ 7.47 (dd, J = 7.9, 1.5 Hz, 1 H), 7.21–7.25 (m, 1 H), 7.04 (dd, J = 8.2, 1.5 Hz, 1 H), 6.80–6.84 (m, 1 H), 5.52 (br. s, 1 H).

3-Isobutoxyphenol,[32] **26c**

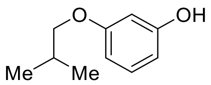

υ_{max} (film): 3396, 2960, 2872, 1595, 1495, 1470, 1288, 1149 cm^{-1}.

^{1}H NMR ($CDCl_3$, 500 MHz): δ 7.10–7.14 (m, 1 H), 6.48–6.52 (m, 1 H), 6.39–6.42 (m, 2 H), 3.70 (d, *J* = 6.5, Hz, 2 H), 2.08 (t, *J* = 6.5 Hz, 1 H), 1.02 (d, *J* = 6.5 Hz, 6 H). OH proton not observed.

4-Nitrophenol,[33] **26c**

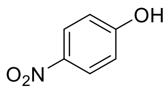

υ_{max} (film): 3309, 1612, 1585, 1489, 1284, 1213 cm^{-1}.

^{1}H NMR ($CDCl_3$, 500 MHz): δ 11.02 (s, 1H), 8.18–8.03 (m, 2H), 6.99–6.86 (m, 2H).

2,3-Dihydrobenzo[b][1,4]dioxin-6-ol,[34] **30c**

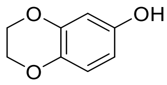

υ_{max} (film): 3385, 2922, 2874, 1608, 1509, 1468, 1454, 1312 cm^{-1}.

^{1}H NMR ($CDCl_3$, 500 MHz): δ 6.72 (d, *J* = 8.5 Hz, 1 H), 6.39 (d, *J* = 2.5 Hz, 1 H), 6.31–6.35 (m, 1 H), 4.22–4.26 (m, 2 H), 4.19–4.22 (m, 2 H). OH proton not observed.

Pyridin-3-ol,[35] **31c**

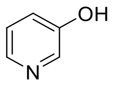

υ_{max} (film): 2422, 1790, 1573, 1478, 1374 cm^{-1}.

^{1}H NMR ($CDCl_3$, 500 MHz): δ 8.32 (d, *J* = 2.7 Hz, 1H), 8.08 (dd, *J* = 4.8, 1.2 Hz, 1H), 7.40 (m, 1H), 7.33 (dd, *J* = 8.4, 4.8 Hz, 1H), 6.93 (s, 1H).

10. References

1. W. L. F. Armarego, C. Chai, Purification of Laboratory Chemicals, 7th ed., Elsevier, Oxford, 2013.
2. JChem for Office 15.12.1400.458, 2008-2015, ChemAxon (http://www.chemaxon.com)
3. Sigma Aldrich, CAS number (256652-04-7), Catalogue number (BML00028).
4. Sigma Aldrich, CAS number (135-19-3), Catalogue number (185507).
5. Sigma Aldrich, CAS number (5122-94-1), Catalogue number (483451).
6. Sigma Aldrich, CAS number (144432-80-4), Catalogue number (BML00125).
7. Sigma Aldrich, CAS number (92-69-3), Catalogue number (134341).
8. Sigma Aldrich, CAS number (371-41-5), Catalogue number (F13207).
9. Sigma Aldrich, CAS number (108-95-2), Catalogue number (185450).
10. Sigma Aldrich, CAS number (150-76-5), Catalogue number (M18655).
11. Alfa Aesar, CAS number (103-90-2), Catalogue number (A11240).
12. Sigma Aldrich, CAS number (99-76-3), Catalogue number (H5501).
13. Sigma Aldrich, CAS number (1953-54-4), Catalogue number (H31859).
14. Sigma Aldrich, CAS number (106-44-5), Catalogue number (C85751).
15. Sigma Aldrich, CAS number (88-75-5), Catalogue number (33444).
16. Sigma Aldrich, CAS number (527-60-6), Catalogue number (T79006).
17. Sigma Aldrich, CAS number (591-20-8), Catalogue number (101079).
18. Apollo Scientific, CAS number (13196-10-6), Catalogue number (OR42004).
19. Combi-Blocks, CAS number (13472-83-8), Catalogue number (YF-6136).
20. Sigma Aldrich, CAS number (288101-48-4), Catalogue number (632694).
21. Sigma Aldrich, CAS number (171364-82-2), Catalogue number (527556).
22. Apollo Scientific, CAS number (402503-13-3), Catalogue number (OR303556).

23. Sigma Aldrich, CAS number (6165-68-0), Catalogue number (436836).

24. Sigma Aldrich, CAS number (193978-23-3), Catalogue number (CDS004652).

25. Sigma Aldrich, CAS number (685103-98-4), Catalogue number (648795).

26. Sigma Aldrich, CAS number (95-55-6), Catalogue number (A71301).

27. Sigma Aldrich, CAS number (99-89-8), Catalogue number (175404).

28. Sigma Aldrich, CAS number (123-31-9), Catalogue number (H9003).

29. Sigma Aldrich, CAS number (95-57-8), Catalogue number (C62794).

30. Sigma Aldrich, CAS number (445264-61-9), Catalogue number (636029).

31. Sigma Aldrich, CAS number (95-56-7), Catalogue number (130915).

32. Combi-Blocks, CAS number (91950-13-9), Catalogue number (YF-6693).

33. Sigma Aldrich, CAS number (100-02-7), Catalogue number (241326).

34. Fluorochem, CAS number (10288-72-9), Catalogue number (209092).

35. Sigma Aldrich, CAS number (109-00-2), Catalogue number (H57009).

11. HPLC Retention Times and Conversion Factors of Products

Compound	Structure	Retention Time (min)	Conversion Factor
**1b**	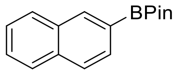	10.4	0.42
**1c**	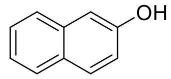	6.2	0.60
**2a**	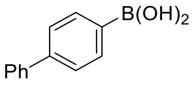	6.6	5.22
**2b**	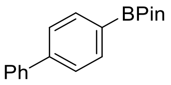	10.8	4.38
**2c**	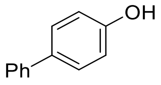	7.3	4.02
**3c**	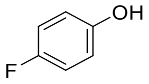	4.2	0.03

**4c**	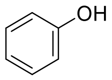	3.5	0.07
**5c**	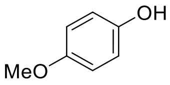	3.4	0.12
**6c**	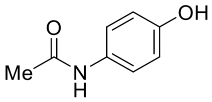	1.8	2.98
**7c**	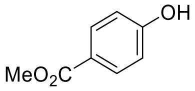	4.3	3.50
**8c**	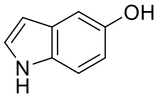	2.7	0.47
**9c**	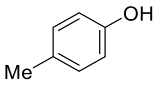	4.8	0.03
**10c**	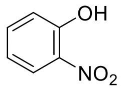	5.5	0.65
**11c**	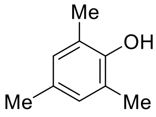	7.1	0.04
**12c**	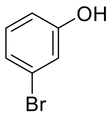	6.1	0.06
**13c**	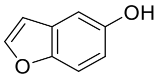	4.5	3.34
**14c**	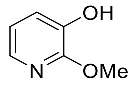	2.2	0.16
**15b**	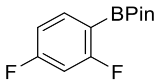	3.7	0.05
**16b**	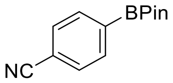	3.4	0.32
**17b**	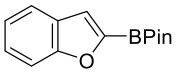	4.5	3.67
**18a**	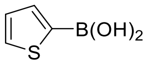	2.5	2.08
**18b**	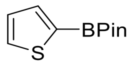	8.7	2.23
**19b**	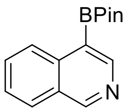	1.1	0.17

**20c**	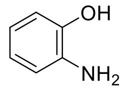	3.0	0.07
**21c**	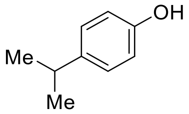	6.9	0.05
**22c**	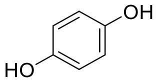	1.4	0.05
**23c**	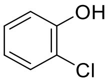	5.2	0.07
**24b**	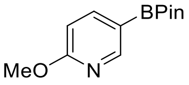	1.8	0.25
**25c**	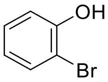	5.6	0.06
**26c**	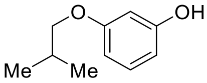	7.4	0.06
**29c**	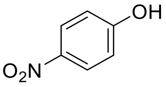	4.7	0.37
**30c**	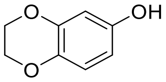	3.5	0.07
**31c**	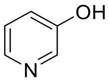	0.5	0.09

12. HPLC Spectra

HPLC of naphthalen-2-ylboronic acid (**1a**) *vs.* [1,1'-biphenyl]-4-ylboronic acid, pinacol ester (**2b**)

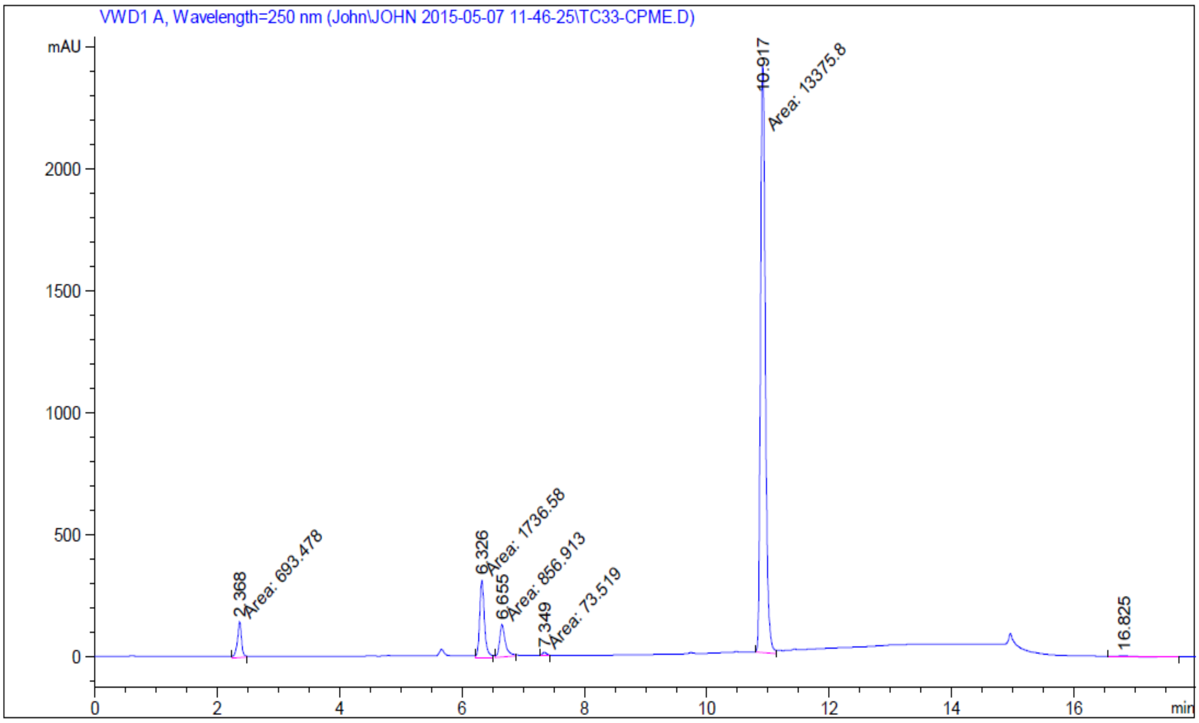

Areas: caf = 693.478, **1c** = 1736.581, **2c** = 73.519

HPLC of (4-fluorophenyl)boronic acid (**3a**) *vs.* [1,1'-biphenyl]-4-ylboronic acid, pinacol ester (**2b**)

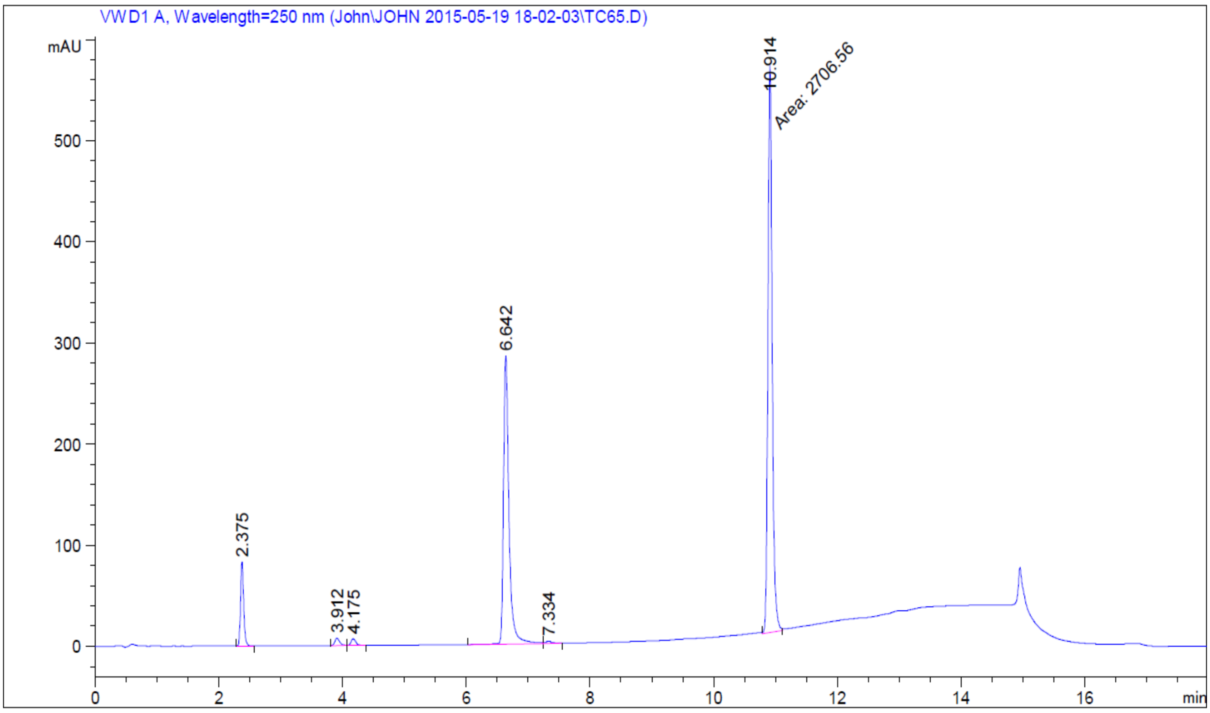

Areas: caf = 295.713, **3c** = 35.525, **2c** = 14.890

HPLC of phenylboronic acid (**4a**) *vs.* [1,1'-biphenyl]-4-ylboronic acid, pinacol ester (**2b**)

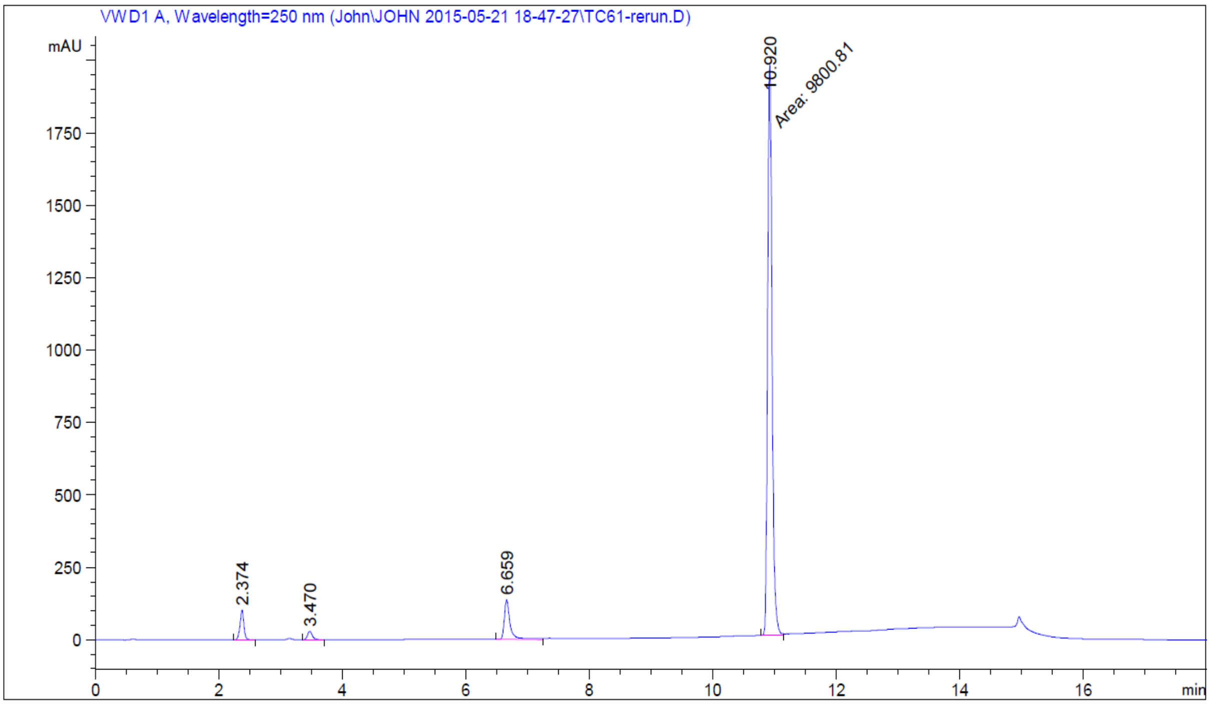

Areas: caf = 434.452, **4c** = 150.785, **2c** = 0

HPLC of (4-methoxyphenyl)boronic acid (**5a**) *vs.* [1,1'-biphenyl]-4-ylboronic acid, pinacol ester (**2b**)

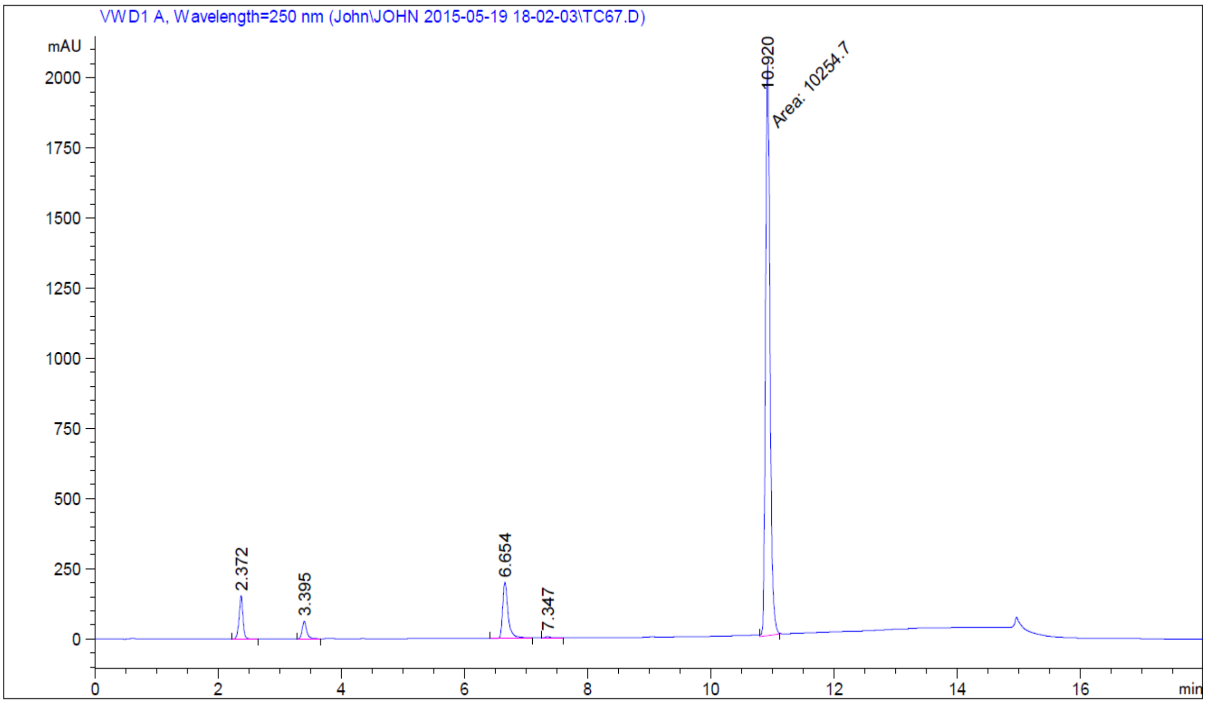

Areas: caf = 651.454, **5c** = 315.976, **2c** = 0

HPLC of (4-acetamidophenyl)boronic acid (**6a**) *vs.* [1,1'-biphenyl]-4-ylboronic acid, pinacol ester (**2b**)

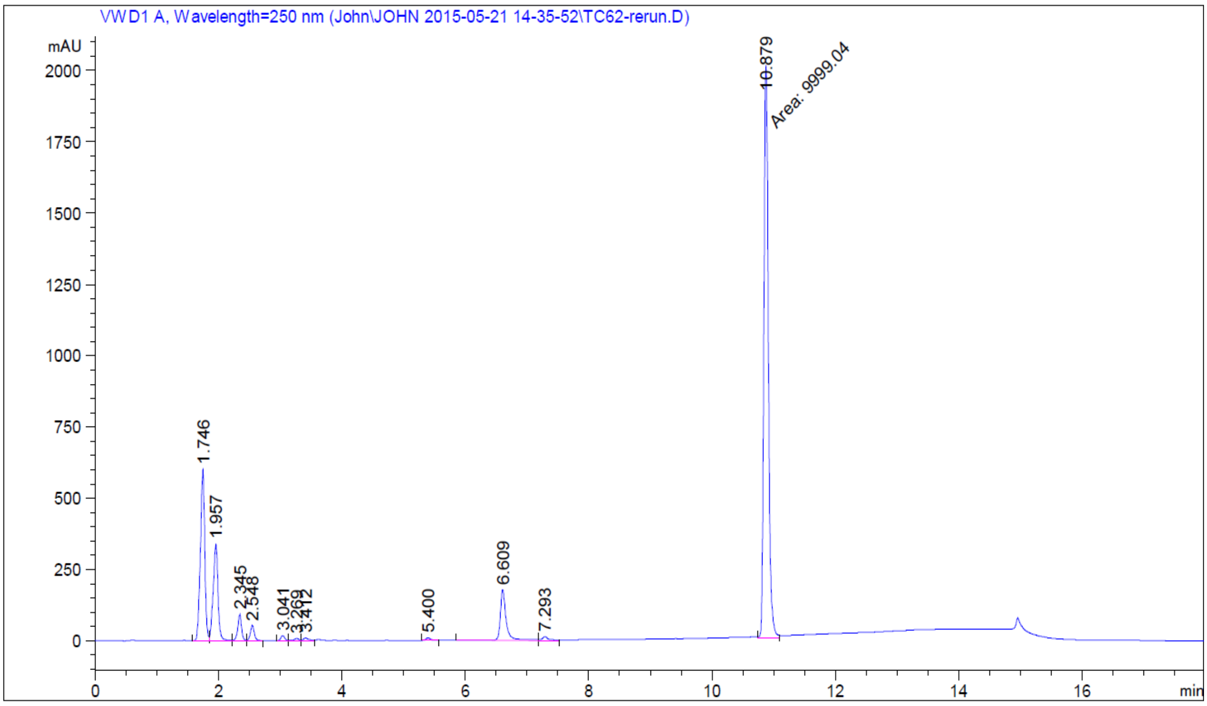

Areas: caf = 394.626, **6c** = 2980.634, **2c** = 94.118

HPLC of (4-(methoxycarbonyl)phenyl)boronic acid (**7a**) *vs.* [1,1'-biphenyl]-4-ylboronic acid, pinacol ester (**2b**)

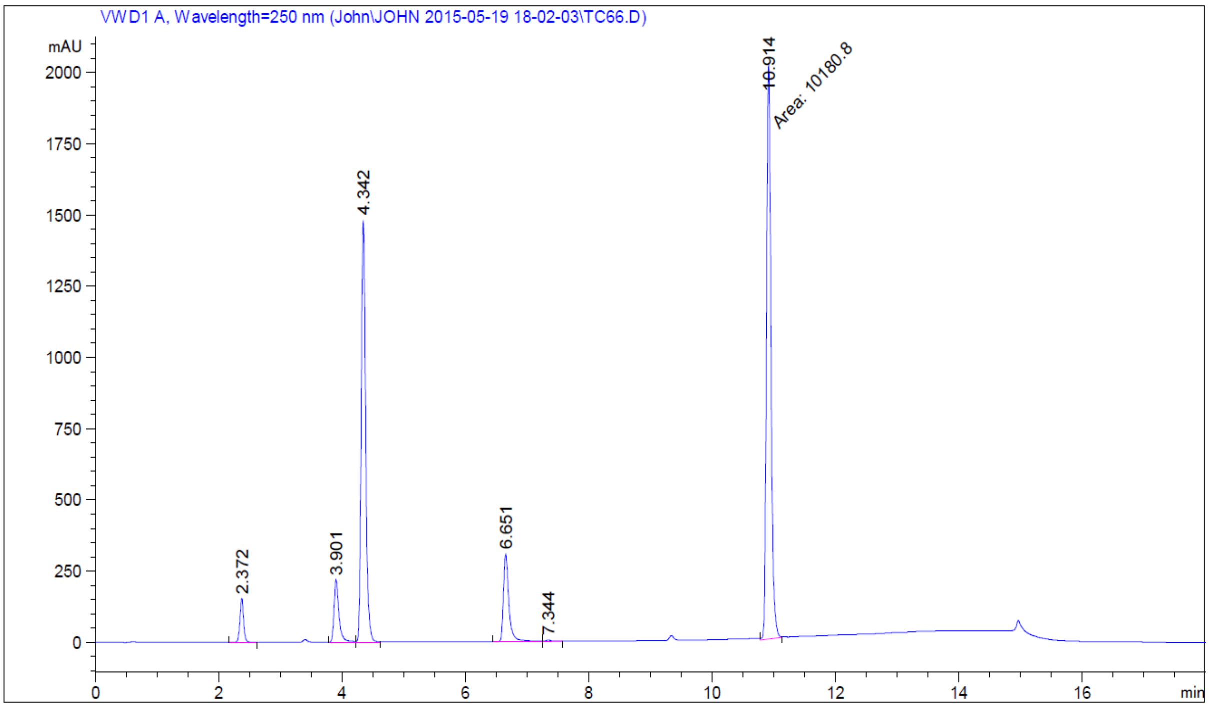

Areas: caf = 616.292, **7c** = 7313.485, **2c** = 29.949

HPLC of (1*H*-Indol-5-yl)boronic acid (**8a**) *vs.* [1,1'-biphenyl]-4-ylboronic acid, pinacol ester (**2b**)

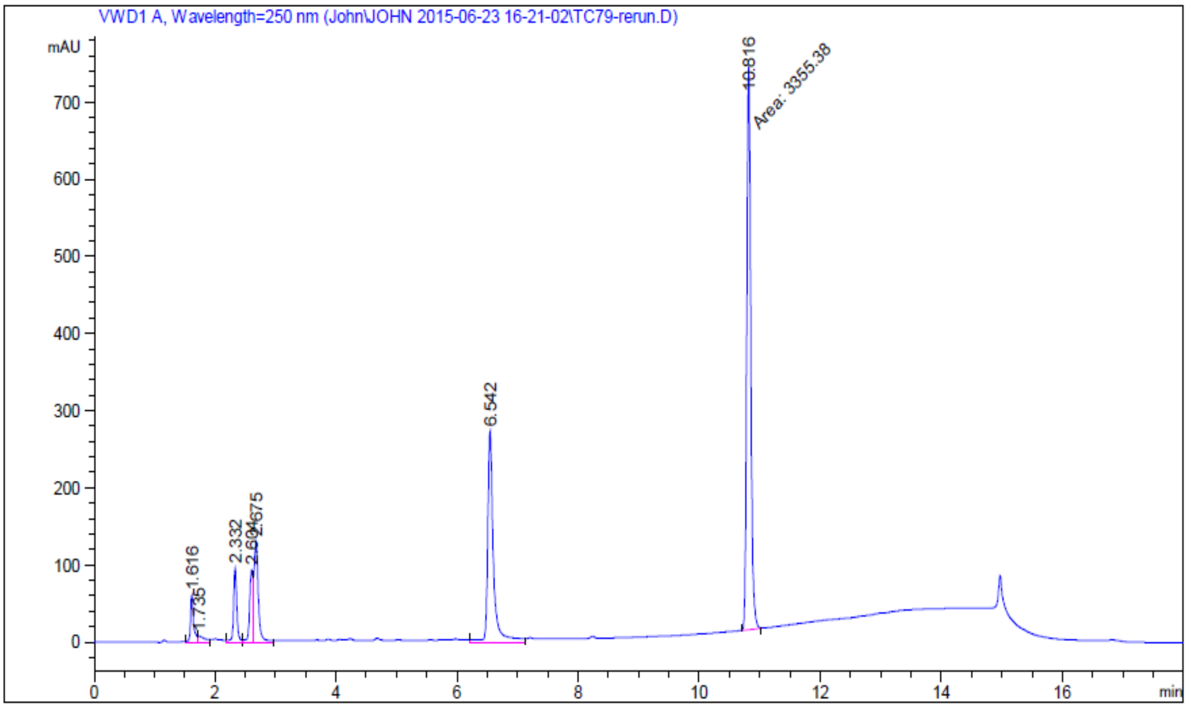

Areas: caf = 357.753, **8c** = 652.601, **2c** = 0

HPLC of 4-methylphenylboronic acid (**9a**) *vs.* [1,1'-biphenyl]-4-ylboronic acid, pinacol ester (**2b**)

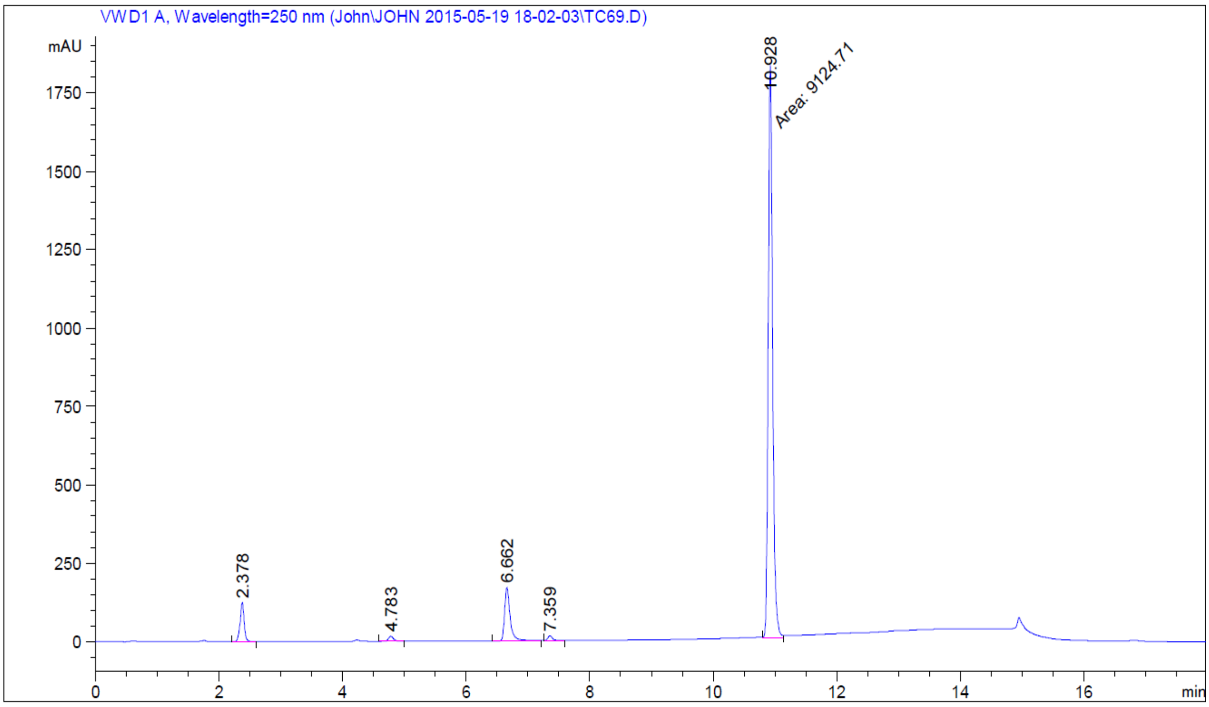

Areas: caf = 558.164, **9c** = 87.264, **2c** = 114.759

HPLC of (2-nitrophenyl)boronic acid (**10a**) *vs.* [1,1'-biphenyl]-4-ylboronic acid, pinacol ester (**2b**)

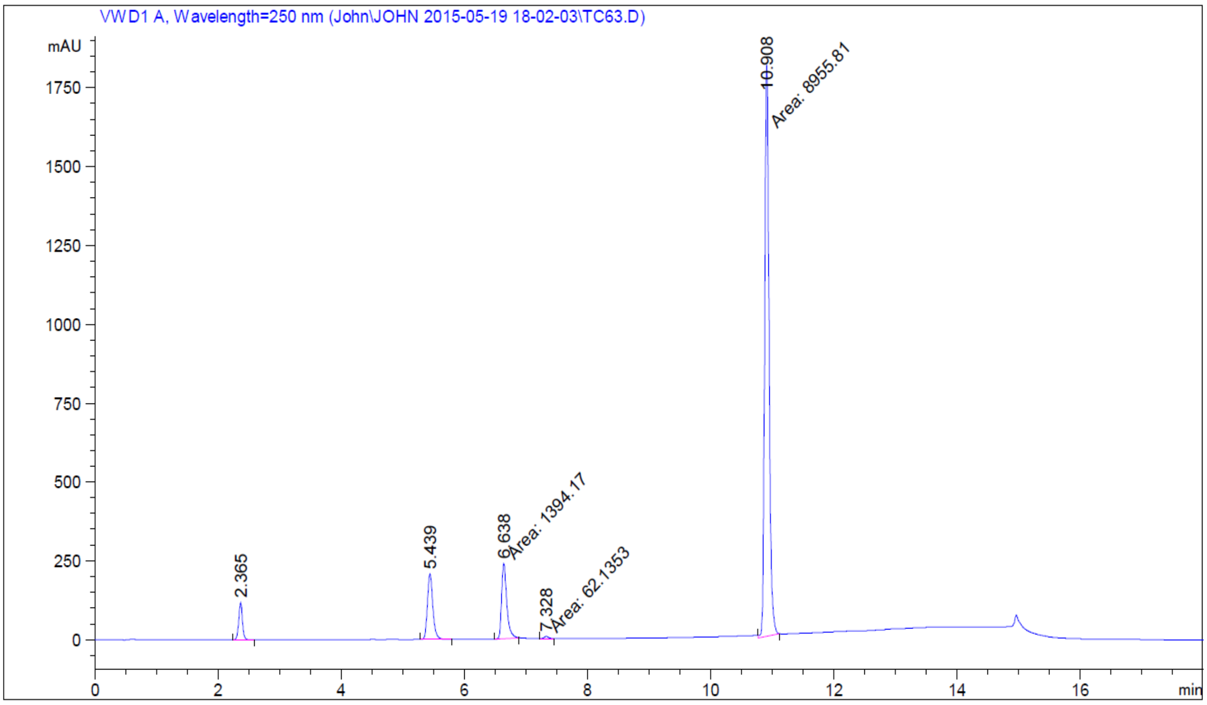

Areas: caf = 481.835, **10c** = 1262.660, **2c** = 62.135

HPLC of mesitylboronic acid (**11a**) *vs.* [1,1'-biphenyl]-4-ylboronic acid, pinacol ester (**2b**)

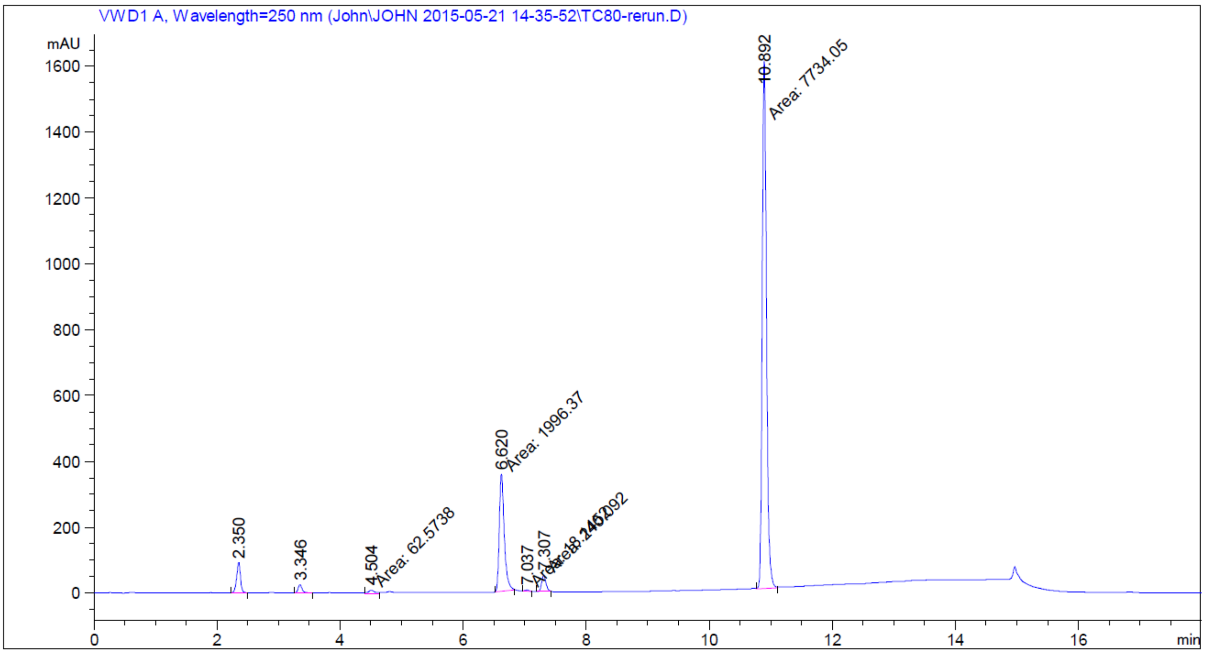

Areas: caf = 396.247, **11c** = 39.563, **2c** = 240.191

HPLC of (3-bromophenyl)boronic acid (**12a**) *vs.* [1,1'-biphenyl]-4-ylboronic acid, pinacol ester (**2b**)

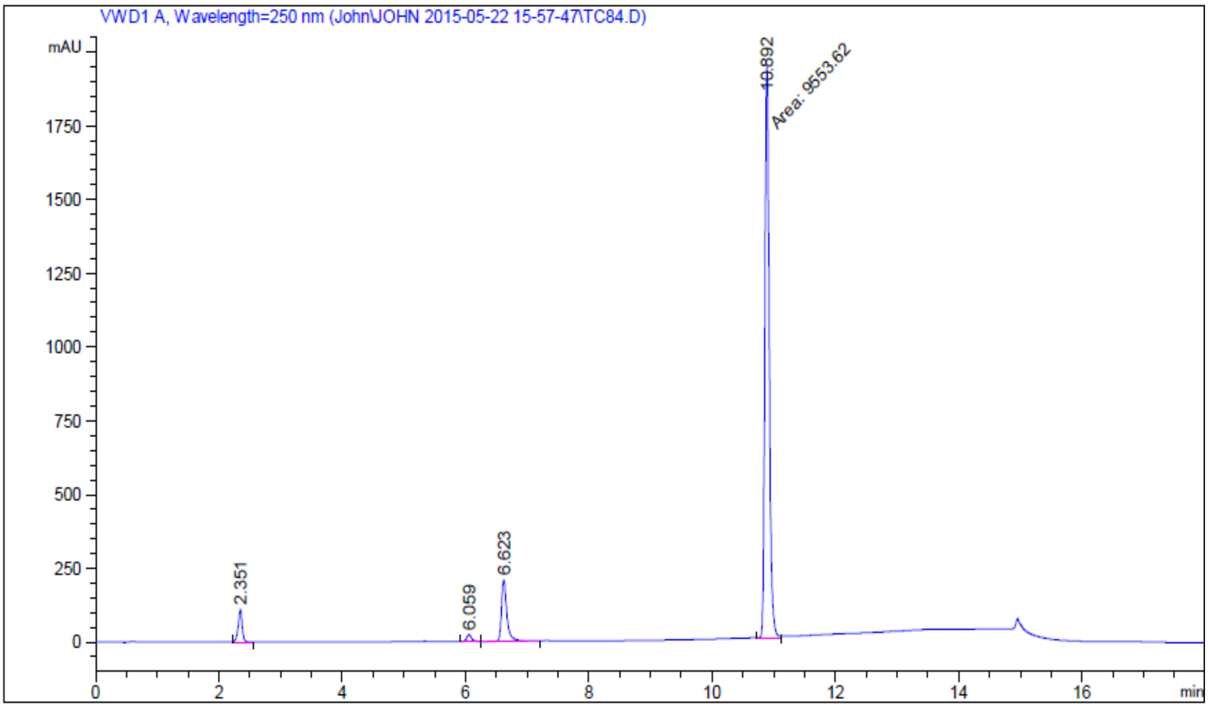

Areas: caf = 488.704, **12c** = 124.412, **2c** = 0

HPLC of benzofuran-5-ylboronic acid (**13a**) *vs.* [1,1'-biphenyl]-4-ylboronic acid, pinacol ester (**2b**)

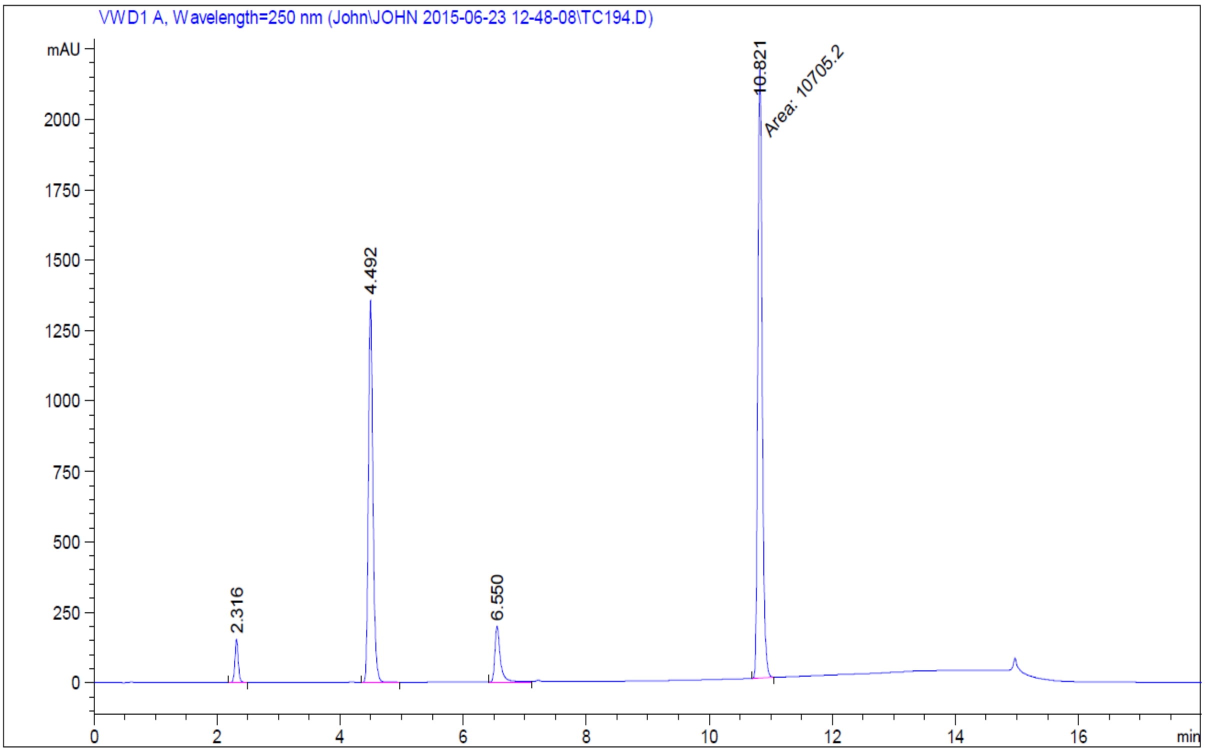

Areas: caf = 581.486, **13c** = 6954.394, **2c** = 0

HPLC of (2-methoxypyridin-3-yl)boronic acid (**14a**) *vs.* [1,1'-biphenyl]-4-ylboronic acid, pinacol ester (**2b**)

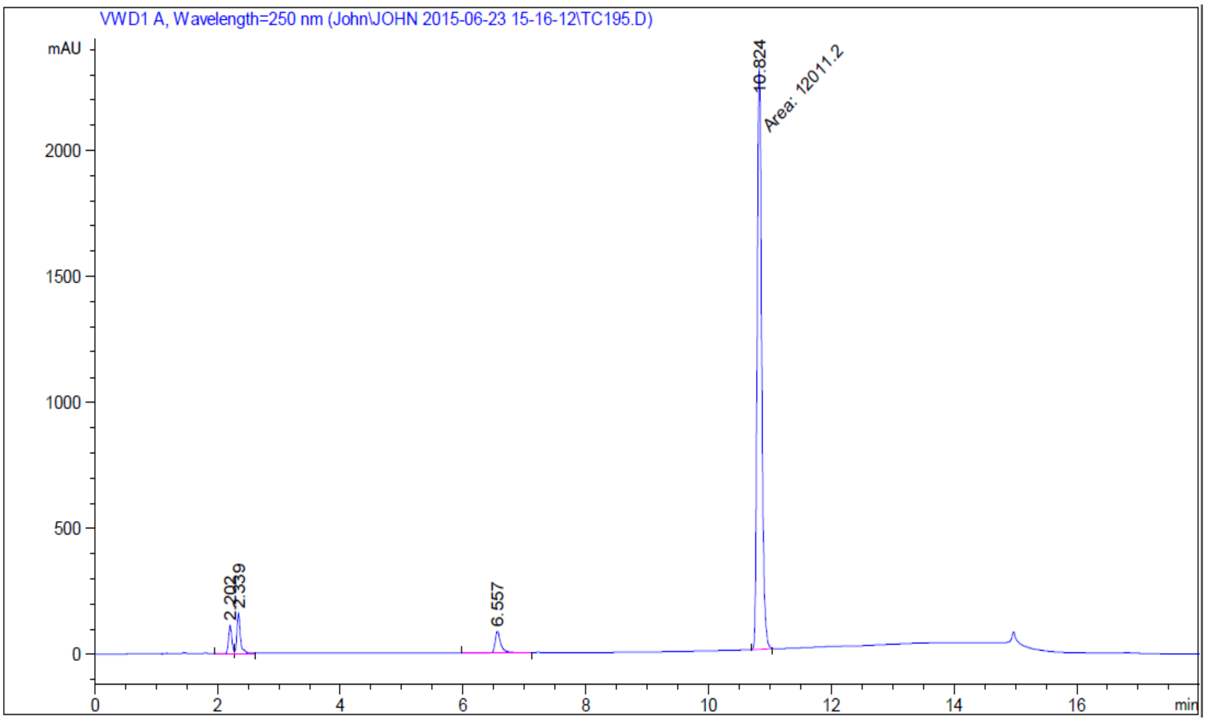

Areas: caf = 628.376, **14c** = 437.232, **2c** = 0

HPLC of naphthalen-2-ylboronic acid (**1a**) *vs.* (4-fluorophenyl)boronic acid, pinacol ester (**3b**)

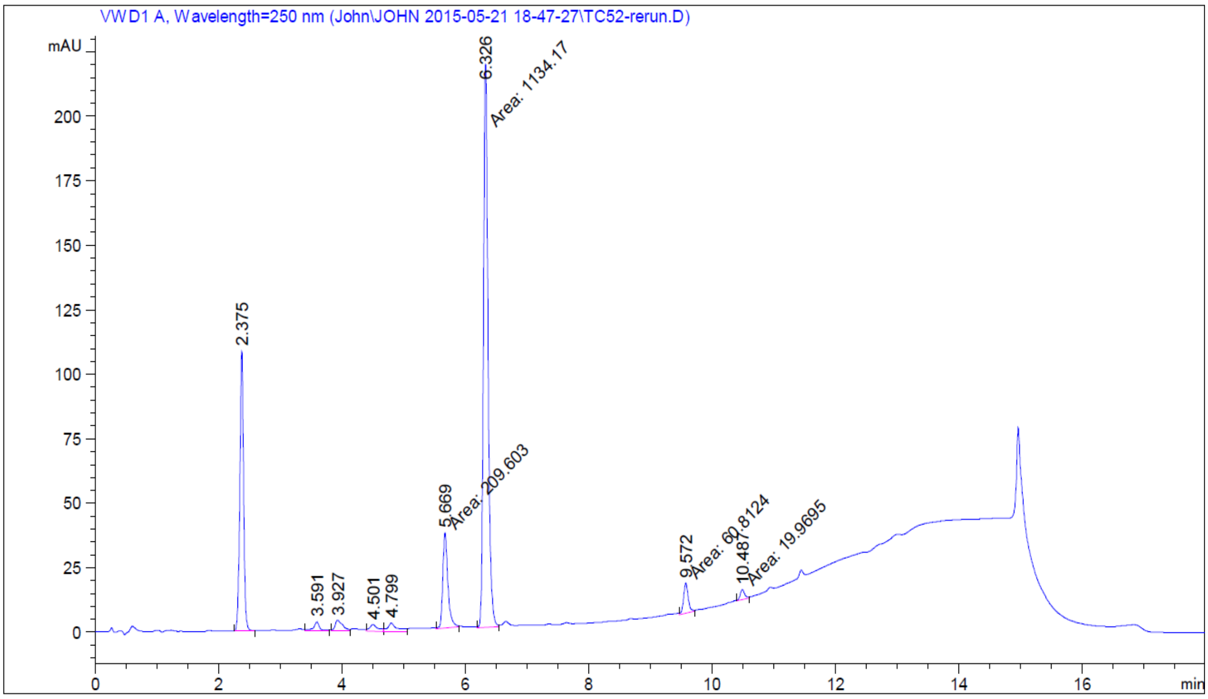

Areas: caf = 456.554, **1c** = 1134.174, **3c** = 0

HPLC of naphthalen-2-ylboronic acid (**1a**) *vs.* phenylboronic acid, pinacol ester (**4b**)

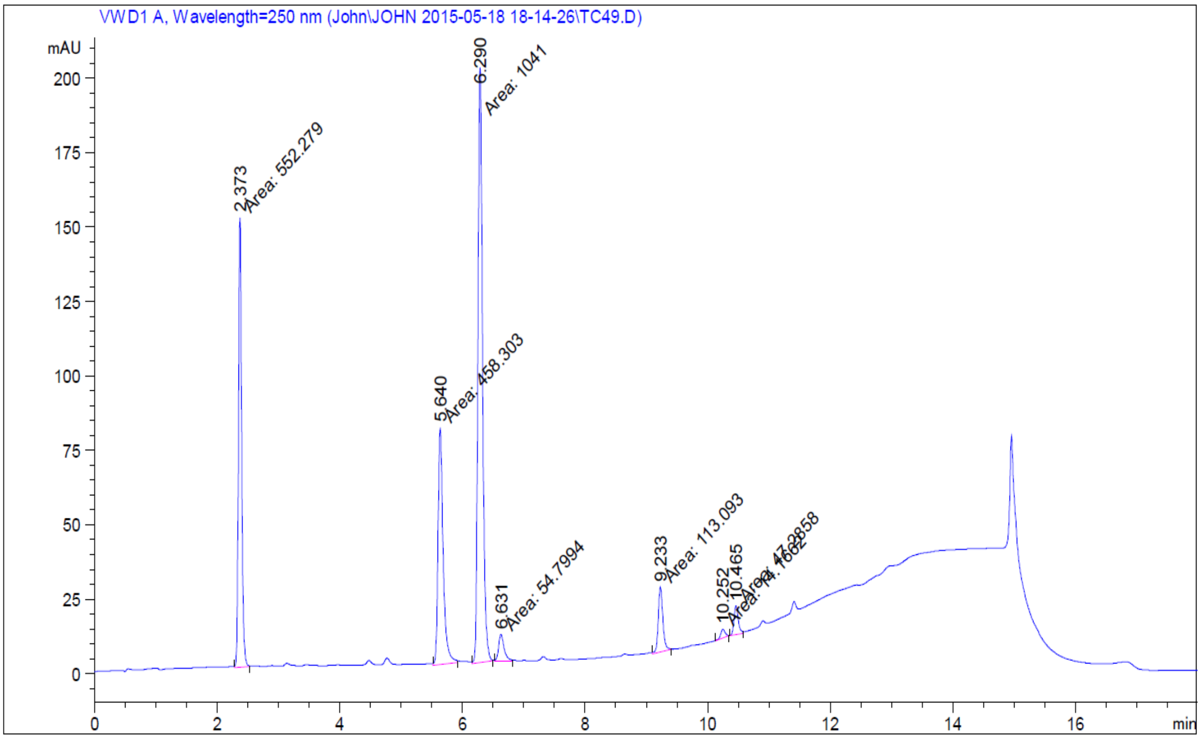

Areas: caf = 552.279, **1c** = 1041.000, **4c** = 0

HPLC of naphthalen-2-ylboronic acid (**1a**) *vs.* (4-methoxyphenyl)boronic acid, pinacol ester (**5b**)

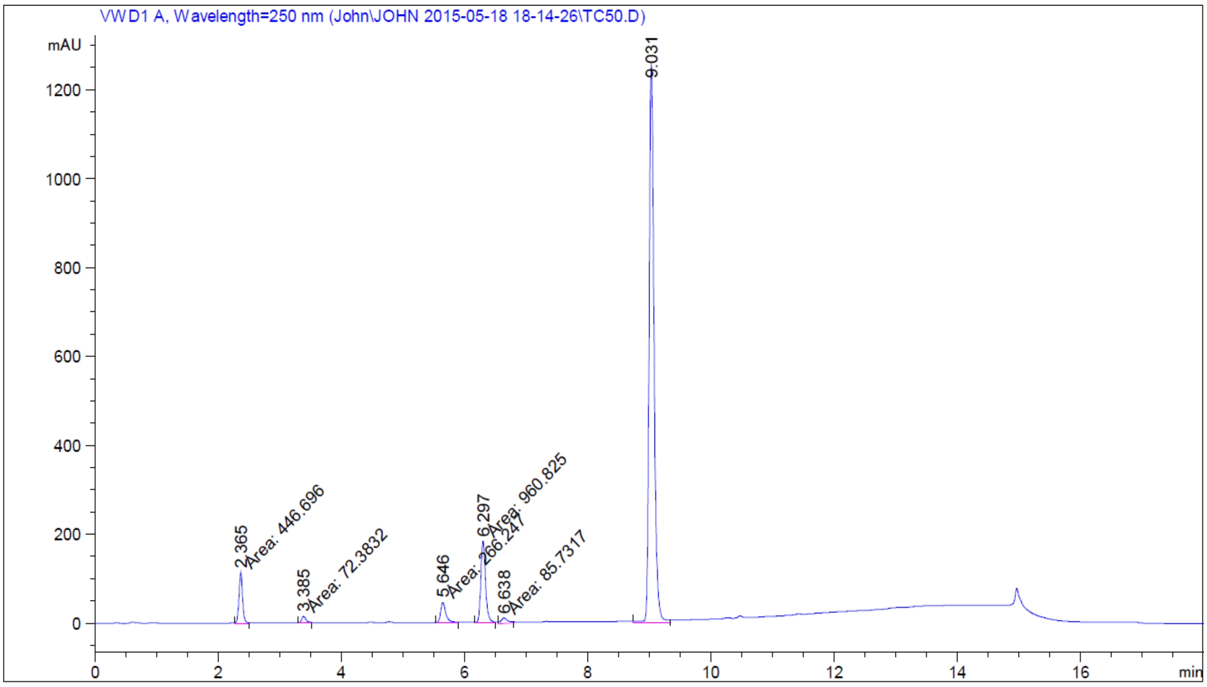

Areas: caf = 446.695, **1c** = 960.825, **5c** = 0

HPLC of naphthalen-2-ylboronic acid (**1a**) *vs.* (4-acetamidophenyl)boronic acid, pinacol ester (**6b**)

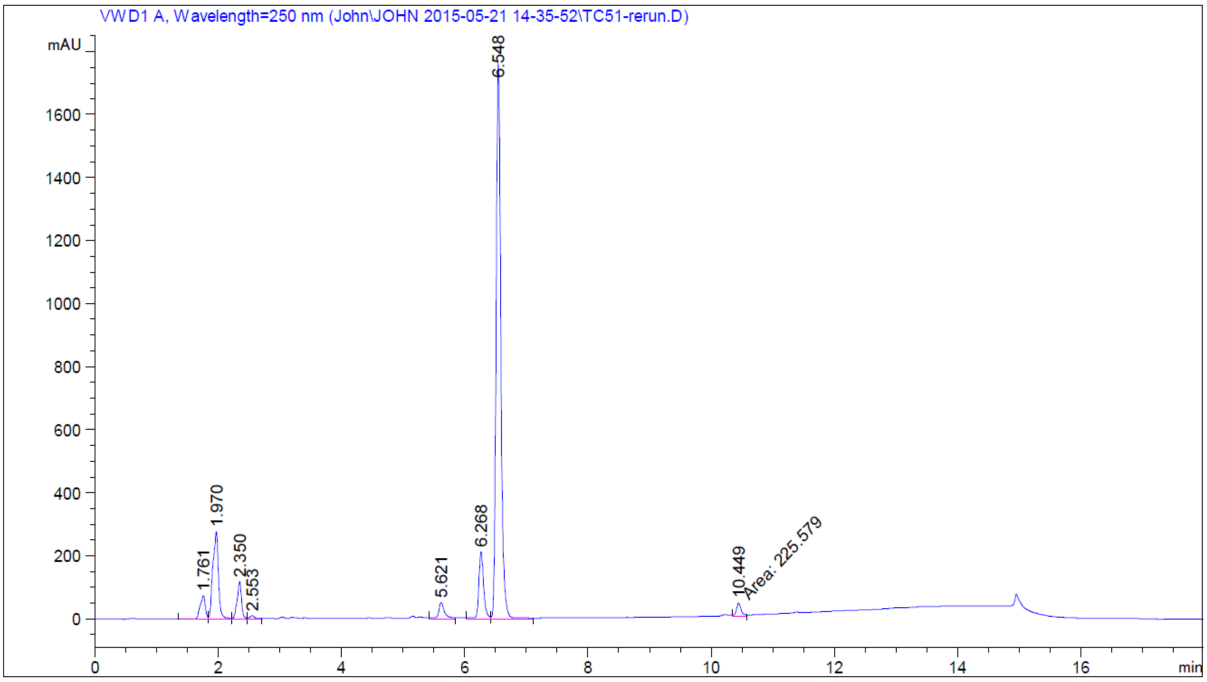

Areas: caf = 602.054, **1c** = 1184.284, **6c** = 454.972

HPLC of naphthalen-2-ylboronic acid (**1a**) *vs.* (4-(methoxycarbonylphenyl)boronic acid, pinacol ester (**7b**)

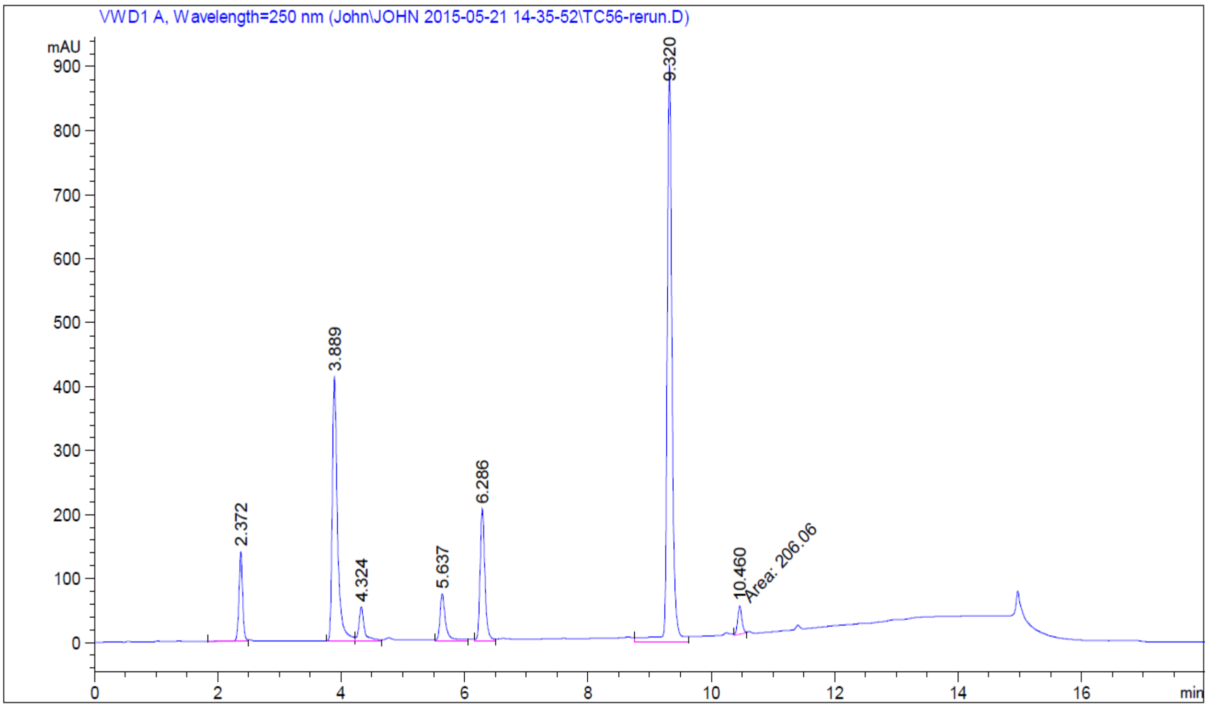

Areas: caf = 571.321, **1c** = 1118.205, **7c** = 333.154

HPLC of naphthalen-2-ylboronic acid (**1a**) *vs.* (1*H*-indol-5-yl)boronic acid, pinacol ester (**8b**)

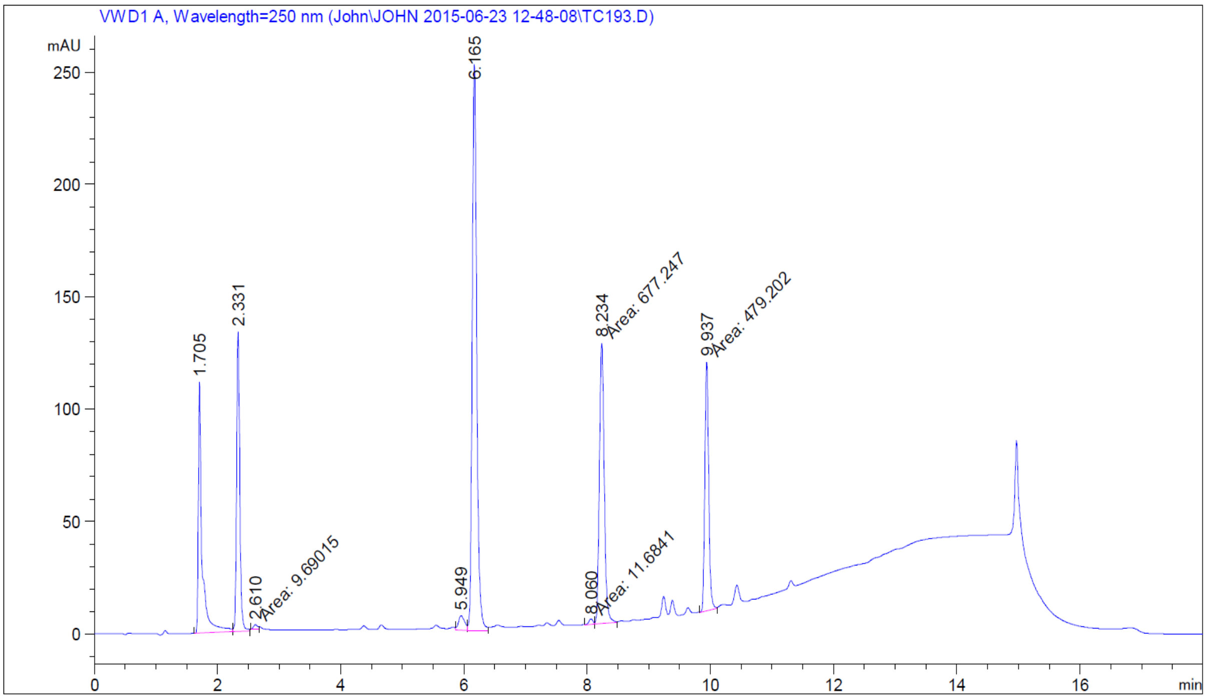

Areas: caf = 478.832, **1c** = 1277.110, **8c** = 9.690

HPLC of naphthalen-2-ylboronic acid (**1a**) *vs.* (2,4-difluorophenyl)boronic acid, pinacol ester (**15b**)

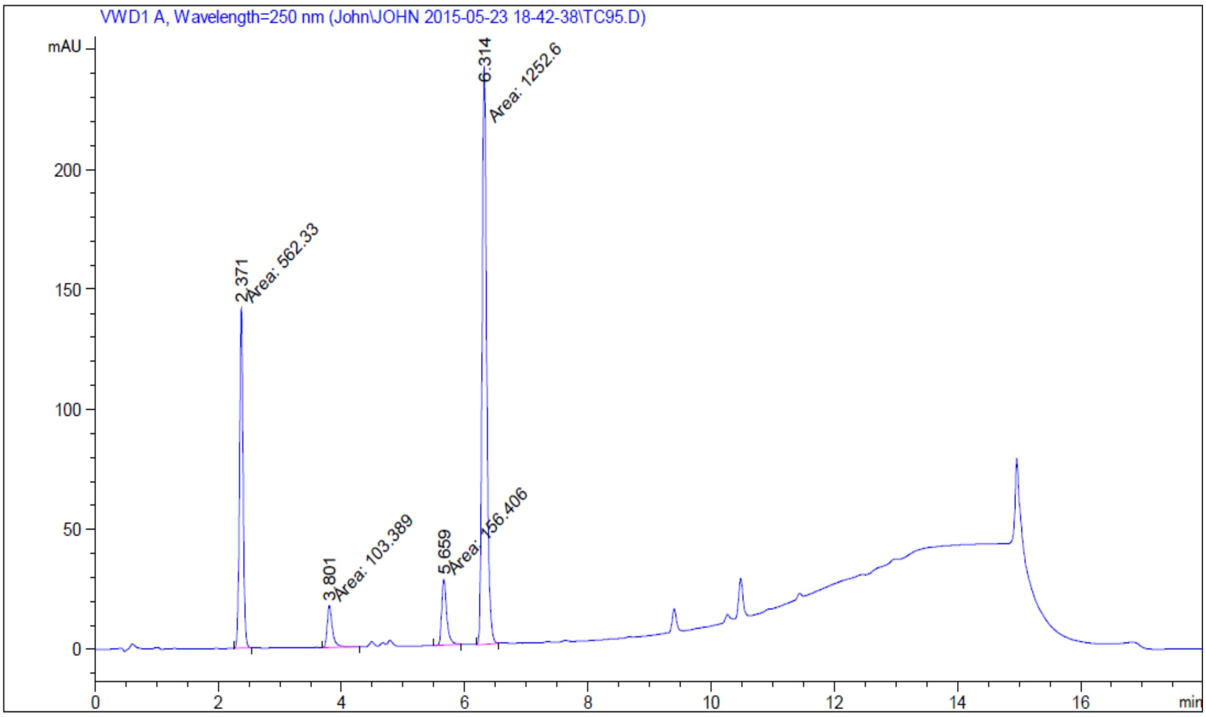

Areas: caf = 562.333, **1c** = 1252.603, **15b** = 103.389

HPLC of naphthalen-2-ylboronic acid (**1a**) *vs.* (4-cyanophenyl)boronic acid, pinacol ester (**16b**)

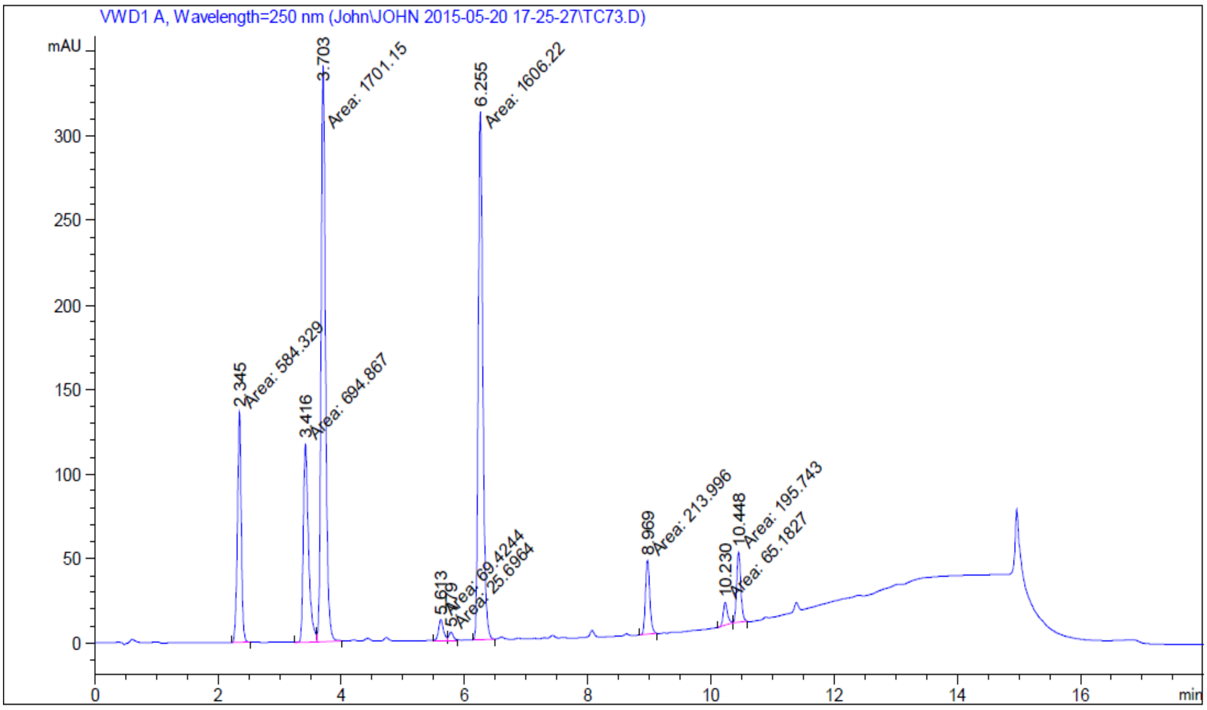

Areas: caf = 584.329, **1c** = 1606.219, **16b** = 694.867

HPLC of naphthalen-2-ylboronic acid (**1a**) *vs.* (benzofuran-2-yl)boronic acid, pinacol ester (**17b**)

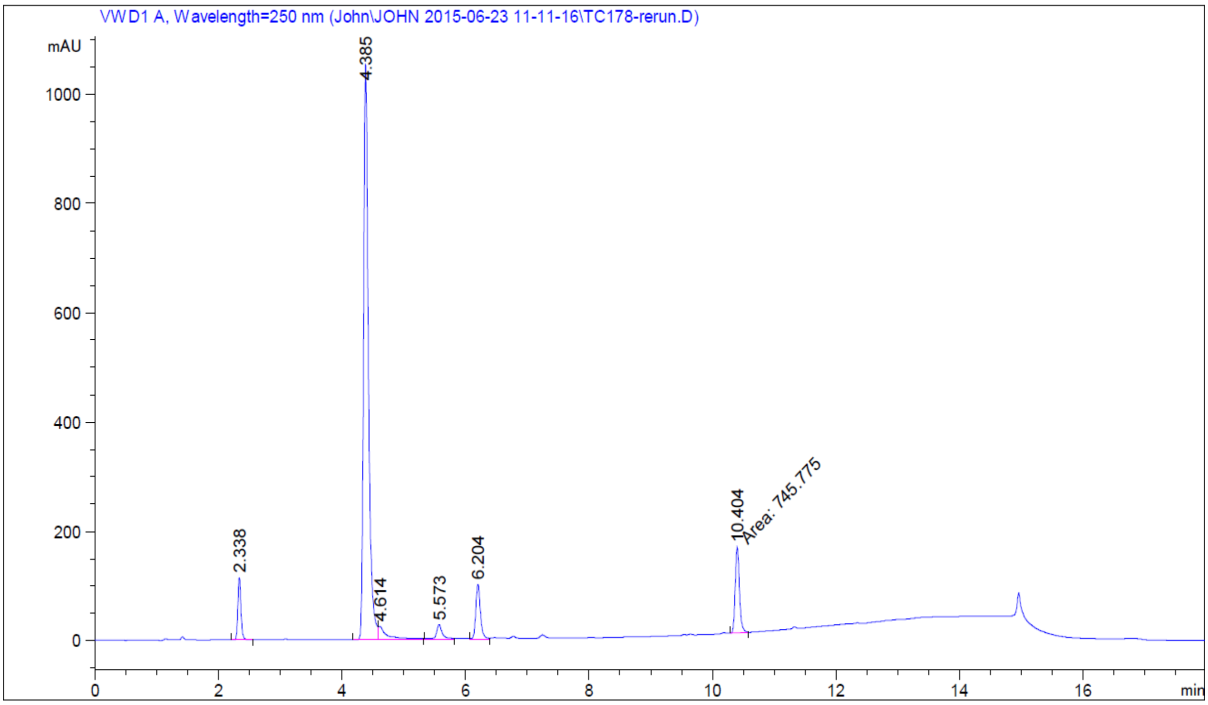

Areas: caf = 399.226, **1c** = 542.455, **17b** = 5781.804

HPLC of naphthalen-2-ylboronic acid (**1a**) *vs.* thiophen-2-ylboronic acid, pinacol ester (**18b**)

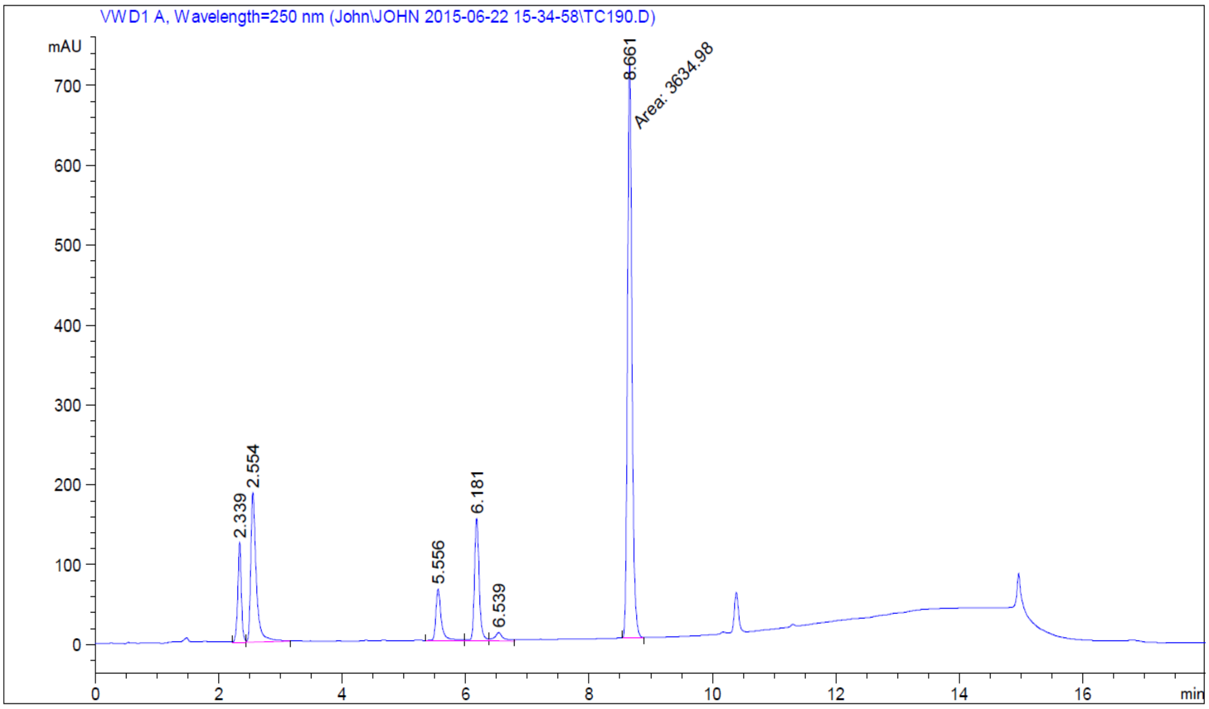

Areas: caf = 506.723, **1c** = 811.394, **18a** = 1160.510, **18b** = 3634.975

HPLC of naphthalen-2-ylboronic acid (**1a**) *vs.* isoquinolin-4-ylboronic acid, pinacol ester (**19b**)

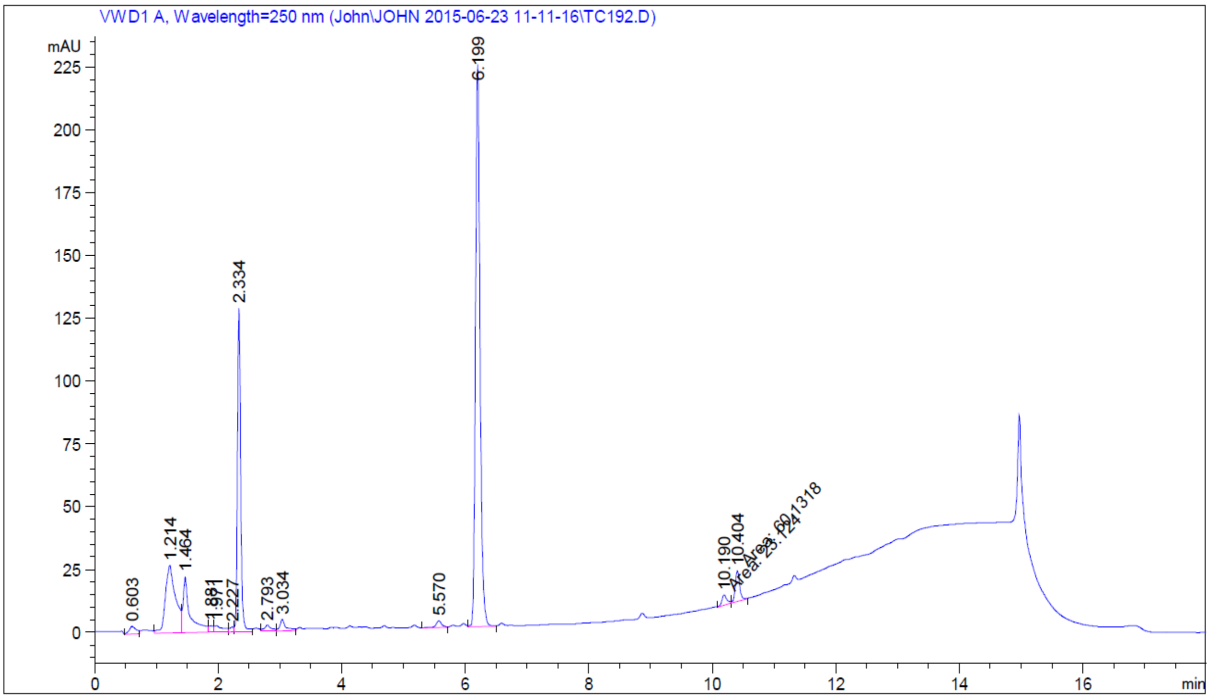

Areas: caf = 469.810, **1c** = 1143.903, **19b** = 311.518

HPLC of naphthalen-2-ylboronic acid (**1a**) *vs.* (2-aminophenyl)boronic acid, pinacol ester (**20b**)

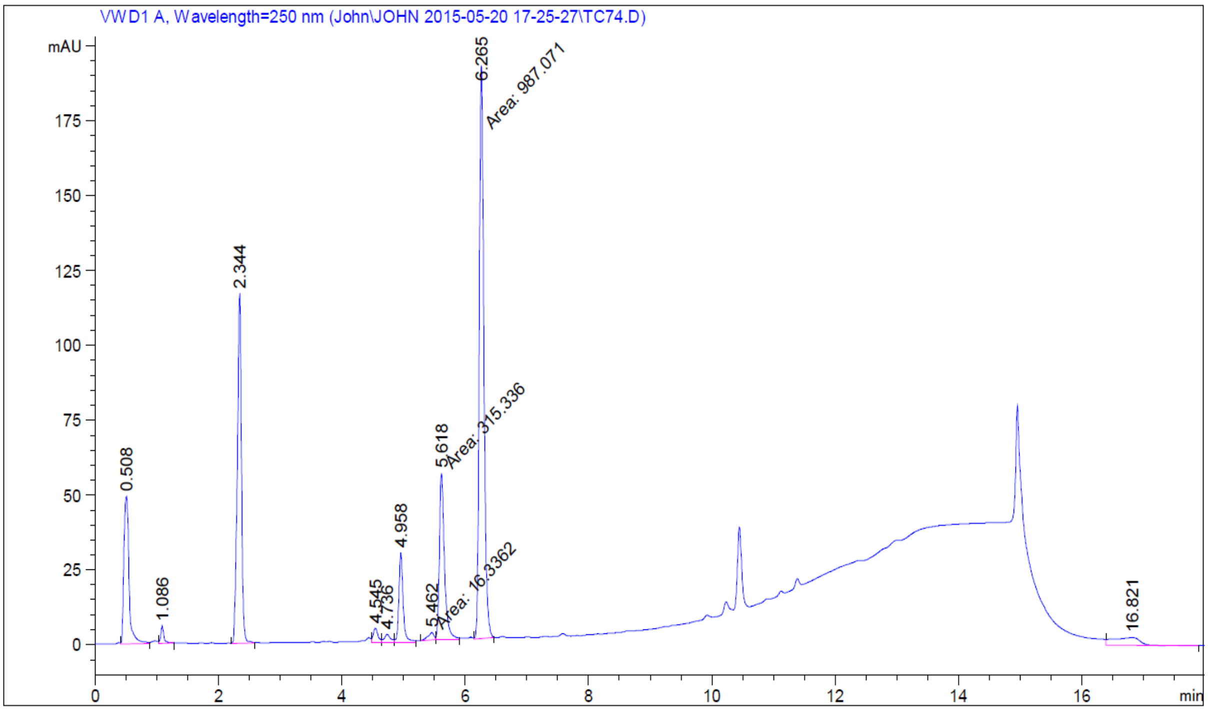

Areas: caf = 546.963, **1c** = 987.071, **20c** = 0

HPLC of naphthalen-2-ylboronic acid, MIDA ester (**1f**) *vs.* phenylboronic acid, pinacol ester (**4b**)

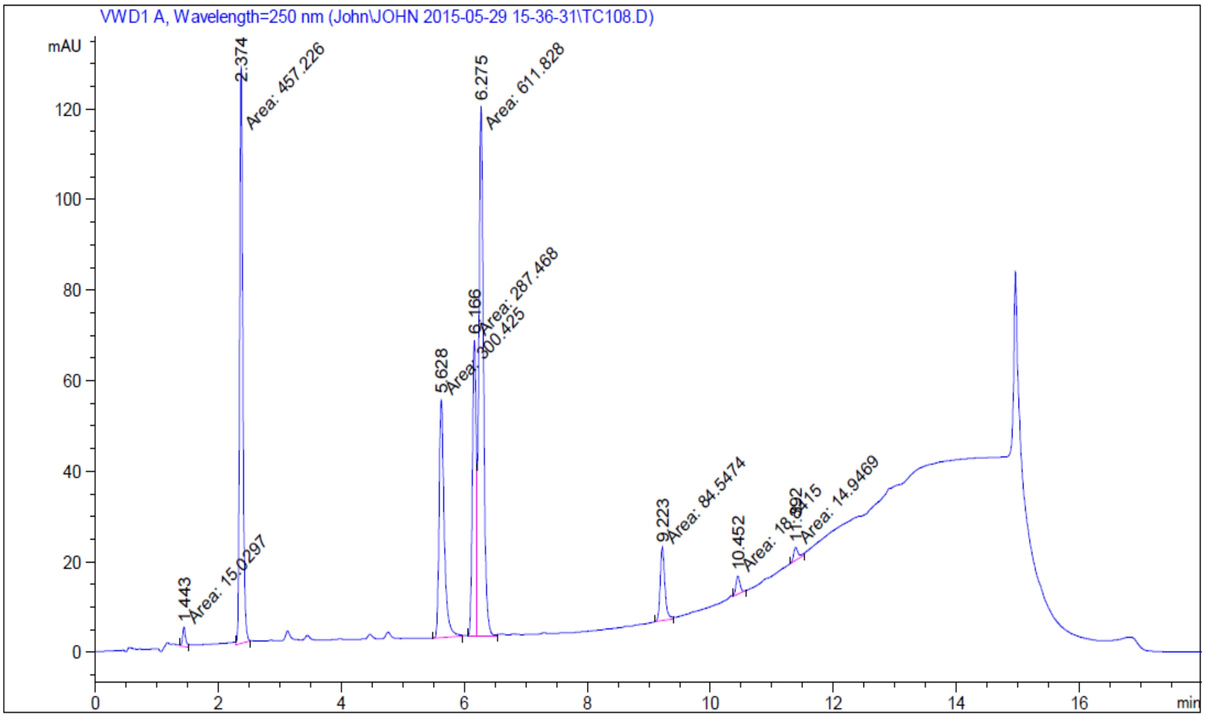

Areas: caf = 457.226, **1c** = 611.828, **4c** = 0

HPLC of naphthalen-2-ylboronic acid, MIDA ester (**1f**) *vs.* (4-methoxyphenyl)boronic acid, pinacol ester (**5b**)

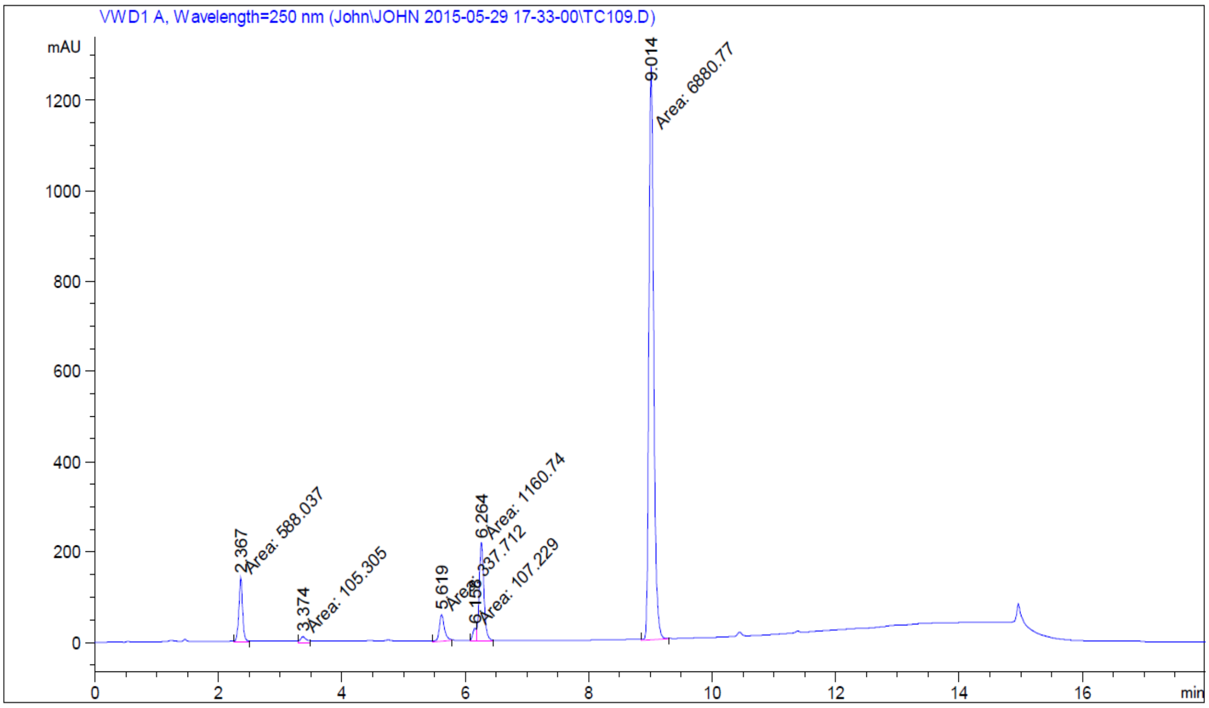

Areas: caf = 588.037, **1c** = 1160.740, **5c** = 0

HPLC of naphthalen-2-ylboronic acid, MIDA ester (**1f**) *vs.* (4-(methoxycarbonyl)phenyl)boronic acid, pinacol ester (**7b**)

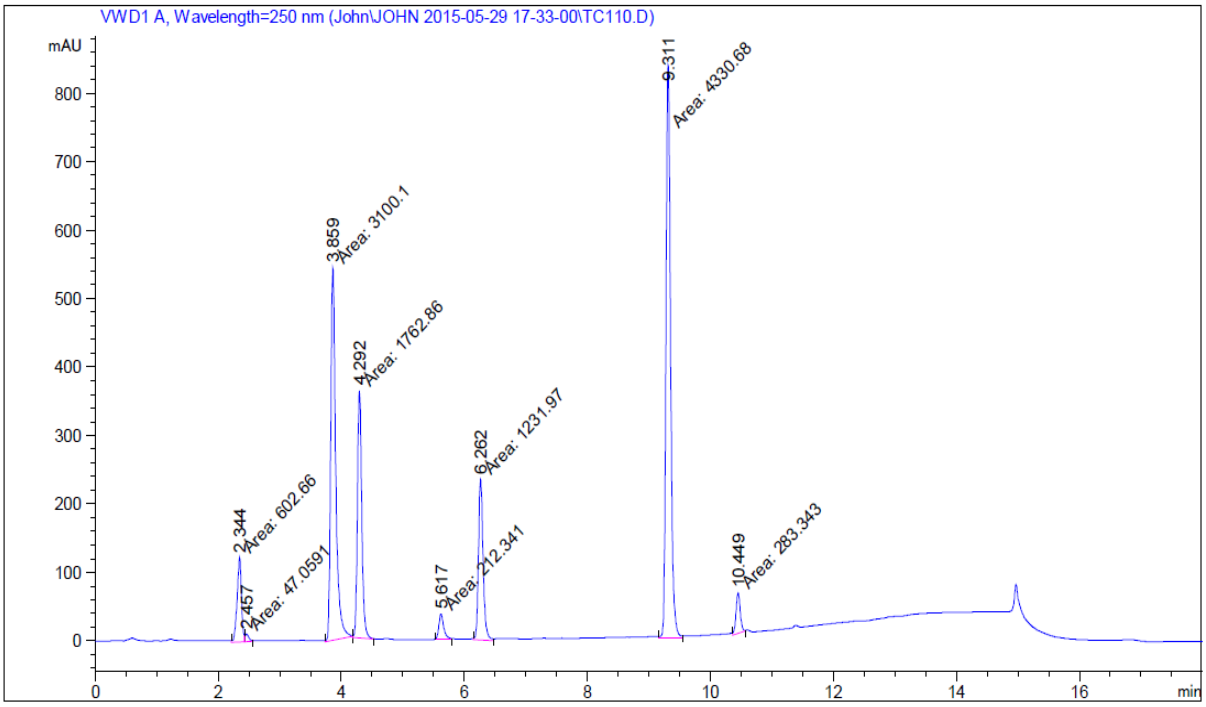

Areas: caf = 602.660, **1c** = 1231.969, **7c** = 1762.860

HPLC of naphthalen-2-ylboronic acid, MIDA ester (**1f**) *vs.* thiophen-2-ylboronic acid, pinacol ester (**18b**)

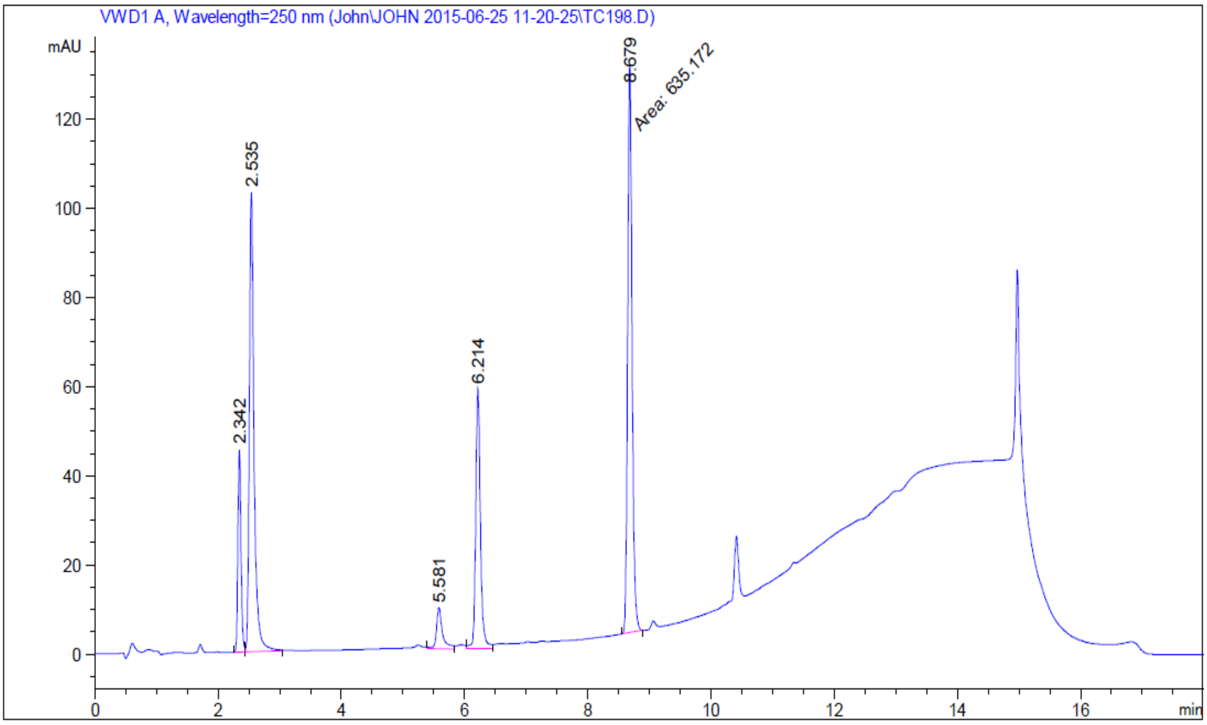

Areas: caf = 157.743, **1c** = 313.512, **18a** = 548.585, **18b** = 635.172

HPLC of naphthalen-2-ylboronic acid, MIDA ester (**1f**) *vs.* (4-isopropylphenyl)boronic acid, pinacol ester (**21b**)

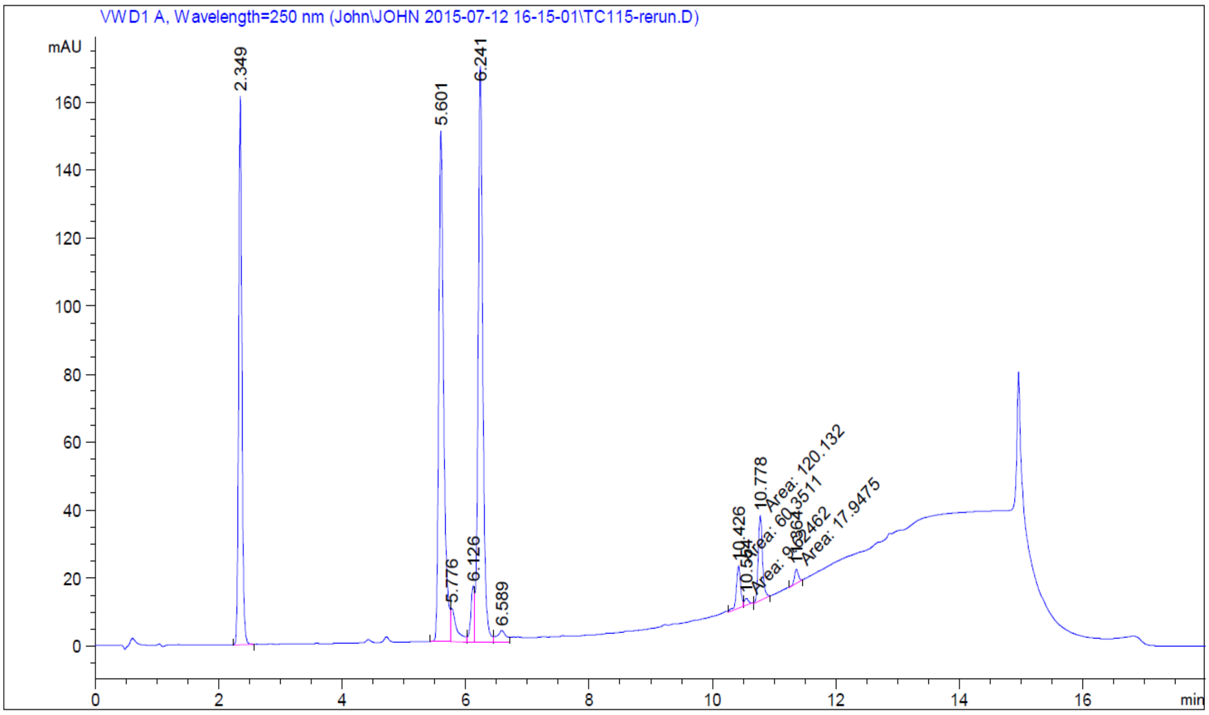

Areas: caf = 598.948, **1c** = 898.749, **21c** = 0

HPLC of naphthalen-2-ylboronic acid, MIDA ester (**1f**) *vs.* (4-hydroxyphenyl)boronic acid, pinacol ester (**22b**)

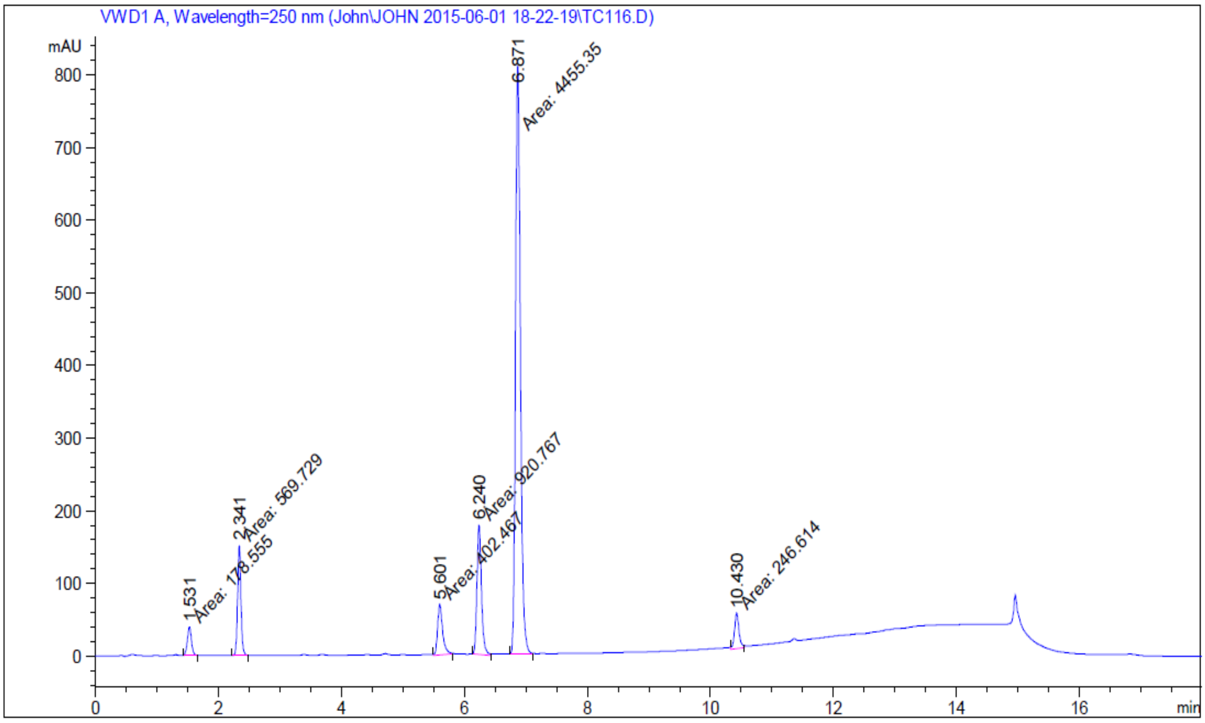

Areas: caf = 569.729, **1c** = 920.767, **22c** = 0

HPLC of naphthalen-2-ylboronic acid, MIDA ester (**1f**) *vs.* (2-chlorophenyl)boronic acid, pinacol ester (**23b**)

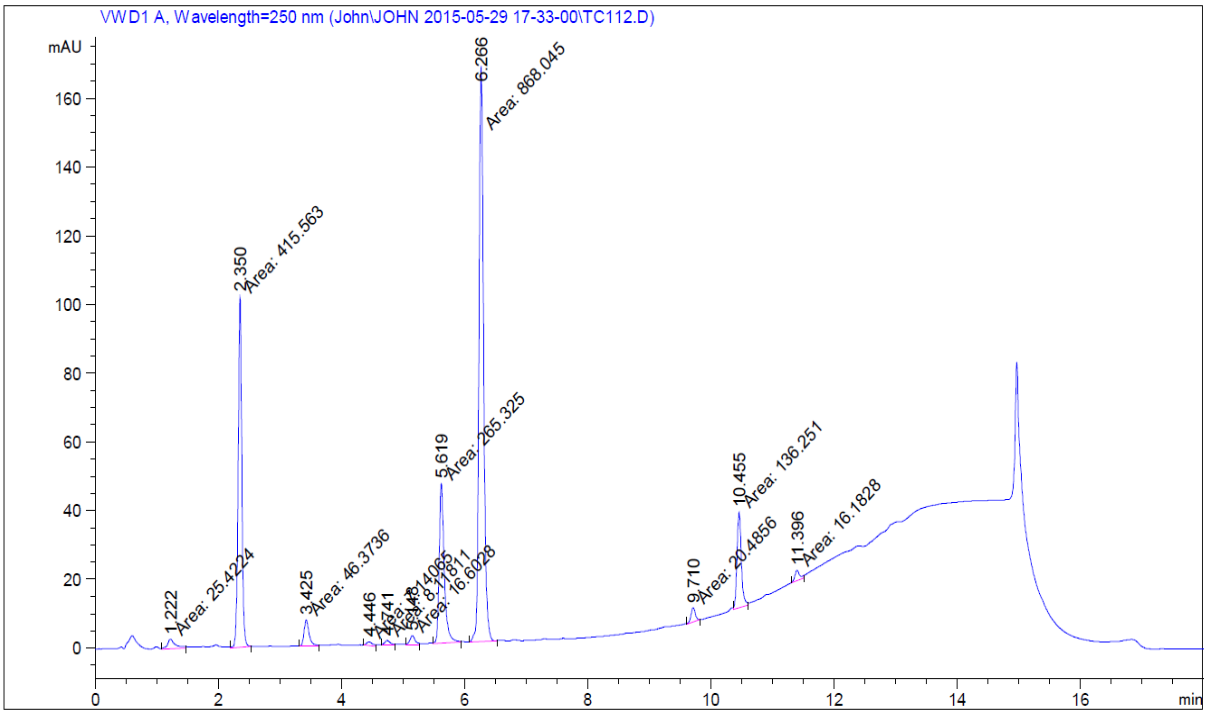

Areas: caf = 415.563, **1c** = 868.045, **23c** = 0

HPLC of naphthalen-2-ylboronic acid, MIDA ester (**1f**) *vs.* (6-methoxypyridin-3-yl)boronic acid, pinacol ester (**24b**)

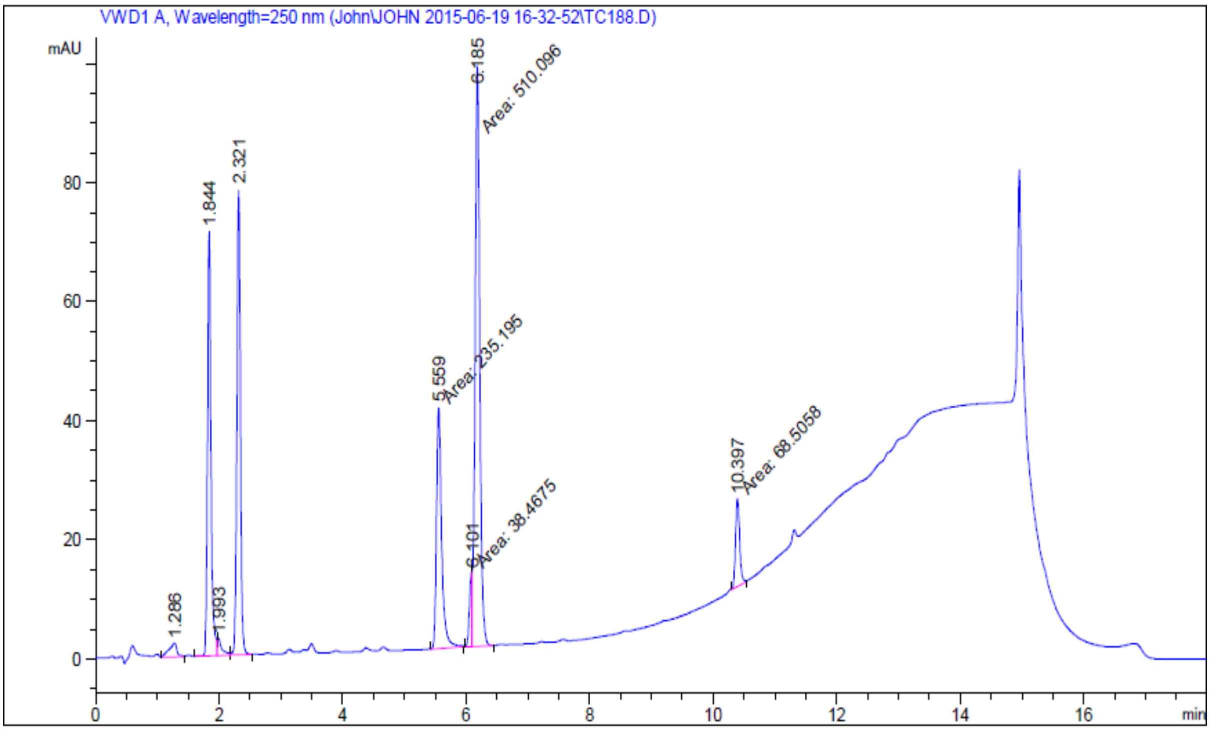

Areas: caf = 306.025, **1c** = 510.096, **24b** = 280.422

HPLC of (4-fluorophenyl)boronic acid, MIDA ester (**3f**) *vs.* [1,1'-biphenyl]-4-ylboronic acid, pinacol ester (**2b**)

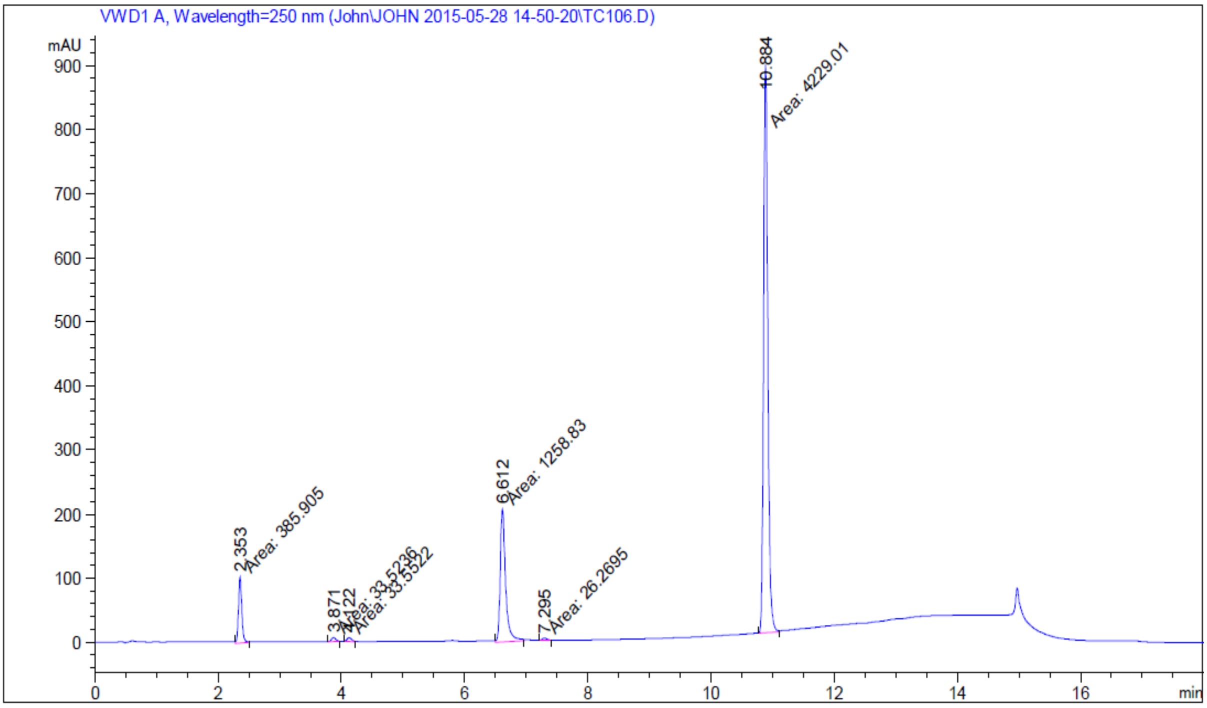

Areas: caf = 385.905, **3c** = 33.552, **2c** = 26.270

HPLC of phenylboronic acid, MIDA ester (**4f**) *vs.* [1,1'-biphenyl]-4-ylboronic acid, pinacol ester (**2b**)

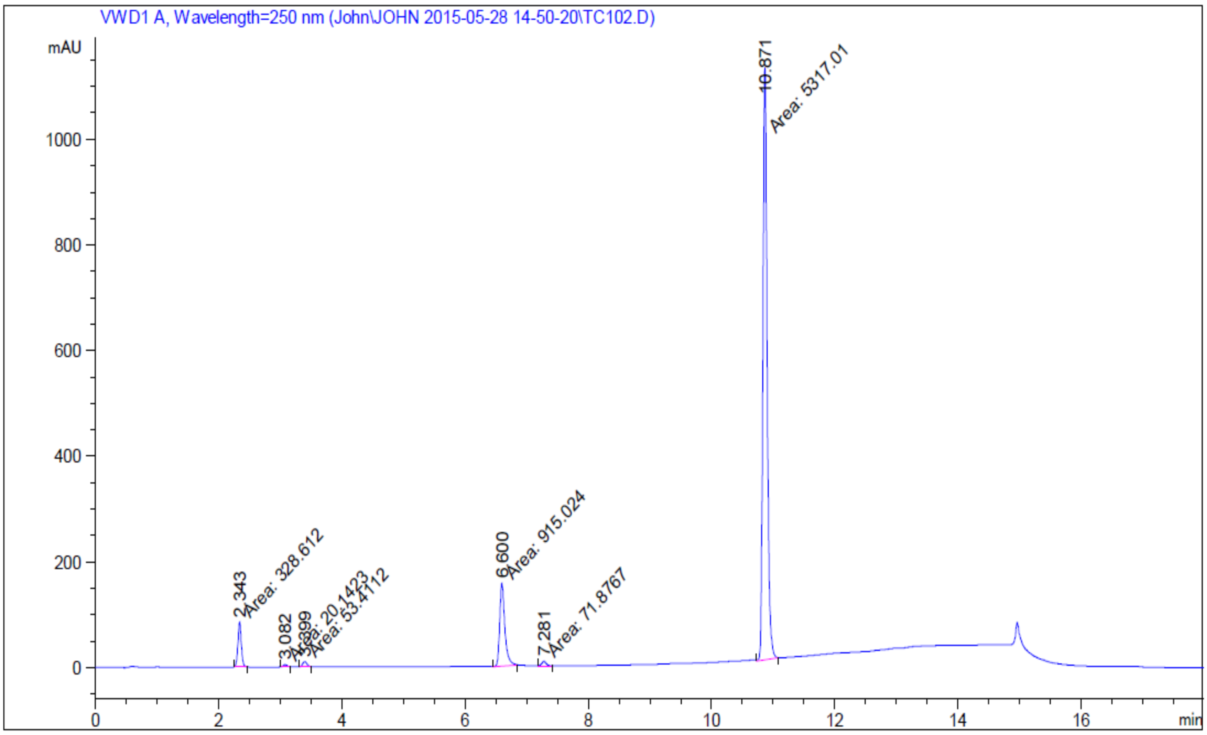

Areas: caf = 328.612, **4c** = 53.411, **2c** = 71.877

HPLC of (1*H*-indol-5-yl)boronic acid, MIDA ester (**8f**) *vs.* [1,1'-biphenyl]-4-ylboronic acid, pinacol ester (**2b**)

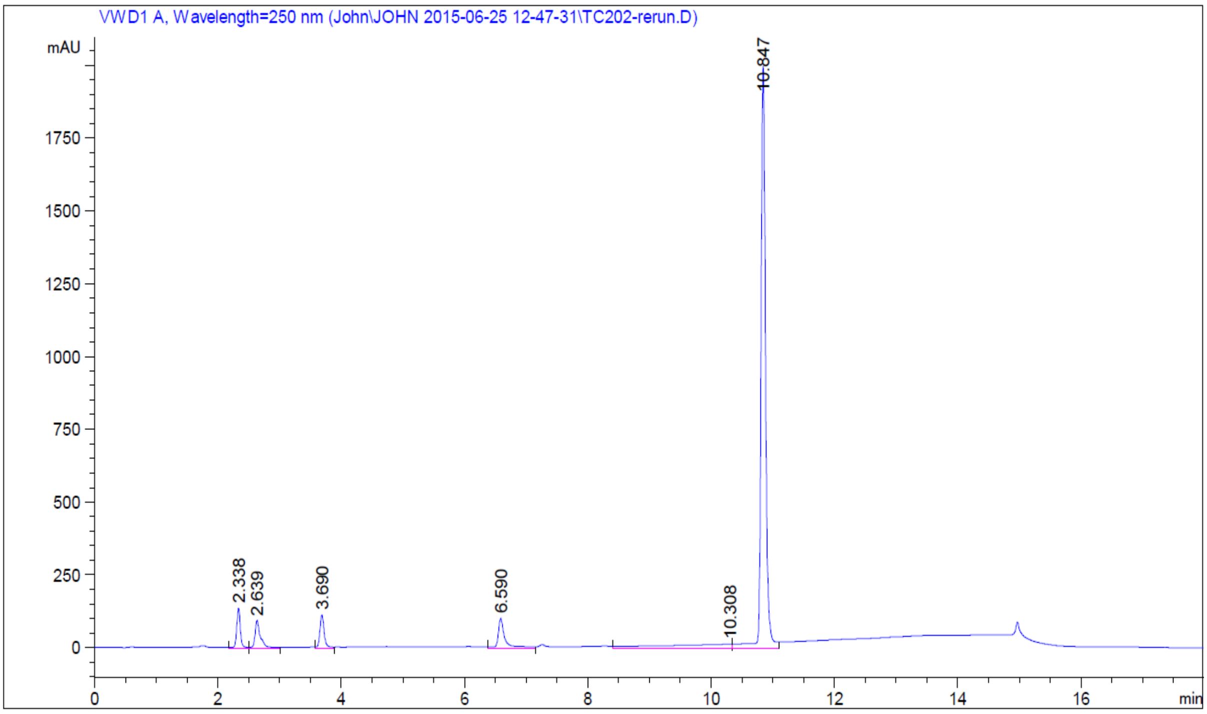

Areas: caf = 549.177, **8c** = 569.511, **2c** = 0

HPLC of *p*-Tolylboronic acid, MIDA ester (**9f**) *vs.* [1,1'-biphenyl]-4-ylboronic acid, pinacol ester (**2b**)

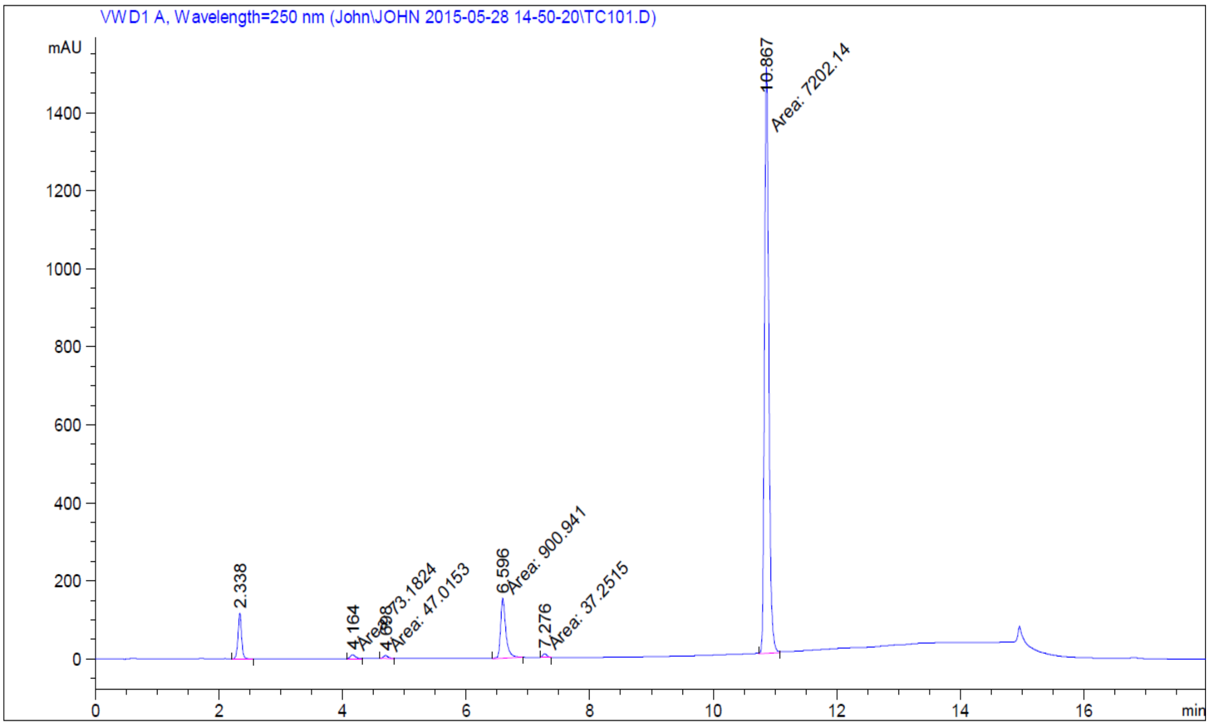

Areas: caf = 464.353, **9c** = 47.015, **2c** = 37.251

HPLC of benzofuran-5-ylboronic acid, MIDA ester (**13f**) *vs.* [1,1'-biphenyl]-4-ylboronic acid, pinacol ester (**2b**)

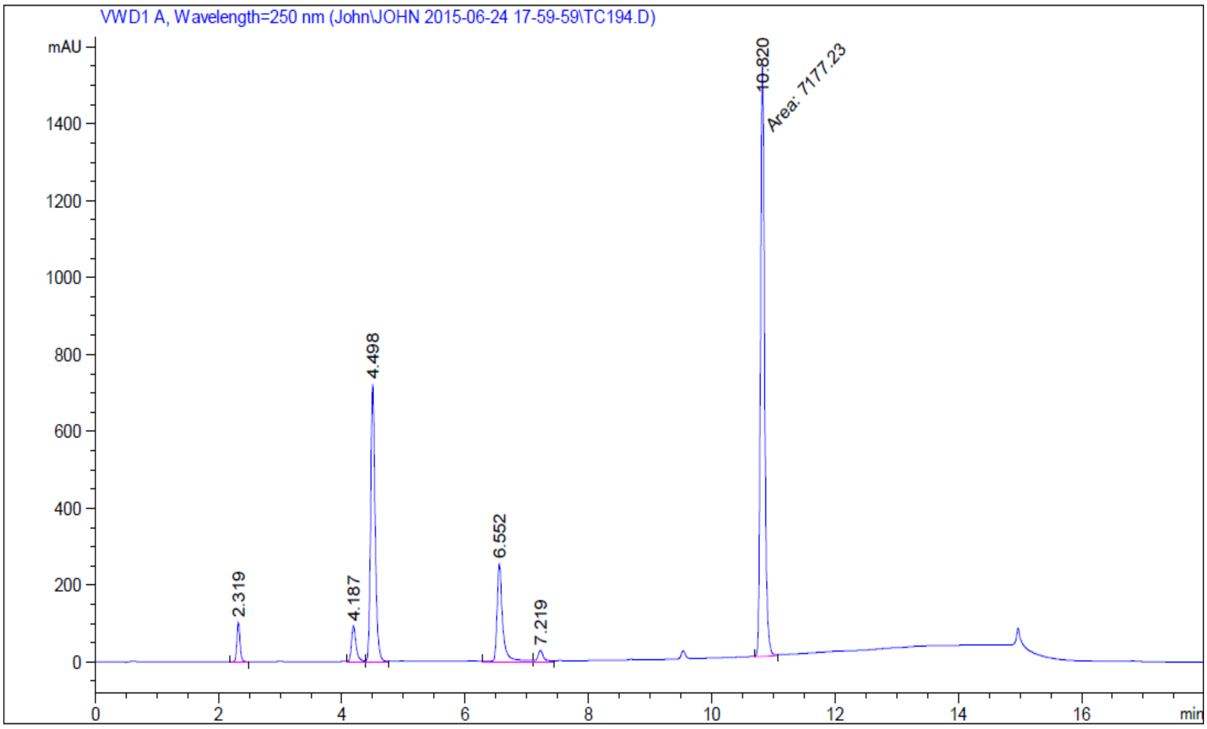

Areas: caf = 363.766, **13c** = 3690.462, **2c** = 197.993

HPLC of (4-hydroxyphenyl)boronic acid, MIDA ester (**22f**) *vs.* [1,1'-biphenyl]-4-ylboronic acid, pinacol ester (**2b**)

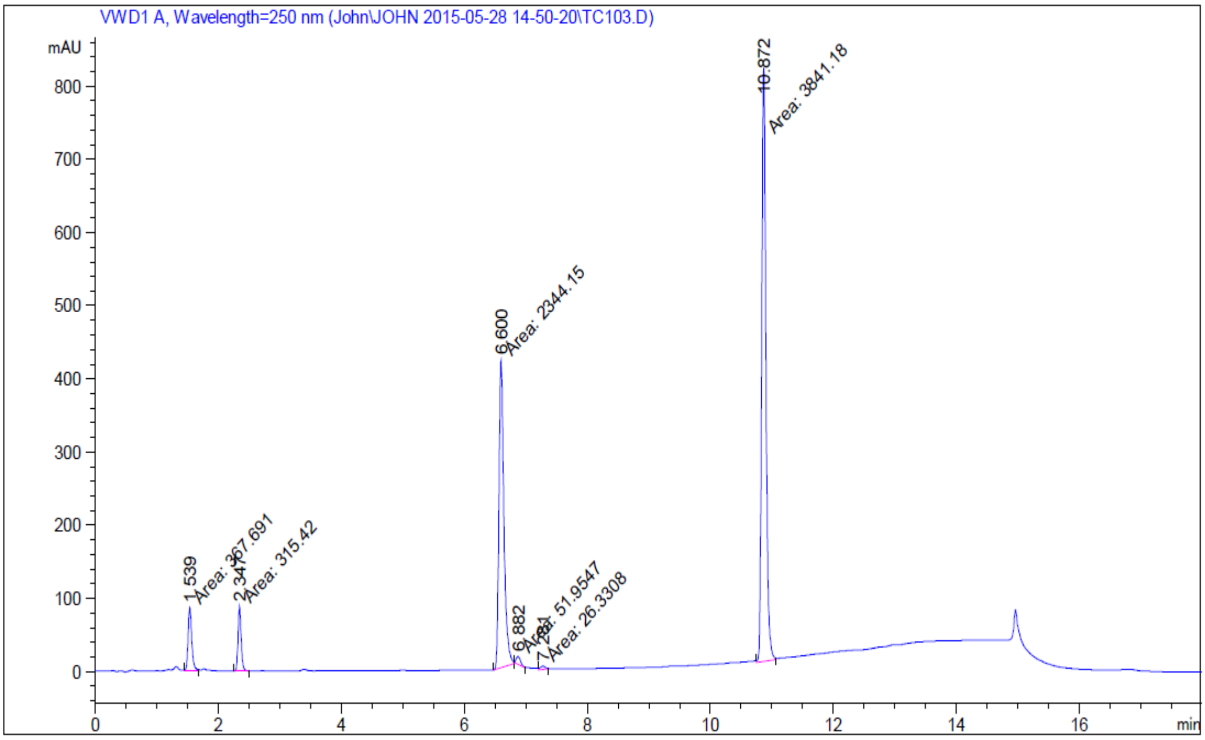

Areas: caf = 315.419, **22c** = 31.774, **2c** = 26.330

HPLC of (2-bromophenyl)boronic acid, MIDA ester (**25f**) *vs.* [1,1'-biphenyl]-4-ylboronic acid, pinacol ester (**2b**)

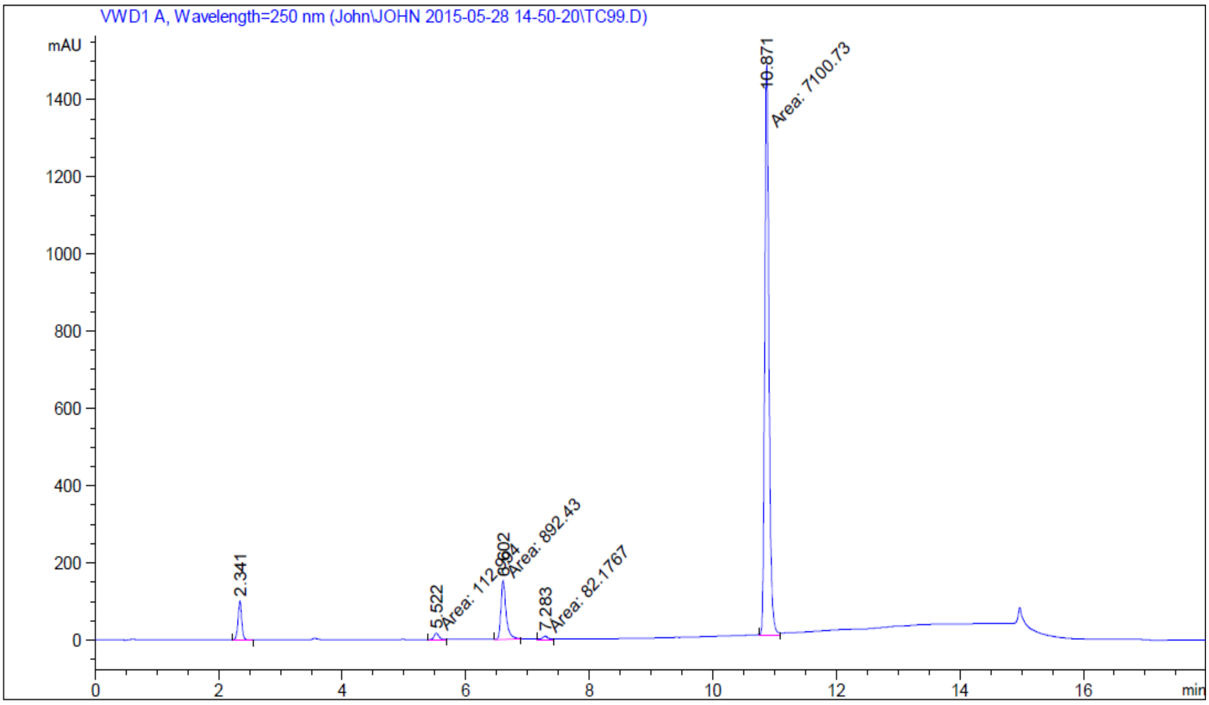

Areas: caf = 418.060, **25c** = 112.994, **2c** = 82.177

HPLC of (3-isobutoxyphenyl)boronic acid, MIDA ester (**26f**) *vs.* [1,1'-biphenyl]-4-ylboronic acid, pinacol ester (**2b**)

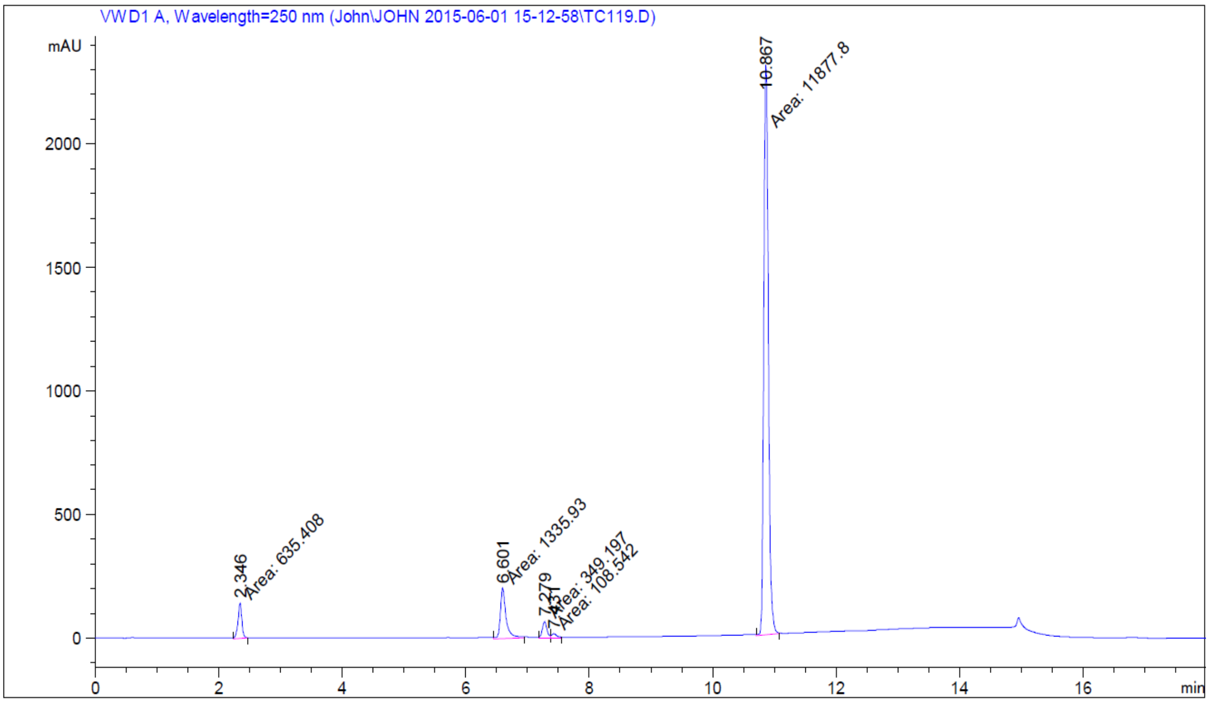

Areas: caf = 635.408, **26c** = 108.542, **2c** = 349.197

HPLC of naphthalen-2-ylboronic acid (**1a**) *vs.* (4-methoxyphenyl)boronic acid (**5a**)

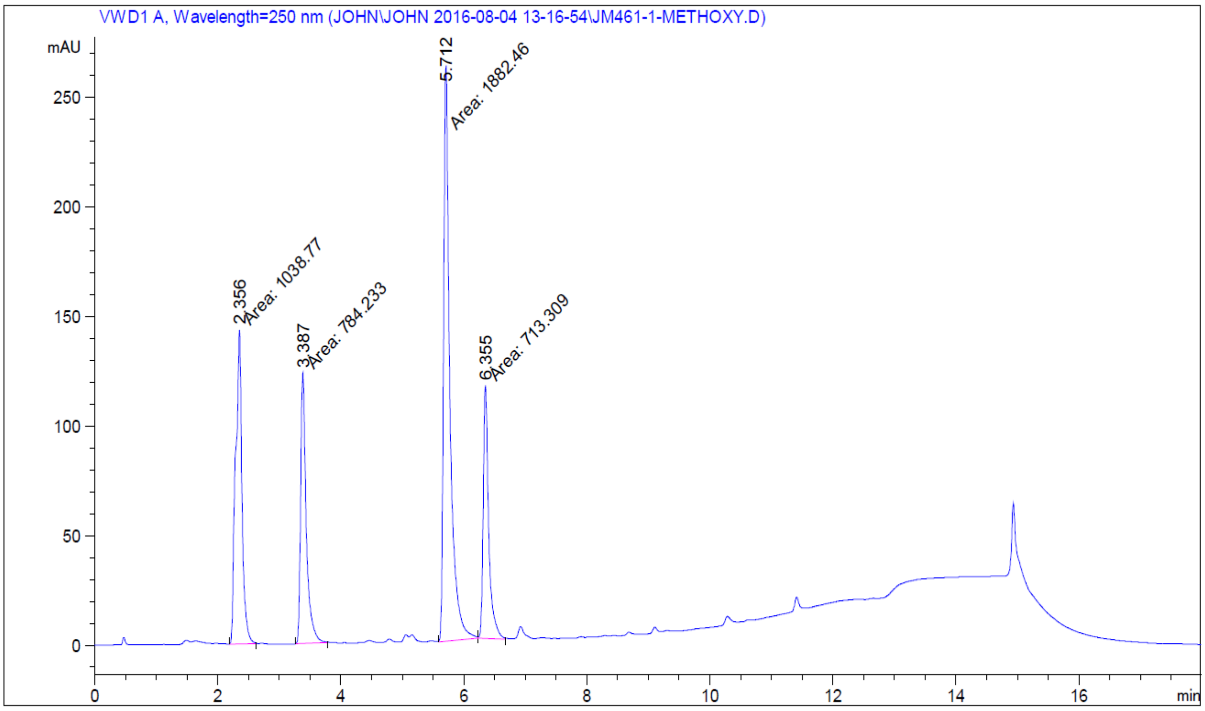

Areas: caf = 1038.77, **1c** = 713.309, **5a** = 0. (**1c** conversion factor = 0.675)

HPLC of naphthalen-2-ylboronic acid (**1a**) *vs.* phenylboronic acid (**4a**)

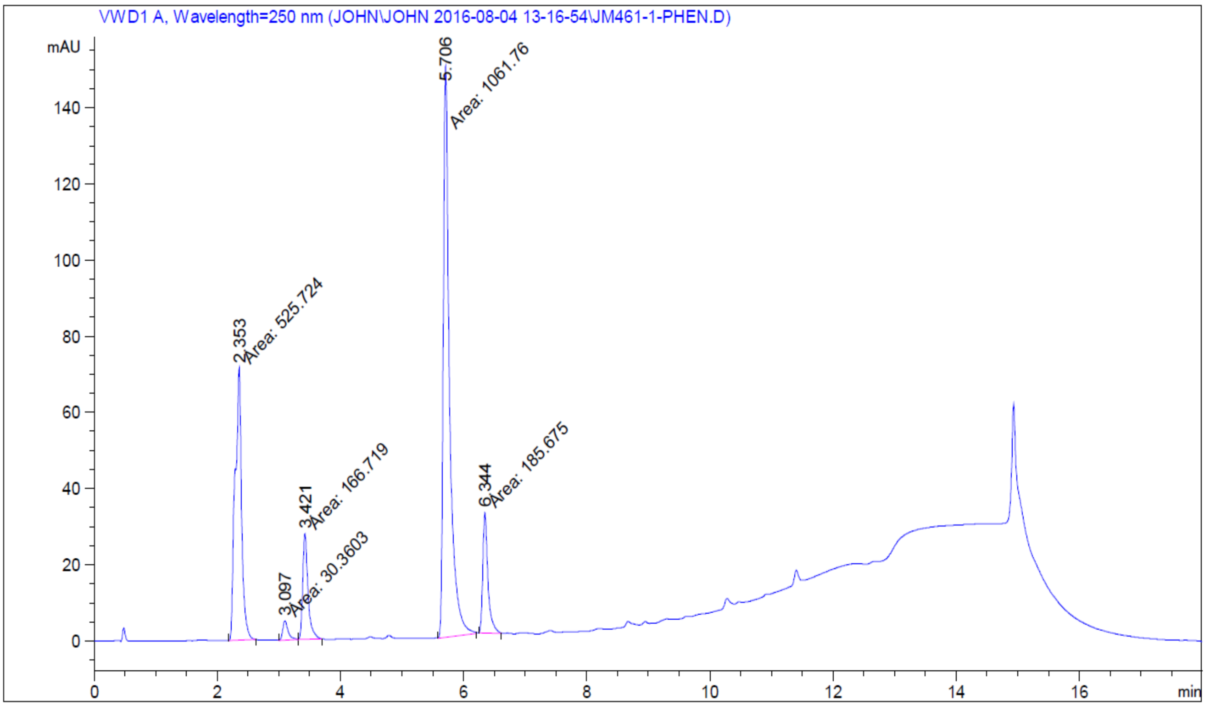

Areas: caf = 525.724, **1c** = 185.675, **4c** = 166.719. (**1c** conversion factor = 0.675, **4c** conversion factor = 0.09)

HPLC of naphthalen-2-ylboronic acid (**1a**) *vs.* (4-fluorophenyl)boronic acid (**3a**)

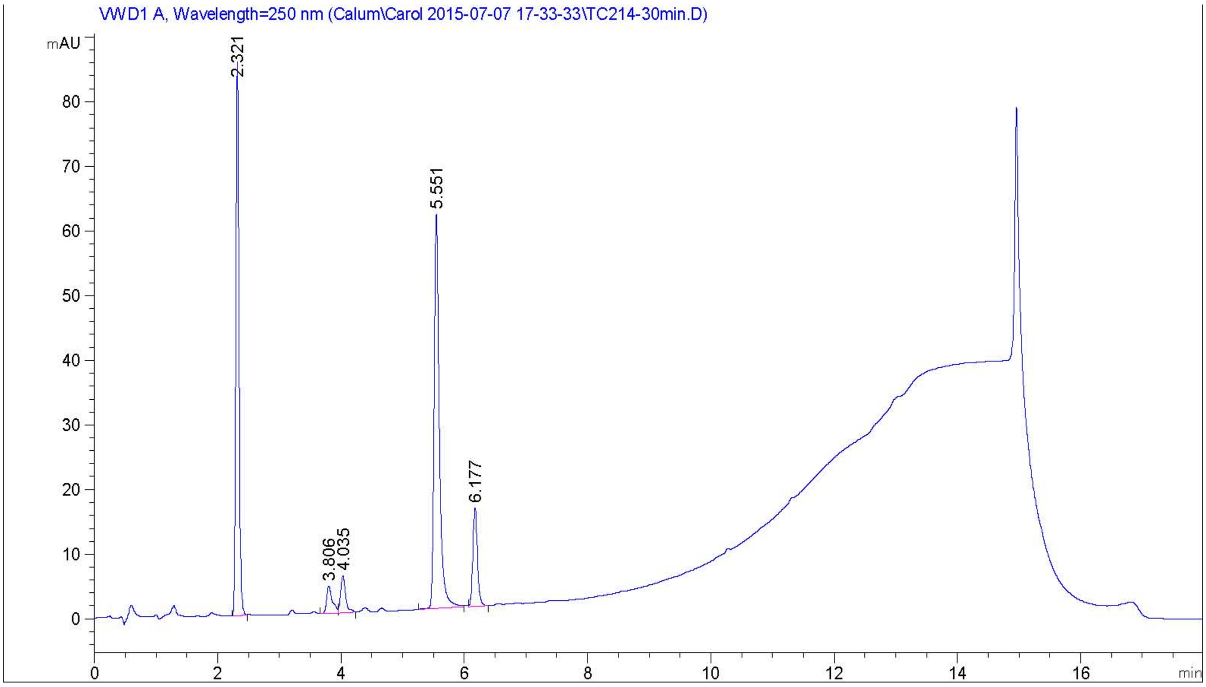

Areas: caf = 301.006, **1c** = 78.010, **3c** = 31.771

HPLC of naphthalen-2-ylboronic acid (**1a**) *vs.* (4-(methoxycarbonyl)phenyl)boronic acid (**7a**)

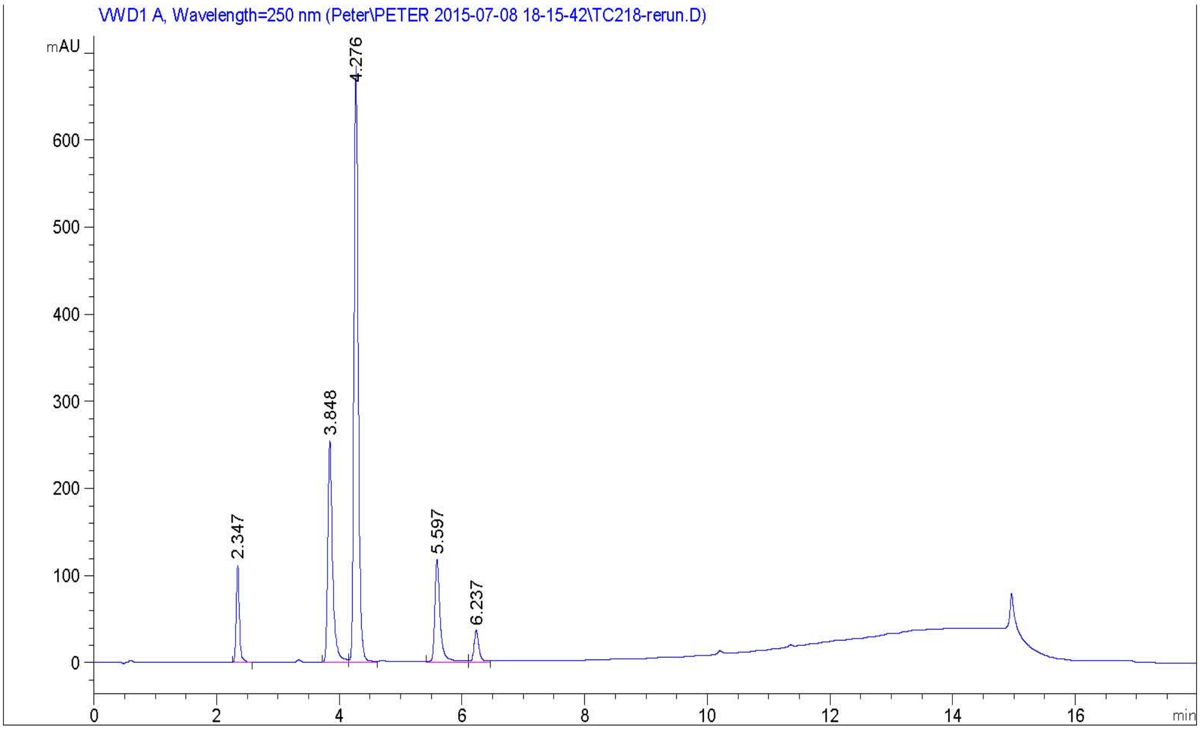

Areas: caf = 406.447, **1c** = 216.428, **7c** = 3277.499

HPLC of naphthalen-2-ylboronic acid (**1a**) *vs.* (4-nitrophenyl)boronic acid (**29a**)

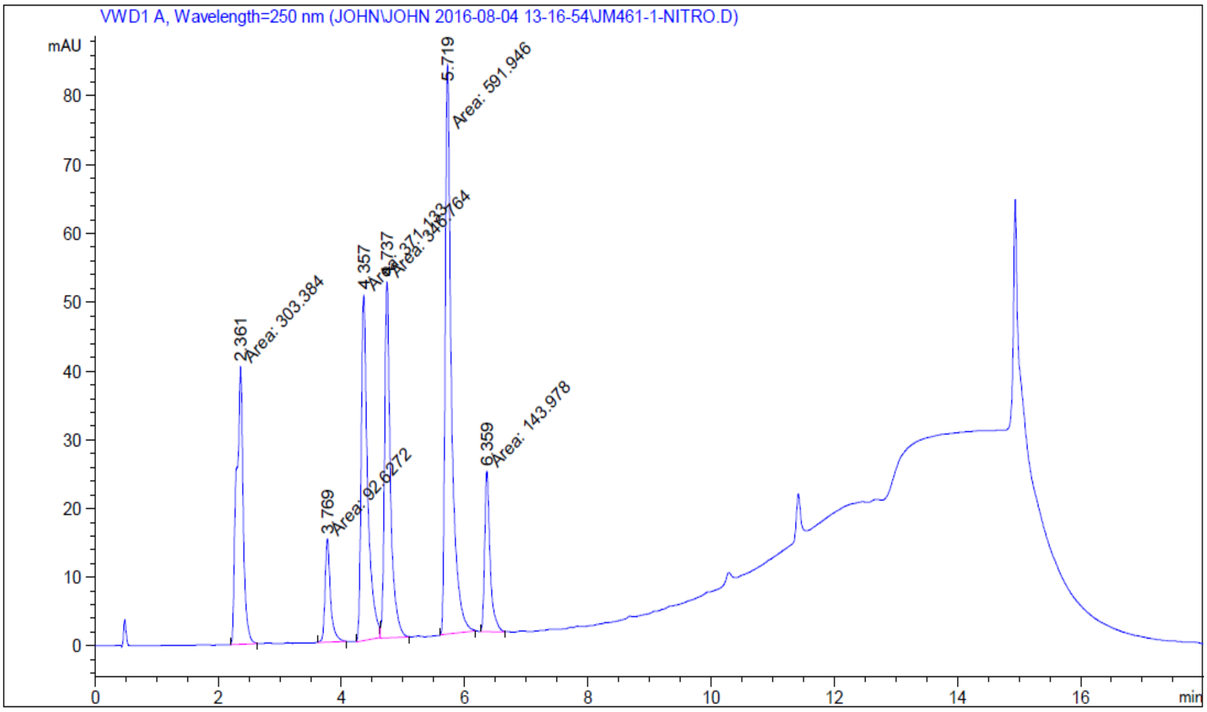

Areas: caf = 303.384, **1c** = 143.978, **29c** = 346.764. (1c conversion factor = 0.675)

HPLC of naphthalen-2-ylboronic acid (**1a**) *vs.* (2-methoxypyridin-3-yl)boronic acid (**14a**)

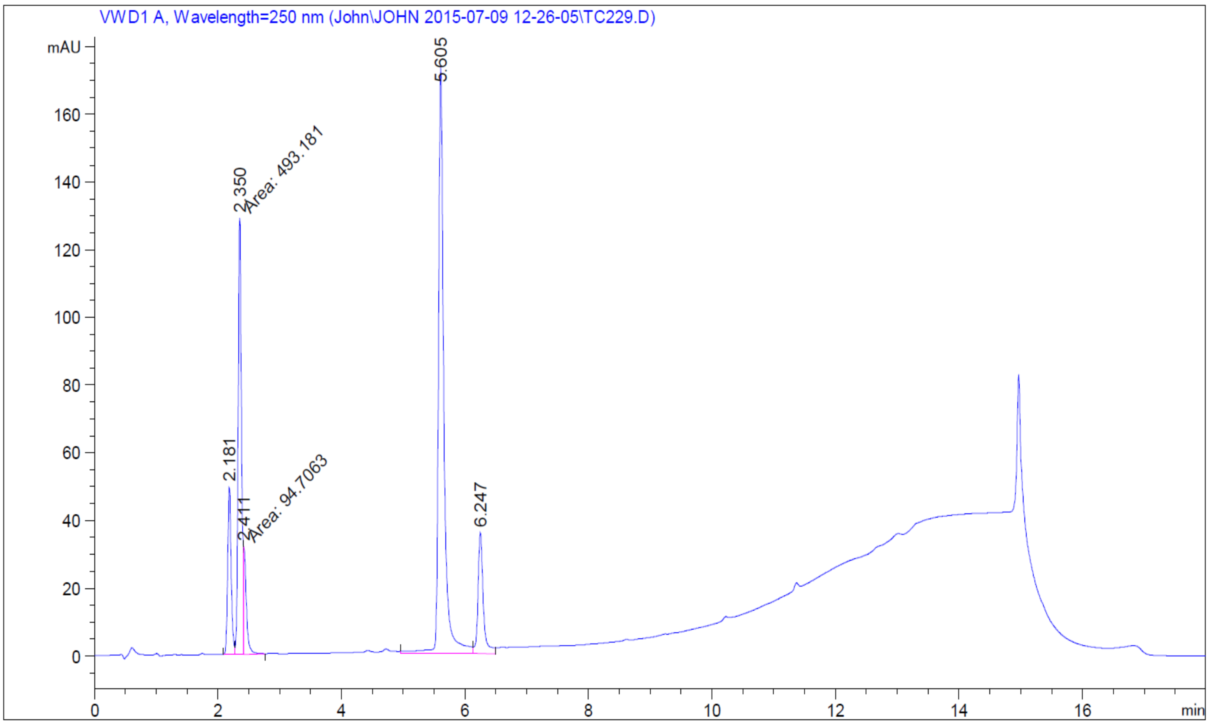

Areas: caf = 487.781, **1c** = 211.965, **14c** = 193.977

HPLC of naphthalen-2-ylboronic acid (**1a**) *vs.* (2,3-dihydrobenzo[b][1,4]dioxin-6-yl)boronic acid (**30a**)

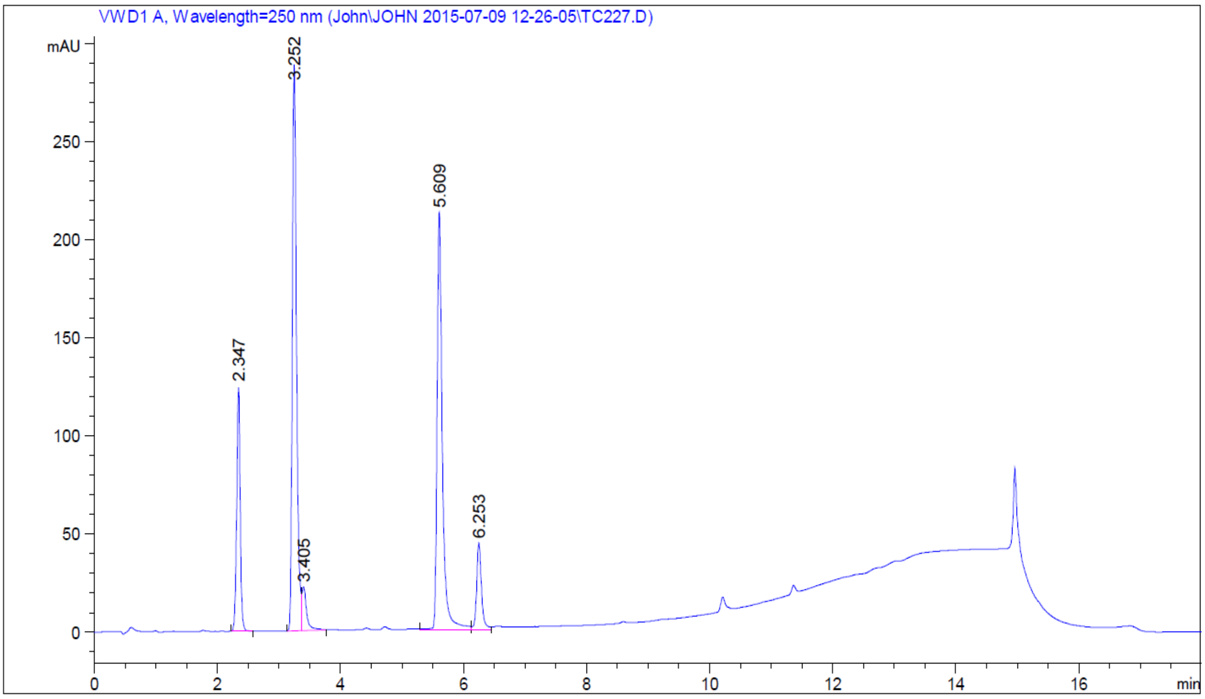

Areas: caf = 495.489, **1c** = 250.989, **29c** = 119.822

HPLC of (2-methoxypyridin-3-yl)boronic acid (**14a**) *vs.* (3-isobutoxyphenyl)boronic acid (**26a**)

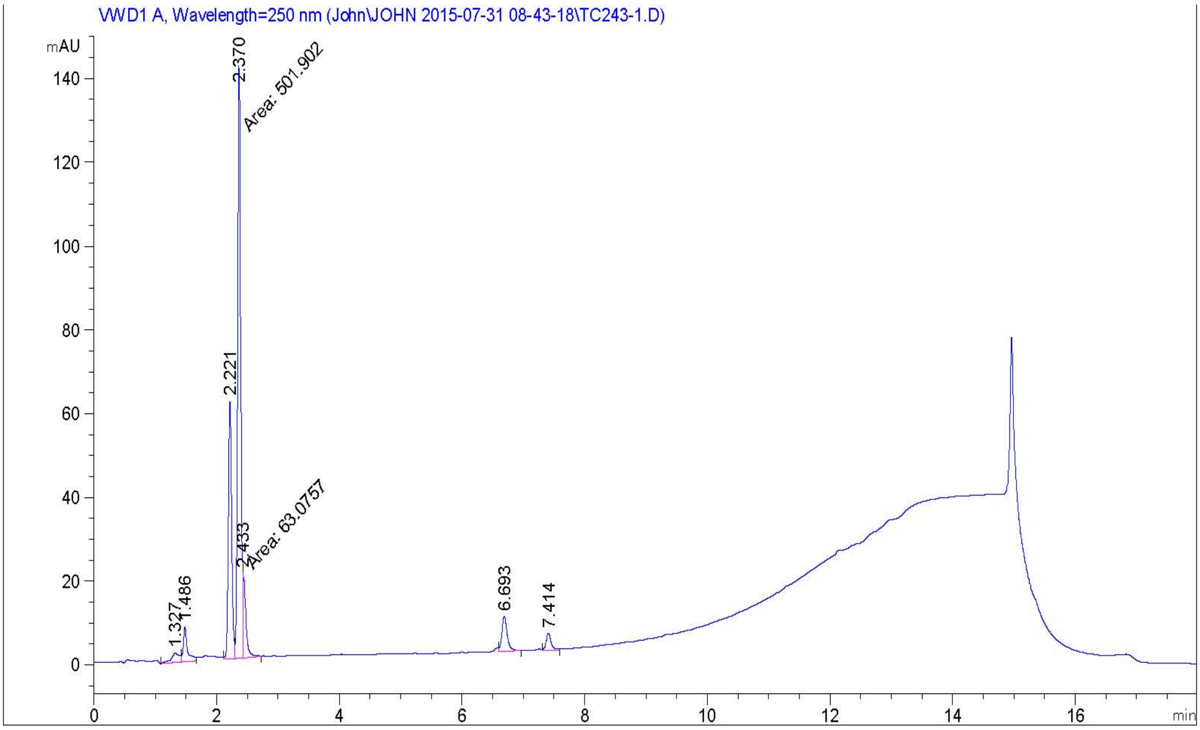

Areas: caf = 501.901, **14c** = 227.721, **26c** = 25.260

HPLC of naphthalen-2-ylboronic acid (**1a**) *vs.* pyridin-3-ylboronic acid (**31a**)

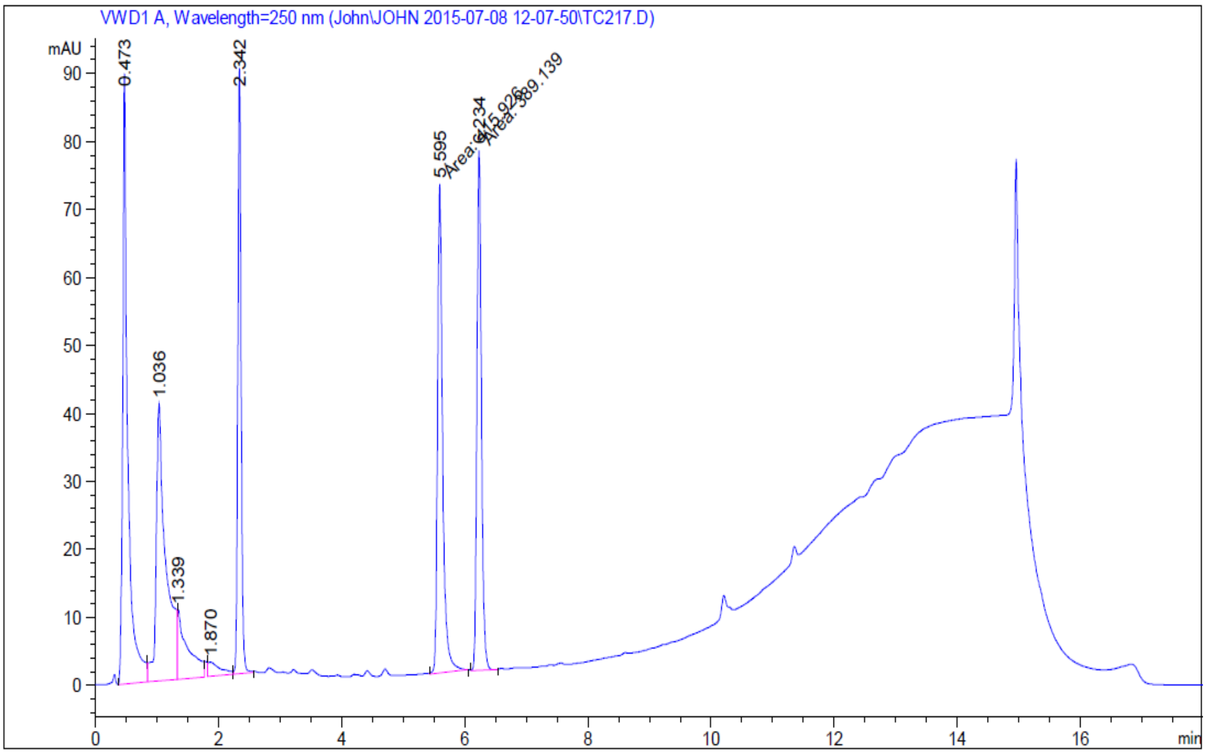

Areas: caf = 331.332, **1c** = 389.139, **30c** = 101.193

HPLC of (2-methoxypyridin-3-yl)boronic acid (**14a**) *vs.* (2-bromophenyl)boronic acid (**25a**)

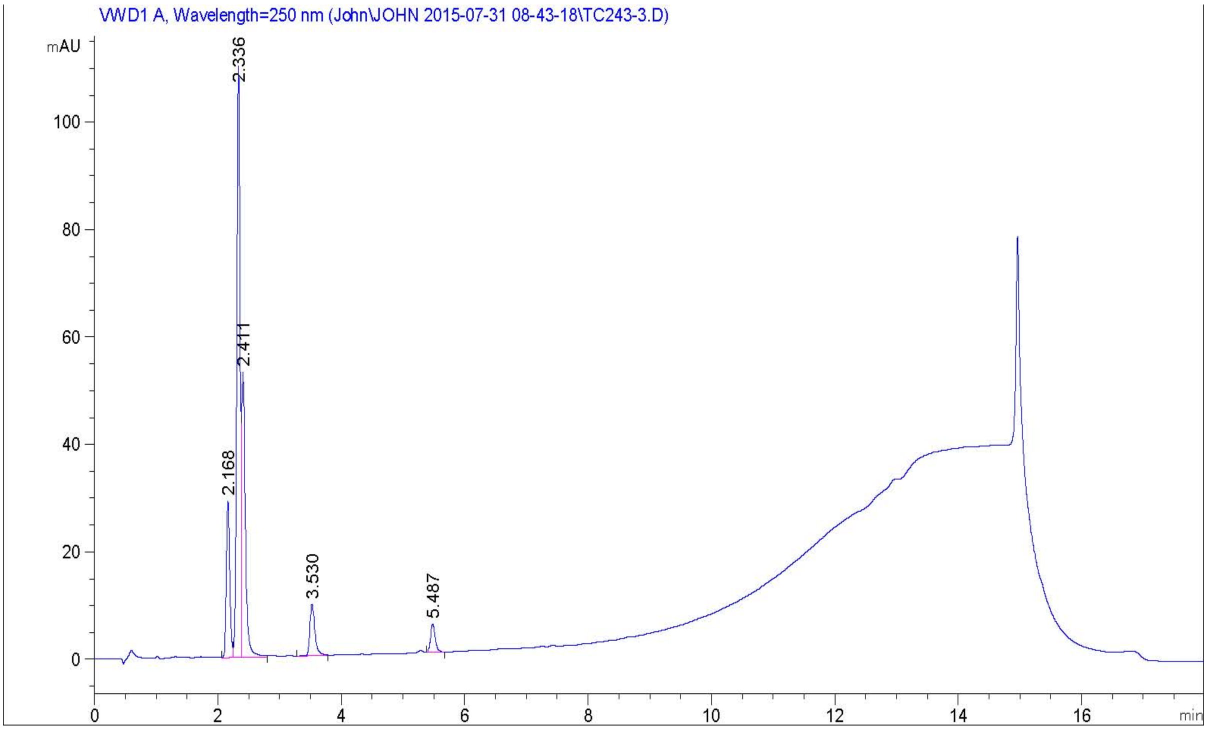

Areas: caf = 415.603, **14c** = 111.991, **25c** = 27.312

13. Appendices

Spectra of Boron Species Controls for NMR Study

^{11}B NMR of $B(OH)_4^-$

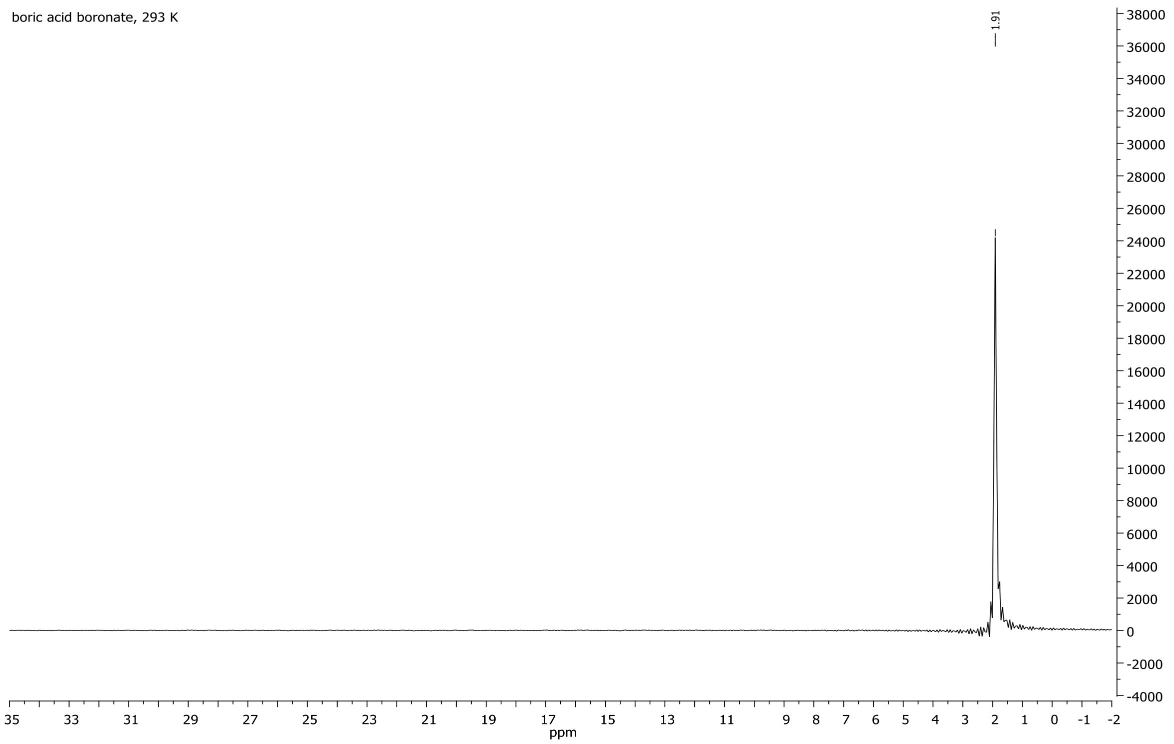

^{11}B NMR of naphthalen-2-ylboronic acid, **1a**

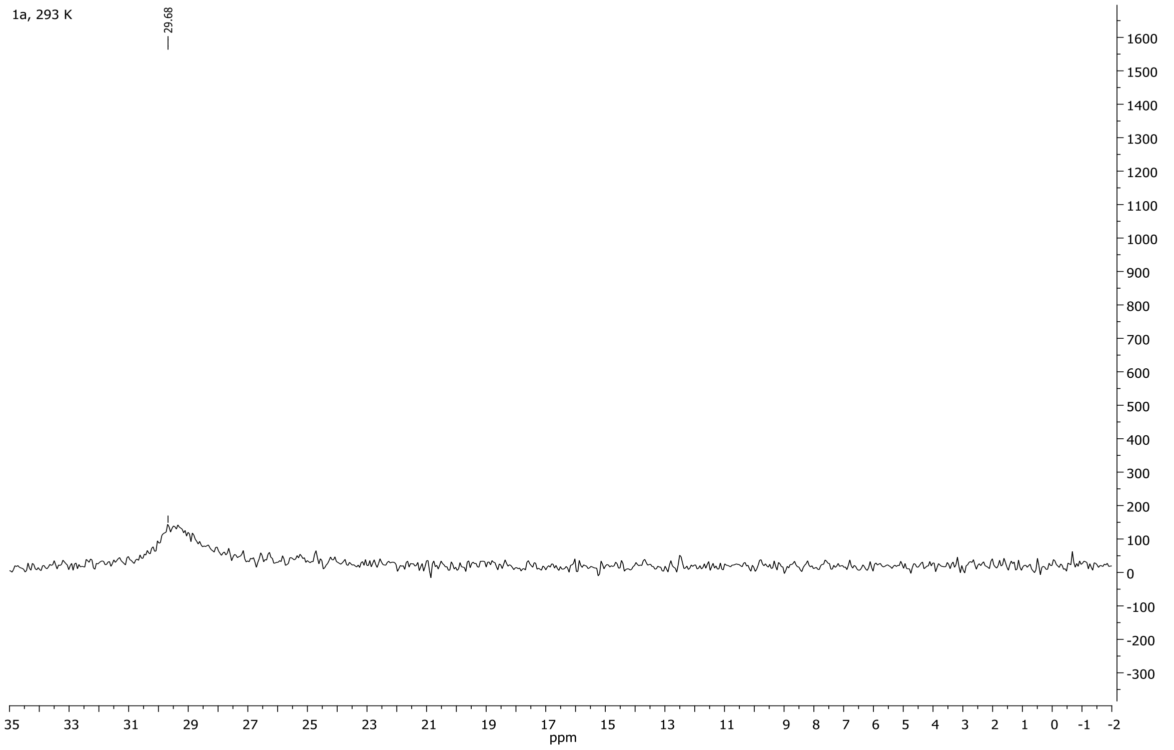

^{11}B NMR of potassium trihydroxy(naphthalen-2-yl)borate, **1d**

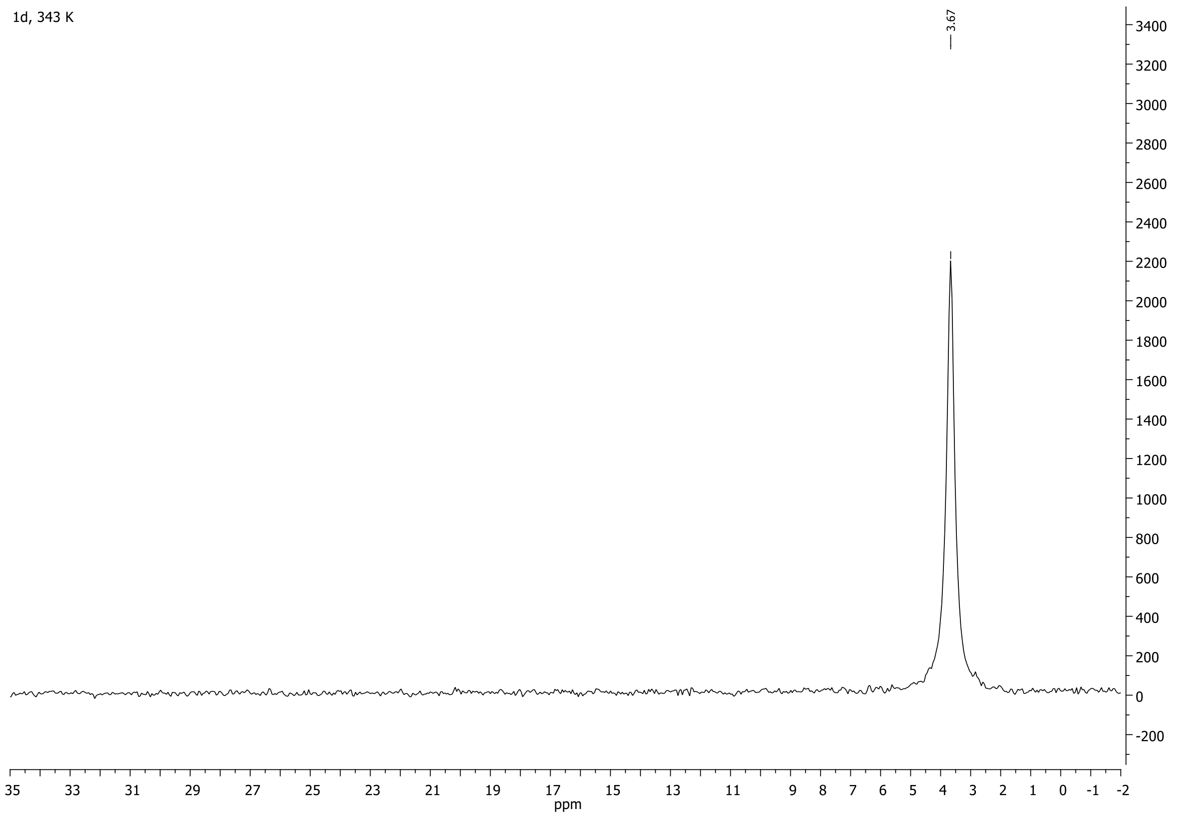

^{11}B NMR of [1,1'-biphenyl]-4-ylboronic acid, pinacol ester, **2b**

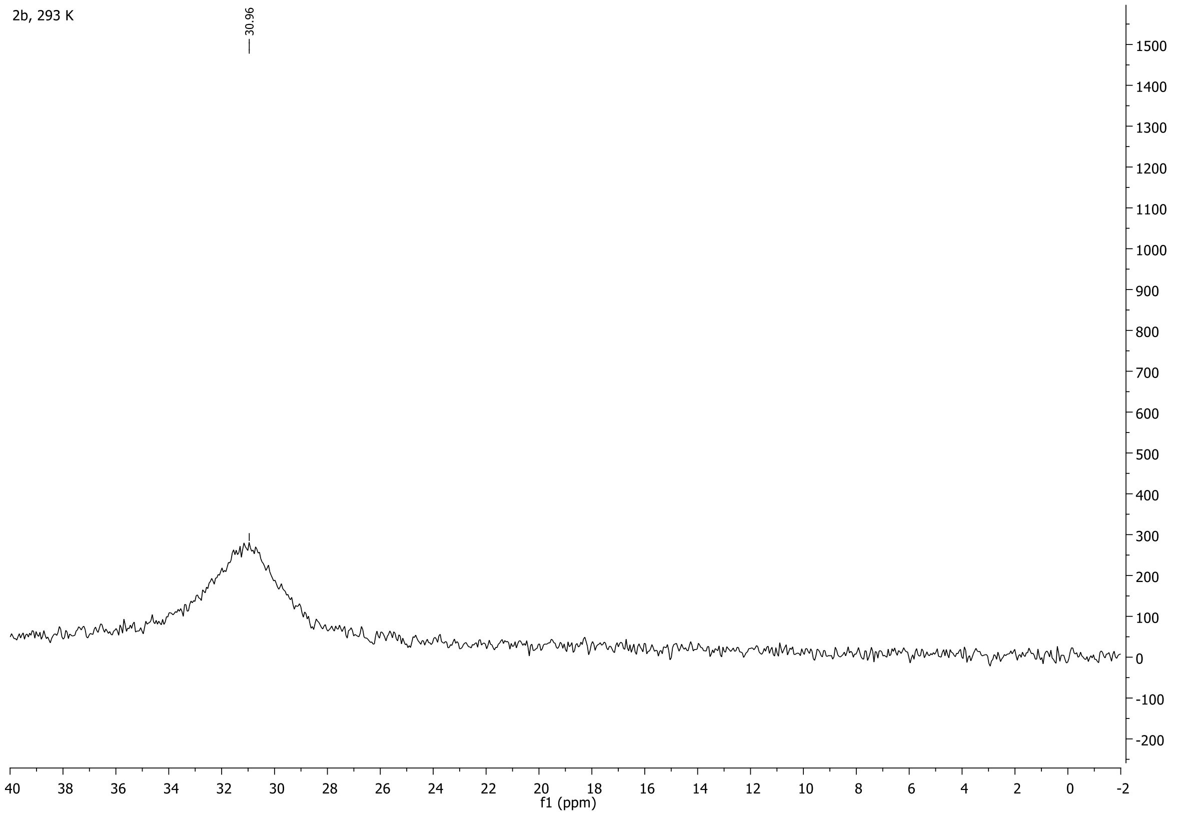

^{11}B NMR of potassium [1,1'-biphenyl]-4-yltrihydroxyborate, **2d**

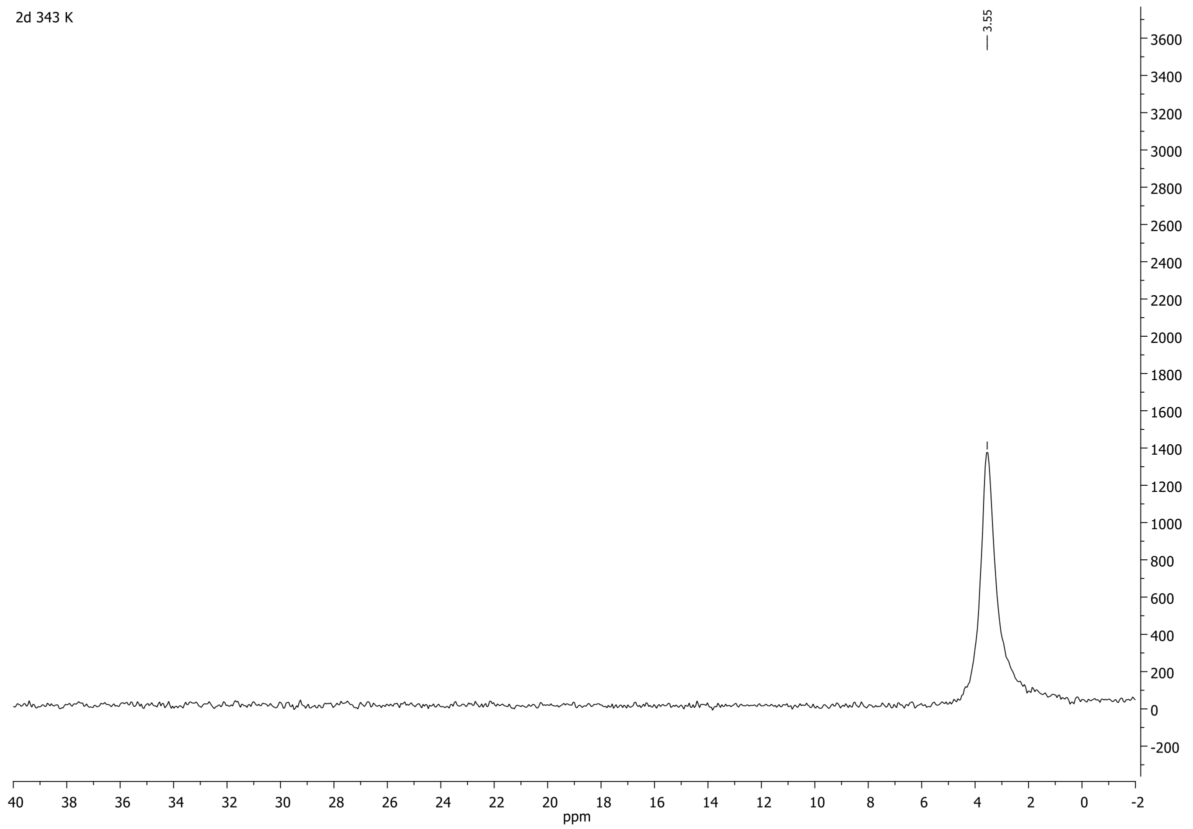

^{11}B NMR of potassium [1,1'-biphenyl]-4-yltrihydroxyborate, pinacol ester, **2e**

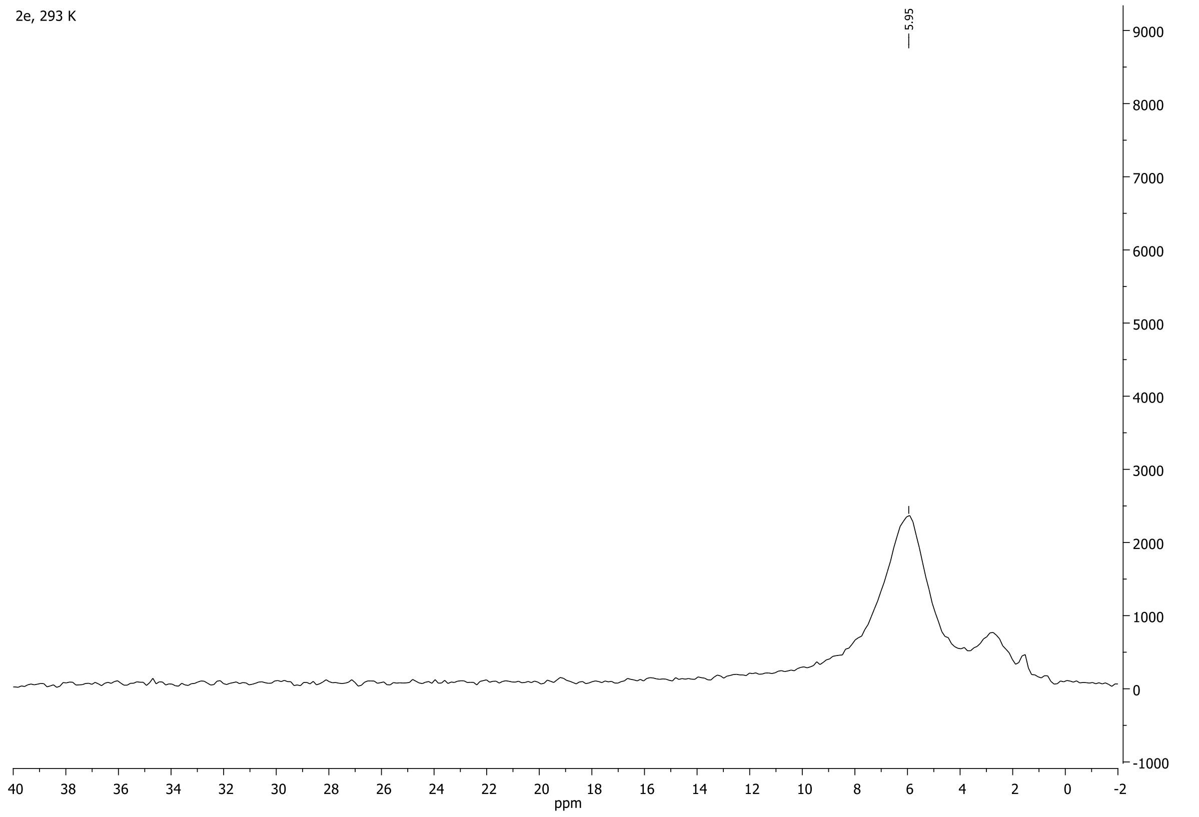

^{11}B NMR of potassium trihydroxy(naphthalen-2-yl)borate (**1d**) and potassium [1,1'-biphenyl]-4-yltrihydroxyborate, pinacol ester (**2e**)

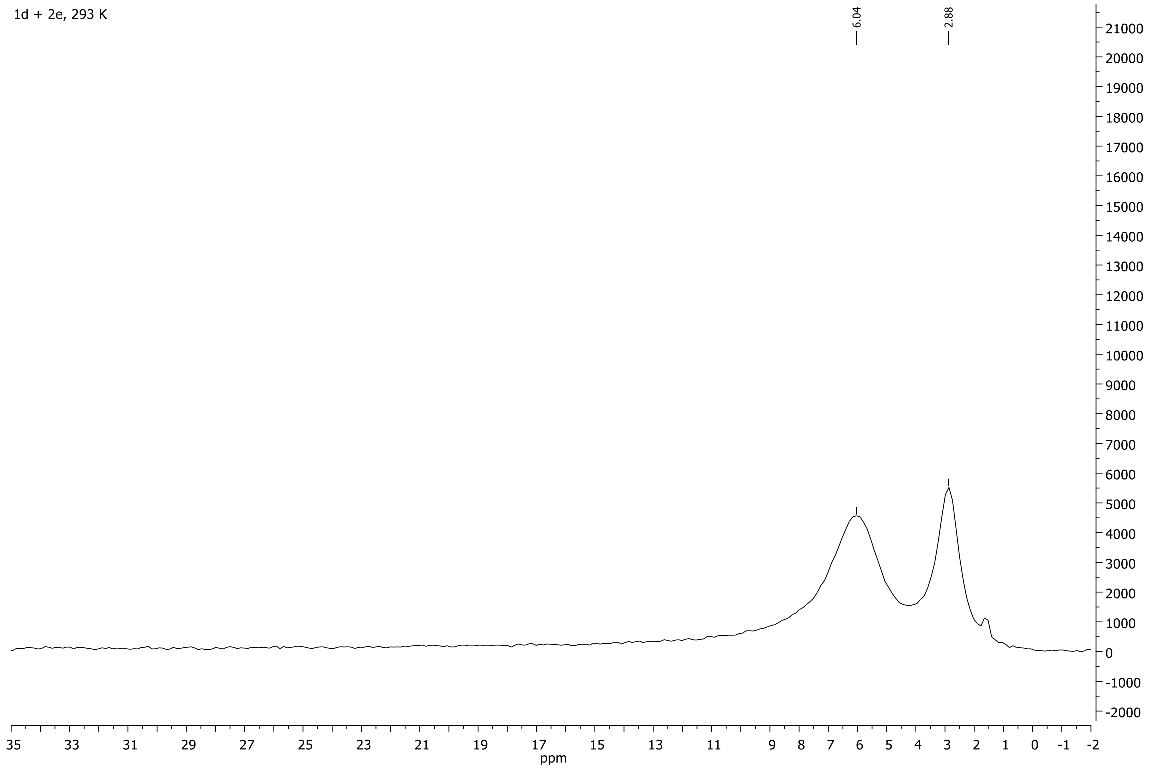

^{11}B NMR of (4-fluorophenyl)boronic acid, **3a**

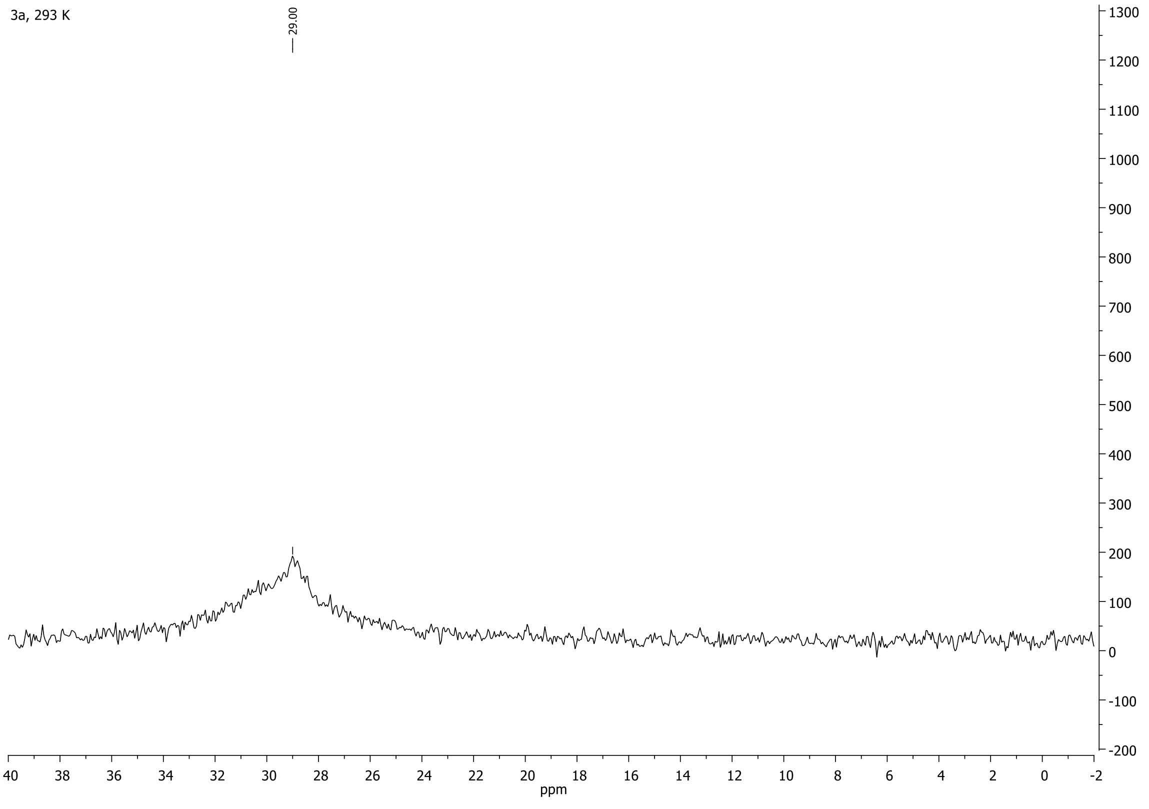

^{19}F NMR of (4-fluorophenyl)boronic acid, **3a**

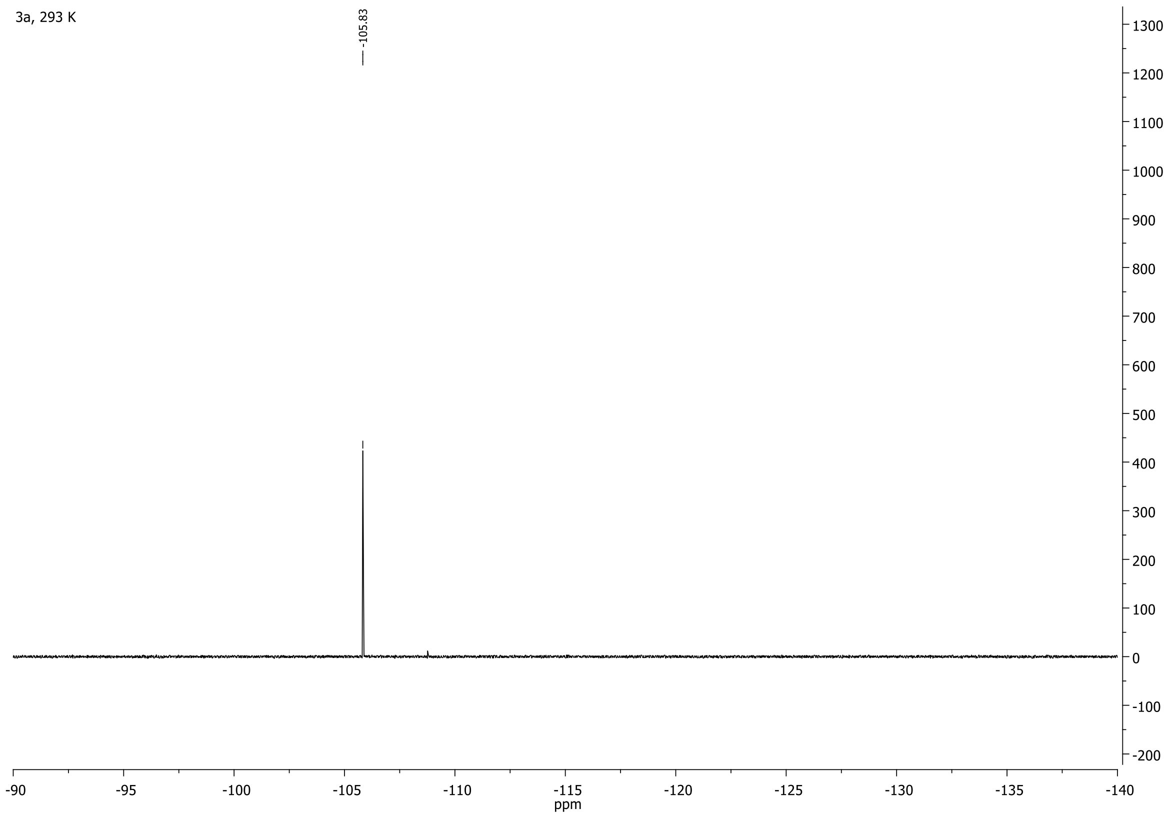

^{11}B NMR of (4-fluorophenyl)boronic acid, pinacol ester, **3b**

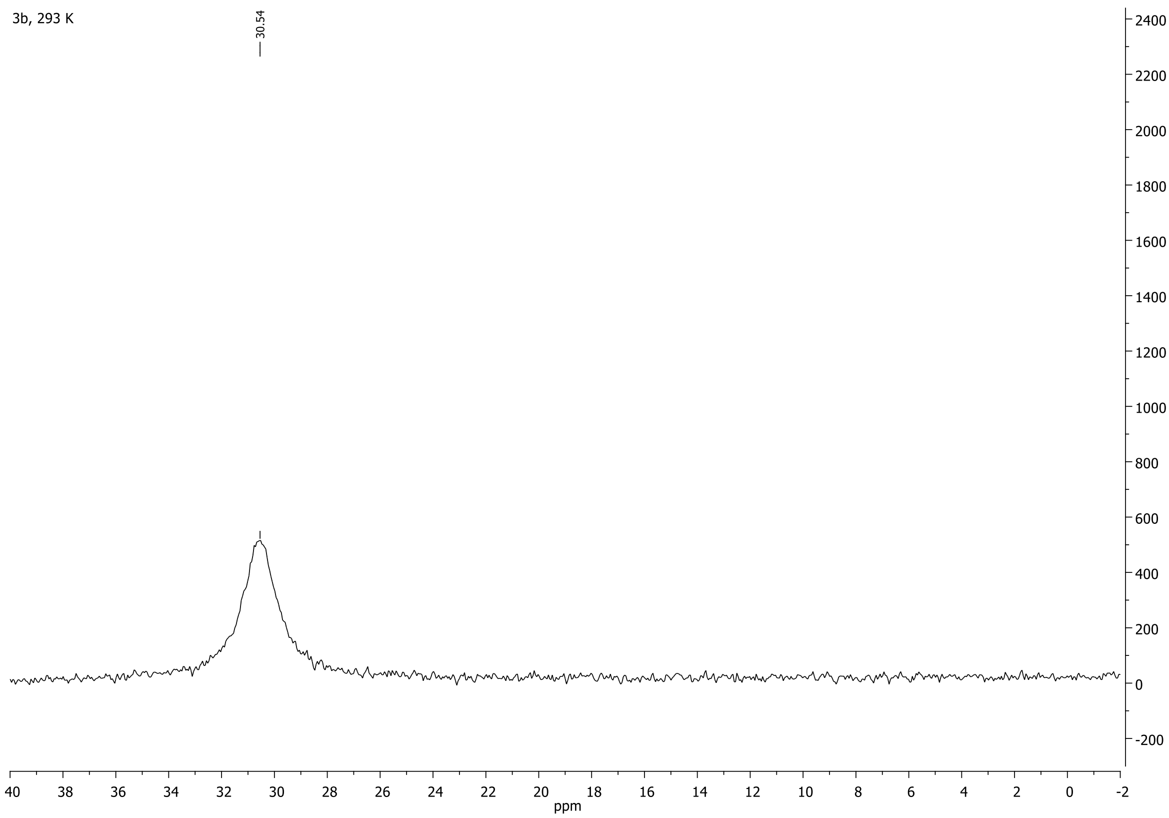

^{19}F NMR of (4-fluorophenyl)boronic acid, pinacol ester **3b**

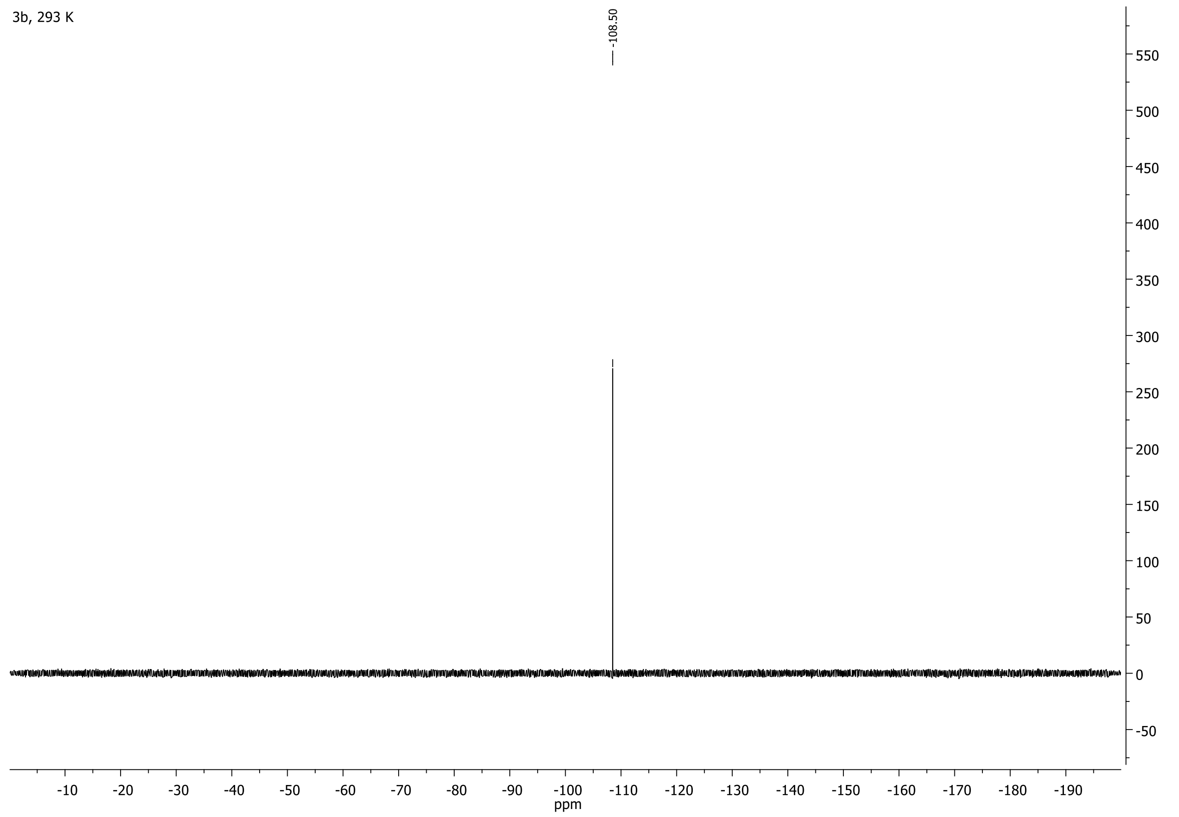

^{11}B NMR of Potassium (4-fluorophenyl)trihydroxyborate, **3d**

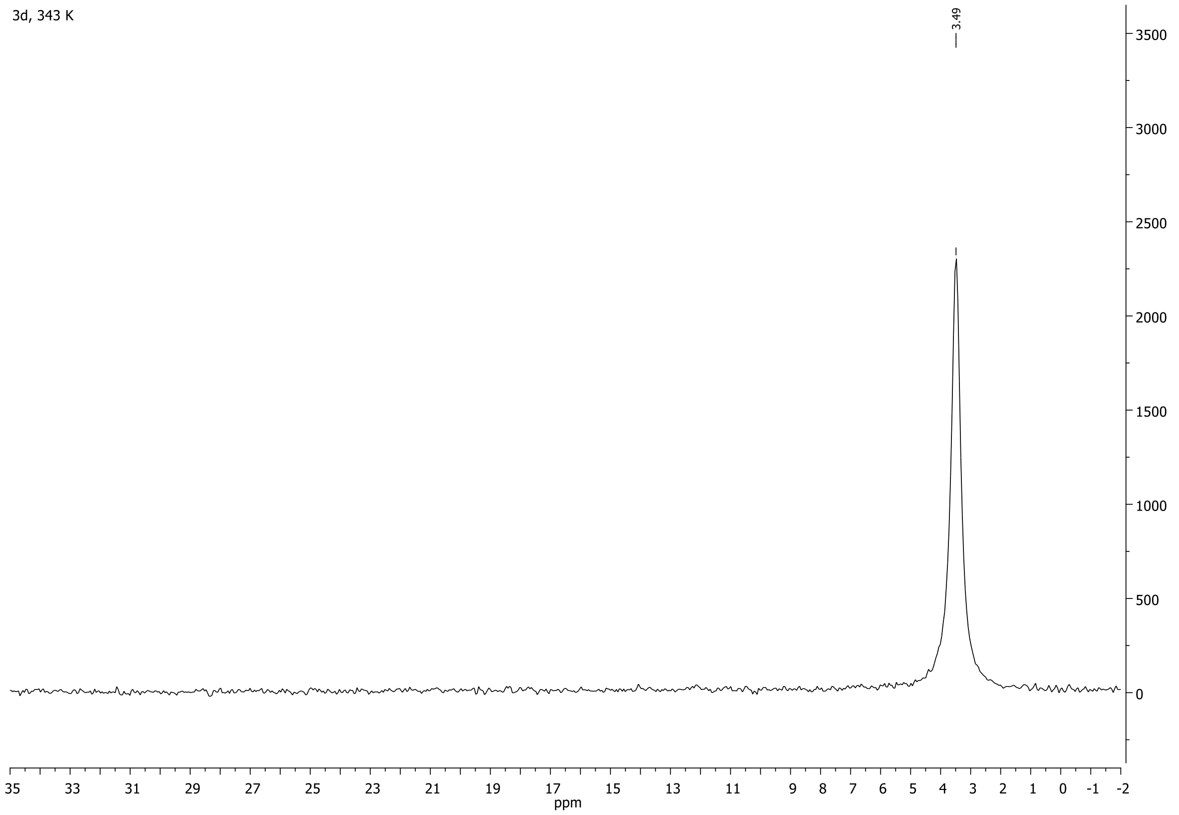

^{19}F NMR of Potassium (4-fluorophenyl)trihydroxyborate, **3d**

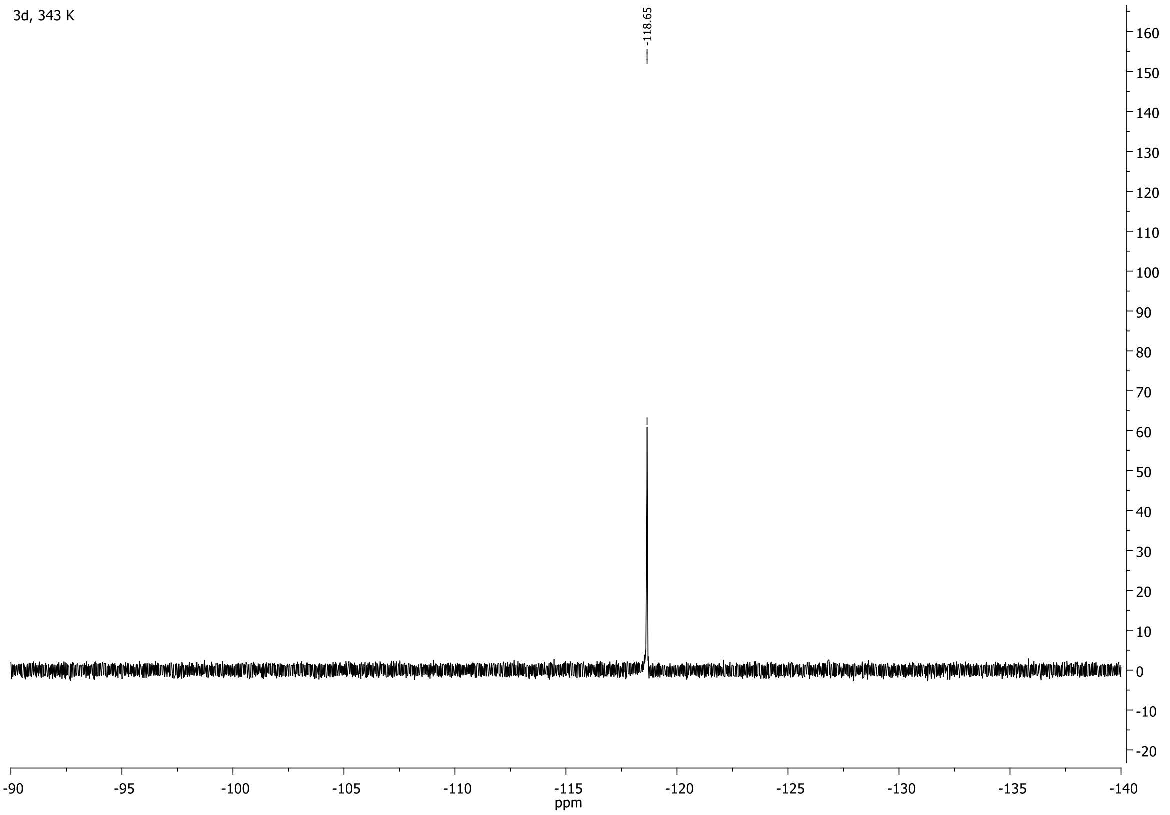

^{11}B NMR of Potassium (4-fluorophenyl)trihydroxyborate, pinacol ester, **3e**

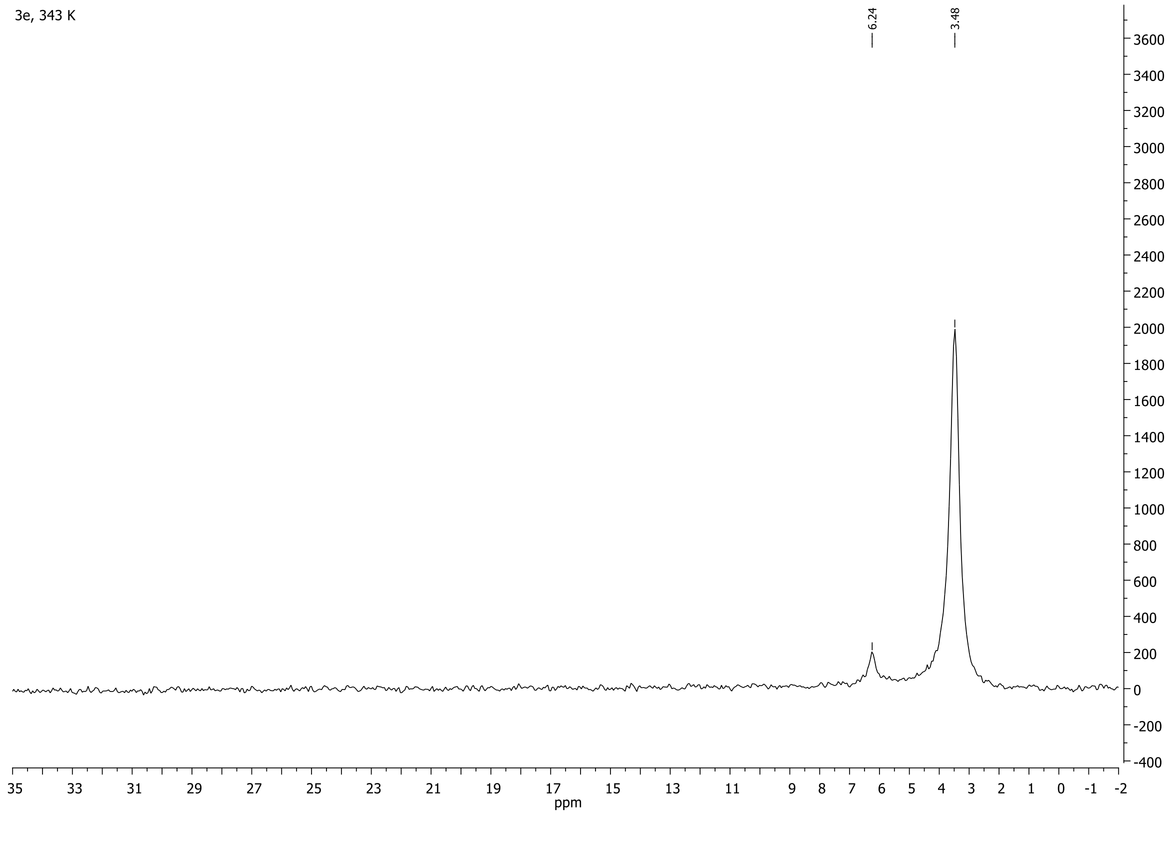

^{19}F NMR of Potassium (4-fluorophenyl)trihydroxyborate, pinacol ester, **3e**

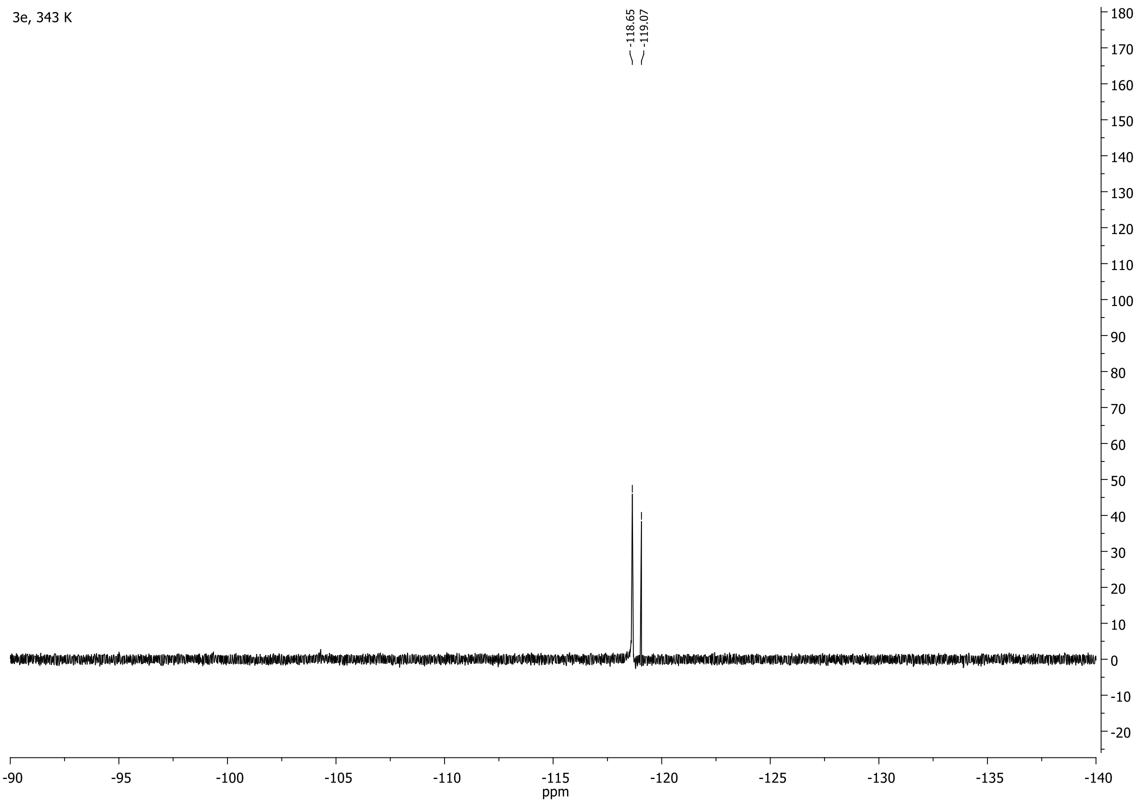

Spectra for BMIDA Intermediates

^{1}H NMR of (1*H*-indol-5-yl)boronic acid, MIDA ester, **8f**

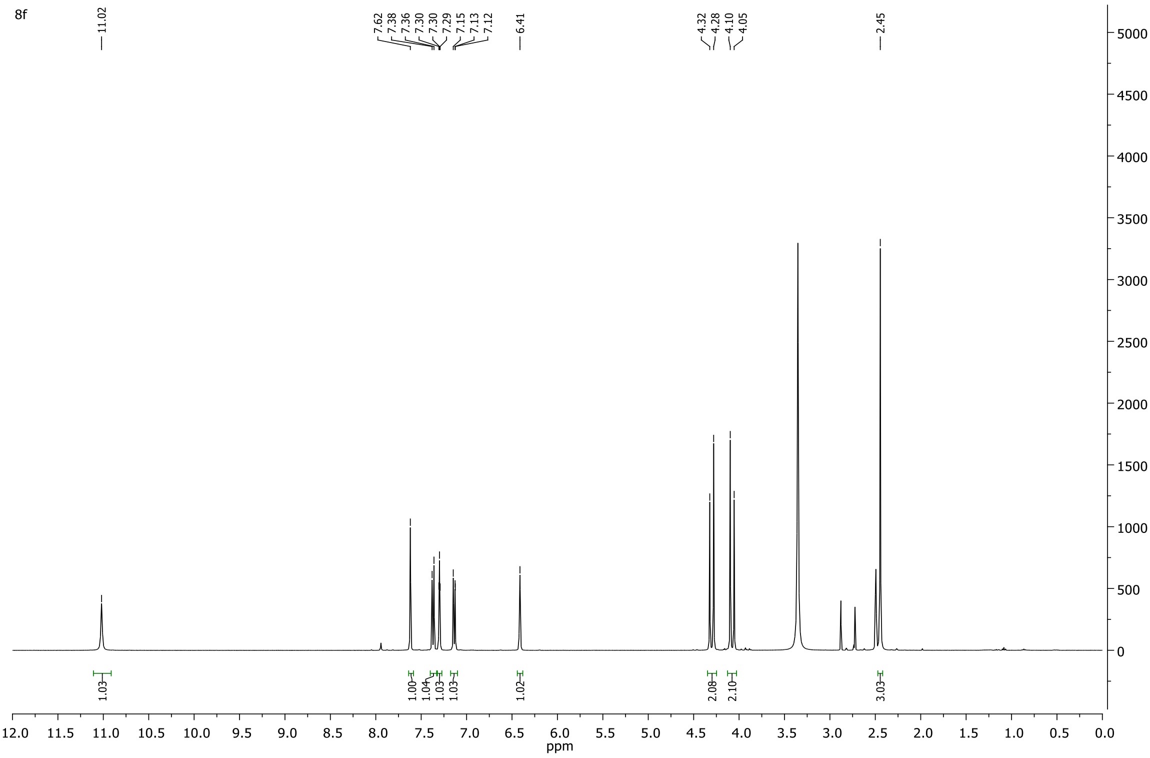

^{13}C NMR of (1*H*-indol-5-yl)boronic acid, MIDA ester, **8f**

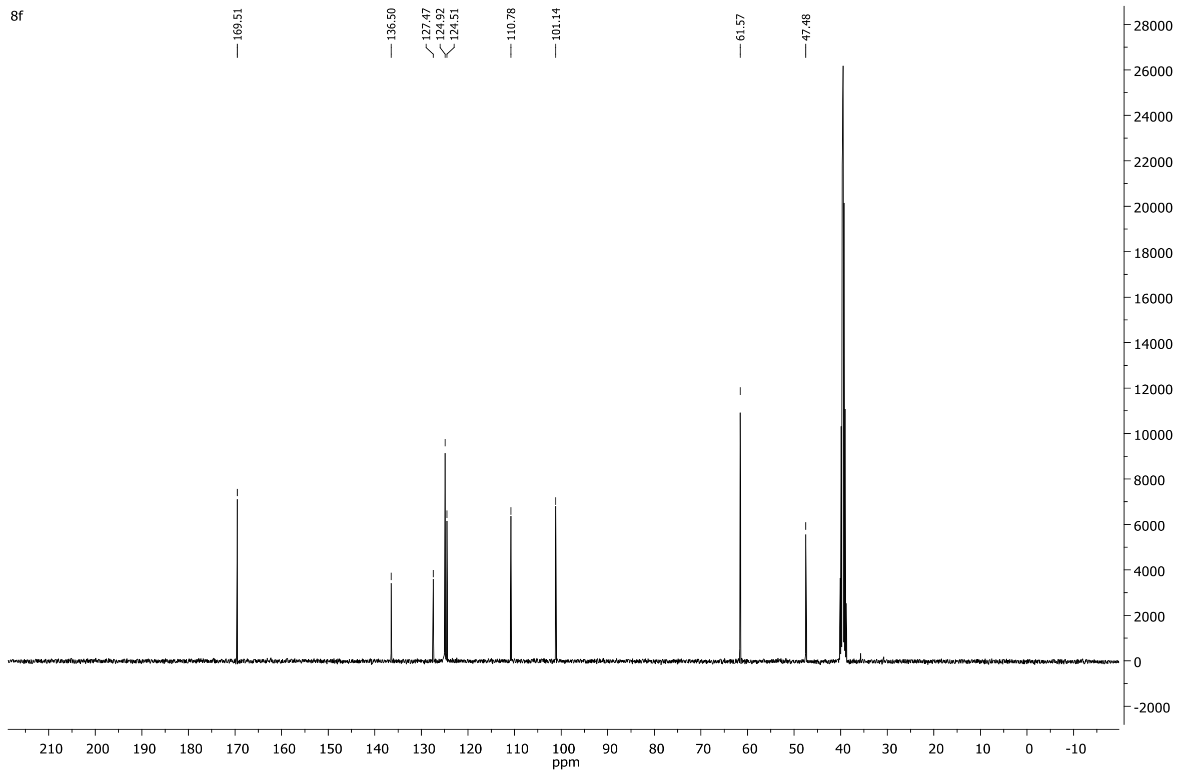

HMRS of (1*H*-indol-5-yl)boronic acid, MIDA ester, **8f**

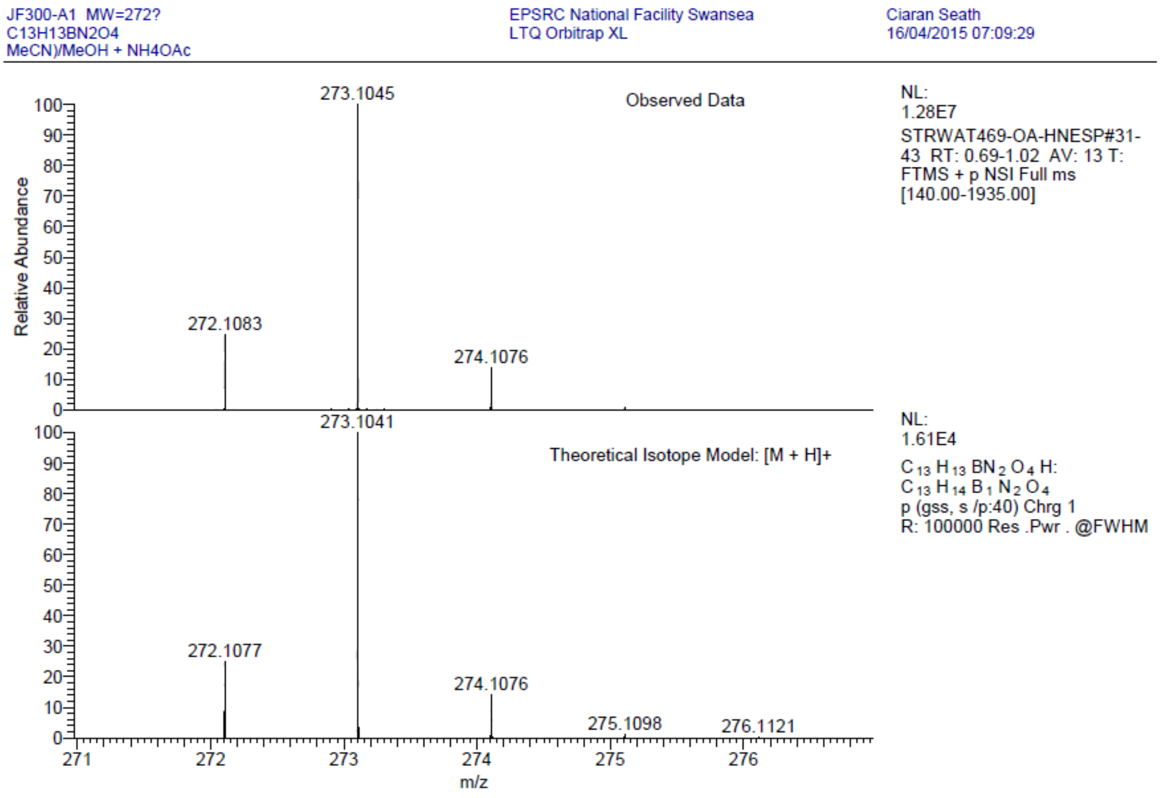

^{1}H NMR of (benzofuran-5-yl)boronic acid, MIDA ester, **13f**

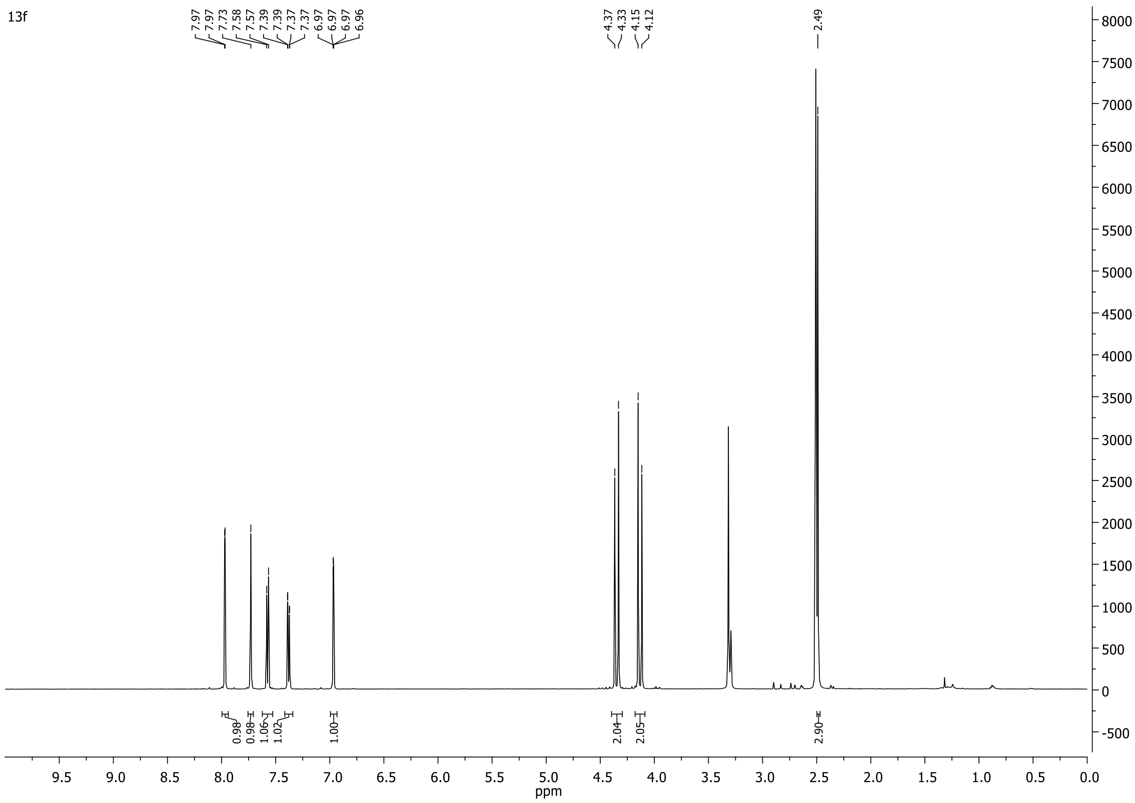

^{13}C NMR of (benzofuran-5-yl)boronic acid, MIDA ester, **13f**

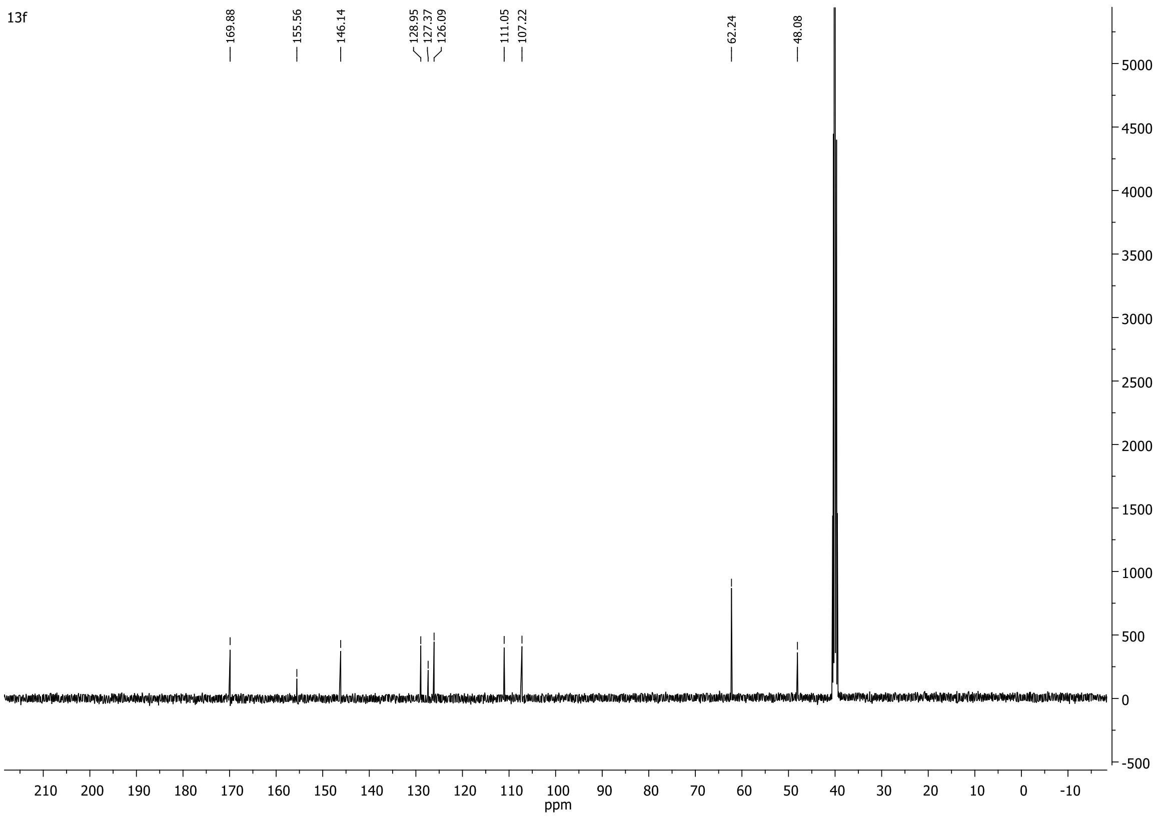

HRMS of (benzofuran-5-yl)boronic acid, MIDA ester, **13f**

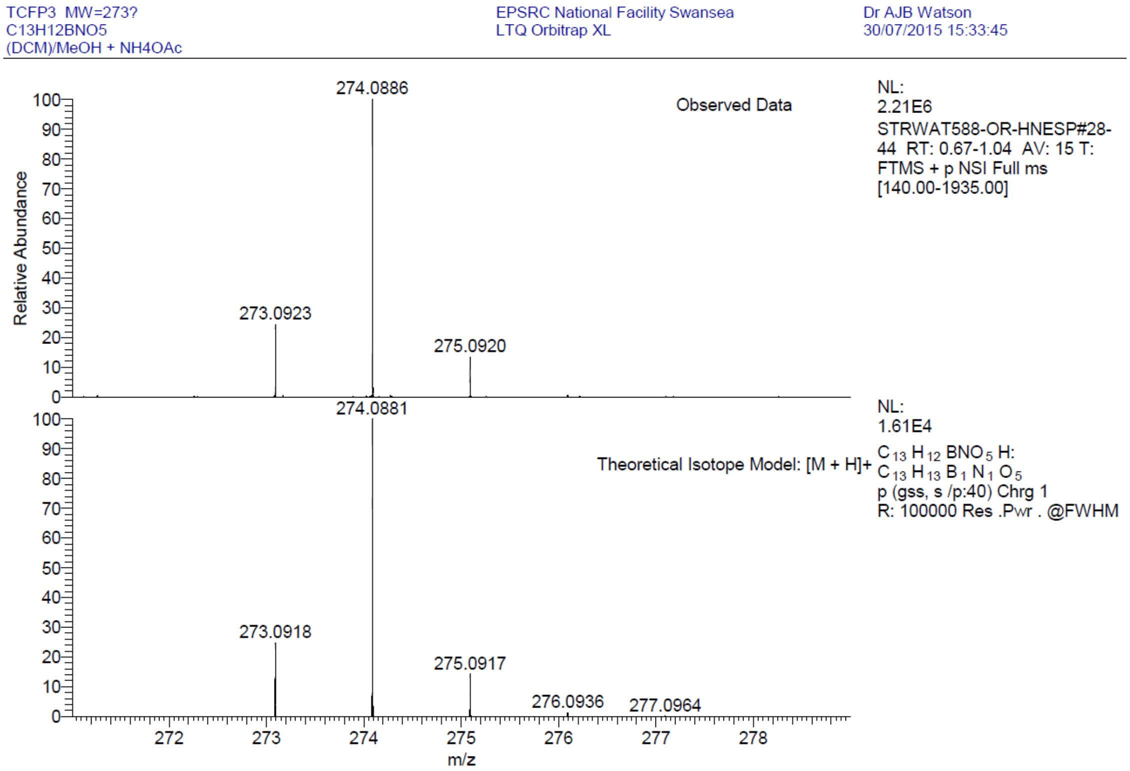

^{1}H NMR of 4-hydroxyphenylboronic acid, MIDA ester, **22f**

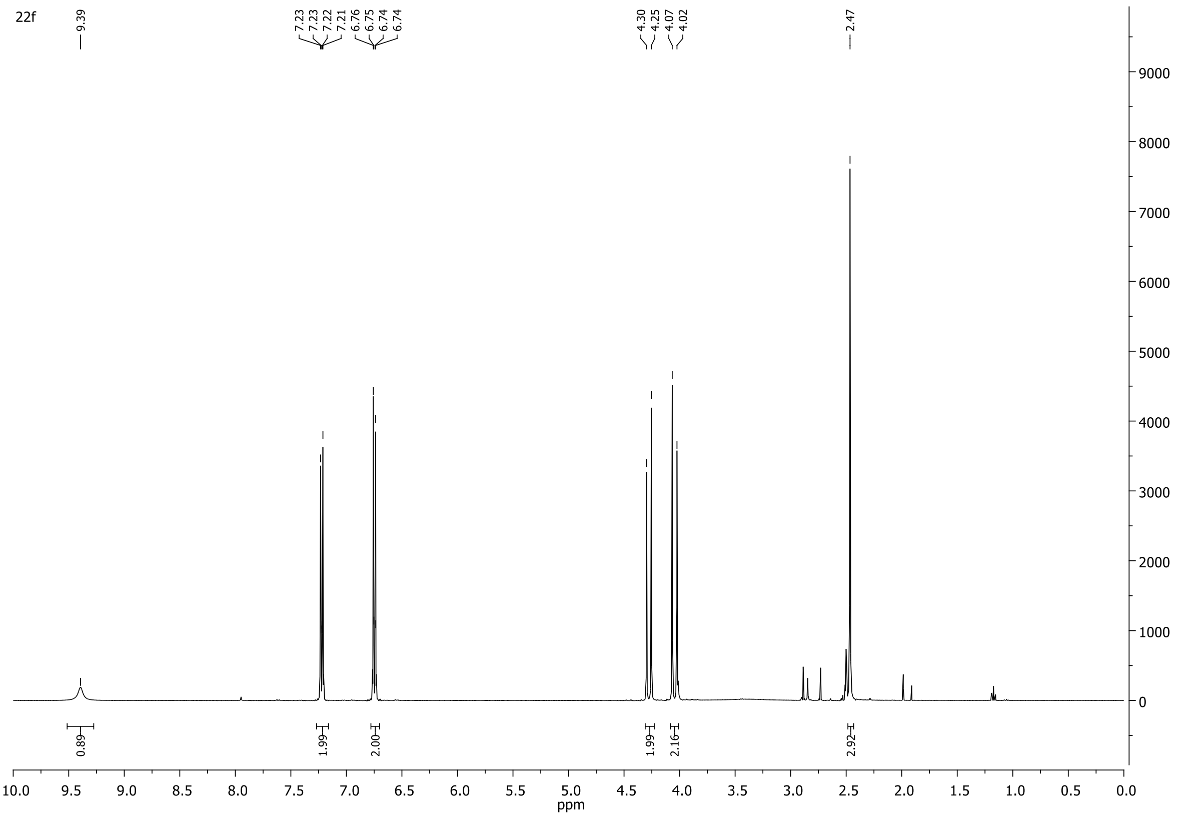

^{13}C NMR of 4-hydroxyphenylboronic acid, MIDA ester, **22f**

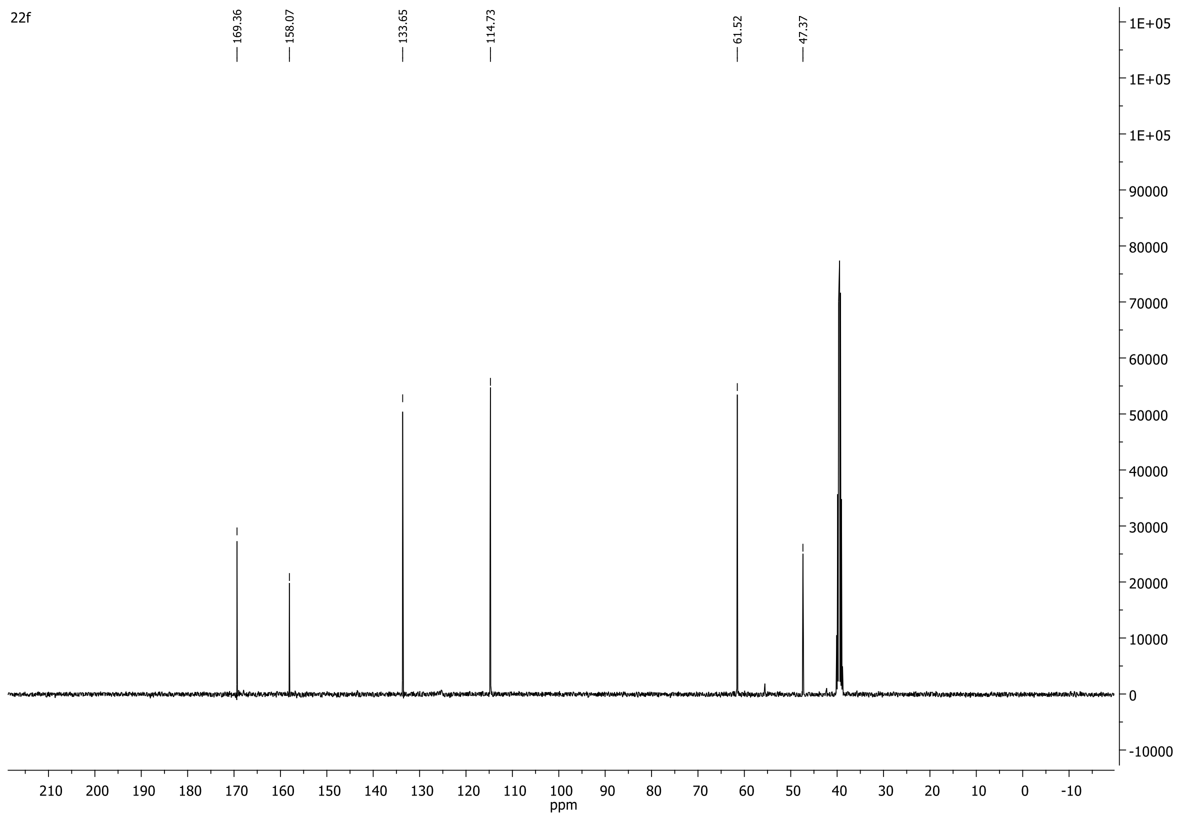

HRMS of 4-hydroxyphenylboronic acid, MIDA ester, **22f**

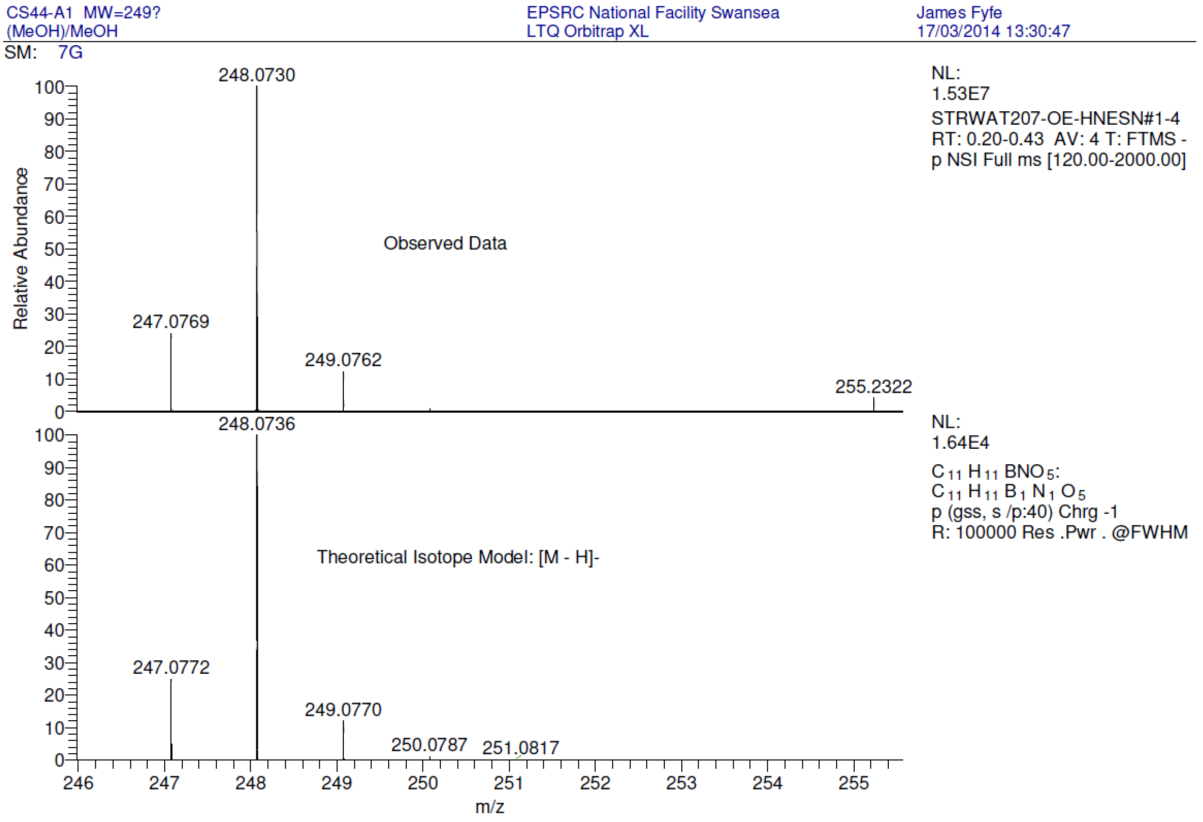

^{1}H NMR of (3-isobutoxyphenyl)boronic acid, MIDA ester, **26f**

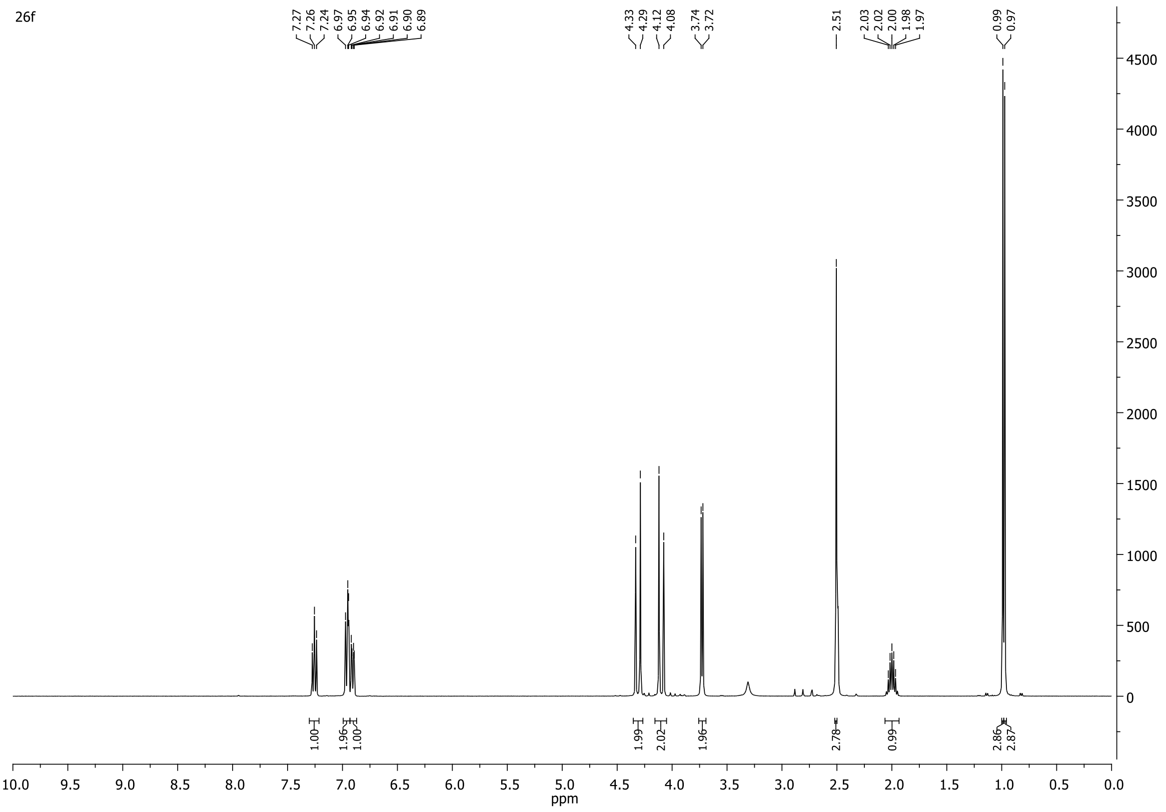

^{13}C NMR of (3-isobutoxyphenyl)boronic acid, MIDA ester, **26f**

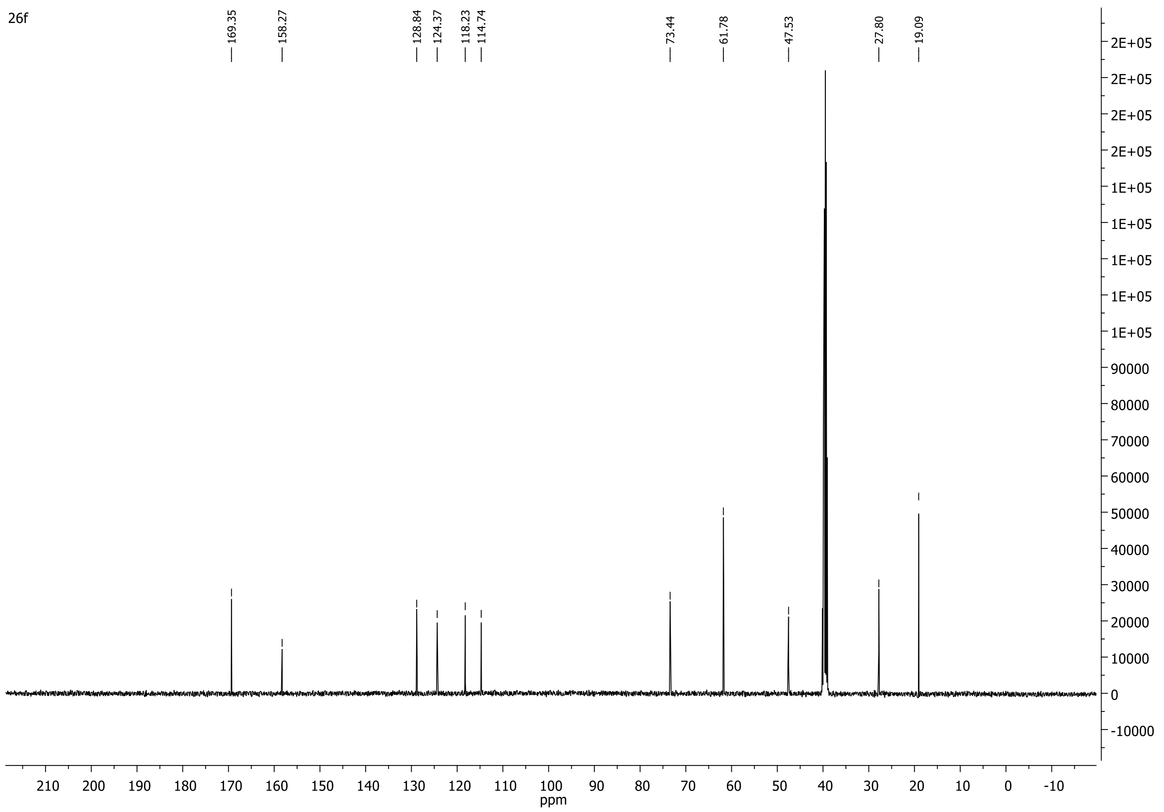

HRMS of (3-isobutoxyphenyl)boronic acid, MIDA ester, **26f**

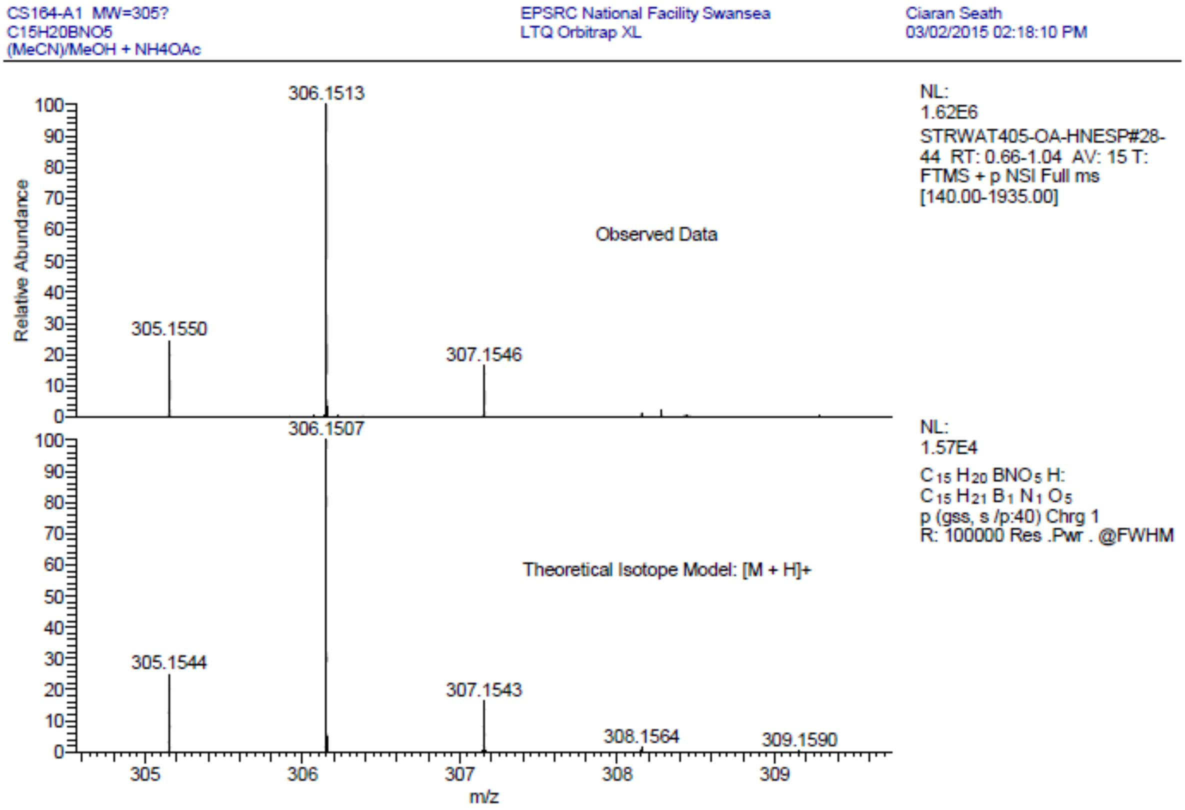

^{1}H NMR of benzene-1-boronic acid, pinacol ester-4-boronic acid, MIDA ester, **27**

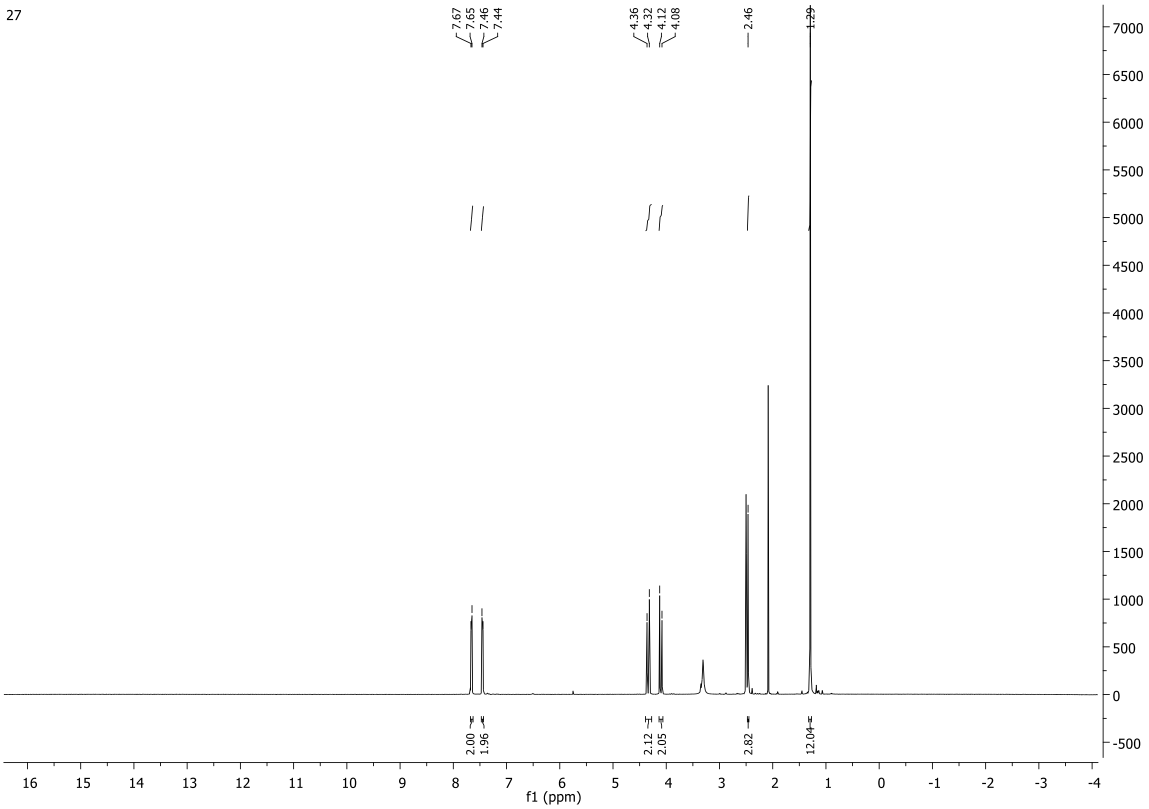

^{13}C NMR of benzene-1-boronic acid, pinacol ester-4-boronic acid, MIDA ester, **27**

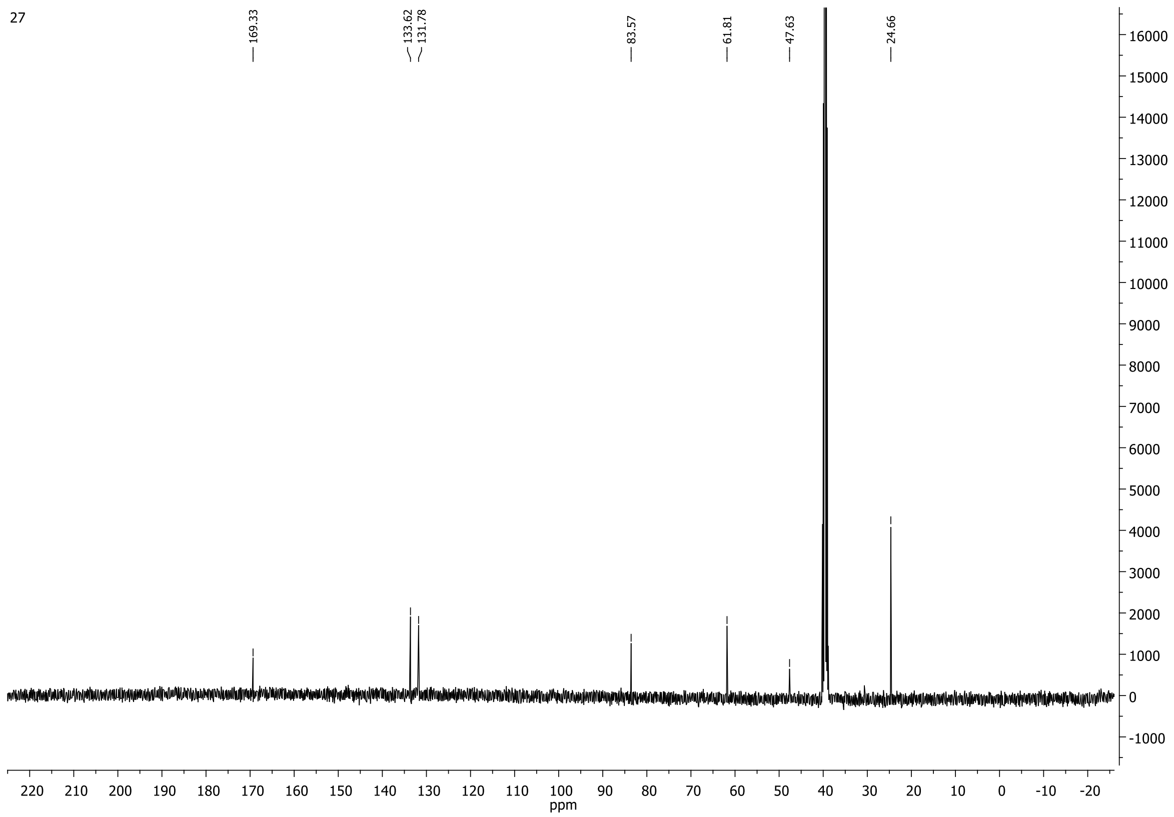

HRMS of benzene-1-boronic acid, pinacol ester-4-boronic acid, MIDA ester, **27**

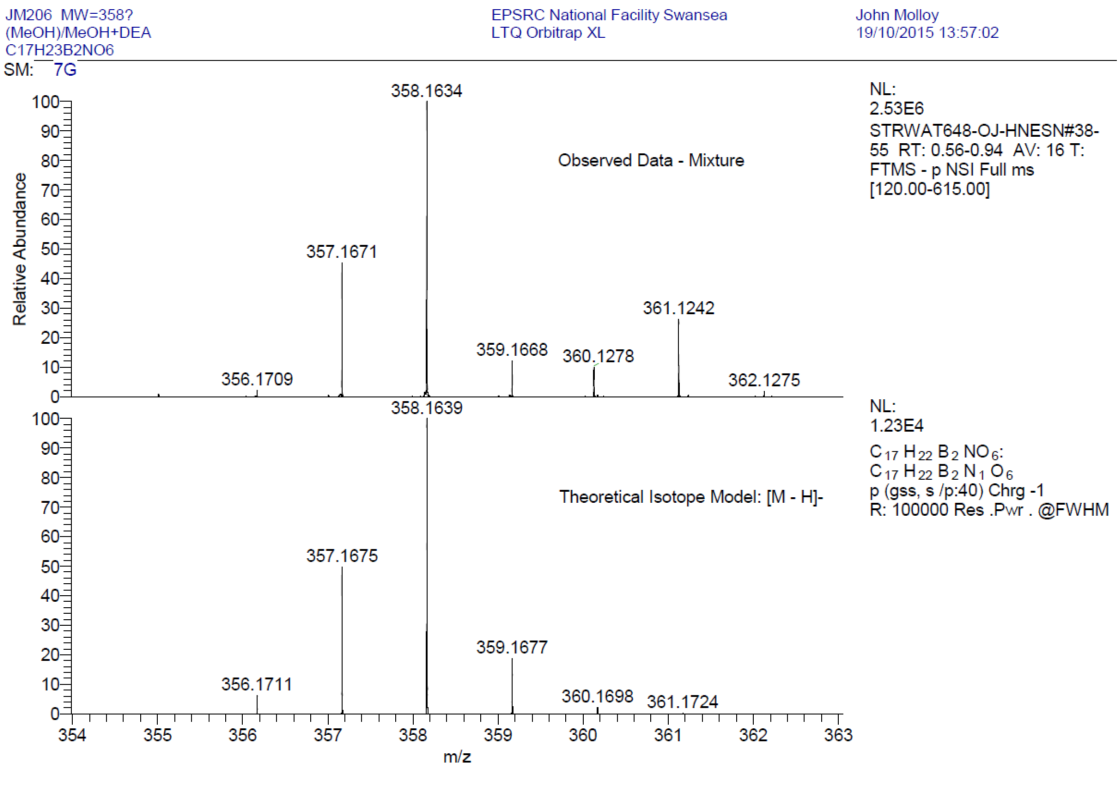

Spectra for Assays

^{1}H NMR of naphthalen-2-ylboronic acid, pinacol ester, **1b**

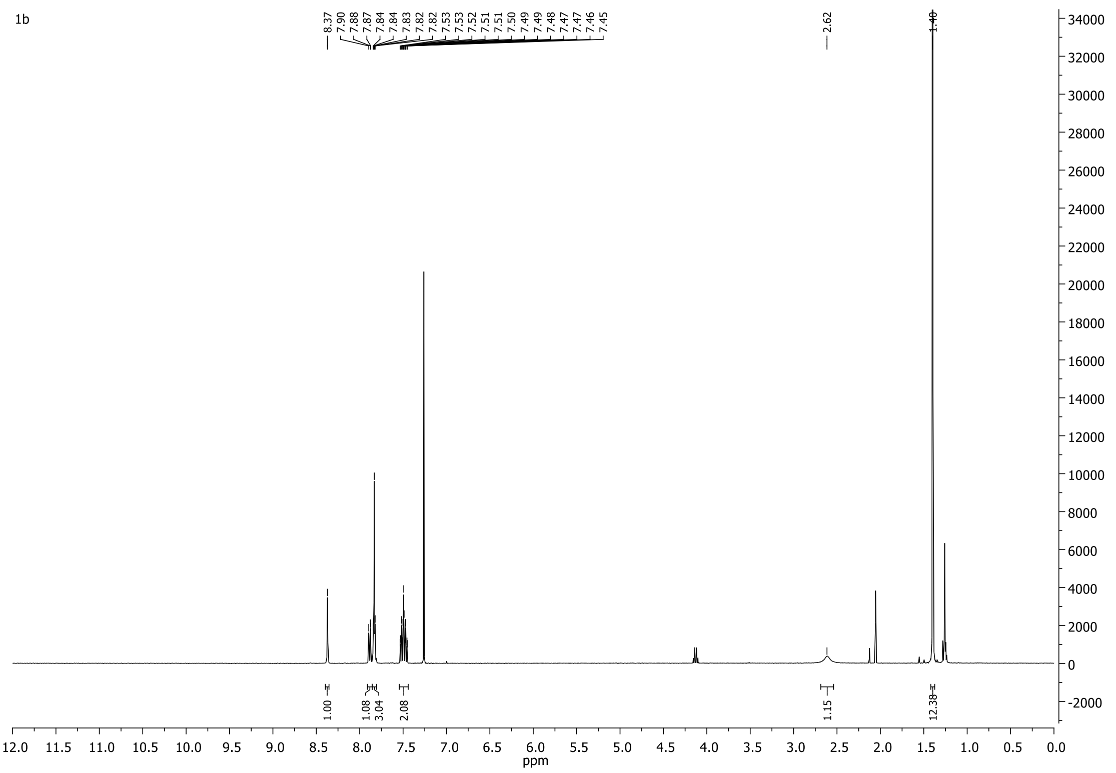

^{1}H NMR of naphthalen-2-ol, **1c**

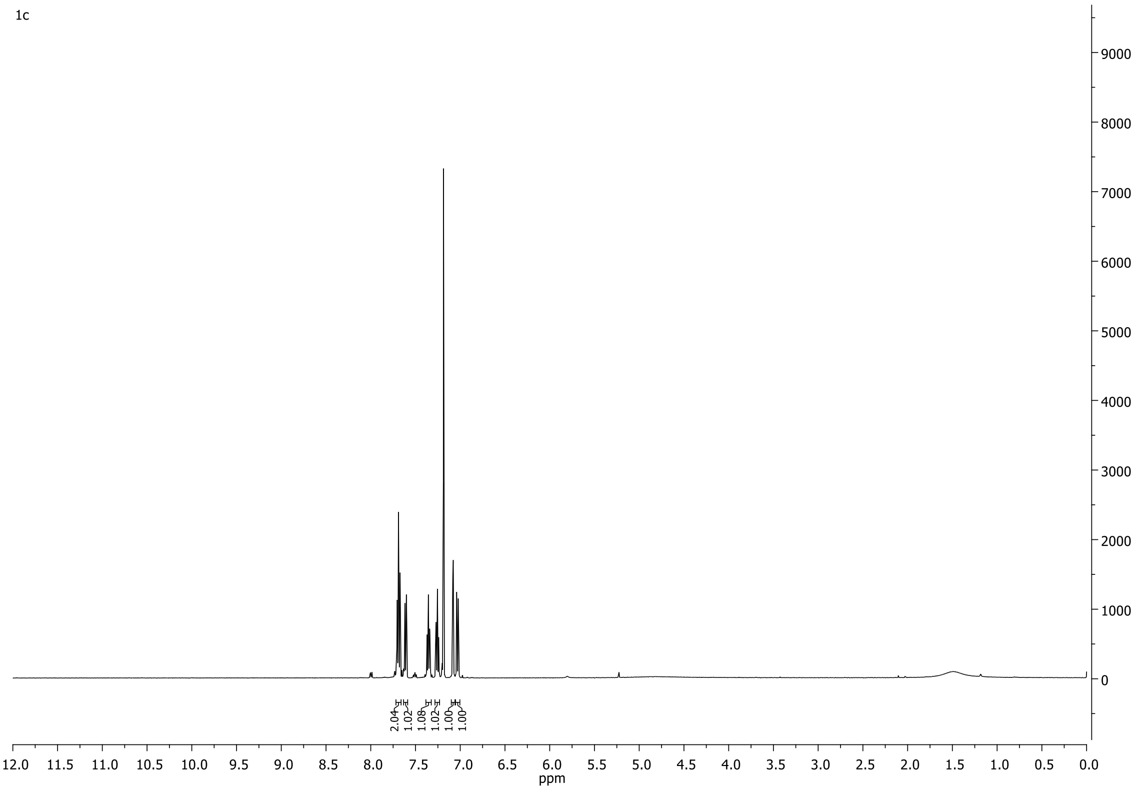

^{1}H NMR of [1,1'-biphenyl]-4-ylboronic acid, **2a**

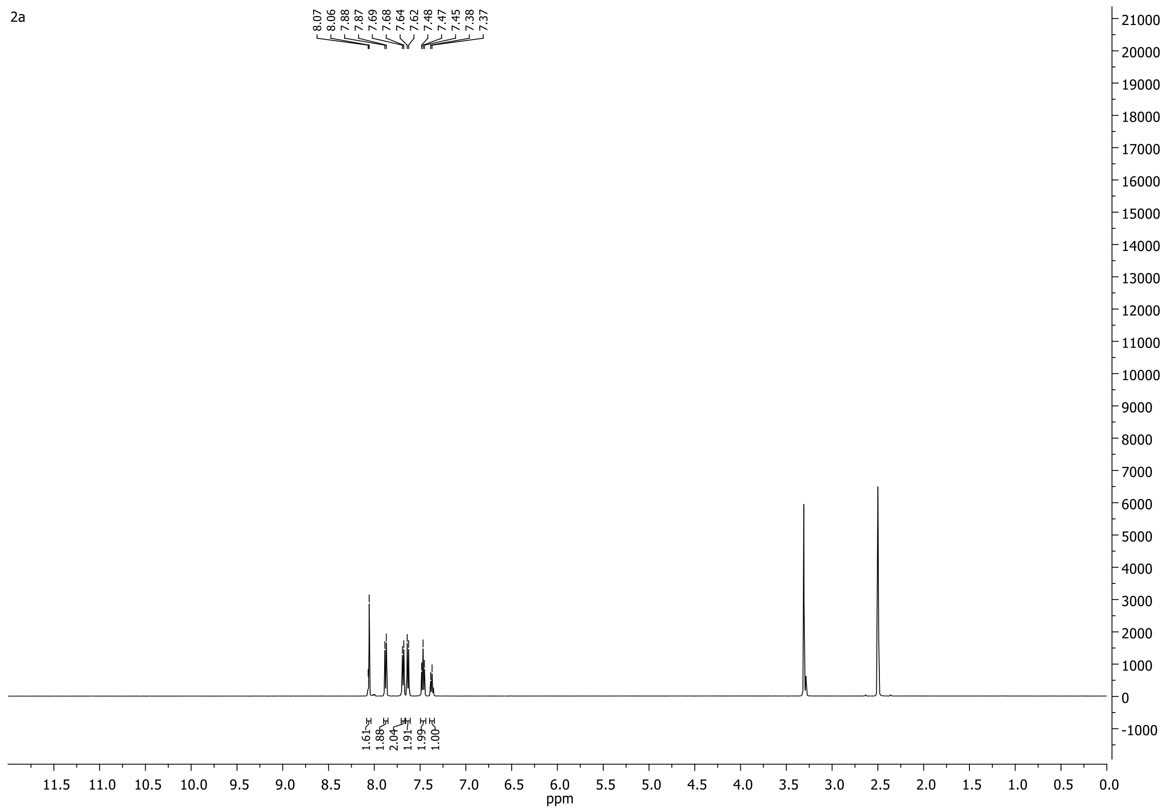

^{1}H NMR of [1,1'-biphenyl]-4-ylboronic acid, pinacol ester, **2b**

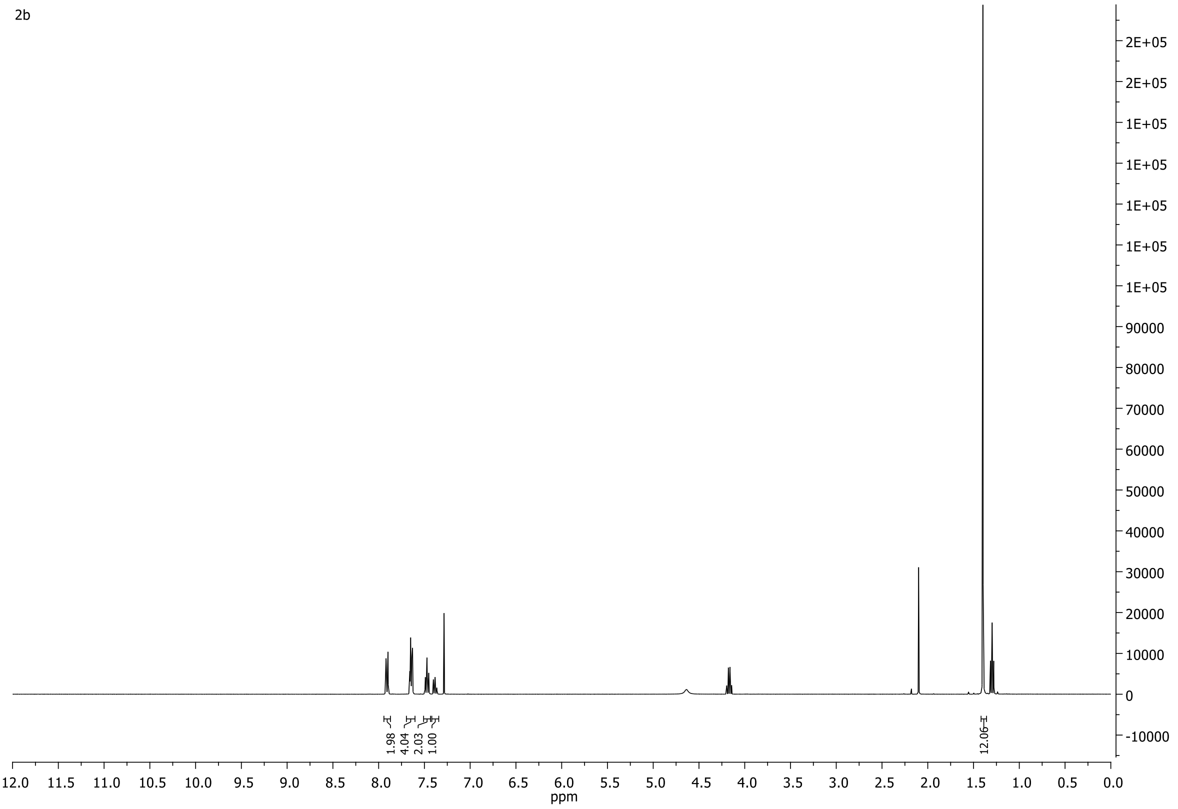

^{1}H NMR of [1,1'-biphenyl]-4-ol, **2c**

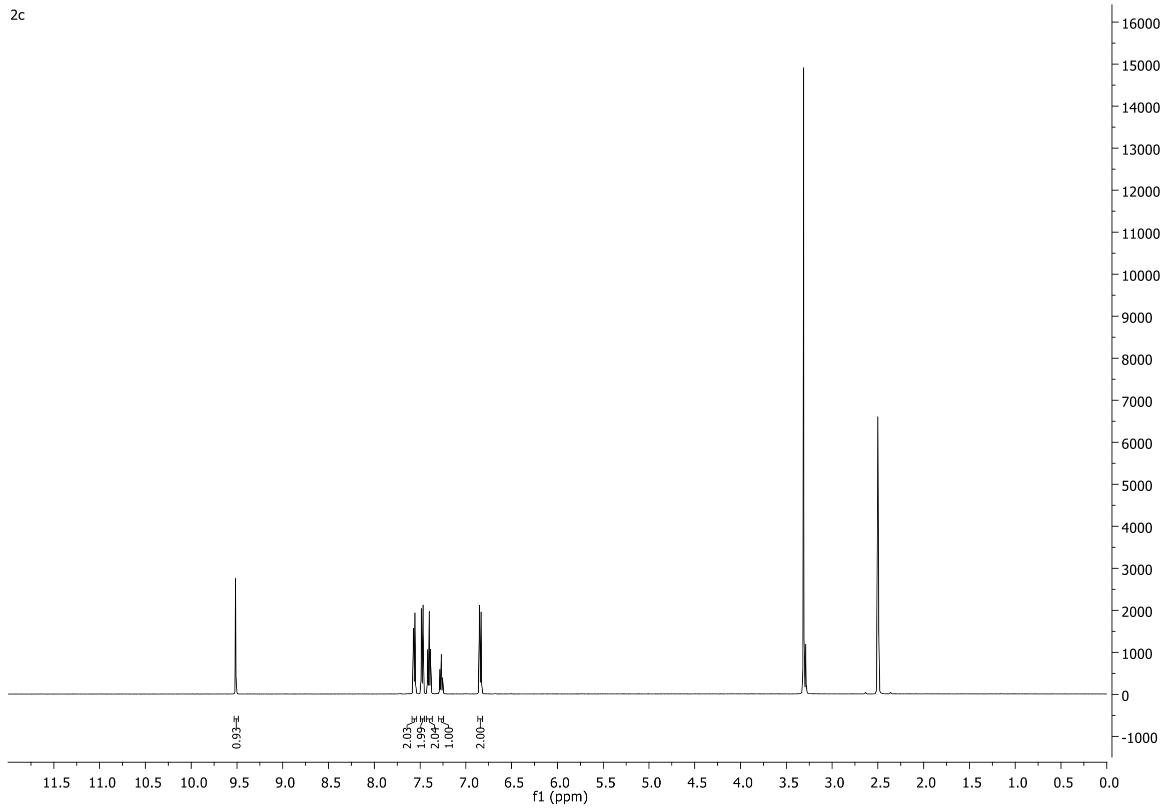

^{1}H NMR of 4-fluorophenol, **3c**

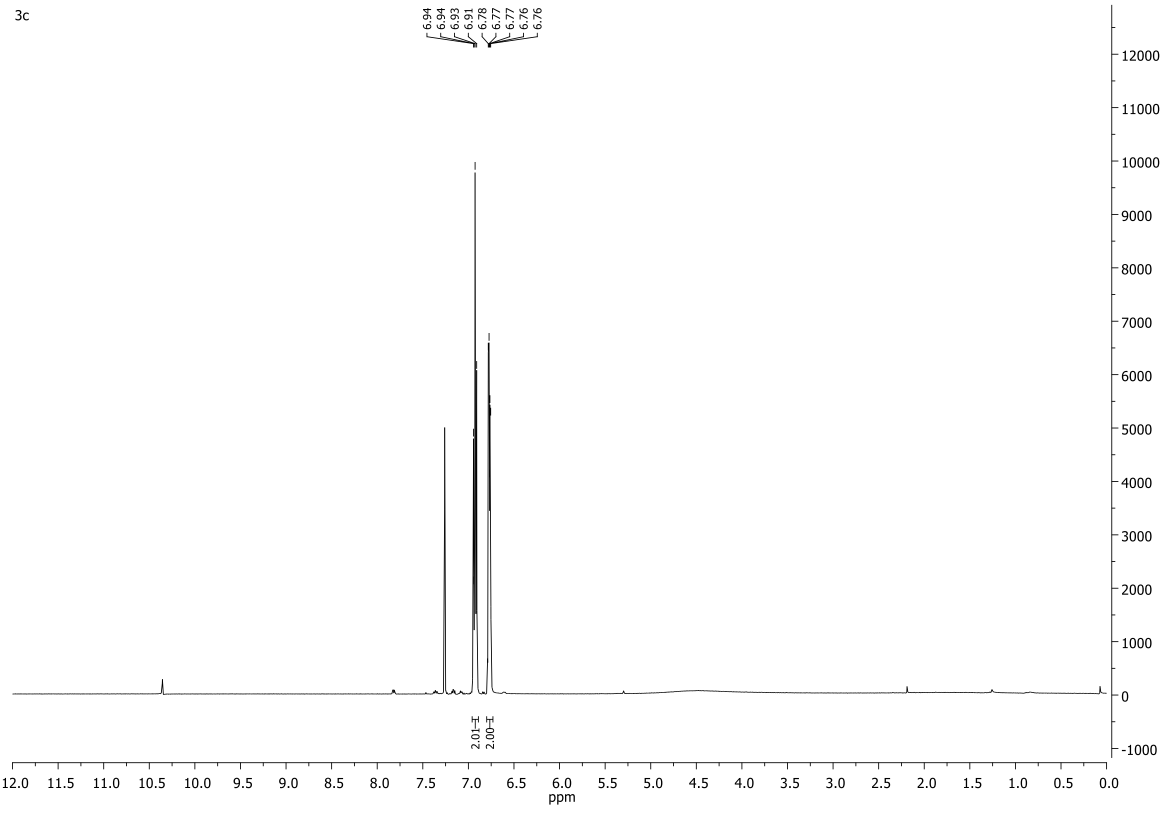

^{1}H NMR of phenol, **4c**

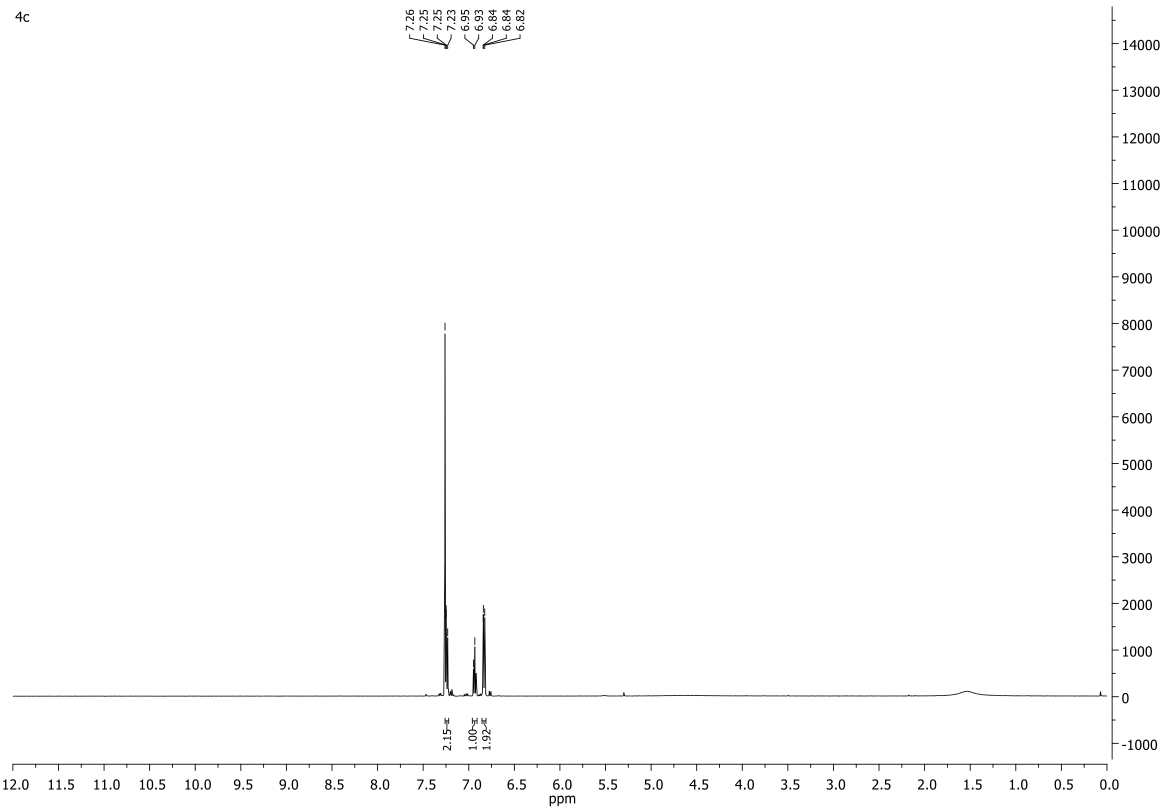

^{1}H NMR of 4-methoxyphenol, **5c**

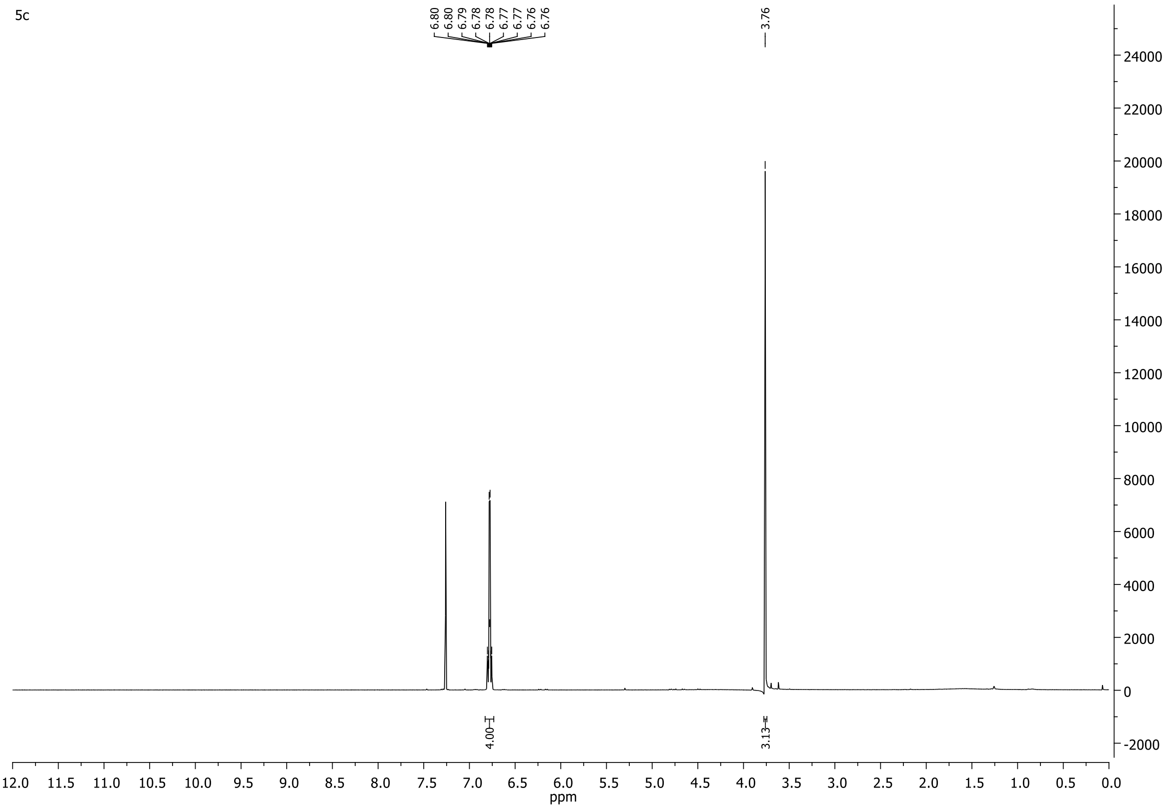

^{1}H NMR of 4-acetamidophenol, **6c**

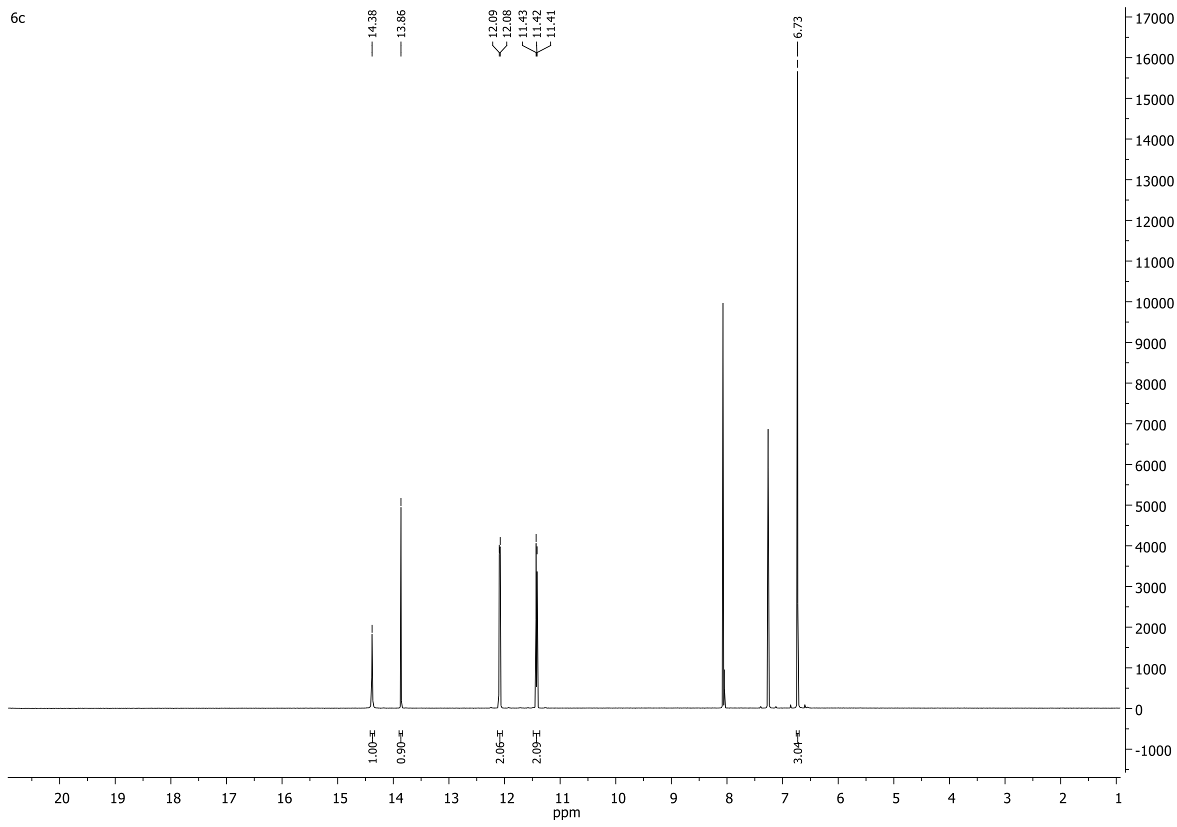

^{1}H NMR of methyl 4-hydroxybenzoate, **7c**

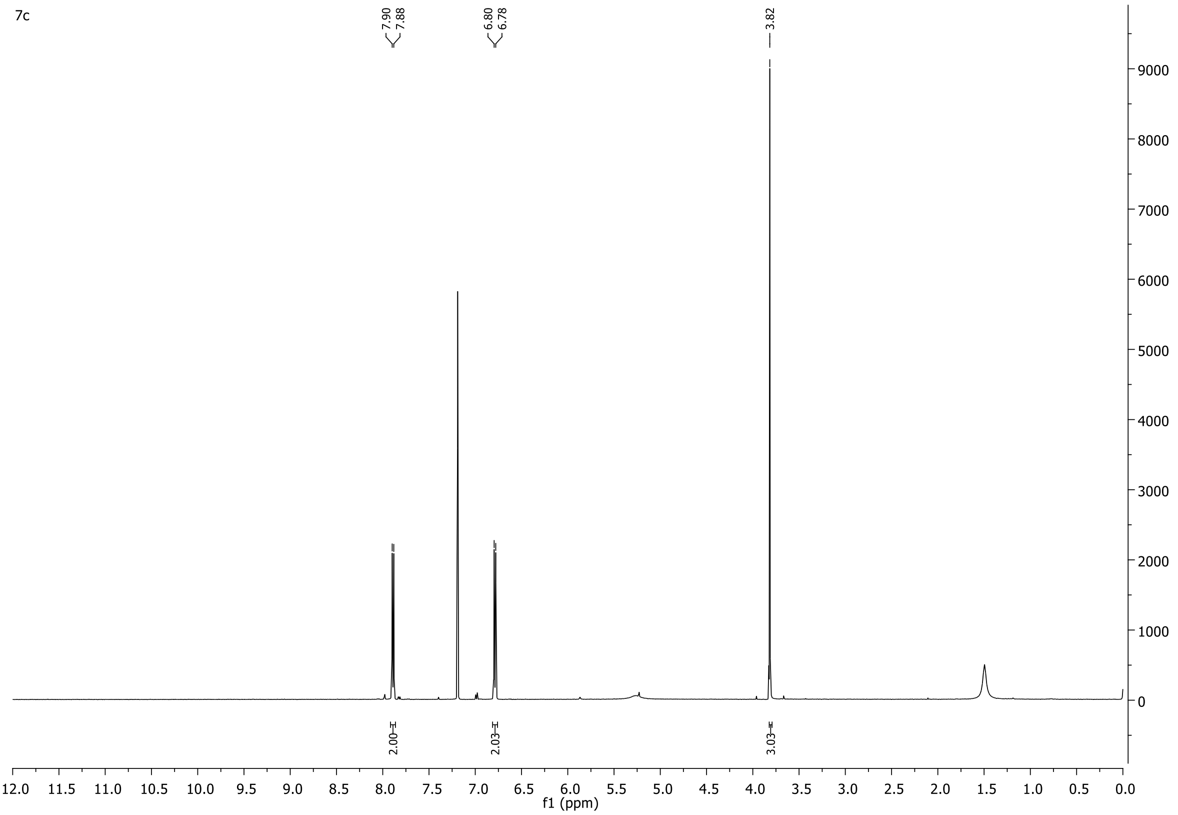

^{1}H NMR of 1*H*-indol-5-ol, **8c**

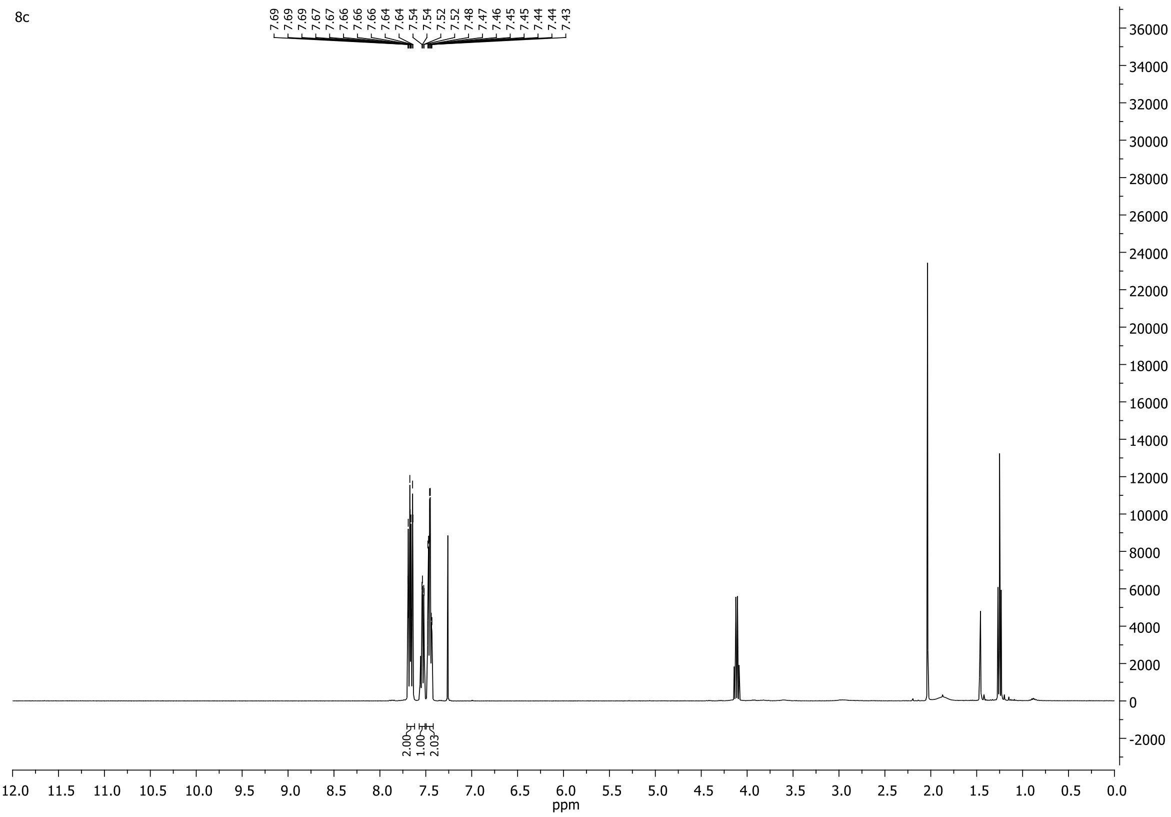

^{1}H NMR of *p*-cresol, **9c**

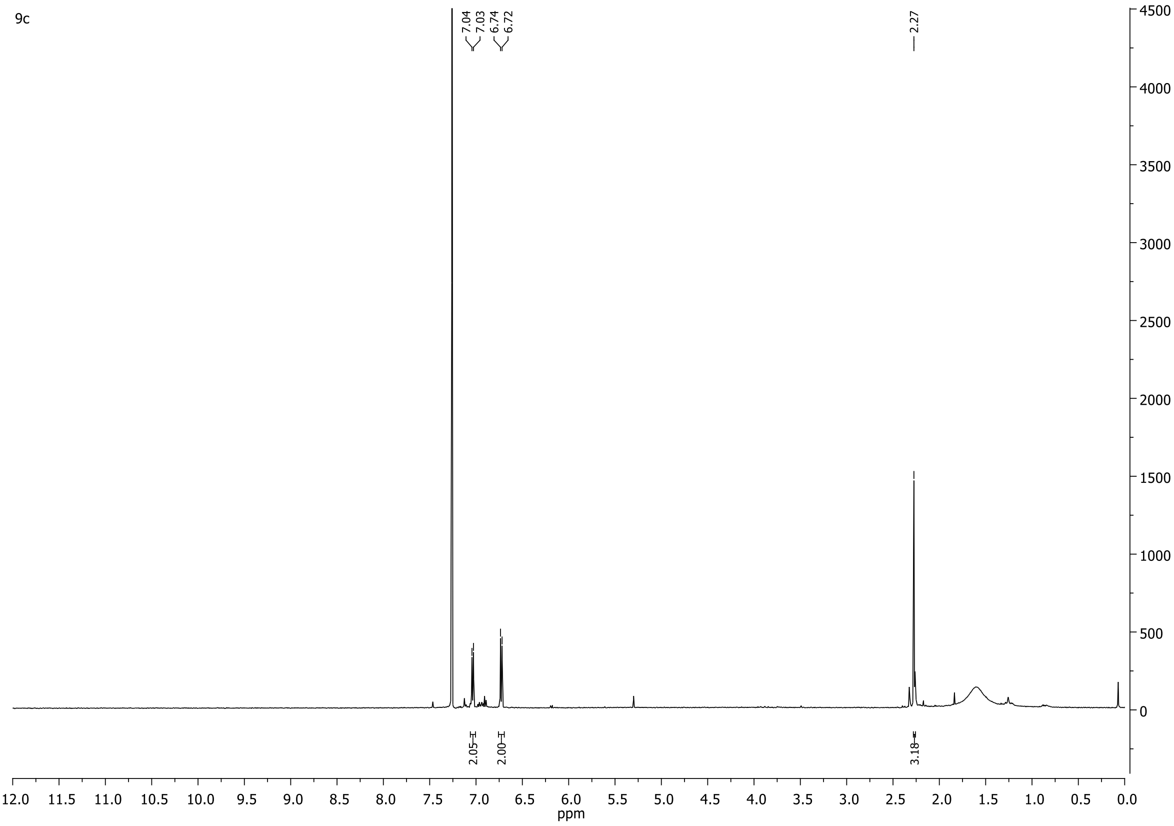

^{1}H NMR of 2-nitrophenol, **10c**

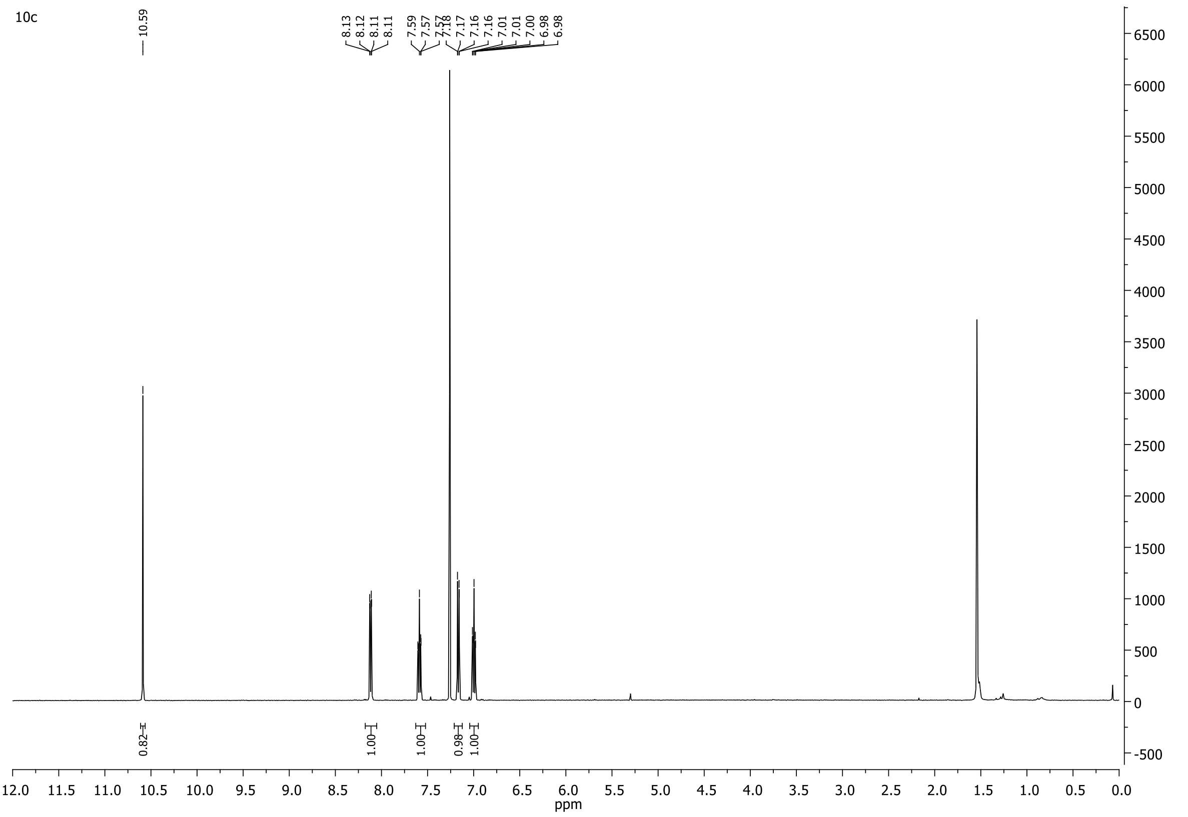

^{1}H NMR of 2,4,6-trimethylphenol, **11c**

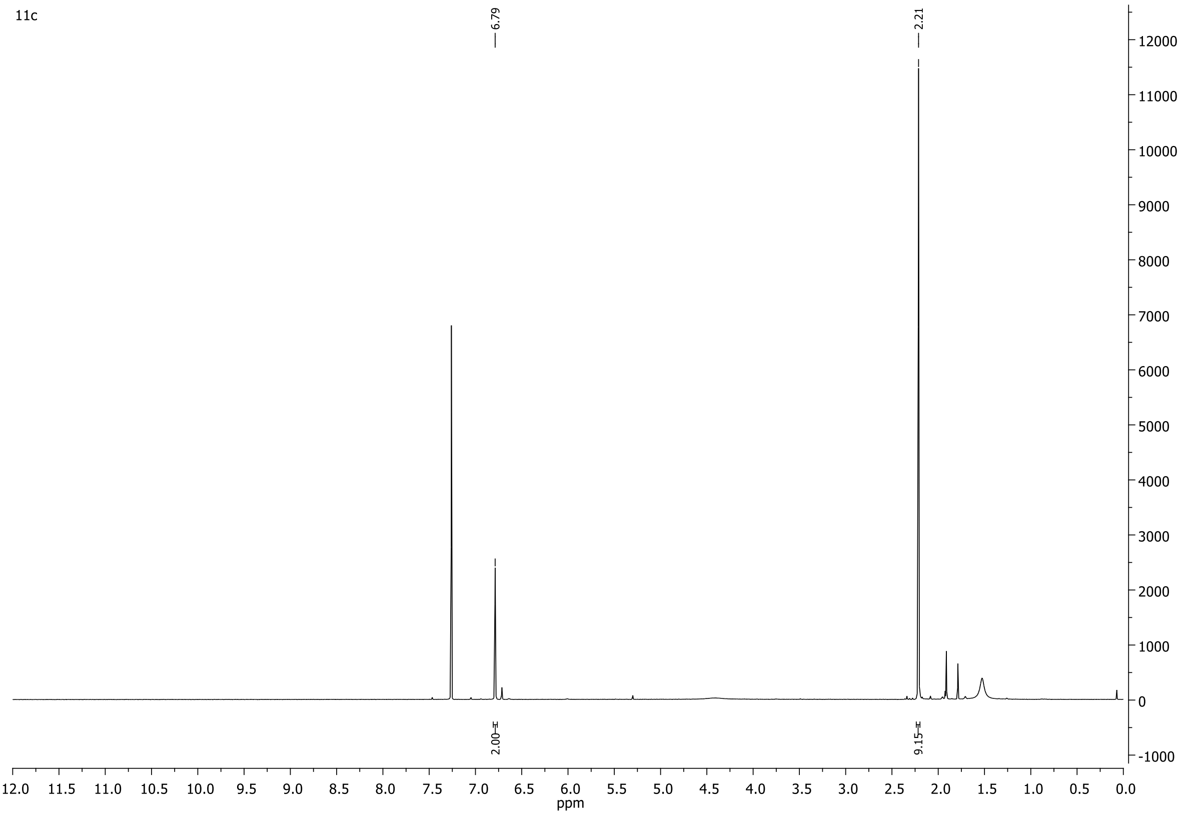

^{1}H NMR of 3-bromophenol, **12c**

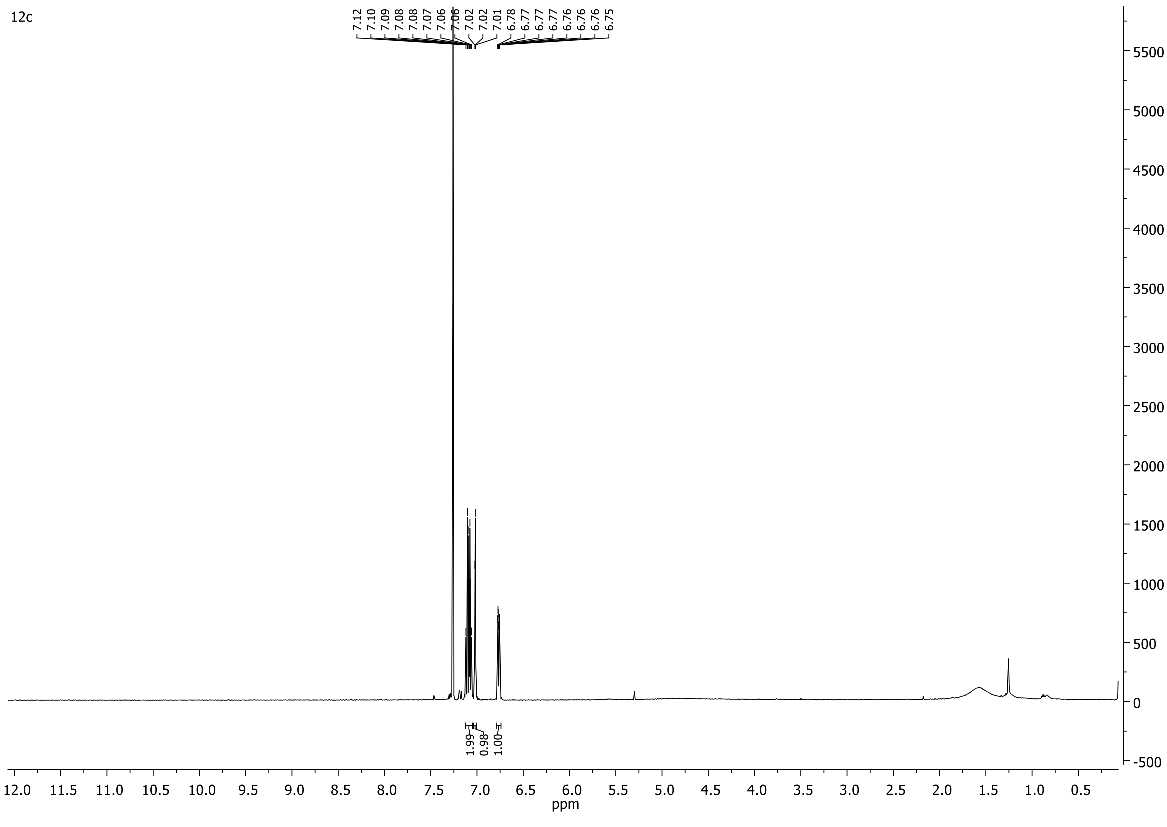

^{1}H NMR of benzofuran-5-ol, **13c**

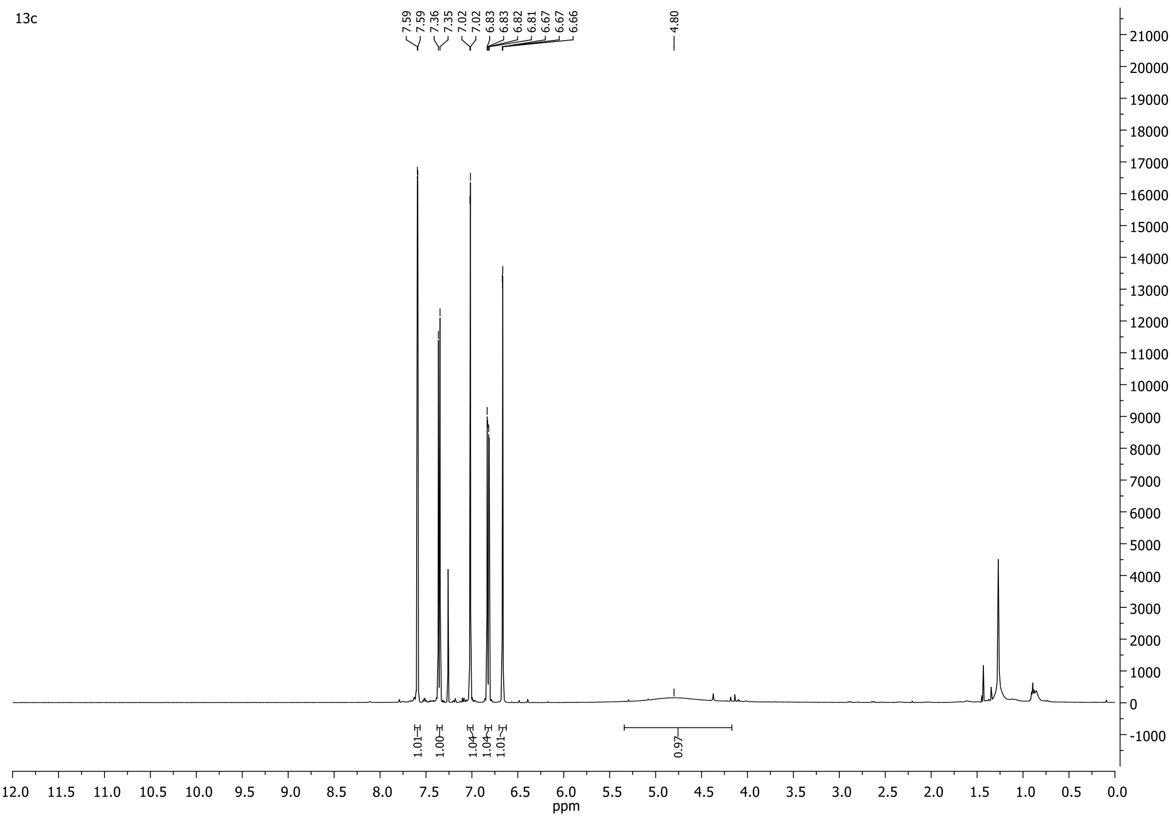

^{1}H NMR of 2-methoxypyridin-3-ol, **14c**

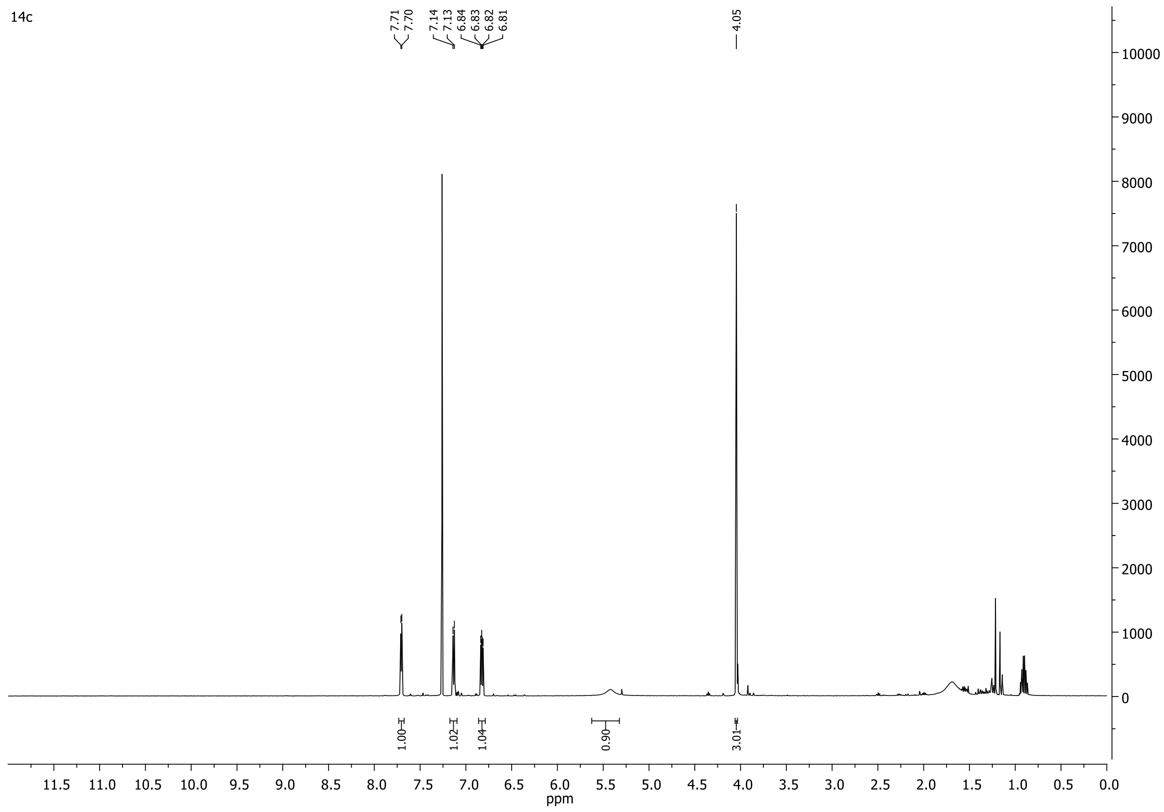

^{1}H NMR of 2,4-difluorophenylboronic acid, pinacol ester, **15b**

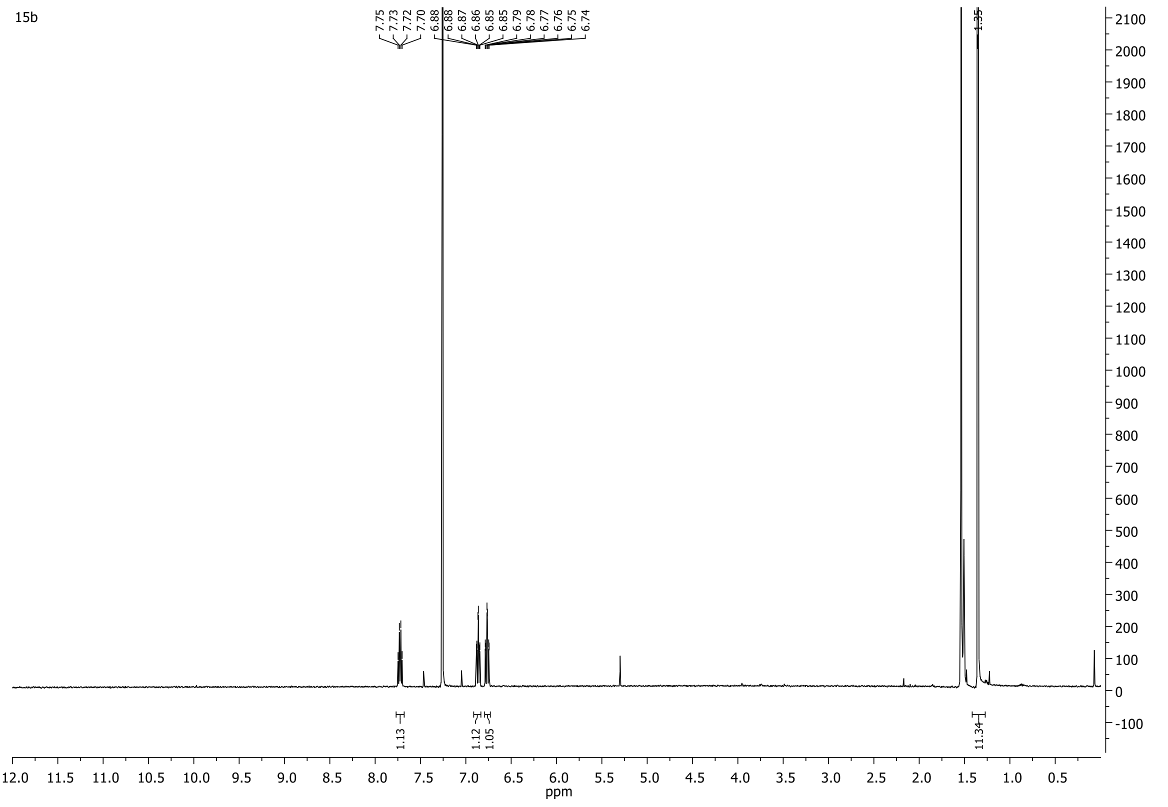

^{1}H NMR of 4-cyanophenylboronic acid, pinacol ester, **16b**

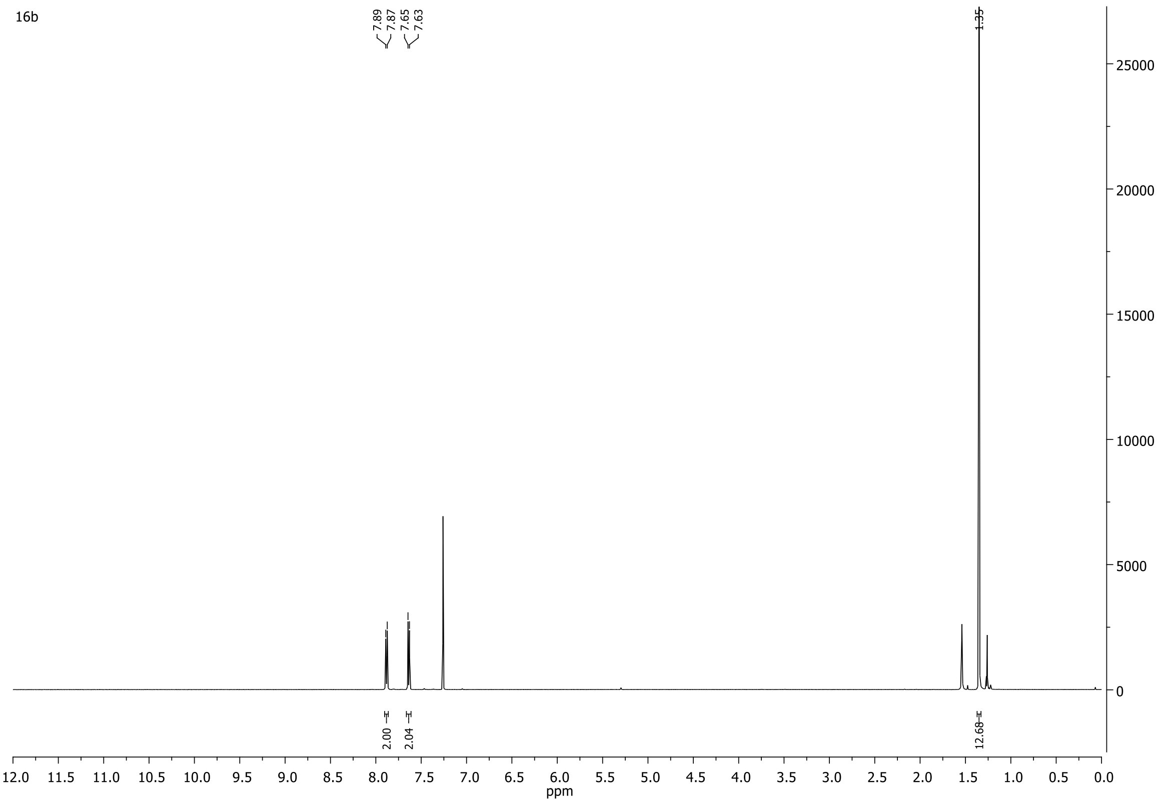

^{1}H NMR of (Benzofuran-2-yl)boronic acid, pinacol ester, **17b**

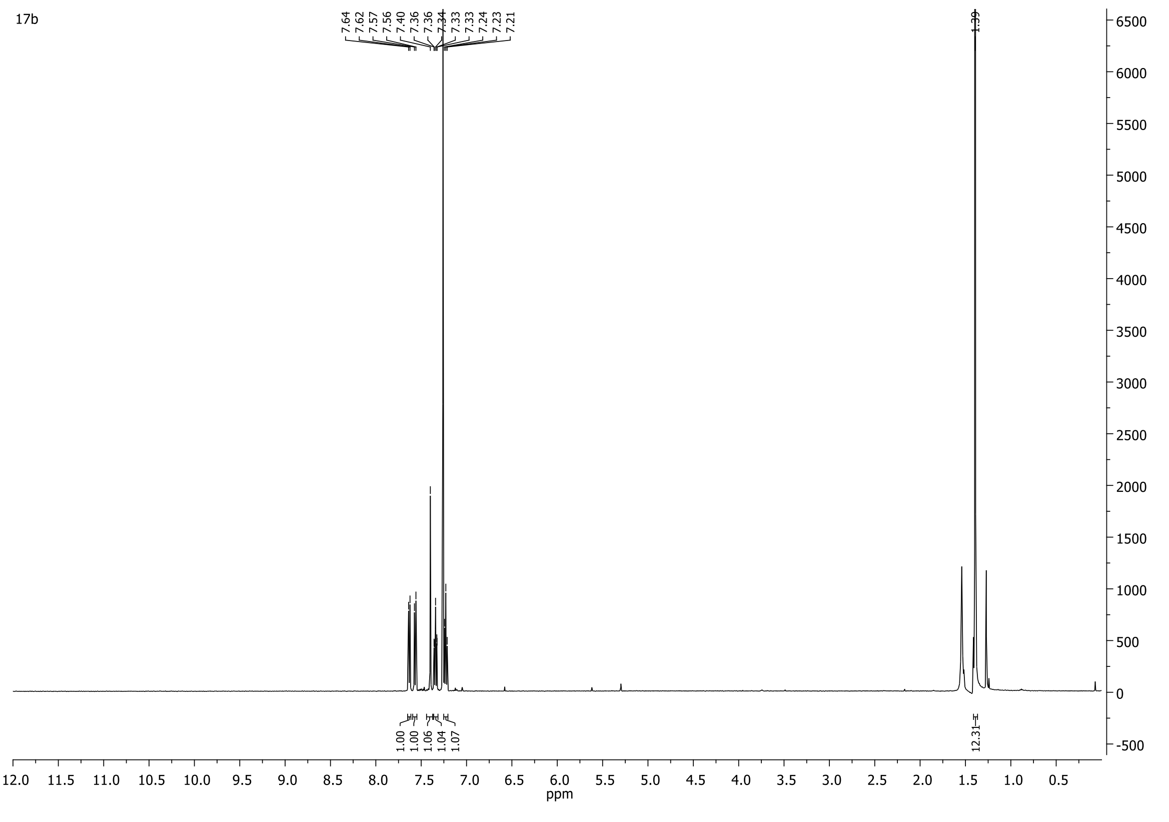

^{1}H NMR of thiophene-2-ylboronic acid, **18a**

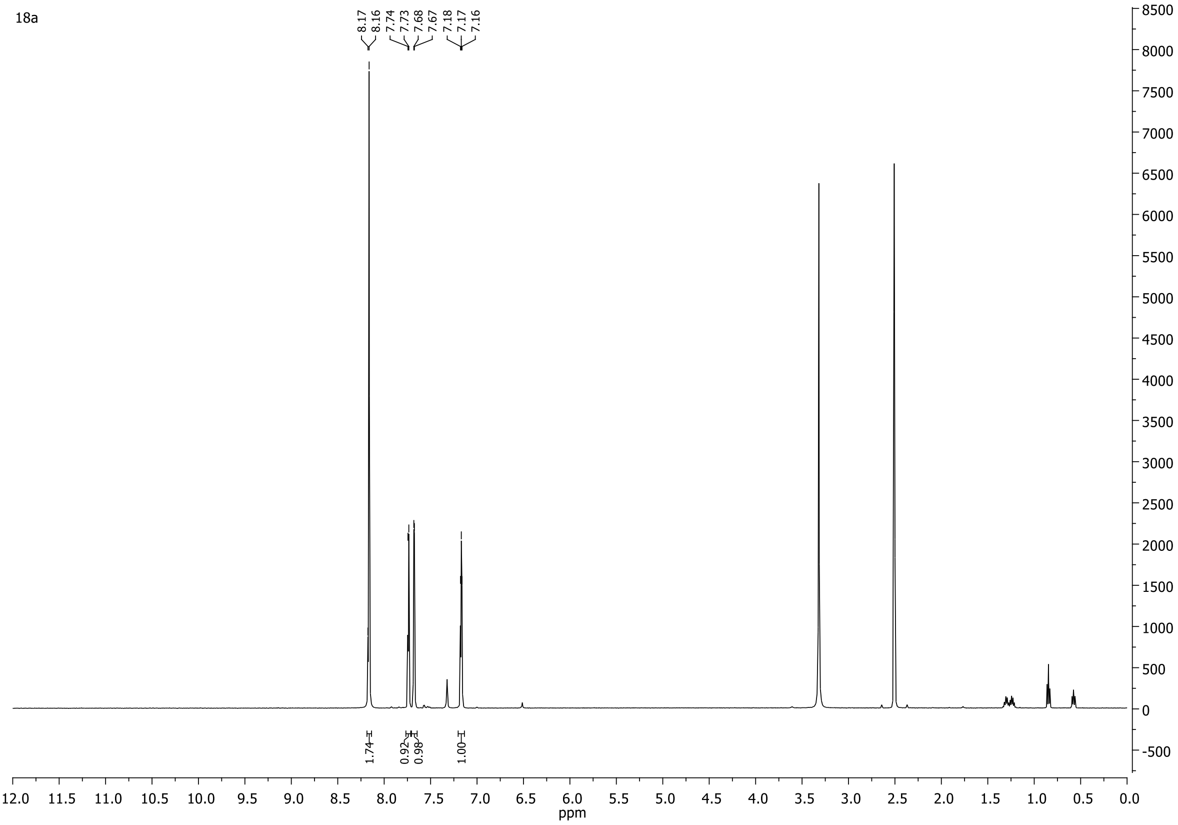

^{1}H NMR of thiophen-2-ylboronic acid, pinacol ester, **18b**

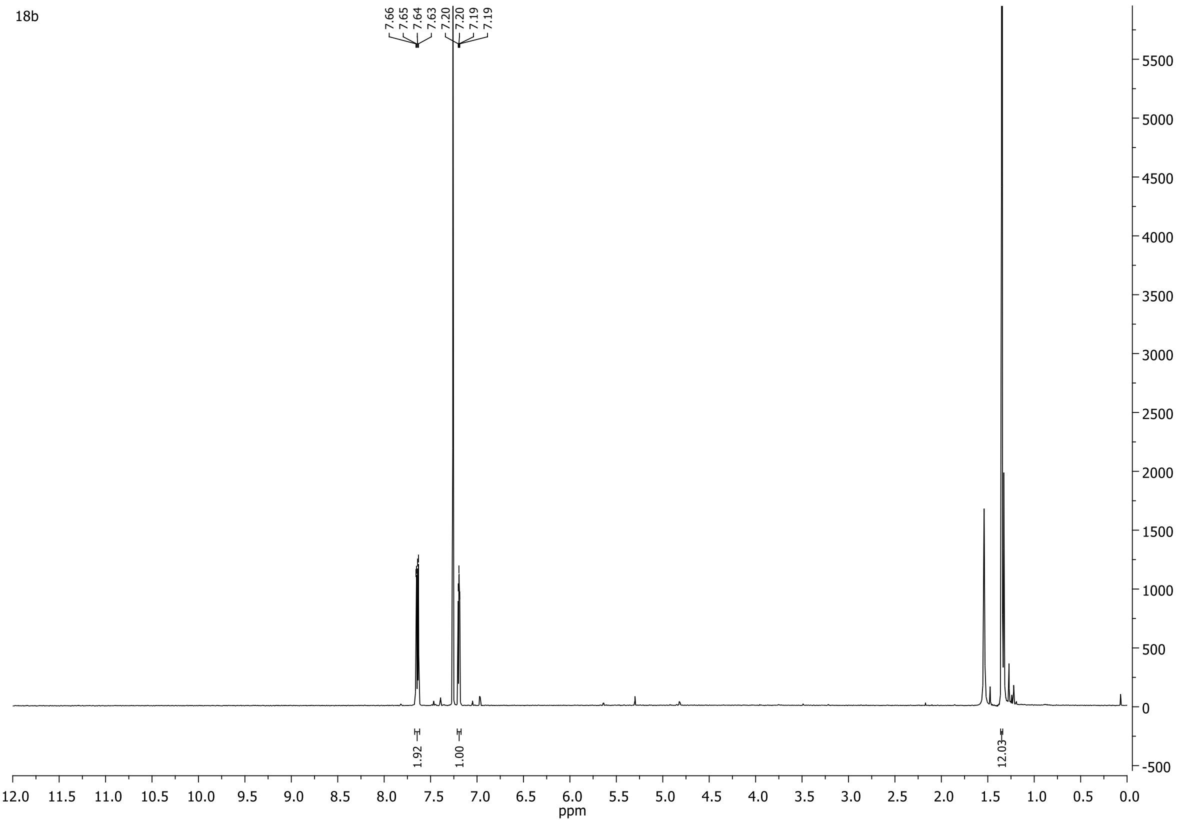

^{1}H NMR of isoquinolin-4-ylboronic acid, pinacol ester, **19b**

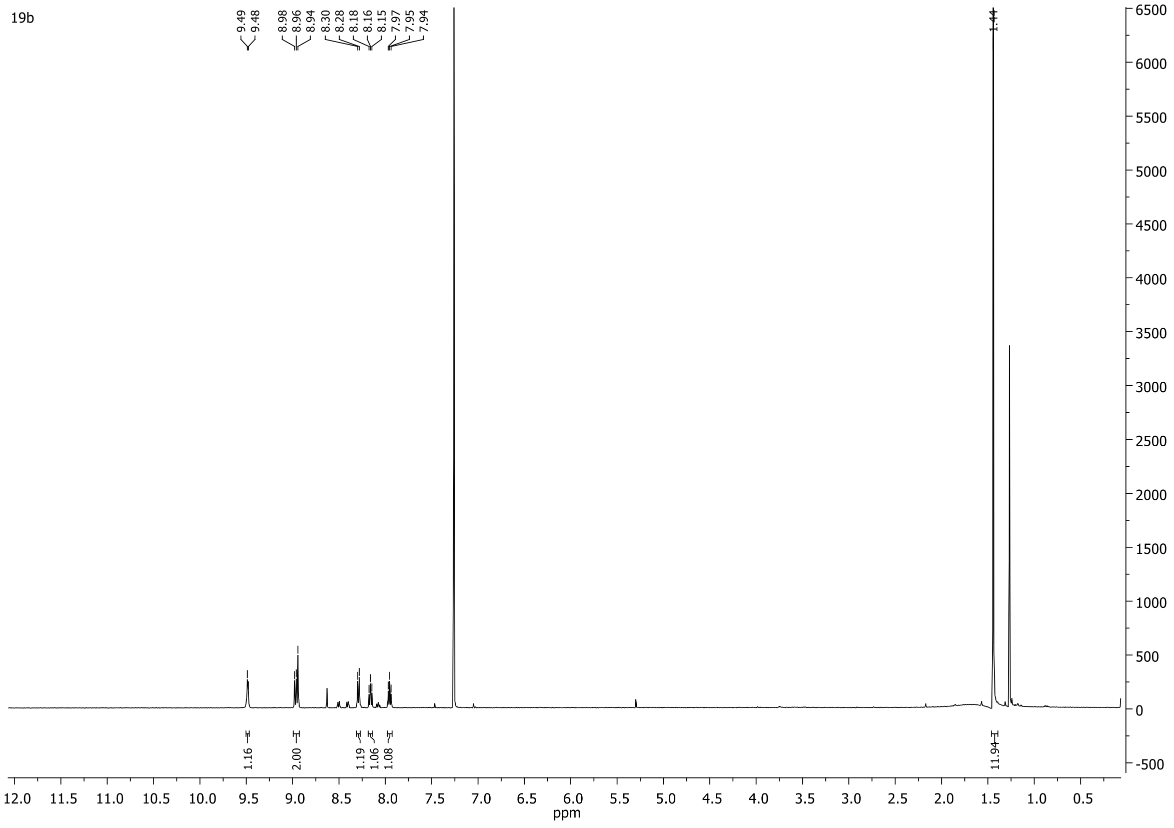

^{1}H NMR of 2-aminophenol, **20c**

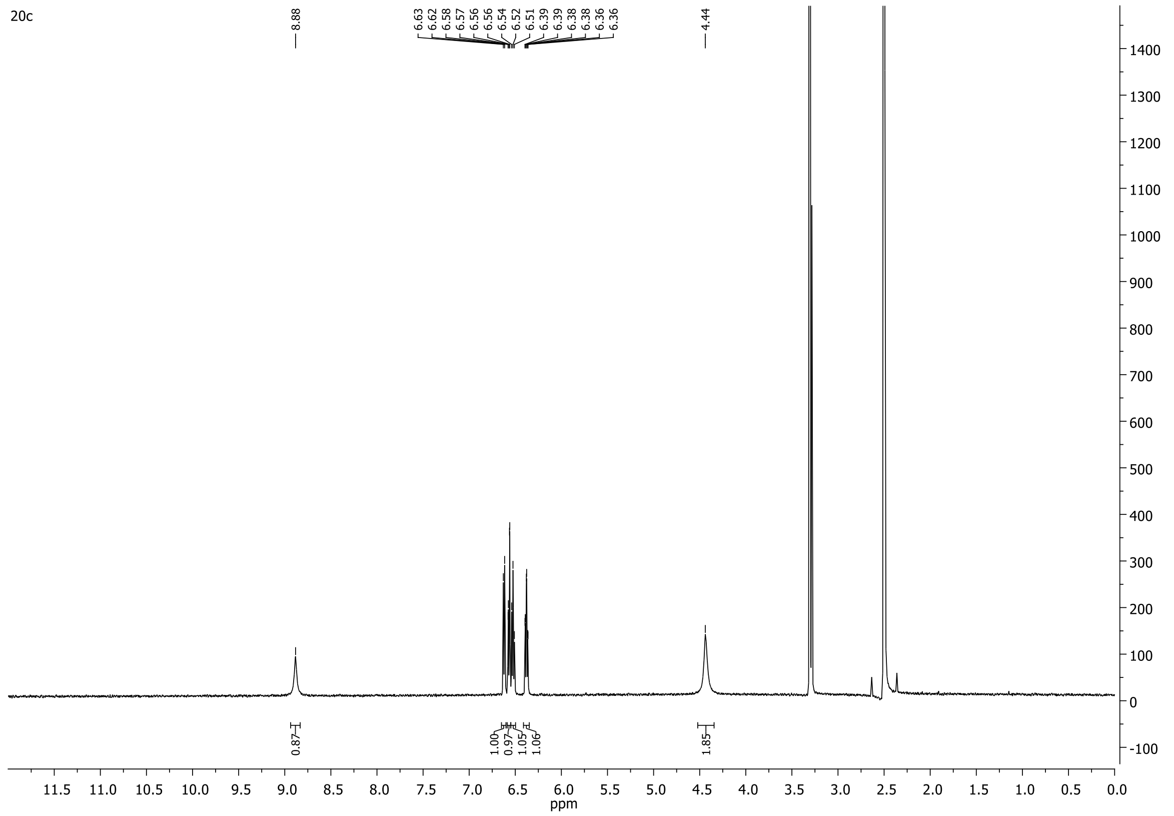

^{1}H NMR of 4-isopropylphenol, **21c**

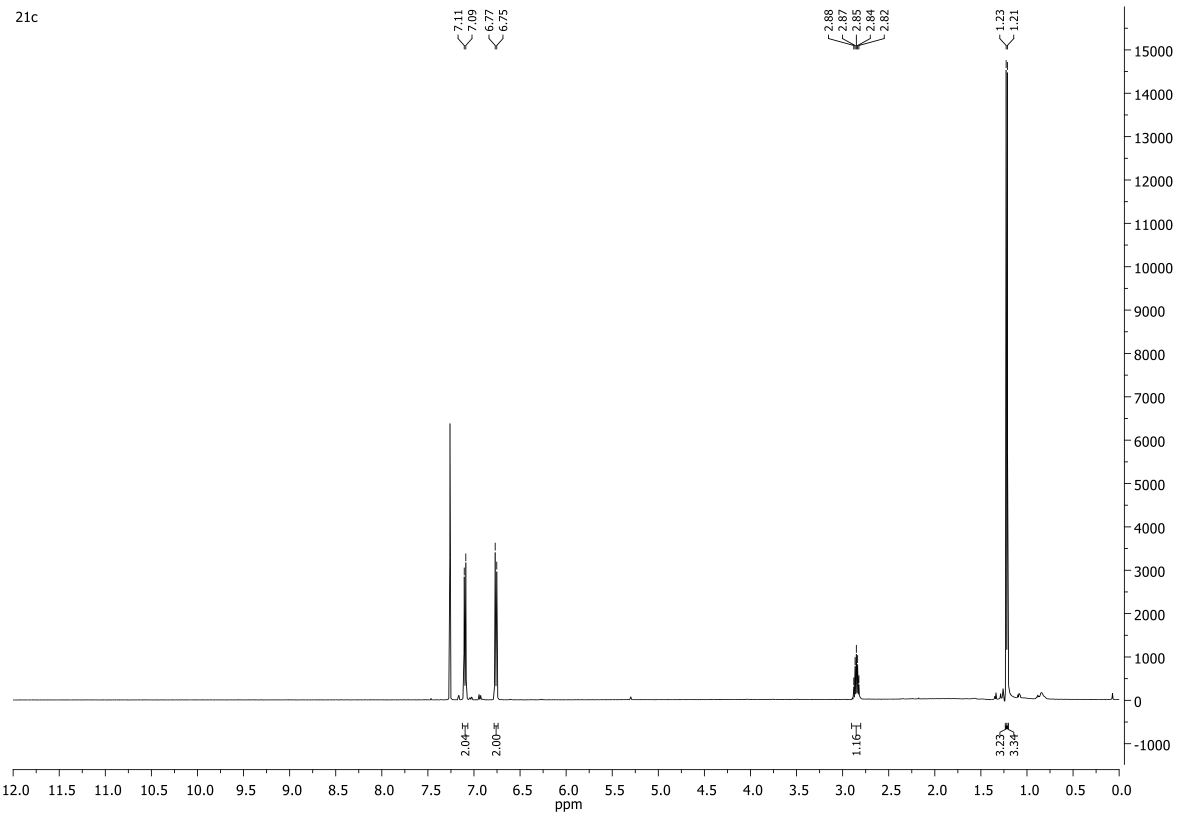

^{1}H NMR of hydroquinone, **22c**

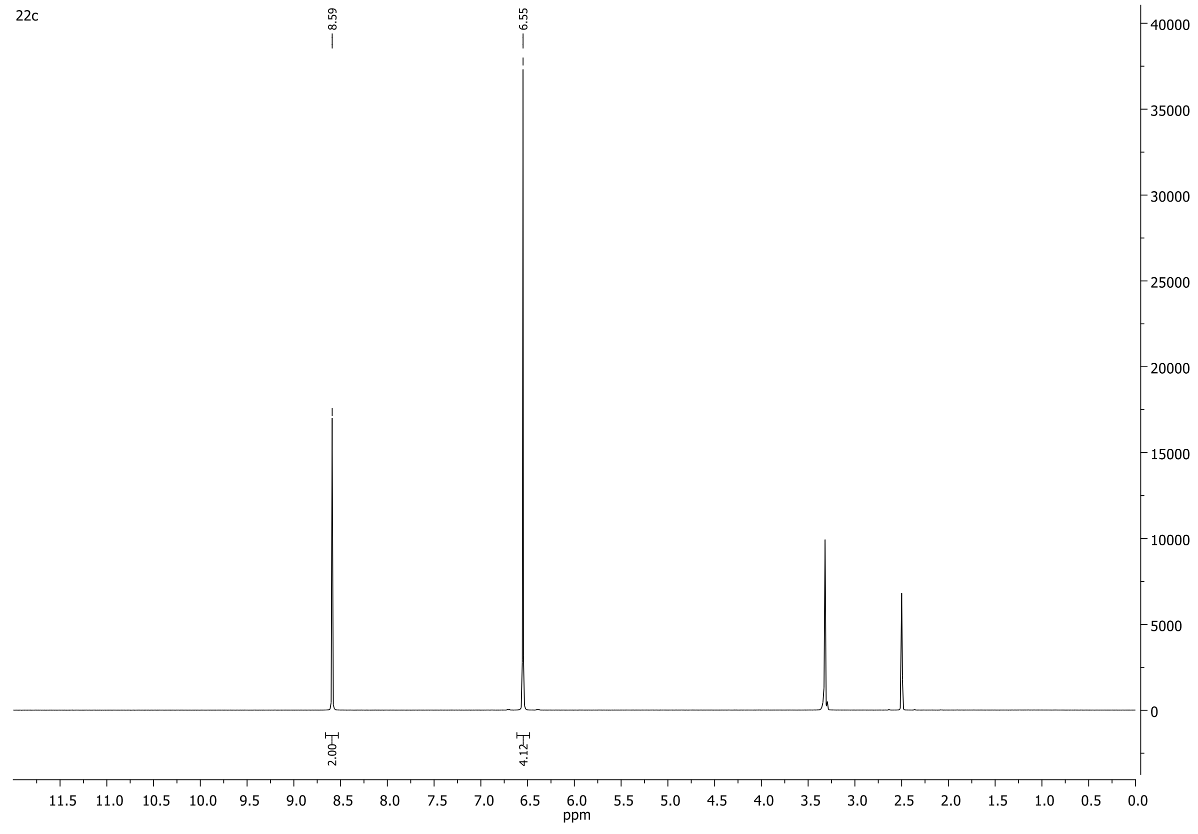

^{1}H NMR of 2-chlorophenol, **23c**

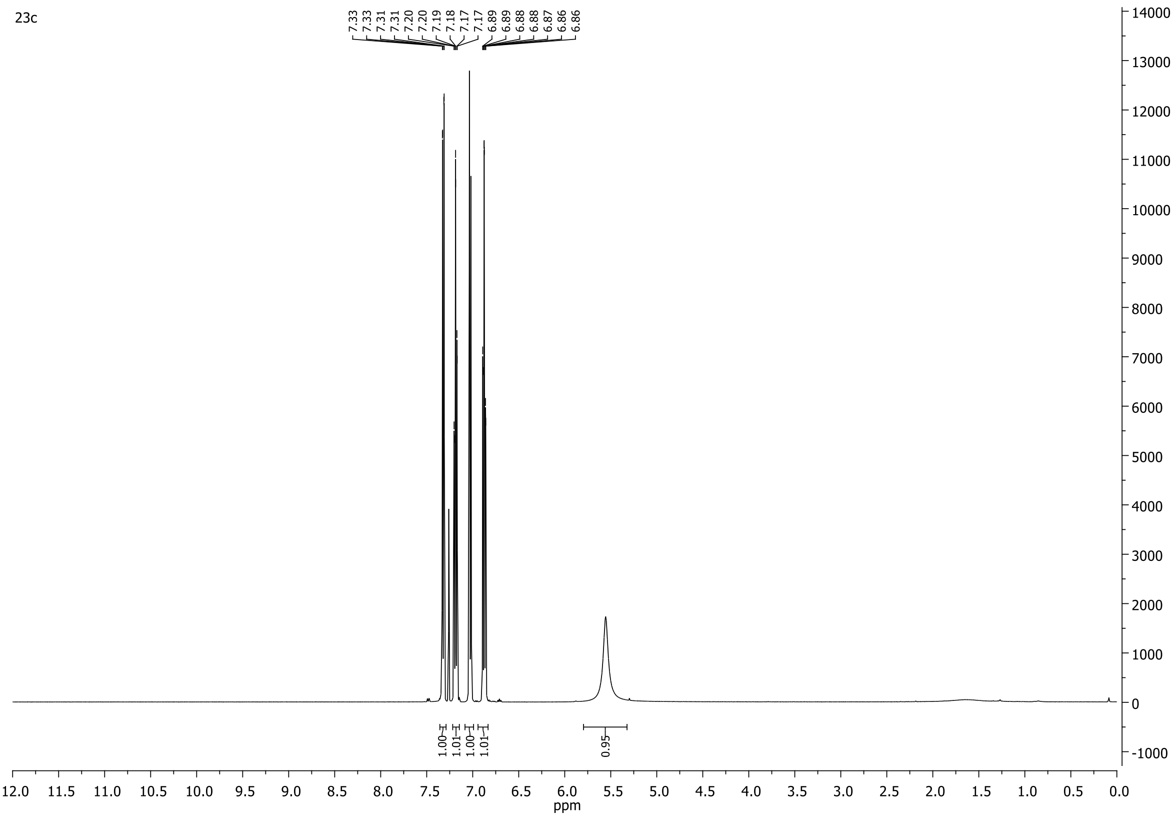

^{1}H NMR of (6-methoxypyridin-3-yl)boronic acid, pinacol ester, **24b**

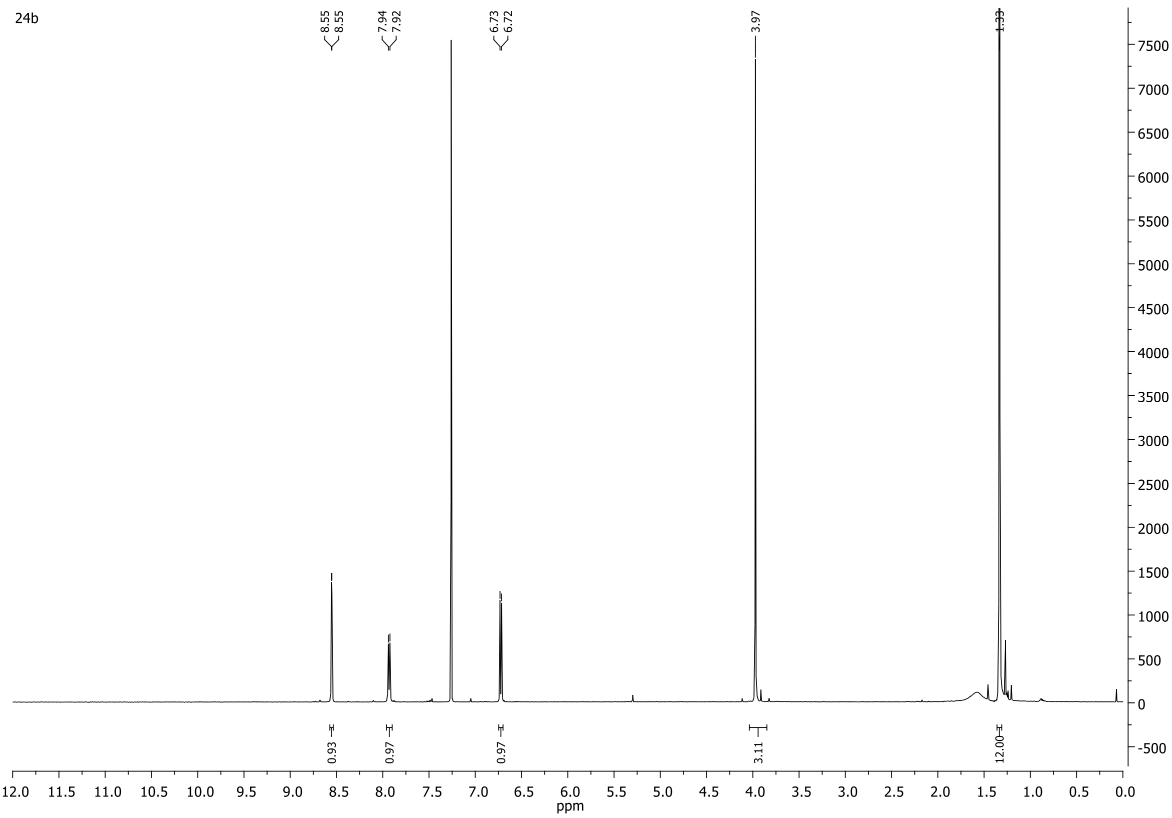

^{1}H NMR of 2-bromophenol, **25c**

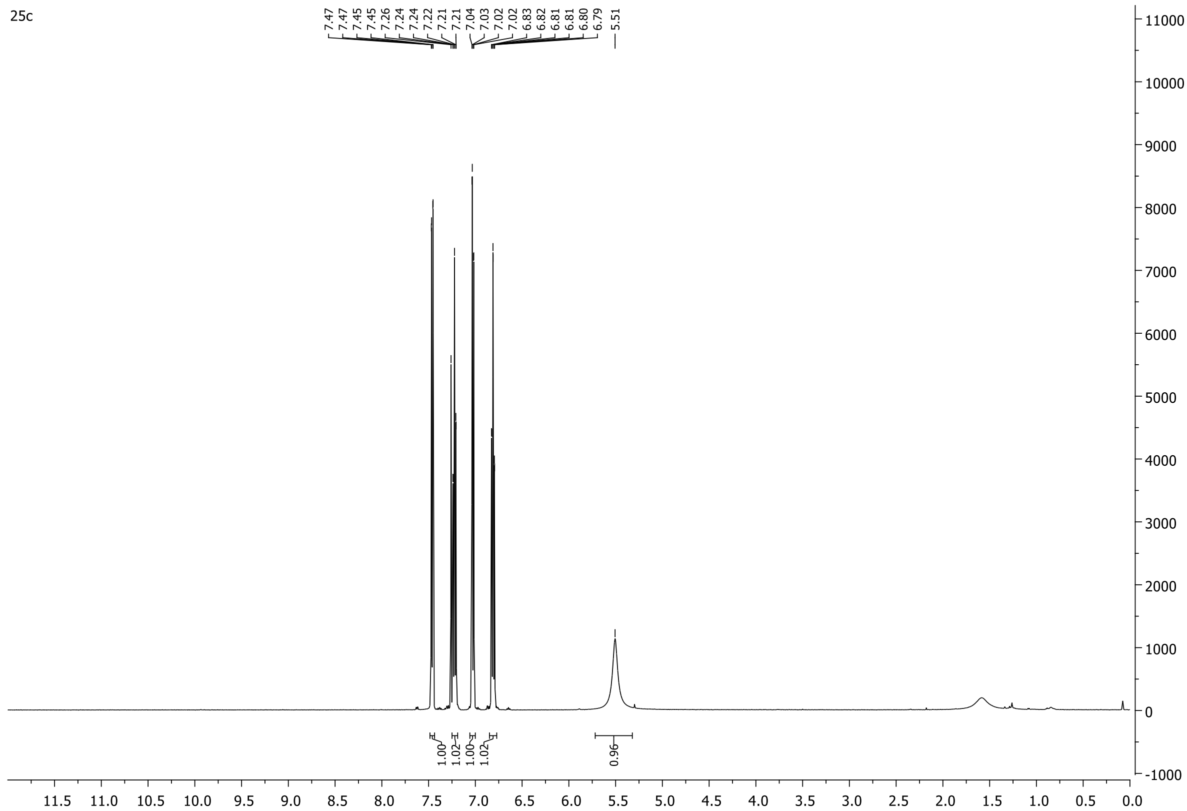

^{1}H NMR of 3-isobutoxyphenol, **26c**

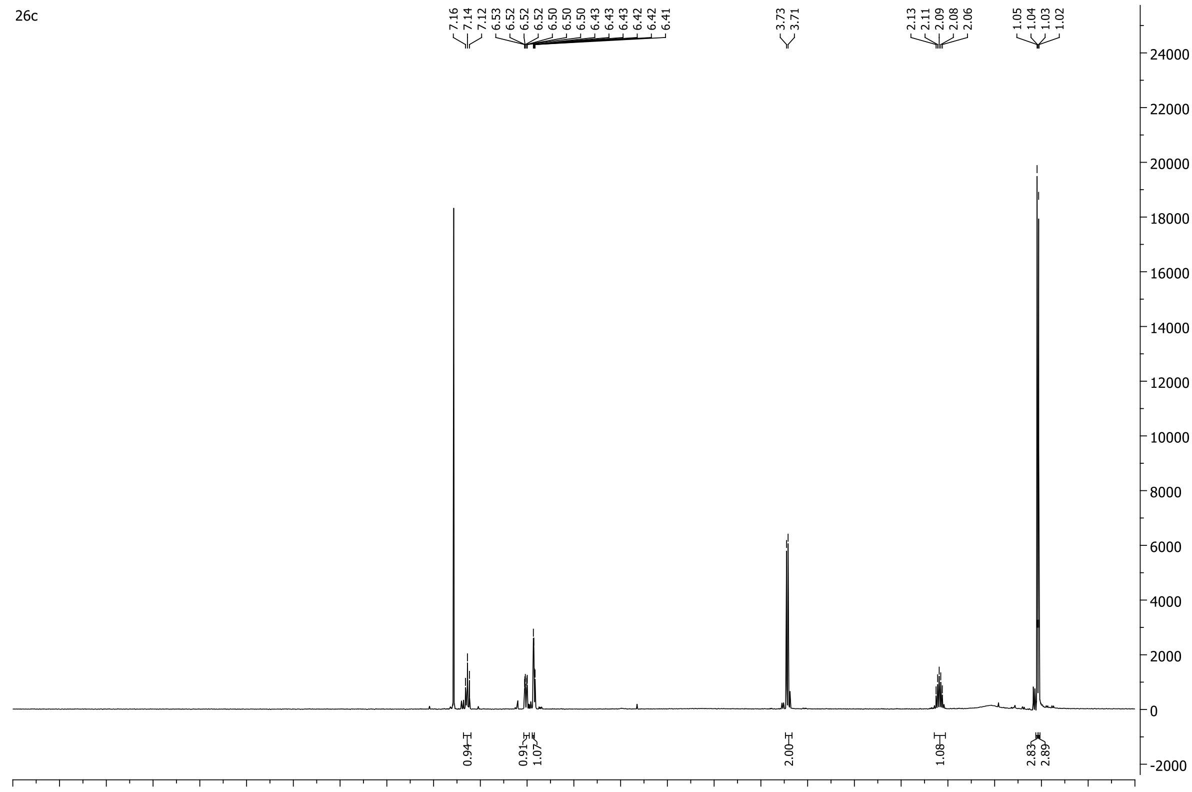

^{1}H NMR of 4-nitrophenol, **29c**

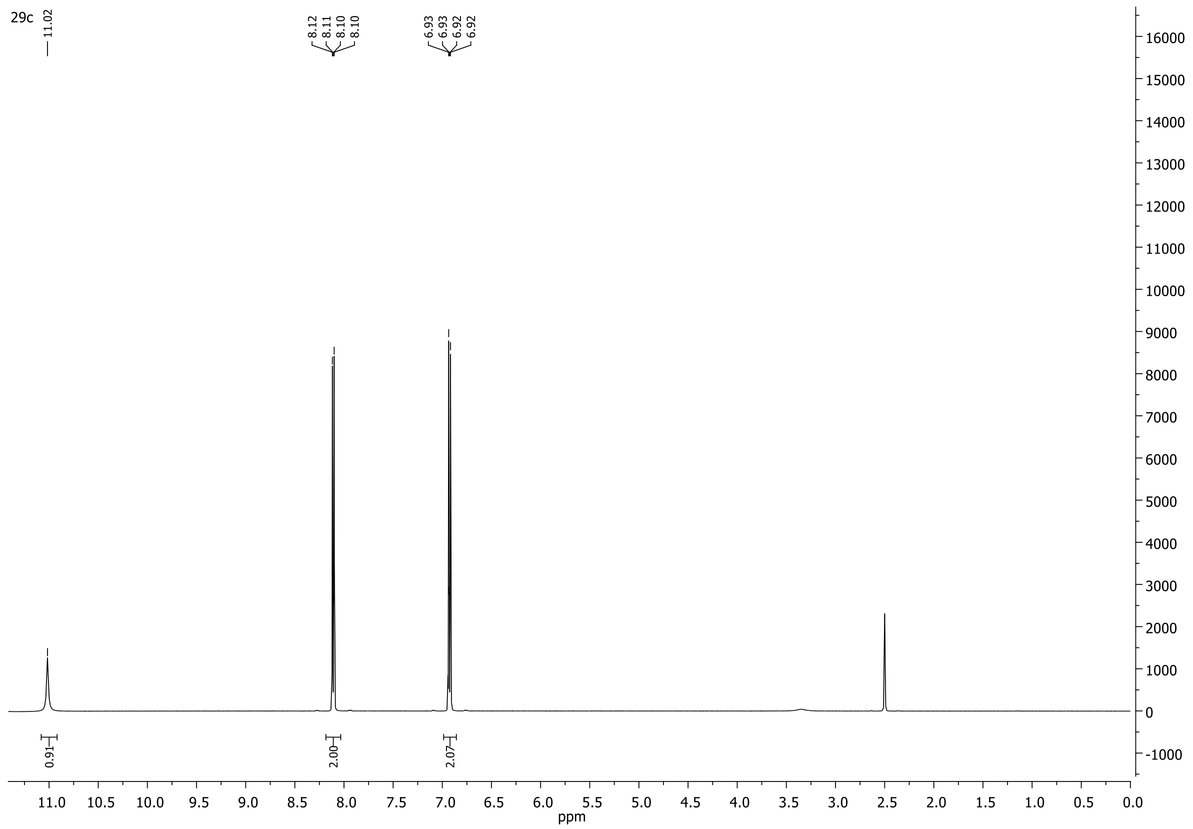

^{1}H NMR of 2,3-dihydrobenzo[b][1,4]dioxin-6-ol, **30c**

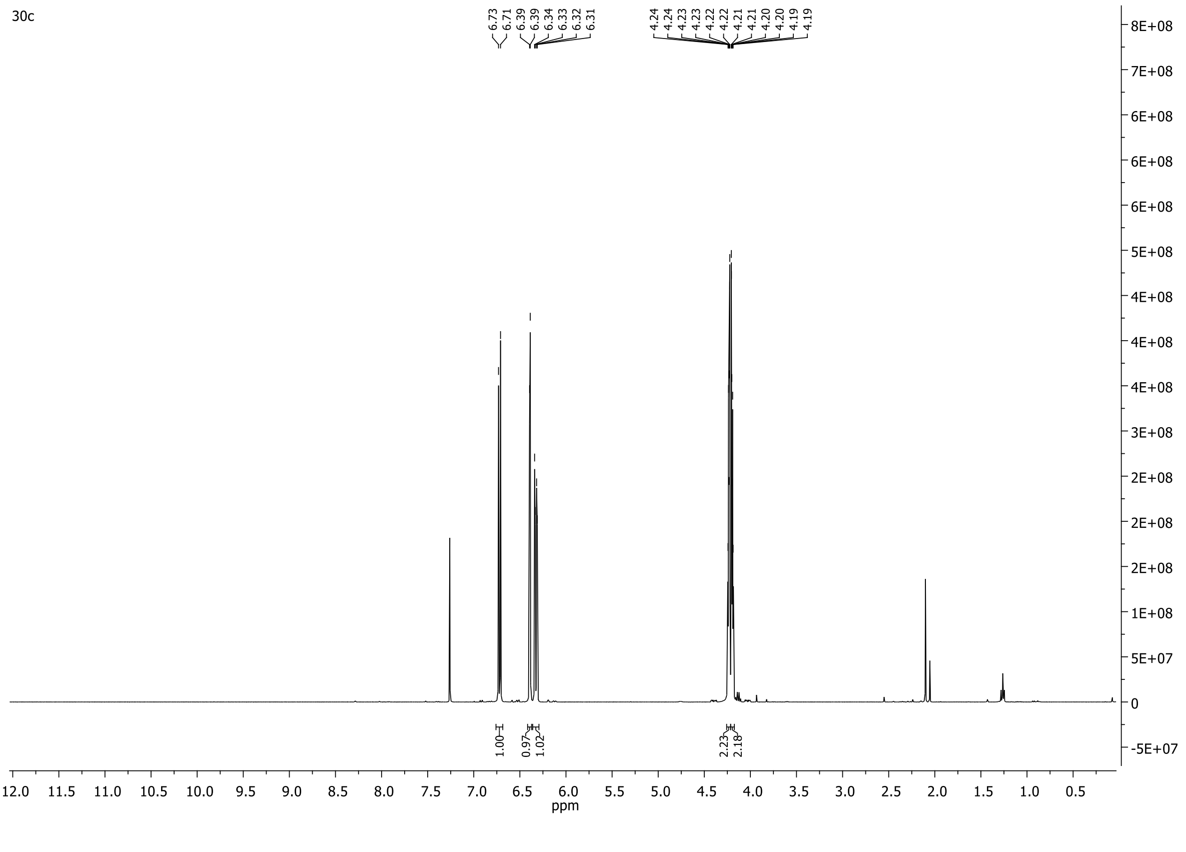

^{1}H NMR of pyridin-3-ol, **31c**

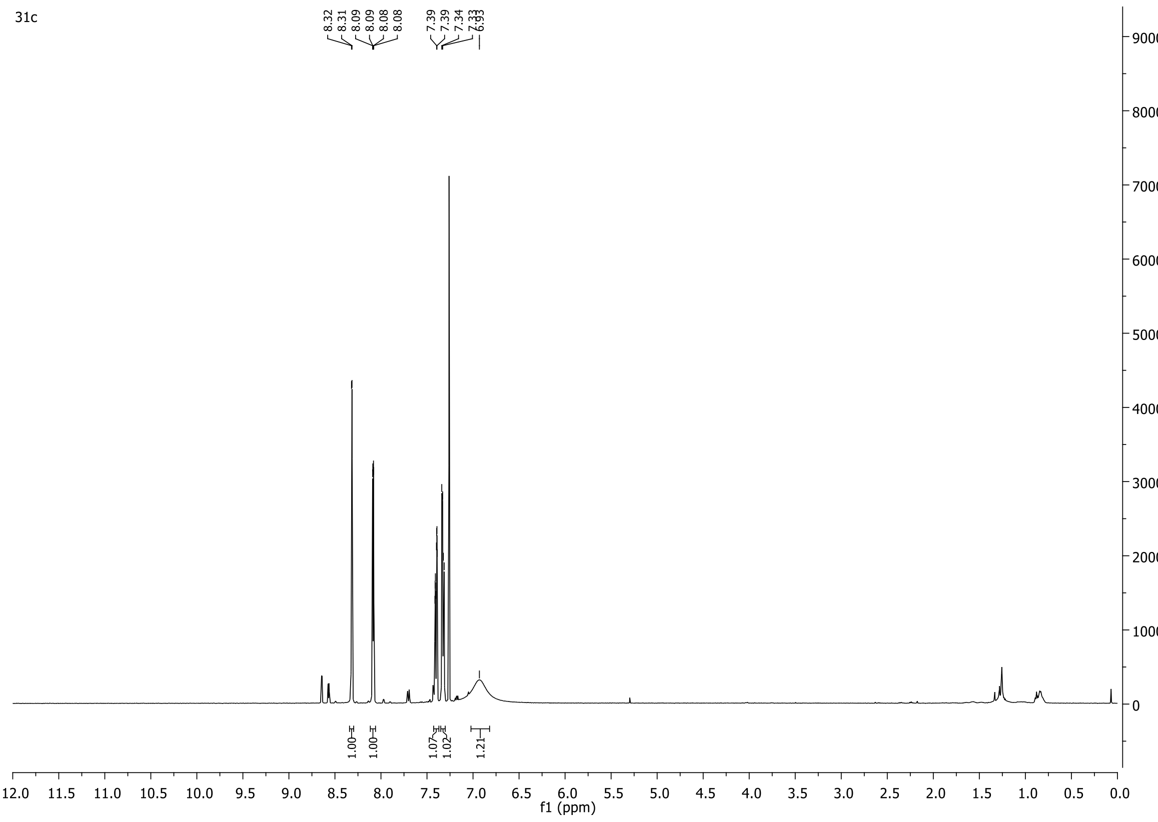

^{1}H NMR of 2-([1,1'-biphenyl]-4-yloxy)naphthalene, **32**

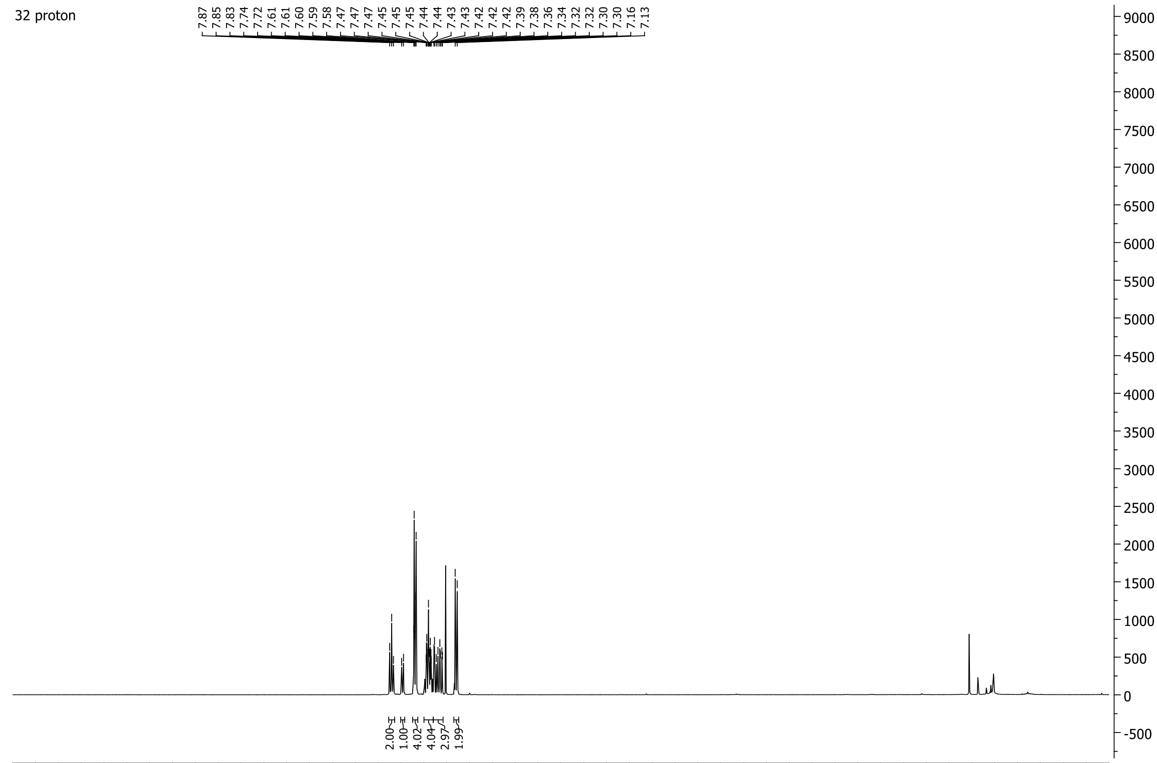

^{13}C NMR of 2-([1,1'-biphenyl]-4-yloxy)naphthalene, **32**

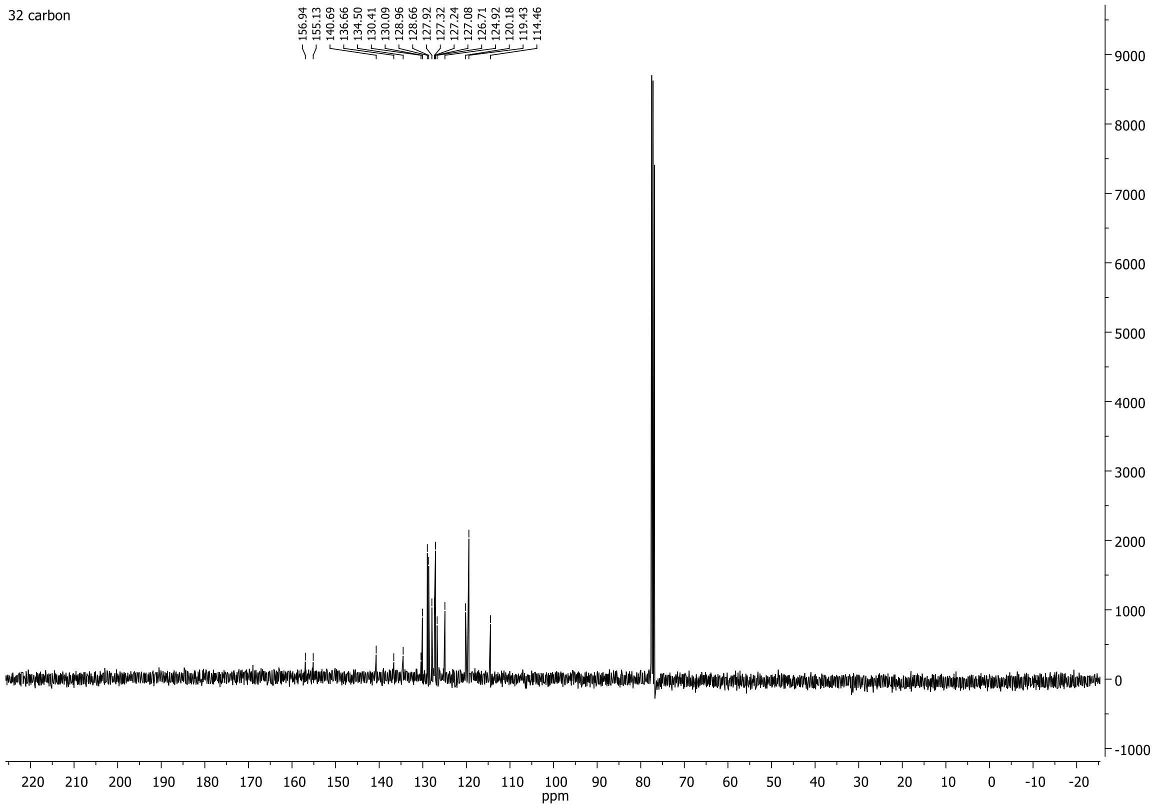

HRMS of 2-([1,1'-biphenyl]-4-yloxy)naphthalene, **32**

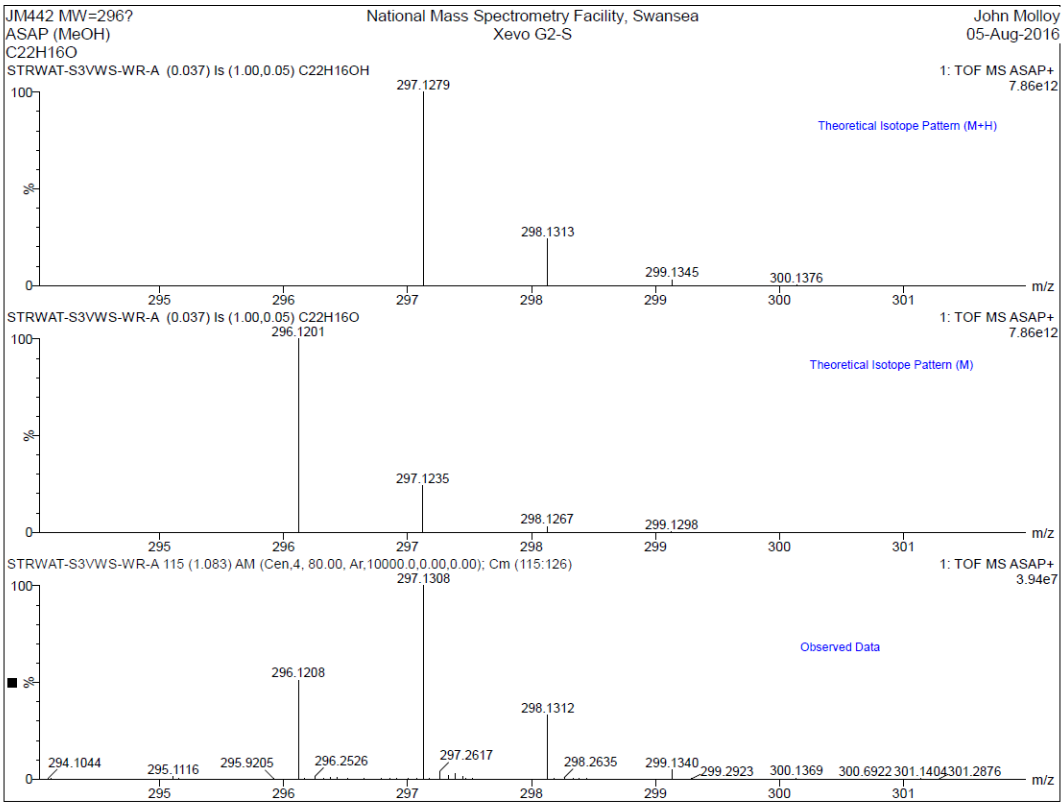

^{1}H NMR of methyl 4-(4-(trifluoromethyl)phenoxy)benzoate, **33**

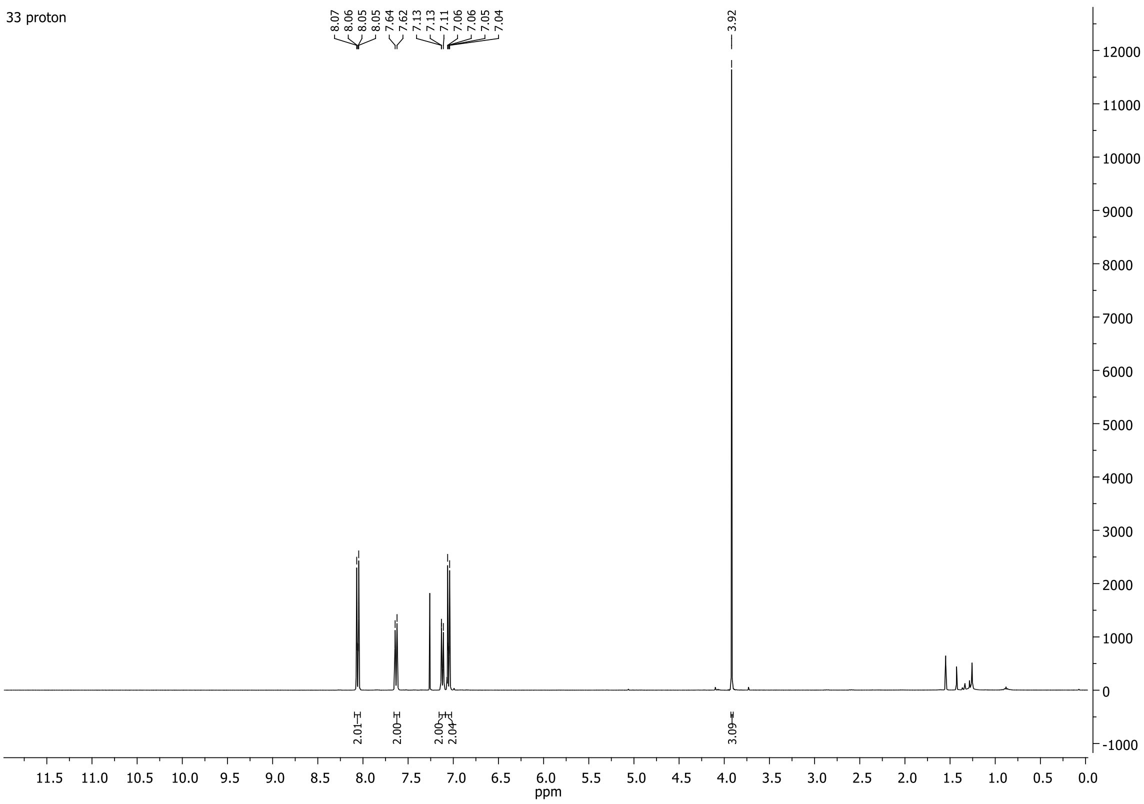

^{13}C NMR of methyl 4-(4-(trifluoromethyl)phenoxy)benzoate, **33**

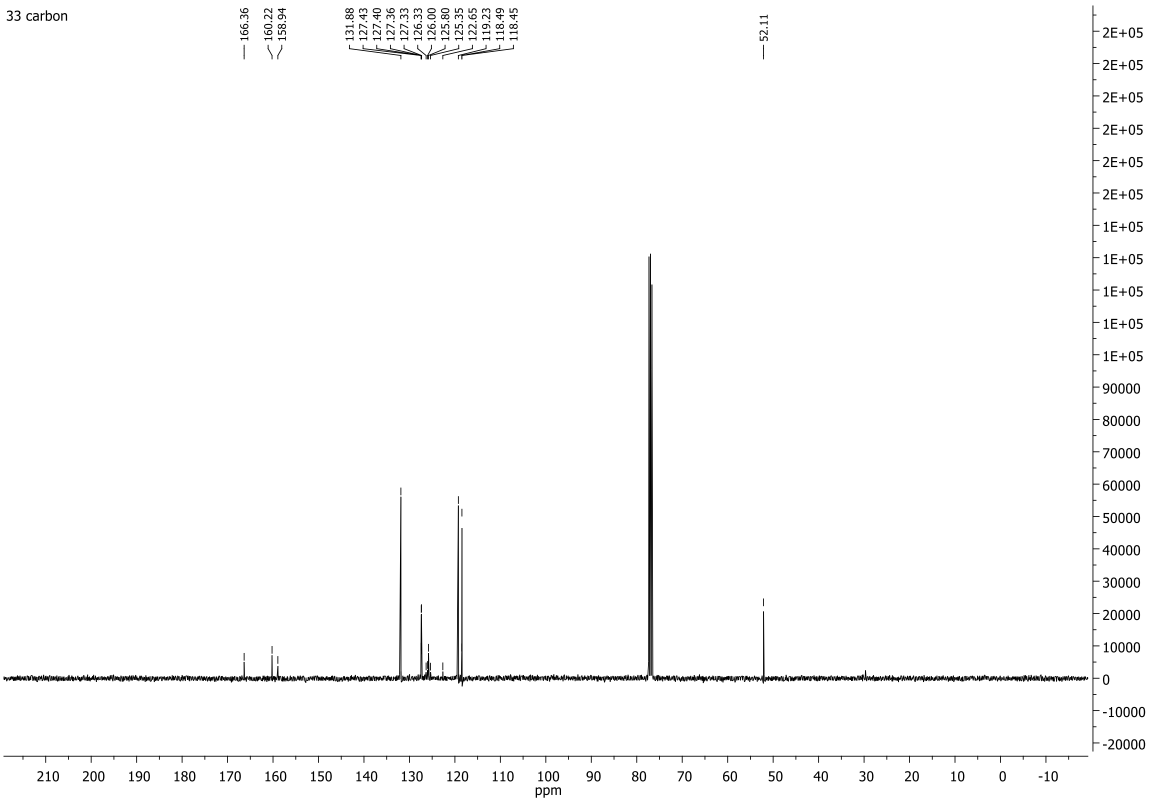

^{19}F NMR of methyl 4-(4-(trifluoromethyl)phenoxy)benzoate, **33**

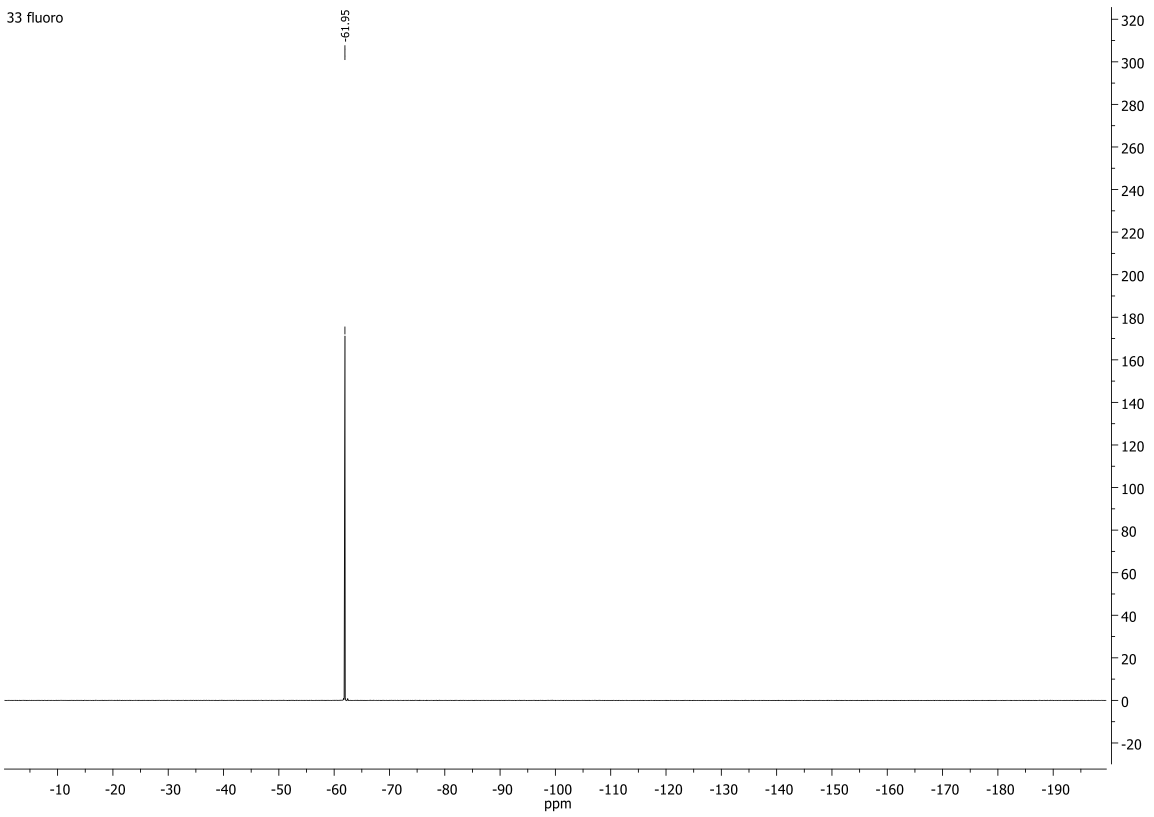

HRMS of methyl 4-(4-(trifluoromethyl)phenoxy)benzoate, **33**

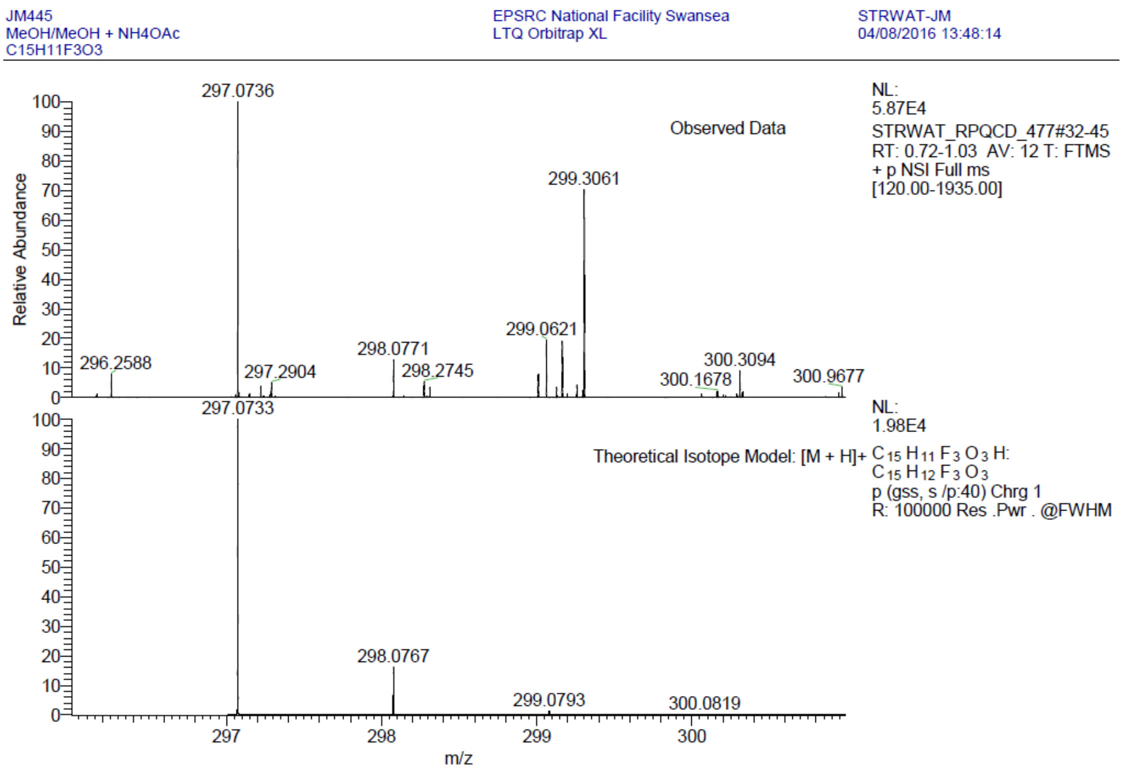

^{1}H NMR of 5-phenoxybenzofuran, **34**

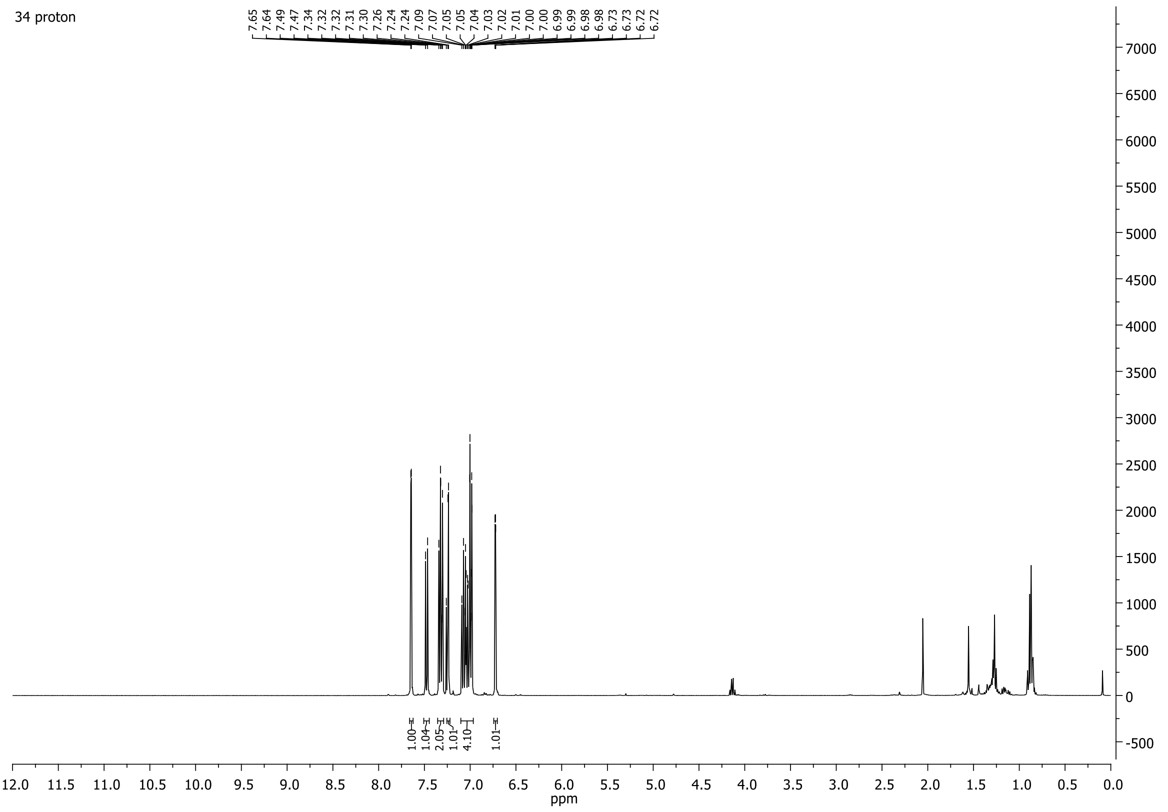

^{13}C NMR of 5-phenoxybenzofuran, **34**

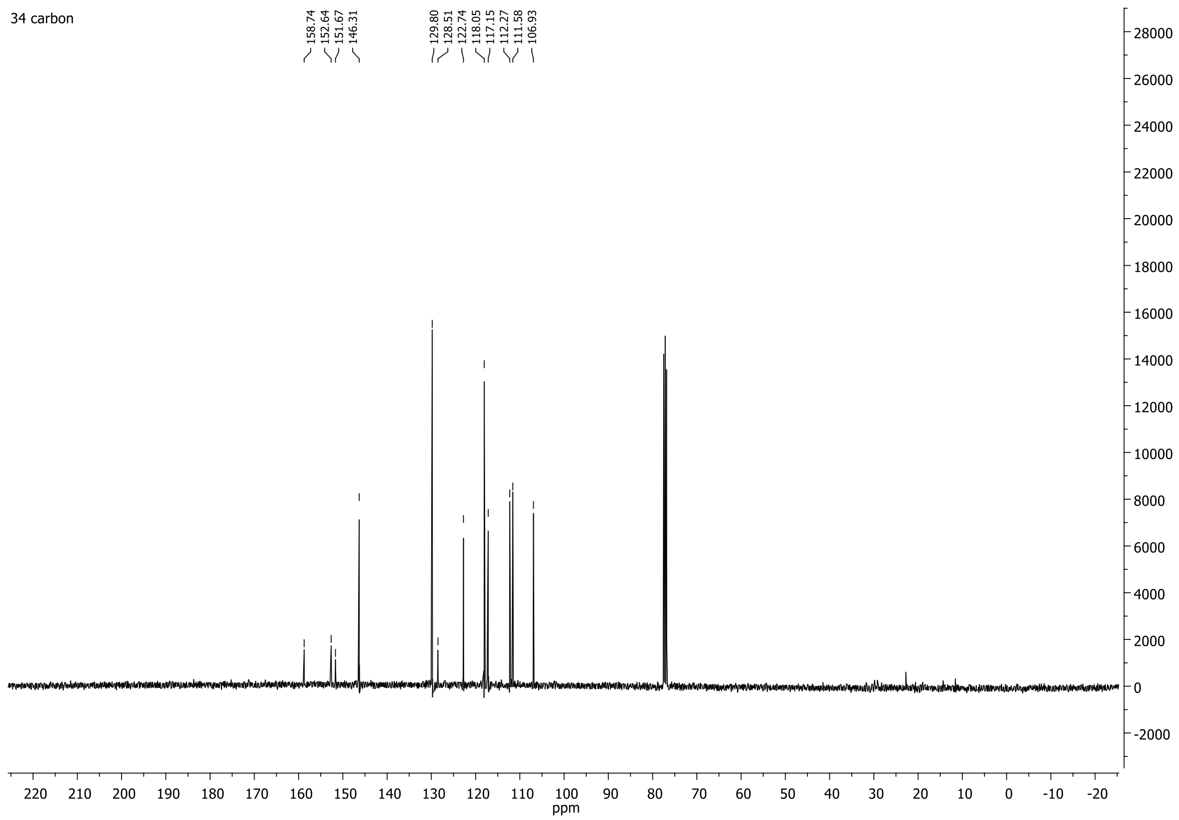

HRMS of 5-phenoxybenzofuran, **34**

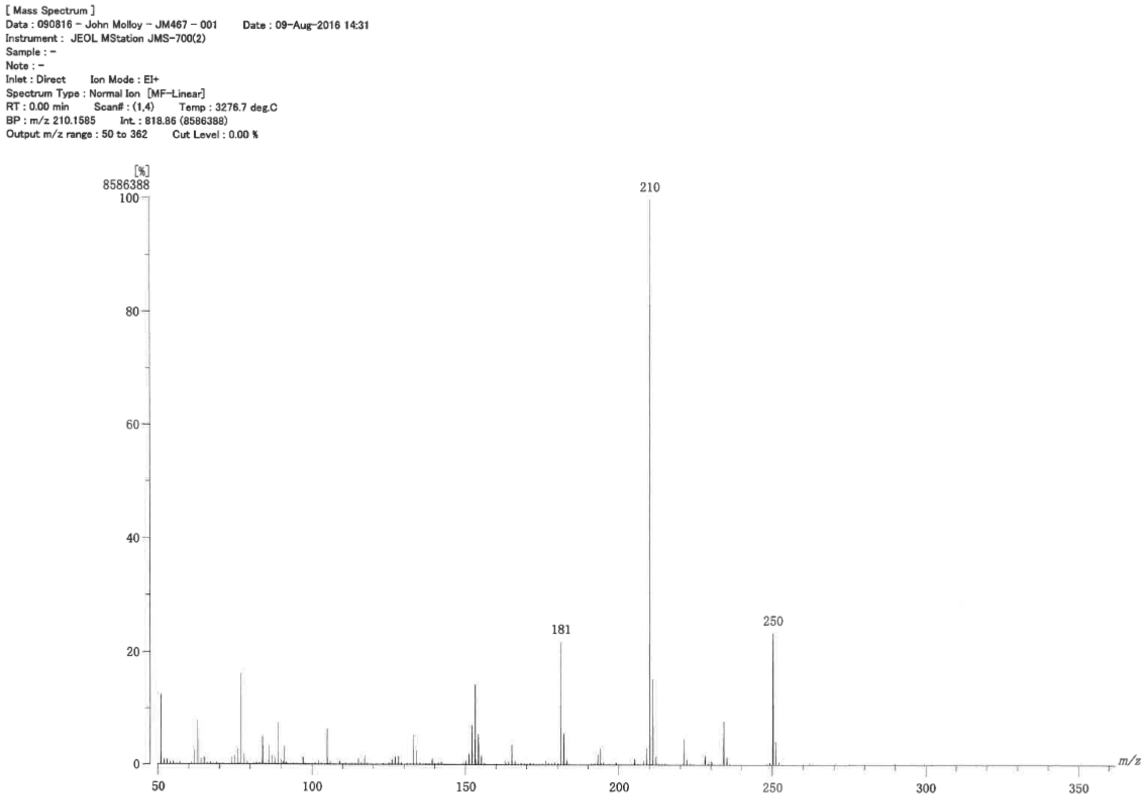

^{1}H NMR of 5-(4-fluorophenoxy)benzofuran, **35**

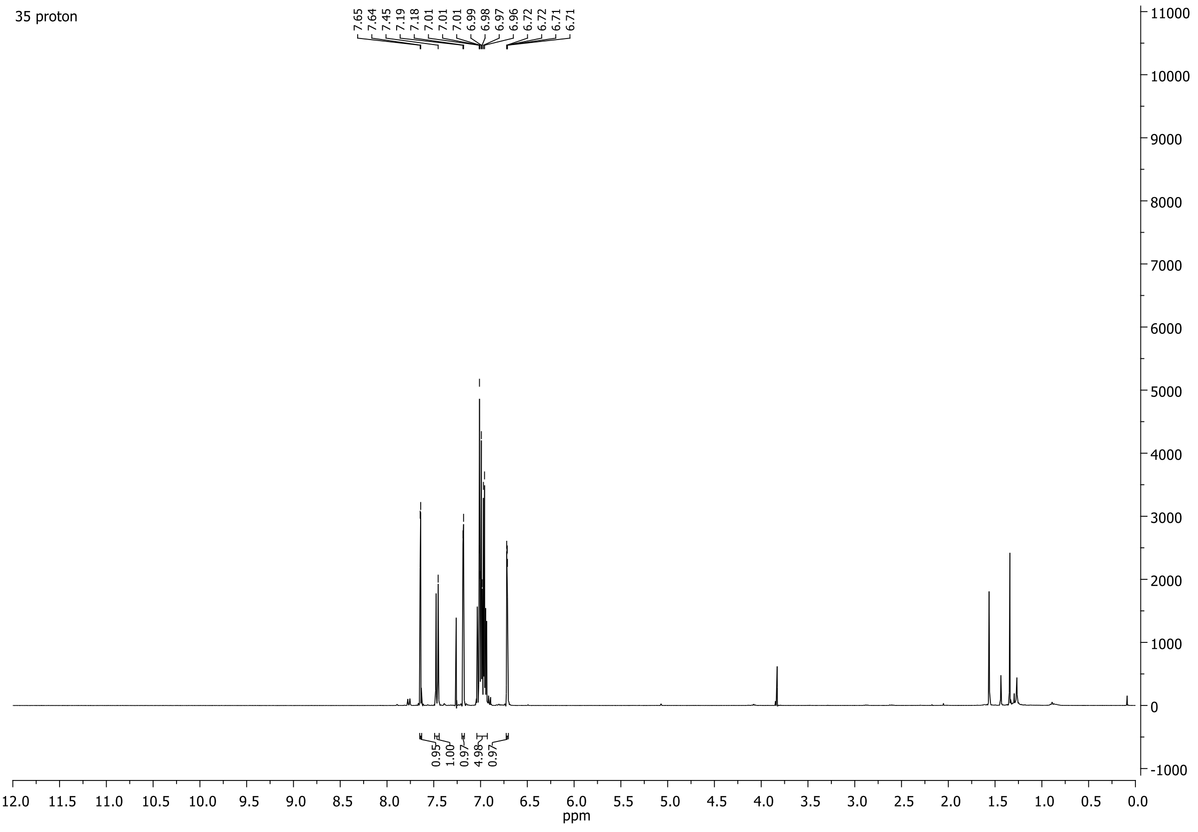

^{13}C NMR of 5-(4-fluorophenoxy)benzofuran, **35**

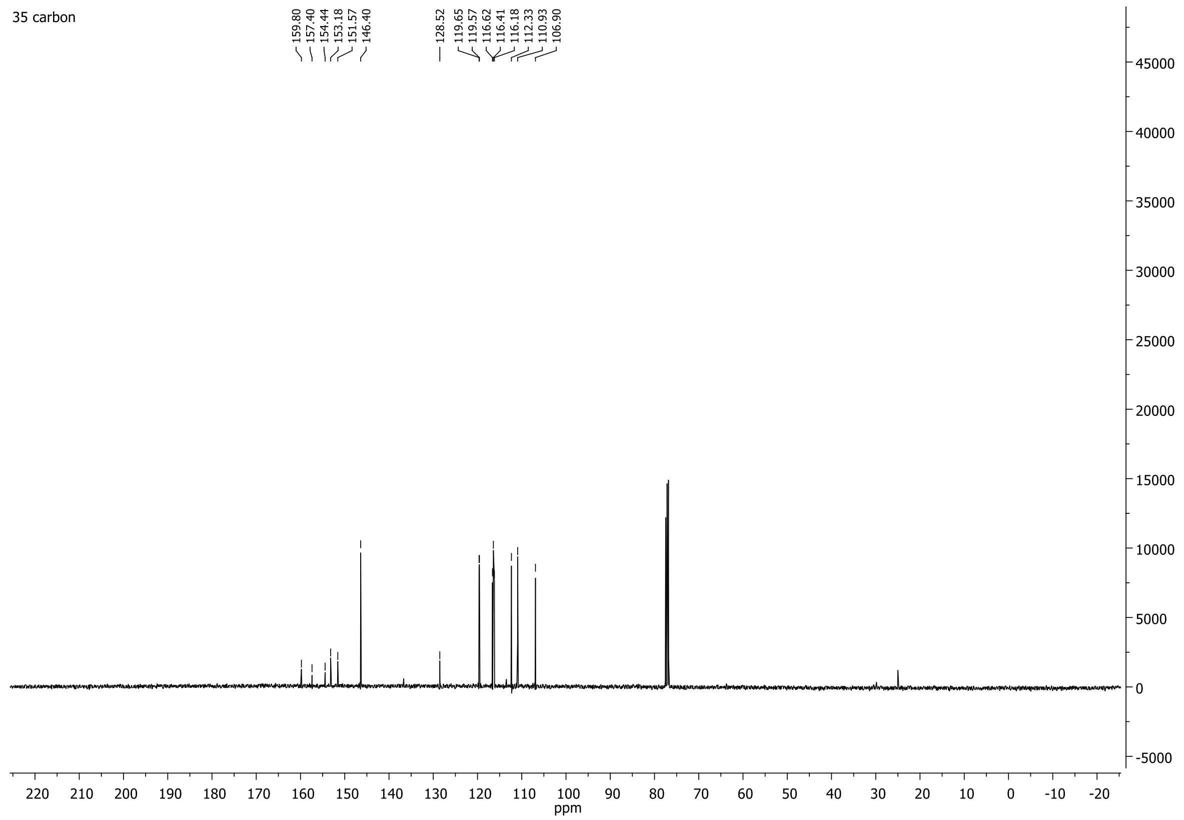

^{19}F NMR of 5-(4-fluorophenoxy)benzofuran, **35**

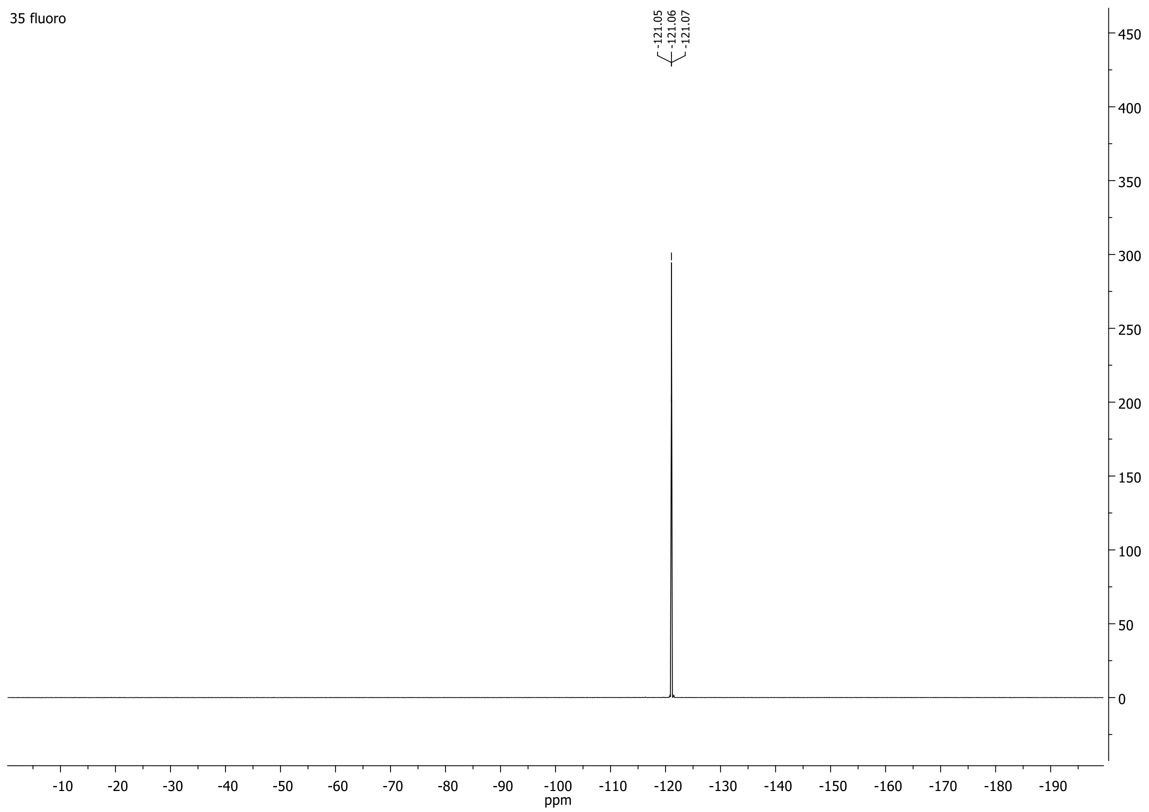

HRMS of 5-(4-fluorophenoxy)benzofuran, **35**

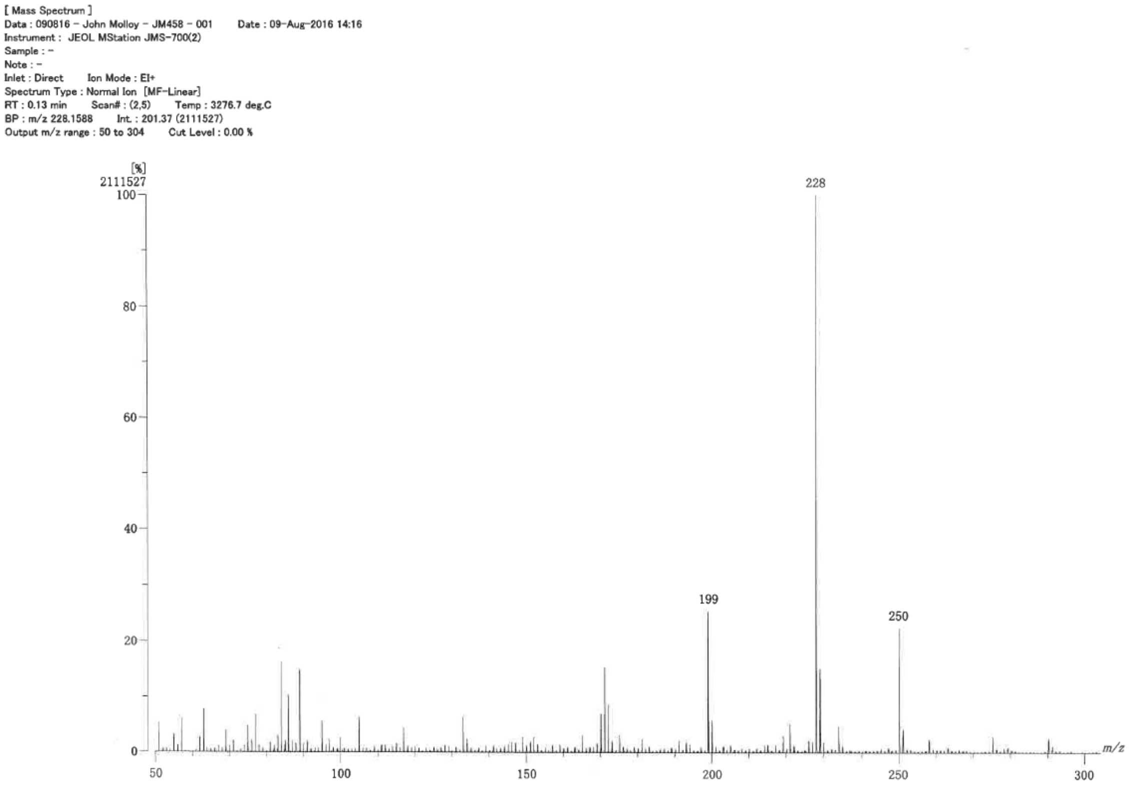

^{1}H NMR of 1,2,3-trimethoxy-5-(4-methoxyphenoxy)benzene, **36**

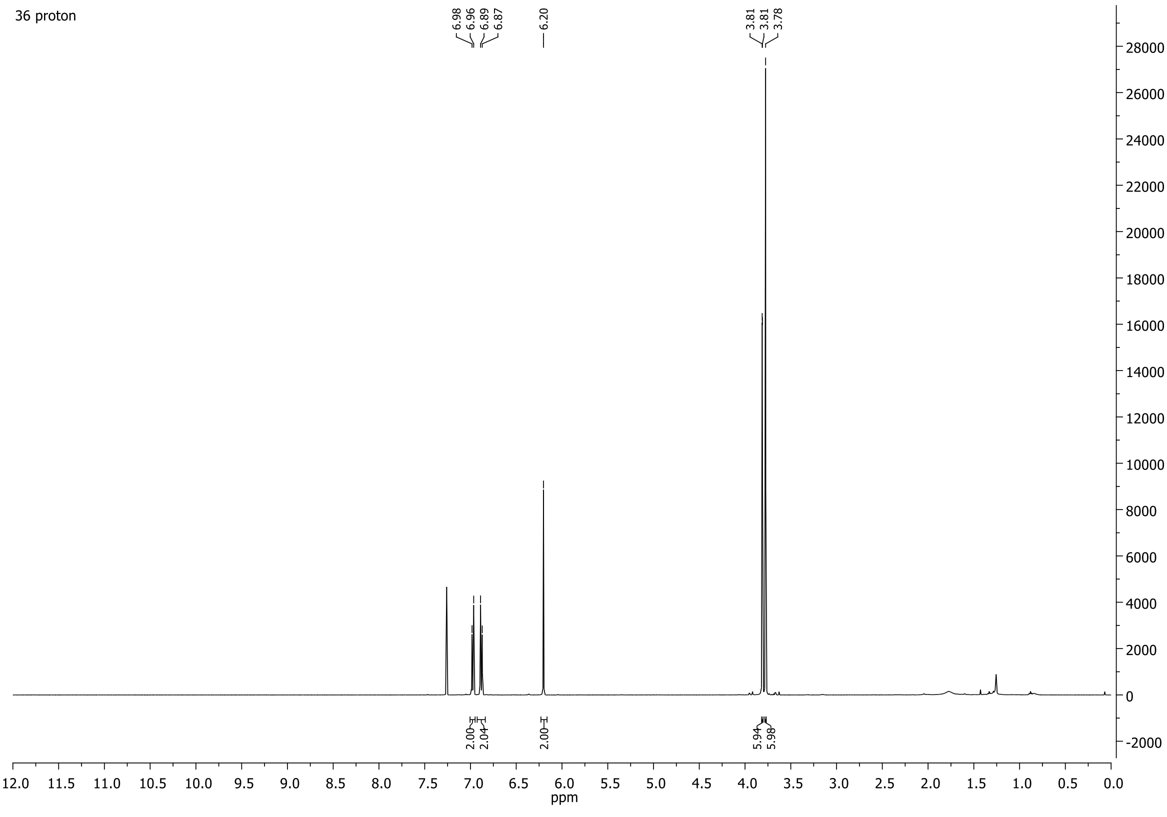

^{13}C NMR of 1,2,3-trimethoxy-5-(4-methoxyphenoxy)benzene, **36**

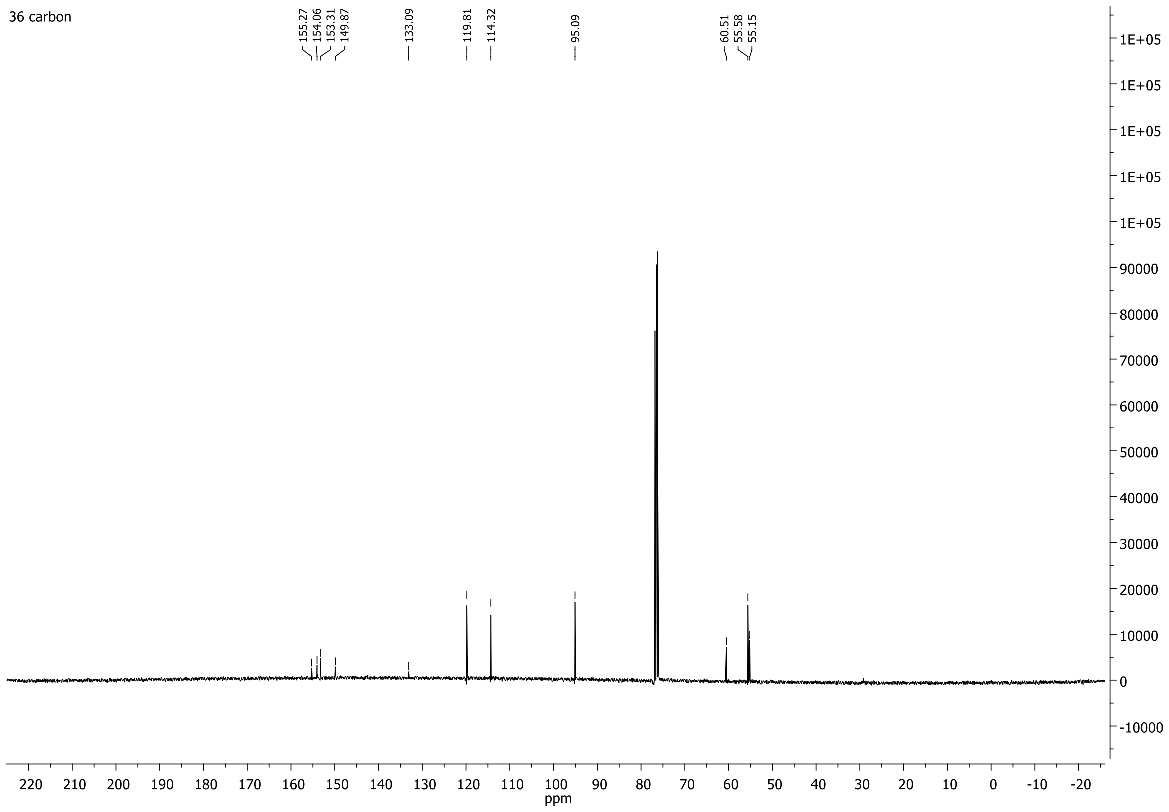

HRMS of 1,2,3-trimethoxy-5-(4-methoxyphenoxy)benzene, **36**

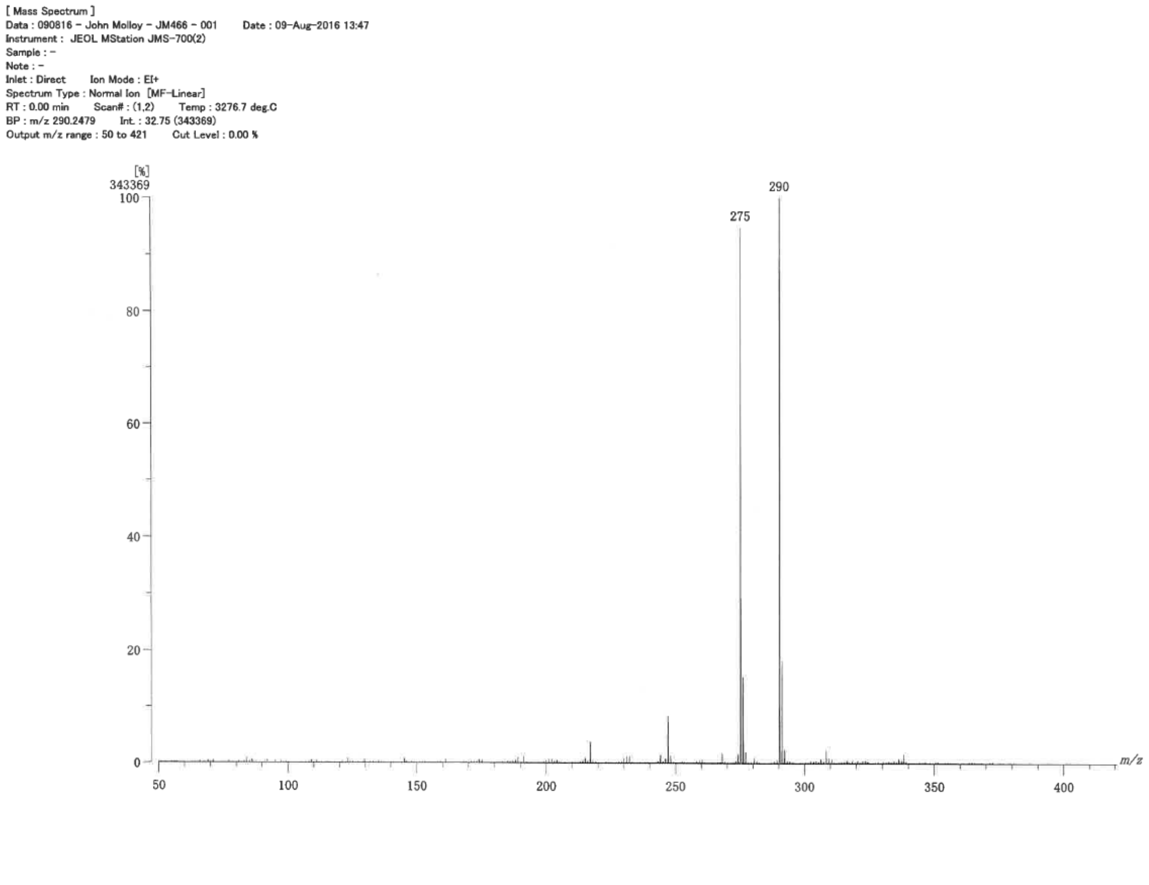

^{1}H NMR of 1-methyl-3-(4-(trifluoromethoxy)phenoxy)benzene, **37**

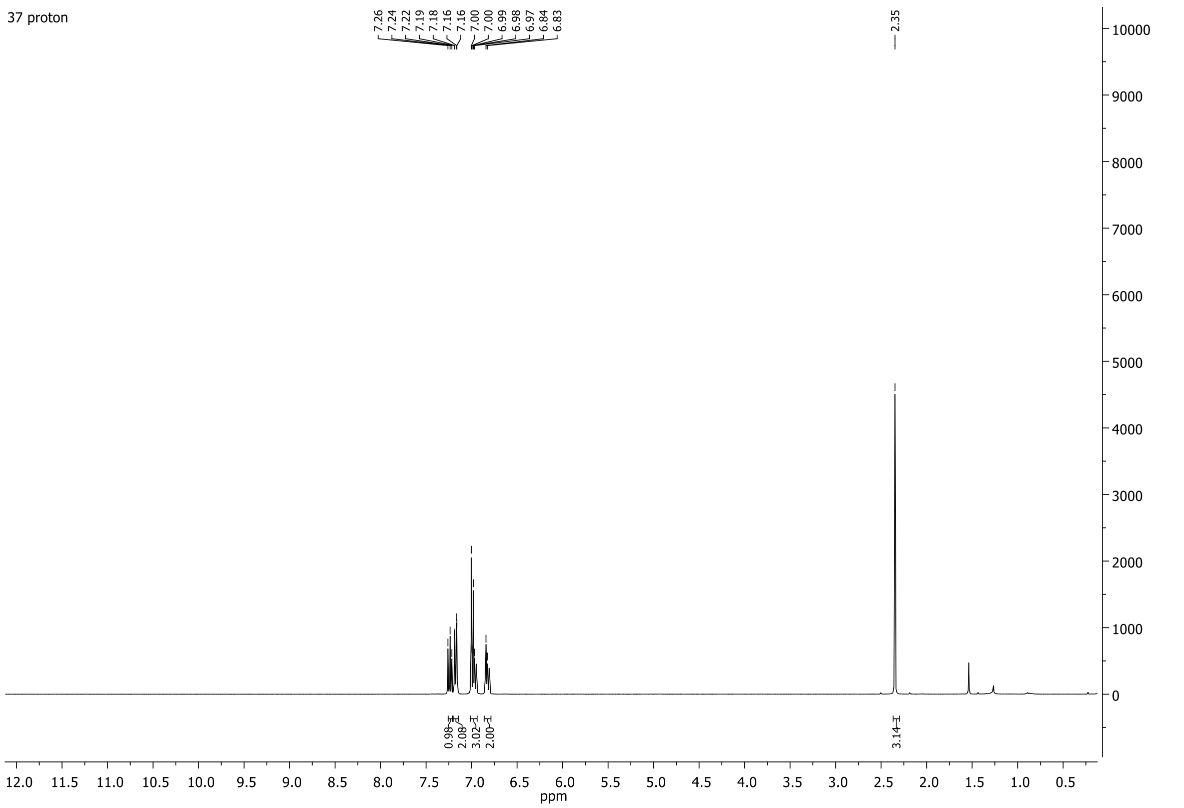

^{13}C NMR of 1-methyl-3-(4-(trifluoromethoxy)phenoxy)benzene, **37**

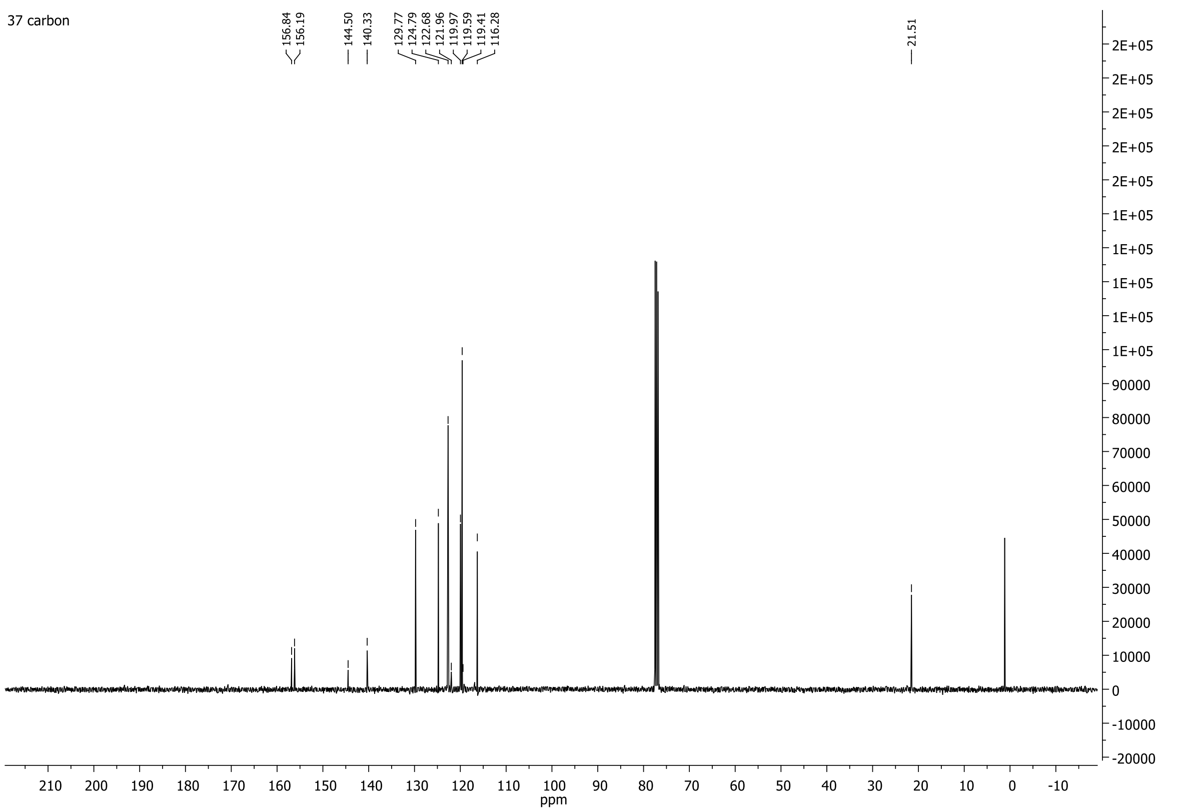

^{19}F NMR of 1-methyl-3-(4-(trifluoromethoxy)phenoxy)benzene, **37**

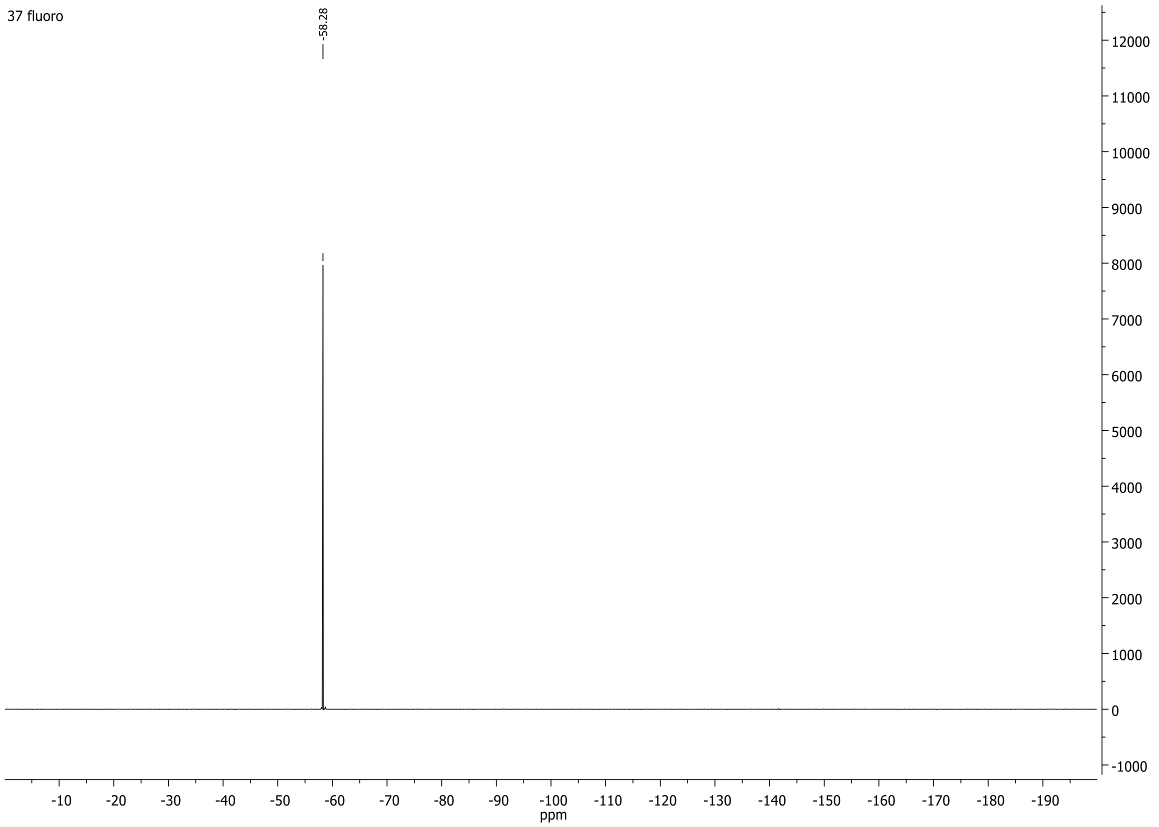

HRMS of 1-methyl-3-(4-(trifluoromethoxy)phenoxy)benzene, **37**

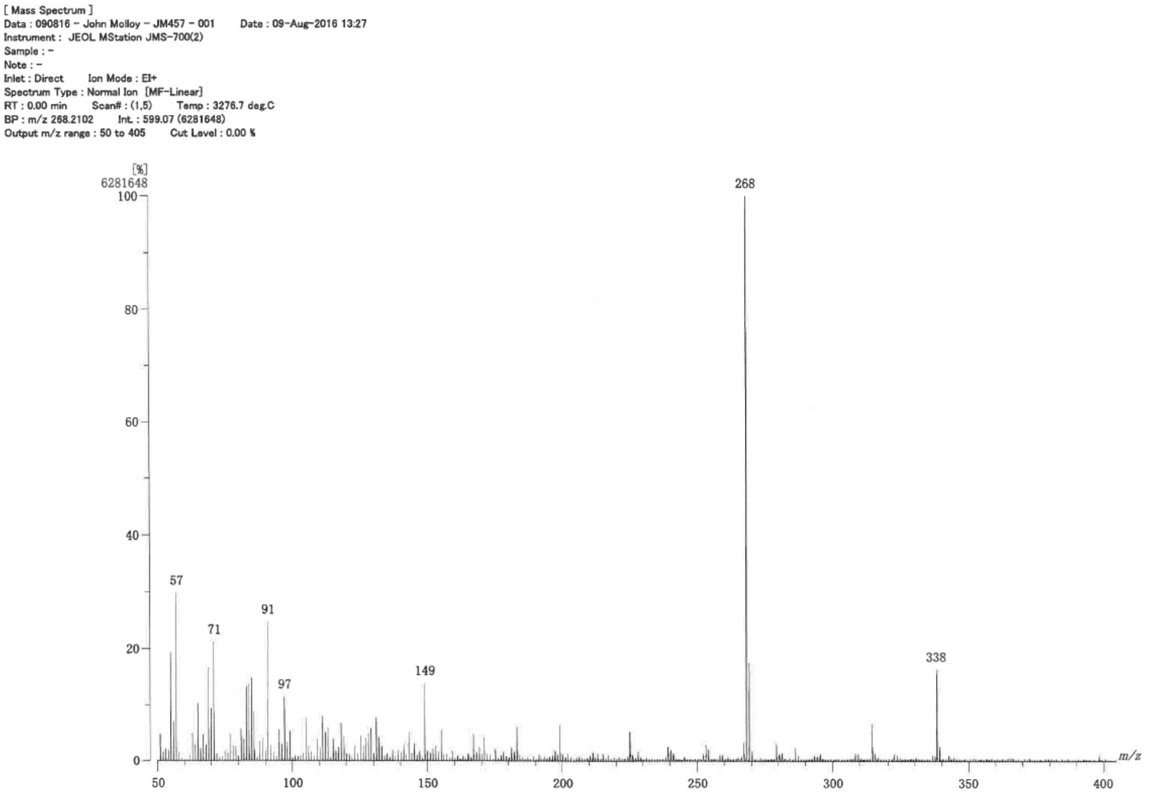

Supplement: Supplementary file 1 [file SC-008-C6SC04014D-s001.pdf]
